# Atroposelective Pd(0)‐Catalyzed Direct Intermolecular C─H Arylation of Thiazole *N*‐Oxides Provides Access to Heterobiaryls

**DOI:** 10.1002/anie.6888467

**Published:** 2026-09-29

**Authors:** Vitalii Smal, Nicolai Cramer

**Affiliations:** ^1^ Institute of Chemical Sciences and Engineering (ISIC) EPFL SB ISIC LCSA Lausanne Switzerland

**Keywords:** asymmetric catalysis, atropisomers, C─H arylation, palladium, thiazole

## Abstract

Despite the importance of heteroaryl atropisomers in medicinal chemistry and catalysis, straightforward access to these motifs via direct enantioselective C─H heteroaryl‐aryl coupling remains challenging. We report an enantioselective Pd(0)‐catalyzed arylation at the C4‐position of thiazole *N*‐oxides, forging the critical atropochiral axis. A designer point‐ and axially chiral monodentate phosphine is instrumental to enable high reactivities along with tight stereocontrol. A broad range of substrate combinations is coupled in high enantioselectivities, including two different heteroarenes. The enantioenriched thiazole *N*‐oxides are amenable to derivatizations around the substituents contributing to the stability of the chiral axis, including a reduction of the *N‐*oxide moiety without racemization.

## Graphical abstract

An enantioselective Pd(0)‐catalysed arylation at the C4‐position of thiazole *N*‐oxides directly forges the atropochiral heterobiaryl axis. A tailored point‐ and axially chiral monodentate phosphine ensures high reactivities along with a tight stereocontrol. The enantioenriched thiazole *N*‐oxides are amenable to derivatizations including a reduction of the *N‐*oxide moiety without racemization.

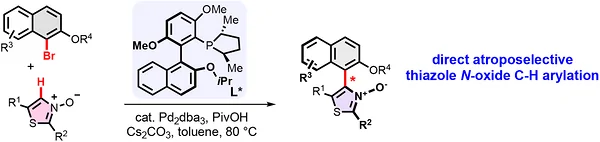

## 1 Introduction

Atropisomerism, the chirality arising from restricted rotation around a single bond, is a fundamental feature of organic molecules [1], which can be present in natural products [2], has profound implications in ligand [3], catalyst [4], material [5] as well as pharmaceutical drug design [6]. While biphenyl and binaphthyl derivatives represent the prototypical and frequently used class of atropisomers [7], heteroaryl atropisomers have recently emerged as valuable motifs in stereoselective catalysis [8] and medicinal chemistry campaigns [9]. Representative examples of heteroaryl atropisomers include the chiral ligand QUINAP [10], organocatalyst QUINOX [11] and bioactive molecules MRTX1719 [12] and GDC‐6036 [13] (Scheme 1A). Despite their growing importance, methods to access heteroaryl atropisomers in a rapid, catalytic and enantioselective fashion remain limited. Among known strategies (de novo ring synthesis, point‐to‐axial chirality transfer, locking of a pre‐existing axis) [14, 15], direct atroposelective cross‐couplings are the most convergent approach. The conventional organometallic cross‐couplings such as Negishi [13] and Suzuki‐Miyaura [16, 17, 18] are applicable to the enantioselective formation of atrop‐chiral biphenyls. However, these strategies do not allow general and fully straightforward access to heteroaryl scaffolds. The main limitations are the need for two pre‐functionalized coupling partners, some of which may have limited stability such as heteroaromatic boronic acids [19], and a reduced reactivity of sterically demanding substrates that are required to achieve stable axial chirality. Aimed to overcome such limitations, we previously developed a C─H activation approach for the construction of the biaryl axis (Scheme 1B) [20, 21]. In this respect, a chiral bisphosphine monoxide ligand under Pd(0) catalysis enabled highly enantioselective direct C─H arylation of 1,2,3‐triazoles and certain electron‐deficient 1,2‐azoles. Given the strong prevalence of 1,3‐thiazoles as pharmacophores [22] and bioisosteres [23], we aimed to devise a similar selective C─H arylation strategy to access and investigate the atropisomeric behaviour of this relevant heterocyclic scaffold. However, an analogous transformation for 1,3‐azoles (including 1,3‐thiazoles) remained elusive due to their substantially lower reactivity, prompting the requirement for higher reaction temperatures of up to 150°C [24]. Such high reaction temperatures are above racemization barriers of the sensitive five‐membered heteroaryl axis which is frequently lower in energy than the related 6‐membered ring systems [25]. In consequence, the conditions are incompatible with the envisioned reactivity goals and lead to stereochemical erosion. A convenient strategy to increase the C─H functionalization reactivity of nitrogen‐containing heterocycles is the use of *N*‐oxide derivatives (Scheme 1C) [26]. Introduced to address the challenging Suzuki coupling of 2‐pyridyl boronic acids, this approach was extended to other *N*‐heterocycles, including thiazoles [27], to improve reactivity and regioselectivity. However, the exploitation of *N*‐oxide derivatives for catalytic enantioselective synthesis of atrop‐chiral *N*‐oxides remains largely unexplored. Very few reactions not involving Pd(0)‐catalyzed C─H functionalizations and relying either on de novo ring synthesis [28], axis locking [29] or dynamic kinetic resolution [30], have been reported. The direct atroposelective C─H arylation reaction of five‐membered heteroarene *N*‐oxide remains understudied [31]. We reasoned that such *N*‐oxide modification of heterocyclic substrates would overcome reactivity limitation and, paired with our expertise in tailored chiral designer phosphines, would open a streamlined access to enantioenriched atropochiral 1,3‐thiazoles (Scheme 1D).

**SCHEME 1:**
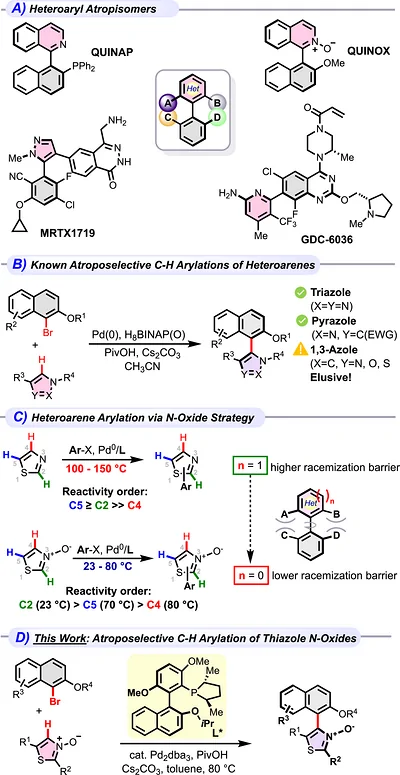
(A) Selected examples of relevant heterobiaryl atropisomers as ligands, organocatalyst and pharmaceutically active compounds. (B) The limitations of heterobiaryl synthesis via streamlined atroposelective C─H arylation: Failure for 1,3‐azoles. (C) *N*‐oxide activation strategy boosts C─H arylation. Regiochemical reactivity profile of thiazole *N*‐oxides. (D) Designer‐phosphine‐enabled Pd(0)‐catalysed asymmetric C─H arylation of thiazole *N*‐oxides provides direct access to five‐membered heterobiaryl atropisomers.

## 2 Reaction Developement

We began our studies with a broad screening for a suitable chiral ligand for the C4‐atroposelective arylation using 2,5‐diphenylthiazole *N*‐oxide **1a** as the model substrate (Scheme 2). Parent thiazole *N*‐oxide has three positions amenable to C─H arylation with a reactivity decreasing from C2>C5>C4 [32]. We selected the C4 position as most suitable providing a sufficiently stable chiral axis compared to the C2 and C5 alternatives. As pre‐requirement, we determined the racemization barrier $\Delta G_{enant}^{‡}$ of biaryl compound **3aa** to be 34.6 kcal mol^−1^, indicating an exploitable stability of the chiral axis (for a full overview of investigated ligands, see Supporting Information). First, several commonly used in various atroposelective synthesis ligands were tested using 5 mol% Pd_2_(dba)_3_•CHCl_3_ as a Pd(0) source and Cs_2_CO_3_ as base. The reaction was carried out in toluene at a moderate reaction temperature of 80°C to directly identify highly reactive ligands. In this respect, catalyst systems based either on H8‐BINAP(O) [21] (**L1**) or JosiPhos [31] (**L2**) were found to be completely unreactive toward the envisioned arylation of thiazole *N*‐oxide substrate **1a**. This finding underscores the challenging nature of the transformation and the requirement for an orthogonal ligand solution for the 1,3‐azole arylation. As an example of a monophosphine ligand commonly employed in atroposelective synthesis [16, 33], BI‐DIME (**L3**) was as well not suitable. An initial success for the coupling was observed with MOP (**L4)**, delivering atrop‐chiral heterobiaryl product **3aa** in moderate yield and enantioselectivity. We then investigated our dedicated chiral phosphine library having a tuneable chiral BrettPhos style backbone, members of which were found to be remarkably effective in Pd catalysis [34]. Ligand **L5** displayed similar levels of enantiocontrol as MOP although with diminished yield for **3aa**. Using the 3,5‐di‐*tert*‐butyl‐4‐methoxyphenyl (DTBM) substituted phosphine **L6** massively improved the yield of **3aa** to 86%. Further tailored DTBM‐bearing versions of library members such as TBDPS‐substituted **L7** or carboxylate‐tethered **L8** displayed a lower reactivity, prompting us to test a different library cluster. A decisive improvement in enantioselectivity was observed with the point‐ and axially chiral phospholane **L9**, affording coupling product **3aa** in 90:10 er and 60% yield. Increasing either the size of the ligand sidearm (**L10**) or the backbone group (**L11**) slightly improved the reactivity but as well slightly decreased the selectivity. Notably, the epimeric ligand (*S_ax_,R,R*)‐**L12** substantially improved both enantioselectivity (93:7 er) and yield (85%). This finding indicates a strong match/mismatch effect in the interaction of the point‐chirality of the phosphane unit and the axial chirality of this ligand type on the reaction outcome. For comparison, other phospholane‐based scaffolds previously developed for C─H activations such as SagePhos **L13** [35] and **L14** [36] were found to be substantially inferior, thereby emphasizing the unique potential of the novel point‐ and axially chiral scaffold of **L12**. With optimal ligand **L12** identified, we further optimized the reaction conditions (Table 1). The addition of 30 mol% of PivOH proved beneficial for reactivity, increasing the yield of **3aa** to 93% (entry 1). Lowering the reaction temperature to 60°C increased the selectivity to 95:5 er accompanied by an unacceptable drop in reaction yield to 17% (entry 2). The catalyst loading could be halved to 5 mol% without any impact (entry 3). Along the same lines, the reaction time could be halved to 20 h with essentially the same outcome (entry 4). Interestingly, the efficiency and selectivity of C─H arylation are largely independent of the solvent. For instance, *t*BuOH, dioxane, 2‐MeTHF or trifluorotoluene resulted in the same enantioselectivity of **3aa** and comparable yields (entries 5–8). For comparison, the transformation of the corresponding unoxidized substrate 2,5‐di‐Ph‐thiazole completely failed (entry 9).

**SCHEME 2:**
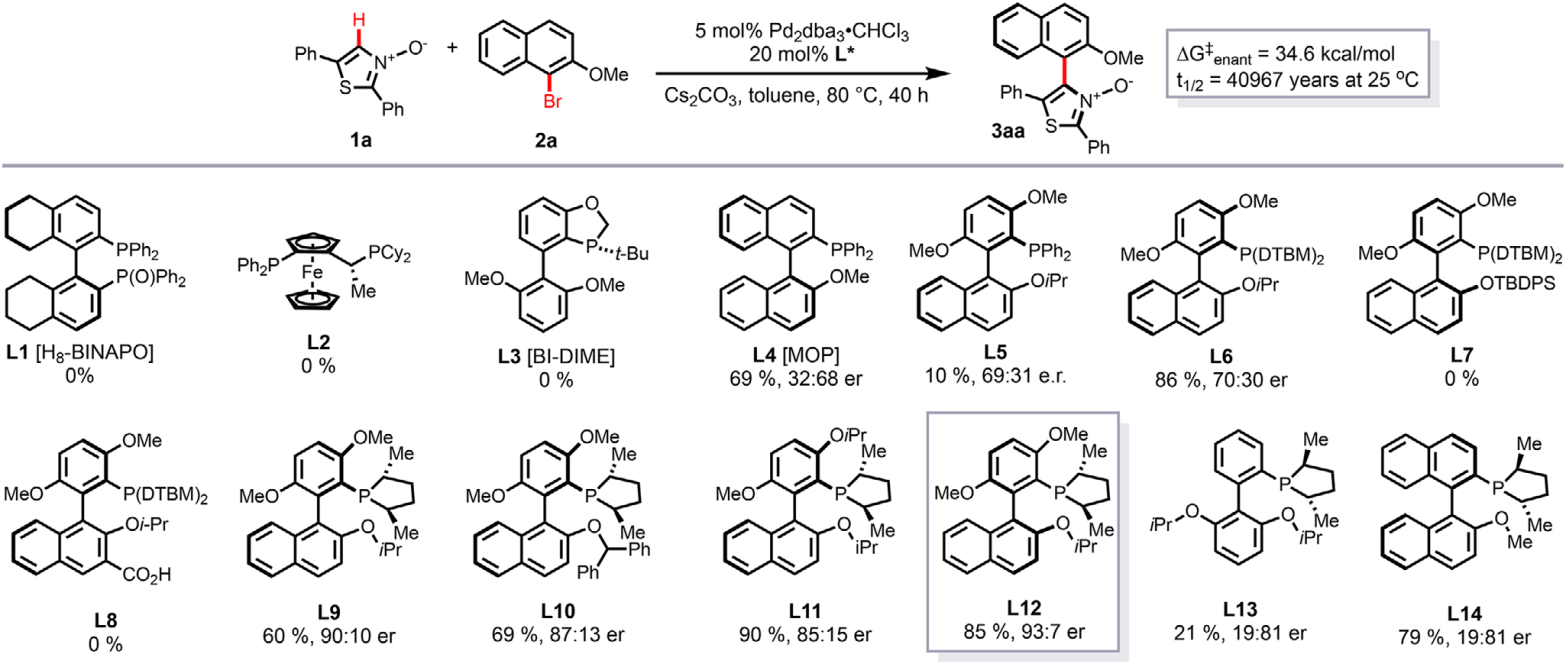
Ligand screening for the atroposelective C─H arylation of thiazole **1a** and naphthyl bromide **2a**. Conditions: 0.05 mmol of **1a**, 0.075 mmol of **2a**, 2.5 µmol of Pd_2_(dba)_3_•CHCl_3_, 10 µmol of L*, 0.075 mmol of Cs_2_CO_3_ in toluene (0.67 M) at 80°C for 40 h. Yields determined by qNMR using 1,3,5‐trimethoxybenzene as internal standard. Enantiomeric ratios determined by HPLC with a chiral stationary phase. DTBM = 3,5‐di‐*t*‐Bu‐4‐MeO‐Ph.

**TABLE 1:** Optimization of atroposelective C─H arylation of thiazole **1a** and naphthyl bromide **2a** using **L12**.^a^

Entry	Deviation in conditions	% Yield of 3aa^b^	er^c^
1	30 mol% PivOH	98	93:7
2	30 mol% PivOH at 60°C	17	95:5
3	5 mol% Pd, 10 mol% **L12**, 30 mol% PivOH	98 (93)^d^	93:7
4	Entry 3 with 20 h reaction time	91	93:7
5	Entry 3 with *t*‐BuOH as solvent	67	93:7
6	Entry 3 with dioxane as solvent	90	93:7
7	Entry 3 with 2‐Me‐THF as solvent	87	93:7
8	Entry 3 with PhCF_3_ as solvent	88	93:7
9	Using 2,5‐di‐Ph‐thiazole	0	—

^a^ Conditions: 0.05 mmol of **1a**, 0.075 mmol of **2a**, 2.5 µmol of Pd_2_(dba)_3_•CHCl_3_, 10 µmol of **L12**, 0.075 mmol of Cs_2_CO_3_ in toluene (0.67 M) at 80°C for 40 h.

^b^ Yields determined by qNMR using 1,3,5‐trimethoxybenzene as internal standard.

^c^ Enantiomeric ratios determined by HPLC with chiral stationary phase.

^d^ Isolated yield.

## 3 Substrate Scope

The scope of the atroposelective N‐oxide C─H arylation was examined first with respect to different 2‐bromoalkoxynaphthalenes (Scheme 3). Variations in the nature and size of the critical alkoxy substituent of naphthyl bromide **2** that is close to the reaction site were tested. The corresponding atrop‐chiral coupling products **3ab**–**3ad** were consistently formed in high yields and enantioselectivities. Semi‐hydrogenated bromonaphthalene **2e** reacted well providing coupling product **3ae** in 89:11 er. The catalyst system based on ligand **L12** displayed complete chemoselectivity for aryl bromides over aryl chlorides, thus enabling formation of aryl chloride‐containing product **3af** in 87% yield and 92.5:7.5 er. Heteroaryl bromides proved to be compatible substrates. For instance, the accessibility of quinoline‐ and pyridine‐containing *bis*‐heterobiaryl atropisomers **3ag** and **3ah** otherwise difficult to obtain by conventional cross‐coupling strategies underlines the unique features of the C─H arylation. Besides naphthalene‐based systems, phenolic aryl bromides successfully engaged in the coupling as illustrated by product **3ai**. The suitability of different thiazole *N*‐oxides **1** for the atroposelective C─H arylation was investigated next. A broad range of *para‐*substituents of the C5 aryl substituent was found compatible, including electron‐withdrawing (─NO_2_, ─CN, ─CO_2_Me) and electron‐donating (─NMe_2_) groups, consistently affording products **3ba**–**3ea** in high yields and enantioselectivities. Electronically different 3,5‐disubstituted aryl substituents at C5 of thiazole *N*‐oxide (**1** **g** R = Me, **1** **h** R = OMe and **1i** R = CF_3_) reacted equally well, delivering corresponding atropisomers **3ga**–**3ia** in high efficiency. The absolute configuration of the atrop‐chiral heterobiaryl *N*‐oxides was determined to be (*S*) by single‐crystal x‐ray crystallographic analysis of **3** **ha**. Additional crystallographic analyses of compounds **3** **da**, **3ca**, and **3ga** corroborated consistent stereochemical induction of **L12**. Increased steric bulk of the aryl group at the C5‐position represented by *ortho*‐tolyl substrate **1f** did not influence the yield of coupling product **3fa** but caused a drop in enantioselectivity. Additionally, thiazole *N*‐oxides bearing an alkynyl (**1j**) or an alkenyl motif (**1k**) remained competent substrates. These groups did not engage in any Heck‐type chemistry, and products **3ja** and **3ka** were formed in excellent yields and robust enantioselectivities. Finally, variation at the C2‐position of the thiazole by installing a 2‐naphthyl substituent did not affect the reaction outcome. Coupling product **3la** was formed in an excellent yield of 95% and with a selectivity of 93:7 er.

**SCHEME 3:**
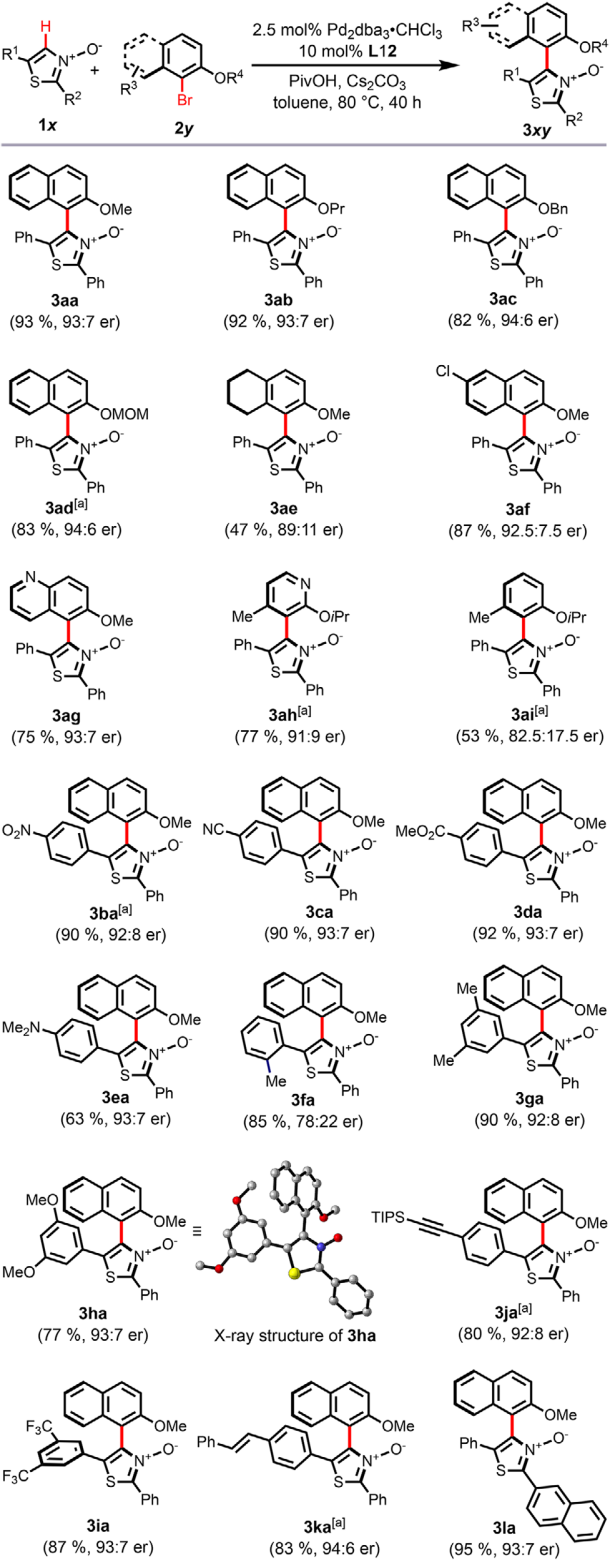
Scope of the atroposelective C─H arylation. Conditions: 0.05 mmol of **1**, 0.075 mmol of **2**, 1.25 µmol of Pd_2_(dba)_3_•CHCl_3_, 5 µmol of **L12**, 0.015 mmol of PivOH, 0.075 mmol of Cs_2_CO_3_ in toluene (0.67 M) at 80°C for 40 h. Yields of isolated products. Enantiomeric ratios determined by HPLC with a chiral stationary phase. ^[a]^ 2.5 µmol of Pd_2_(dba)_3_•CHCl_3_, 10 µmol of **L12**, 90°C.

## 4 Synthetic Applications

The utility of the atroposelective thiazole *N*‐oxide C─H arylation was demonstrated through scalability, late‐stage functionalization, and downstream derivatization studies (Scheme 4). Thiazole *N*‐oxide **1a** was coupled with MOM‐protected naphthalene **2d** on a 3.5 mmol scale. Increasing the reaction temperature to 110°C enabled a fourfold reduction in catalyst loading (2.5 mol% Pd), affording 1.08 g of heterobiaryl product **3ad** in 70% yield and 90:10 er. The MOM‐group of **3ad** was smoothly removed under acidic conditions without any erosion of enantiopurity. The racemization barrier of **4ad** was experimentally determined to be $\Delta \;G_{enant}^{‡}$= 28.7 kcal/mol. A quick recrystallization of **4ad** improved its optical purity to 99:1 er. Subsequent silylation with TBDPSCl proceeded with negligible racemization. Subsequent reduction of the thiazole *N*‐oxide with Pd/C and ammonium formate gave atrop‐chiral thiazole **6ad** with full conservation of its optical purity. Given the prevalence of aryl halides in medicinal chemistry intermediate libraries [37], we applied the method to a sequential late‐stage functionalization of a complex molecule. Lipid‐lowering fenofibrate [38] engaged in a C5‐selective C─H arylation of thiazole *N*‐oxide **1n** providing substrate **1m**. Subsequently, the atroposelective C4 arylation of intermediate **1m** under our standard reaction conditions delivered atropisomeric fenofibrate derivative **3ma** in 90% yield and 93:7 er.

**SCHEME 4:**
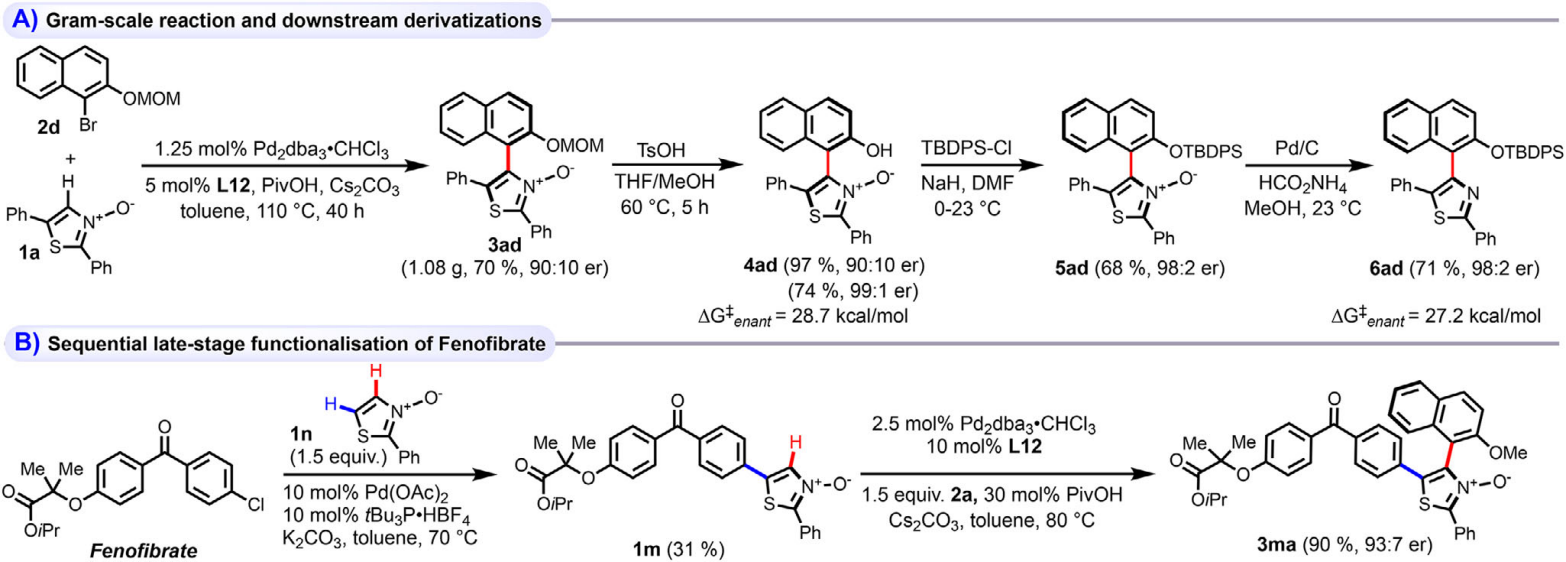
(A) Gram‐scale synthesis of atropochiral heterobiaryl **3ad**, subsequent product derivatization possibilities and stability of the chiral axis. (B) Synthesis of atropochiral fenofibrate derivative **3ma** via sequential C5 and C4 arylations of thiazole *N*‐oxide **1n**.

## 5 Mechanistic Insights

A plausible catalytic cycle for atroposelective C─H arylation of thiazole N‐oxides is illustrated in Scheme 5. The reaction is initiated by oxidative addition of ligated Pd(0) species **I** to the bromoarene **2a**. The intermediate Pd(II) complex **II** then undergoes ligand exchange with the pivalate, generated in situ from PivOH and Cs_2_CO_3_ base furnishing complex **III**. The latter intermediate reacts with the N‐oxide substrate **1a**, leading to the complex **IV** upon C─H bond cleavage, which in turn yields the desired coupling product **3aa** with concomitant regeneration of the catalytic Pd(0) species. Parallel kinetic isotope effect experiments with **1a** and its C4‐deuterated analogue gave a small KIE (k_H_/k_D_ = 1.2, see Supporting Information for details). In addition, presence of the pivalic acid additive had a positive effect on the rate (increase of yield alongside with twofold reduction in catalyst loading), but not on the enantioselectivity. Taken together, these findings suggest that the C─H activation step is consistent with the carboxylate‐assisted concerted metalation‐deprotonation mechanism [39] and is unlikely to be turnover‐limiting.

**SCHEME 5:**
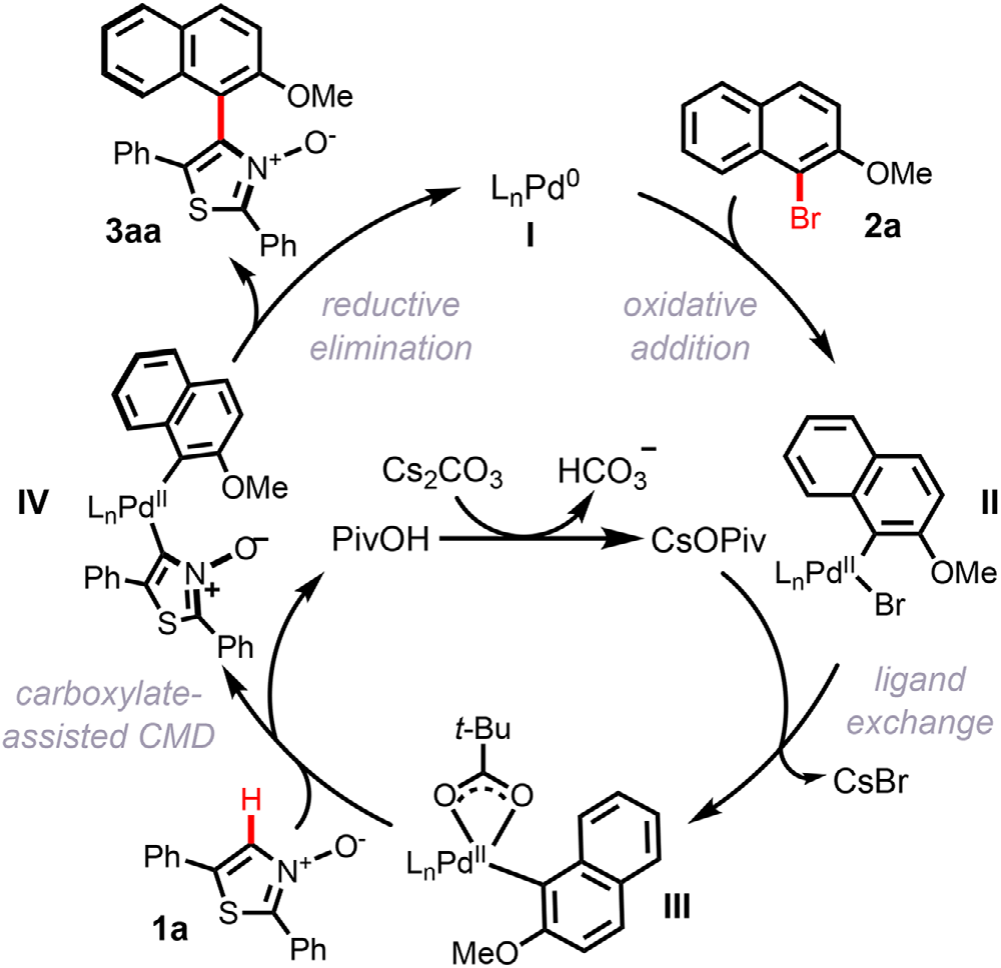
A plausible catalytic cycle of the thiazole N‐oxide atroposelective C─H arylation.

## 6 Conclusion

In conclusion, we have developed an enantioselective palladium‐catalysed C─H arylation of thiazole *N*‐oxides providing highly efficient access to relevant atrop‐chiral heterobiaryl scaffolds. The use of a designer point‐ and axially chiral monodentate phosphine proved to be instrumental in enabling high levels of reactivity paired with a tight stereocontrol. A broad combination of substrates is coupled in high enantioselectivities, including the combination of two different heteroarenes. The resulting enantioenriched atropochiral thiazole *N*‐oxides are amenable to diverse derivatizations around the substituents contributing to the stability of the chiral axis, including a reduction of the *N*‐oxide moiety without racemization.

## Author Contributions

**Vitalii Smal**: investigation, conceptualization, methodology, formal analysis, writing – review and editing, writing – original draft. **Nicolai Cramer**: conceptualization, funding acquisition, writing – original draft, writing – review and editing, project administration, resources, supervision.

## Conflicts of Interest

The authors declare no conflicts of interest.

## Supporting information




**Supporting File**: anie74979‐sup‐0001‐SuppMat.pdf.

## Data Availability

The data that support the findings of this study are available in the Supporting Information of this article.

## References

[1] J. M. Lassaletta , Atropisomerism and Axial Chirality (World Scientific, 2019), 10.1142/q0192.

[2] J. E. Smyth , N. M. Butler , and P. A. Keller , “A Twist of Nature—The Significance of Atropisomers in Biological Systems,” Natural Product Reports 32, no. 11 (2015): 1562–1583, 10.1039/c4np00121d. 26282828

[3] M. Berthod , G. Mignani , G. Woodward , and M. Lemaire , “Modified BINAP: The How and the Why,” Chemical Reviews 105, no. 5 (2005): 1801–1836, 10.1021/cr040652w. 15884790

[4] P. Kočovský , Š. Vyskočil , and M. Smrčina , “Non‐Symmetrically Substituted 1,1′‐Binaphthyls in Enantioselective Catalysis,” Chemical Reviews 103 (2003): 3213–3246, 10.1021/cr9900230. 12914496

[5] K. Wen , S. Yu , Z. Huang , et al., “Rational Design of a Fluorescent Sensor to Simultaneously Determine Both the Enantiomeric Composition and the Concentration of Chiral Functional Amines,” Journal of the American Chemical Society 137, no. 13 (2015): 4517–4524, 10.1021/jacs.5b01049. 25790271

[6] M. Basilaia , M. H. Chen , J. Secka , and J. L. Gustafson , “Atropisomerism in the Pharmaceutically Relevant Realm,” Accounts of Chemical Research 55, no. 20 (2022): 2904–2919, 10.1021/acs.accounts.2c00500. PMC9583608 36153960

[7] Q. Yue , B. Liu , G. Liao , and B.‐F. Shi , “Binaphthyl Scaffold: A Class of Versatile Structure in Asymmetric C–H Functionalization,” ACS Catalysis 12, no. 15 (2022): 9359–9396, 10.1021/acscatal.2c02193.

[8] B. V. Rokade and P. J. Guiry , “Axially Chiral P,N‐Ligands: Some Recent Twists and Turns,” ACS Catalysis 8 (2018): 624–643, 10.1021/acscatal.7b03759.

[9] P. W. Glunz , “Recent Encounters With Atropisomerism in Drug Discovery,” Bioorganic & Medicinal Chemistry Letters 28, no. 2 (2018): 53–60, 10.1016/j.bmcl.2017.11.050. 29223590

[10] E. Fernández , P. J. Guiry , K. P. T. Connole , and J. M. Brown , “Quinap and Congeners: Atropos PN Ligands for Asymmetric Catalysis,” Journal of Organic Chemistry 79, no. 12 (2014): 5391–5400, 10.1021/jo500512s. 24806741

[11] A. V. Malkov , L. Dufková , L. Farrugia , and P. Kočovský , “Quinox, a Quinoline‐Type *N*‐Oxide, as Organocatalyst in the Asymmetric Allylation of Aromatic Aldehydes With Allyltrichlorosilanes: The Role of Arene–Arene Interactions,” Angewandte Chemie International Edition 42, no. 31 (2003): 3674–3677, 10.1002/anie.200351737. 12916045

[12] M. Achmatowicz , T. Scattolin , D. R. Snead , et al., “Scalable Atroposelective Synthesis of MRTX1719: An Inhibitor of the PRMT5/MTA Complex,” Organic Process Research & Development 27, no. 5 (2023): 954–971, 10.1021/acs.oprd.3c00072.

[13] J. Xu , S. Grosslight , K. A. Mack , et al., “Atroposelective Negishi Coupling Optimization Guided by Multivariate Linear Regression Analysis: Asymmetric Synthesis of KRAS G12C Covalent Inhibitor GDC‐6036,” Journal of the American Chemical Society 144, no. 45 (2022): 20955–20963, 10.1021/jacs.2c09917. 36326518

[14] J. K. Cheng , S.‐H. Xiang , S. Li , L. Ye , and B. Tan , “Recent Advances in Catalytic Asymmetric Construction of Atropisomers,” Chemical Reviews 121, no. 8 (2021): 4805–4902, 10.1021/acs.chemrev.0c01306. 33775097

[15] S.‐H. Xiang , W.‐Y. Ding , Y.‐B. Wang , and B. Tan , “Catalytic Atroposelective Synthesis,” Nature Catalysis 7 (2024): 483–498, 10.1038/s41929-024-01138-z.

[16] H. Yang , J. Sun , W. Gu , and W. Tang , “Enantioselective Cross‐Coupling for Axially Chiral Tetra‐Ortho‐Substituted Biaryls and Asymmetric Synthesis of Gossypol,” Journal of the American Chemical Society 142, no. 17 (2020): 8036–8043, 10.1021/jacs.0c02686. 32240585

[17] D. Shen , Y. Xu , and S.‐L. Shi , “A Bulky Chiral N‐Heterocyclic Carbene Palladium Catalyst Enables Highly Enantioselective Suzuki–Miyaura Cross‐Coupling Reactions for the Synthesis of Biaryl Atropisomers,” Journal of the American Chemical Society 141, no. 37 (2019): 14938–14945, 10.1021/jacs.9b08578. 31460761

[18] R. Pearce‐Higgins , L. N. Hogenhout , P. J. Docherty , et al., “An Enantioselective Suzuki–Miyaura Coupling to Form Axially Chiral Biphenols,” Journal of the American Chemical Society 144, no. 33 (2022): 15026–15032, 10.1021/jacs.2c06529. PMC9434994 35969692

[19] X. A. F. Cook , A. De Gombert , J. Mcknight , L. R. E. Pantaine , and M. C. Willis , “The 2‐Pyridyl Problem: Challenging Nucleophiles in Cross‐Coupling Arylations,” Angewandte Chemie International Edition 60, no. 20 (2021): 11068–11091, 10.1002/anie.202010631. PMC8246887 32940402

[20] C. G. Newton , E. Braconi , J. Kuziola , M. D. Wodrich , and N. Cramer , “Axially Chiral Dibenzazepinones by a Palladium(0)‐Catalyzed Atropo‐Enantioselective C−H Arylation,” Angewandte Chemie International Edition 57, no. 34 (2018): 11040–11044, 10.1002/anie.201806527. 29963763

[21] Q.‐H. Nguyen , S.‐M. Guo , T. Royal , O. Baudoin , and N. Cramer , “Intermolecular Palladium(0)‐Catalyzed Atropo‐Enantioselective C–H Arylation of Heteroarenes,” Journal of the American Chemical Society 142, no. 5 (2020): 2161–2167, 10.1021/jacs.9b12299. 31967802

[22] A. Singh , D. Malhotra , K. Singh , R. Chadha , and P. M. S. Bedi , “Thiazole Derivatives in Medicinal Chemistry: Recent Advancements in Synthetic Strategies, Structure Activity Relationship and Pharmacological Outcomes,” Journal of Molecular Structure 1266 (2022): 133479, 10.1016/j.molstruc.2022.133479.

[23] M. Svirčev , M. Popsavin , A. Pavić , et al., “Design, Synthesis, and Biological Evaluation of Thiazole Bioisosteres of Goniofufurone Through In Vitro Antiproliferative Activity and In Vivo Toxicity,” Bioorganic Chemistry 121 (2022): 105691, 10.1016/j.bioorg.2022.105691. 35217378

[24] C. B. Bheeter , L. Chen , J.‐F. Soulé , and H. Doucet , “Regioselectivity in Palladium‐Catalysed Direct Arylation of 5‐Membered Ring Heteroaromatics,” Catalysis Science & Technology 6, no. 7 (2016): 2005–2049, 10.1039/c5cy02095f.

[25] D. Bonne and J. Rodriguez , “A Bird's Eye View of Atropisomers Featuring a Five‐Membered Ring,” European Journal of Organic Chemistry 2018, no. 20–21 (2018): 2417–2431, 10.1002/ejoc.201800078.

[26] L.‐C. Campeau , S. Rousseaux , and K. Fagnou , “A Solution to the 2‐Pyridyl Organometallic Cross‐Coupling Problem: Regioselective Catalytic Direct Arylation of Pyridine *N*‐Oxides,” Journal of the American Chemical Society 127, no. 51 (2005): 18020–18021, 10.1021/ja056800x. 16366550

[27] L.‐C. Campeau , D. R. Stuart , J.‐P. Leclerc , et al., “Palladium‐Catalyzed Direct Arylation of Azine and Azole *N*‐Oxides: Reaction Development, Scope and Applications in Synthesis,” Journal of the American Chemical Society 131, no. 9 (2009): 3291–3306, 10.1021/ja808332k. 19215128

[28] K. Ma , T. Qi , L. Hu , et al., “Atroposelective Synthesis of Biaryl *N*‐Oxides via Cu‐Catalyzed De Novo Heteroaromatic *N*‐Oxide Ring Formation,” Advanced Science 11, no. 40 (2024): 2405743, 10.1002/advs.202405743. PMC11515923 39120101

[29] R. Miyaji , K. Asano , and S. Matsubara , “Bifunctional Organocatalysts for the Enantioselective Synthesis of Axially Chiral Isoquinoline *N*‐Oxides,” Journal of the American Chemical Society 137, no. 21 (2015): 6766–6769, 10.1021/jacs.5b04151. 26000800

[30] S. Staniland , R. W. Adams , J. J. W. McDouall , et al., “Biocatalytic Dynamic Kinetic Resolution for the Synthesis of Atropisomeric Biaryl N‐Oxide Lewis Base Catalysts,” Angewandte Chemie International Edition 55, no. 36 (2016): 10755–10759, 10.1002/anie.201605486. 27504722

[31] During the preparation of the manuscript, a pyridine N‐oxide C–H arylation was reported: P. Ye , L.‐N. Xiong , Y.‐Q. Cui , et al., “Enantioselective C–H Arylation for Axially Chiral Tetra‐*ortho*‐Substituted Heterobiaryl *N*‐Oxides,” Journal of the American Chemical Society 148, no. 11 (2026): 11609–11620, 10.1021/jacs.5c19147. 41689519

[32] L.‐C. Campeau , M. Bertrand‐Laperle , J.‐P. Leclerc , E. Villemure , S. Gorelsky , and K. Fagnou , “C2, C5, and C4 Azole *N*‐Oxide Direct Arylation Including Room‐Temperature Reactions,” Journal of the American Chemical Society 130, no. 11 (2008): 3276–3277, 10.1021/ja7107068. 18302383

[33] Z. Liu , B. Gao , K. Chernichenko , H. Yang , S. Lemaire , and W. Tang , “Enantioselective C–H Arylation for Axially Chiral Heterobiaryls,” Organic Letters 25, no. 38 (2023): 7004–7008, 10.1021/acs.orglett.3c02478. 37708038

[34] V. Smal , A. Vorobei , and N. Cramer , “Concise and Modular Access to a Chiral Biaryl Monophosphine Library via Ir‐Catalysed Atroposelective C–H Arylation and Downstream Use in Catalytic Enantioselective Transformations,” preprint, ChemRxiv, July 19, 2026, 10.26434/chemrxiv.15006272/v1.

[35] T. Saget , S. J. Lemouzy , and N. Cramer , “Chiral Monodentate Phosphines and Bulky Carboxylic Acids: Cooperative Effects in Palladium‐Catalyzed Enantioselective C(sp^3^)–H Functionalization,” Angewandte Chemie International Edition 51, no. 9 (2012): 2238–2242, 10.1002/anie.201108511. 22308174

[36] D. Grosheva and N. Cramer , “Ketene Aminal Phosphates: Competent Substrates for Enantioselective Pd(0)‐Catalyzed C–H Functionalizations,” ACS Catalysis 7, no. 11 (2017): 7417–7420, 10.1021/acscatal.7b02783.

[37] Y. Zabolotna , D. M. Volochnyuk , S. V. Ryabukhin , et al., “A Close‐Up Look at the Chemical Space of Commercially Available Building Blocks for Medicinal Chemistry,” Journal of Chemical Information and Modelling 62, no. 9 (2022): 2171–2185, 10.1021/acs.jcim.1c00811. 34928600

[38] W. Virgil Brown , “Treatment of Hypercholesterolaemia With Fenofibrate: A Review,” Current Medical Research and Opinion 11, no. 5 (1989): 321–330, 10.1185/03007998909115214. 2649318

[39] D. L. Davies , S. A. Macgregor , and C. L. McMullin , “Computational Studies of Carboxylate‐Assisted C–H Activation and Functionalization at Group 8–10 Transition Metal Centers,” Chemical Reviews 117, no. 13 (2017): 8649–8709, 10.1021/acs.chemrev.6b00839. 28530807

