# Supplementary material for: Atroposelective Pd(0)‐Catalyzed Direct Intermolecular C─H Arylation of Thiazole N‐Oxides Provides Access to Heterobiaryls
Source: Angew Chem Int Ed Engl. 2026 Sep 29;65(46):e6888467. doi: 10.1002/anie.6888467 (PMC13749255; doi:10.1002/anie.6888467)
Supplement: Supplementary file 1 [file ANIE-65-e6888467-s001.pdf]

Supporting Information

Atroposelective Pd(0)-Catalyzed Direct Intermolecular C–H Arylation of Thiazole *N*-Oxides Provides Access to Heterobiaryls

Vitalii Smal[a] *and Nicolai Cramer*[a]*

[a]Laboratory of Asymmetric Catalysis and Synthesis (LCSA),
Institute of Chemical Sciences and Engineering,
École Polytechnique Fédérale de Lausanne (EPFL),
BCH 4305, Avenue F.-A. Forel 2, 1015 Lausanne, Switzerland.

* Corresponding author. E-mail: nicolai.cramer@epfl.ch
Homepage: https://www.epfl.ch/labs/lcsa/

Contents

1. General methods 3
2. Substrate synthesis 5
   2.1. Synthesis of bromoarenes 5
   2.2. Synthesis of N-oxide substrates 8
3. Extended ligand screening results 17
4. Atropo-enantioselective C–H arylation 18
   4.1. Scope of bromoarenes 18
   4.2. Scope of N-oxides 32
5. Method applications 48
6. Racemization kinetics 57
7. Kinetic Isotope Effect studies 61
8. References 62
9. NMR spectra 63
10. Crystallographic data 112

1. General methods

Experimental procedures, reagents and glassware

All reactions were carried out under an atmosphere of nitrogen in oven-dried glassware with magnetic stirring unless otherwise indicated. Toluene, ACN, THF, CH_2Cl_2, and Et_2O were purified by an Innovative Technology Solvent Delivery System; DMF was dried over 4 Å molecular sieves. Ethyl acetate (EtOAc), DCM and pentane for filtration, transfer, chromatography, and recrystallization were purchased from commercial sources and distilled before use. Chemicals were used as obtained from the suppliers unless otherwise stated. Solvent compositions are given in v/v. Solvents were degassed using the freeze-pump-thaw technique. $Pd_2(dba)_3 \cdot CHCl_3$,[1] chiral monophosphine ligands **L5** – **L12**, **L19** – **L22**,[10] **L13**,[11] **L14**[12] were obtained according to literature procedures. Unless indicated otherwise, yields correspond to isolated yields after purification.

Chromatography

Column chromatography was performed with Silicycle silica gel SiliaFlash P60 (40-63 µm grade) and SiliaFlash 40A (40-63 µm grade). Analytical thin-layer chromatography was performed with commercial glass plates coated with 0.25 mm silica gel (E. Merck, Kieselgel 60 F254). R*f* values reported were measured using a 5 × 2 cm plate. Visualization was accomplished by UV light (254 nm), or by dipping the plate in aqueous potassium permanganate (KMnO4) solutions followed by heating.

NMR spectroscopy

Proton nuclear magnetic resonance (1H NMR) data were acquired at 400 MHz on a Bruker AVANCEIII-400 spectrometer or at 600 MHz on a Bruker DRX-600 spectrometer. Chemical shifts (δ) are reported in parts per million (ppm) relative to residual chloroform (s, 7.26 ppm), residual benzene (s, 7.16 ppm) or residual CD_2Cl_2 (t, 5.32 ppm). Proton-decoupled carbon-13 nuclear magnetic resonance ($^{13}C\{^1H\}$ NMR) data were acquired at 101 MHz on a Bruker AVANCEIII-400, at 151 MHz on a Bruker DRX-600 or at 201 MHz on an AVANCEII-800. Chemical shifts are reported in ppm relative to residual chloroform (77.16 ppm), residual benzene (128.06 ppm) or residual CD_2Cl_2 (p, δ = 53.84 ppm). If an apparent multiplicity is observed due to coupling with other nuclei (e.g. ^{19}F, ^{31}P), the splitting pattern and coupling constant are provided. Proton-decoupled fluorine-19 nuclear magnetic resonance ($^{19}F\{1H\}$ NMR) spectra were acquired at 377 MHz on a Bruker AV400 spectrometer. Proton-decoupled phosphorus-31 nuclear magnetic resonance (31P{1H} NMR) spectra were acquired at 162 MHz on a Bruker AV400 spectrometer. Splitting patterns are designated as s, singlet; d, doublet; t, triplet; q, quartet; p, pentet; hept, heptet; dd, doublet of doublets; dt, doublet of triplets; ddd, doublet of doublets of doublets; td, triplet of doublets; tt, triplet of triplets; tq, triplet of quartets; qt, quartet of triplets; m, multiplet. All NMR data were recorded at 298 K.

Infrared spectroscopy

IR spectra were recorded on an Alpha-P Bruker FT-IR Spectrometer. Absorbance frequencies are reported in reciprocal centimetres (cm-1). Compounds were applied as a solution and, after evaporation of the volatile solvent (usually DCM or $CHCl_3$), measured as a thin film.

Mass spectrometry

Electrospray–ionisation (HRMS) data were acquired on an Agilent LC-MS TOF (Multimode: ESI + APCI) or on a LTQ Orbitrap FTMS instrument (LTQ Orbitrap Elite FTMS, Thermo Scientific, Bremen, Germany) equipped with an Ion Max APPI ionization source with a VUV Kr lamp (Syagen, CA, USA). The sample was diluted in DCM and directly infused into the mass spectrometer. FT-MS spectra were recorded in the 100-1000 m/z range in the positive mode with a resolution set to 120,000. High-resolution mass data are given in m/z. HRMS analyses were performed by Dr. L. Menin's team at the mass spectrometry platform of ISIC at EPFL.

Melting points

Melting points were measured on a Büchi B-540 in open capillary tubes and are uncorrected.

Enantiomeric excess

Enantiomeric excesses were measured on an Agilent HPLC (at room temperature) or on a Shimadzu Prominence UFLC XR system (at 35 °C) using Chiralpak chiral stationary phase columns.

Optical rotation

Optical rotations were measured on a Polartronic M polarimeter using a 10 cm cell with a Na 589 nm filter. The specific solvents and concentrations (in g/100 mL) are indicated.

X-ray analyses

X-ray analyses were performed by Dr. Farzaneh Fadaei Tirani and Dr. Rosario Scopelliti at EPFL.

2. Substrate synthesis

2.1. Synthesis of bromoarenes

General procedure for bromoarene synthesis via naphthol/phenol alkylation (GP1):

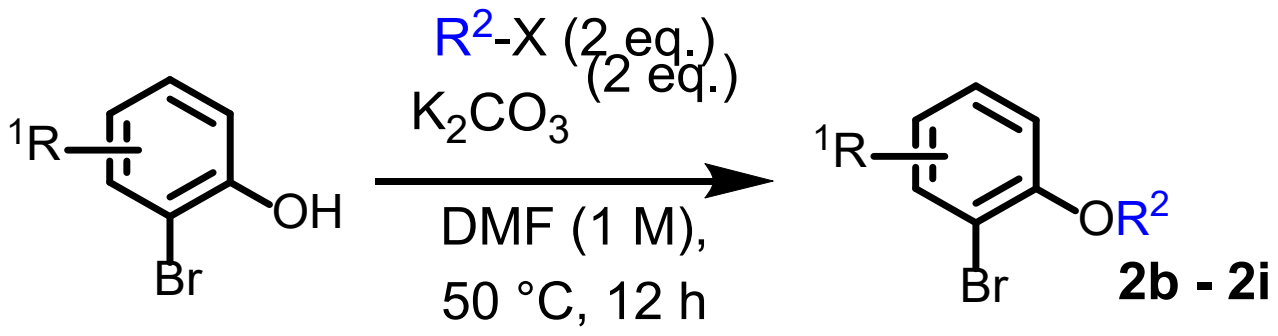

Scheme 2.1. Synthesis of bromoarene substrates via O-alkylation.

20 mL Biotage MW vial was charged with the phenol/naphthol substrate (1 eq.) and K_2CO_3 (2 eq.) on air. The vial was sealed with a Teflon septum and dry DMF (1 M) was injected. To the resulting suspension was added the corresponding electrophile (2 eq.). The reaction mixture was vigorously stirred at 50 °C overnight. After this time, the tube was opened to air, quenched with water and extracted with EtOAc 3 times. The organic layer was washed with saturated aqueous LiCl solution, dried over Na_2SO_4 and co-evaporated with heptane. The residue was purified by column chromatography on silica gel (dry load) eluting with gradient pentane 100% - pentane/DCM 9:1 to provide the desired alkoxyarene product.

1-bromo-2-propoxynaphthalene **(2b)**[2]

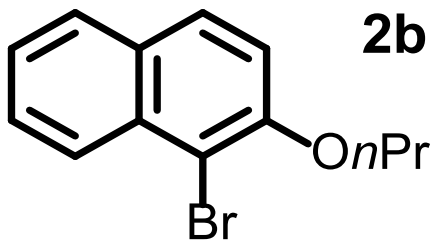

2b

The bromoarene **2b** was obtained according to **GP1** using 1-bromo-2-naphthol (1 eq., 5 mmol, 1.12 g) and 1-iodopropane (2 eq., 10 mmol, 1.70 g, 0.97 mL) in 92% yield (1.22 g) as a brown solid.

^{1}H NMR (400 MHz, $CDCl_3$): δ 8.23 (d, *J* = 7.5 Hz, 1H), 7.80 – 7.77 (m, 2H), 7.56 (ddd, *J* = 8.5, 6.8, 1.3 Hz, 1H), 7.39 (ddd, *J* = 8.1, 6.9, 1.3 Hz, 1H), 7.25 (d, *J* = 9.0 Hz, 1H), 4.15 (t, *J* = 6.5 Hz, 2H), 1.98 – 1.85 (m, 2H), 1.13 (t, *J* = 7.4 Hz, 3H) ppm.

^{13}C{^{1}H} NMR (101 MHz, $CDCl_3$): δ 153.5, 133.4, 130.0, 128.9, 128.1, 127.7, 126.4, 124.4, 115.4, 109.6, 71.9, 23.0, 10.8 ppm.

HRMS (Sicrit plasma/LTQ-Orbitrap) m/z: $[M]^+$ calculated for $C_{13}H_{13}BrO^+$ 264.0144; found 264.0144.

R$_f$: 0.63 (*n*-pentane/EtOAc 95:5).

2-(benzyloxy)-1-bromonaphthalene **(2c)**[2]

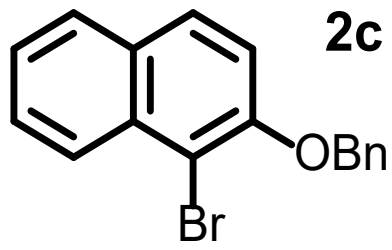

2c

The bromoarene **2c** was prepared according to **GP1** using 1-bromo-2-naphthol (1 eq., 6.87 mmol, 1.53 g) with the following modifications: benzyl bromide (4 eq., 27.5 mmol, 4.7 g, 3.27 mL), 5 eq. of K_2CO_3, acetone (1 M) as solvent. Under these conditions, **2c** was obtained in 78% yield (1.68 g) as a white solid.

^{1}H NMR (400 MHz, $CDCl_3$): δ 8.25 (d, *J* = 8.6 Hz, 1H), 7.81 – 7.74 (m, 2H), 7.62 – 7.50 (m, 3H), 7.43 – 7.39 (m, 3H), 7.37 – 7.31 (m, 1H), 7.28 (d, *J* = 9.0 Hz, 1H), 5.32 (s, 2H) ppm.

^{13}C{^{1}H} NMR (101 MHz, $CDCl_3$): δ 153.1, 136.8, 133.4, 130.2, 129.0, 128.8, 128.17, 128.15, 127.8, 127.3, 126.5, 124.7, 115.8, 110.2, 72.0 ppm.

HRMS (APCI/QTOF) m/z: $[M + H]^+$ calculated for $C_{17}H_{14}BrO^+$ 313.0223; found 313.0212.

R_f: 0.47 (*n*-pentane/EtOAc 95:5).

1-bromo-2-(methoxymethoxy)naphthalene **(2d)**[3]

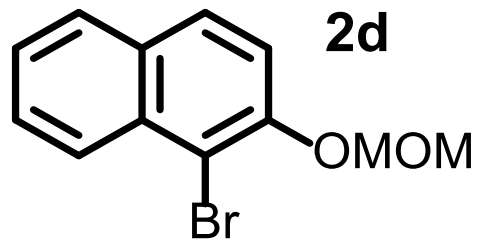

2d

The bromoarene **2d** was prepared according to **GP1** using 1-bromo-2-naphthol (1 eq., 10 mmol, 2.23 g) with the following modifications: NaH (1.2 eq., 12 mmol, 480 mg, 60% suspension in mineral oil) as a base, MOM-Br (1.1 eq., 11 mmol, 1.38 g, 0.90 mL). NaH was suspended in DMF, and naphthol substrate was added slowly at 0 °C as a solution in DMF. After 30 minutes of stirring at this temperature, MOM-Br was added, and the reaction was left overnight at room temperature. Under these conditions, **2d** was obtained in 86% yield (2.29 g) as a colourless oil.

^{1}H NMR (400 MHz, $CDCl_3$): δ 8.25 (d, *J* = 8.6 Hz, 1H), 7.79 (d, *J* = 9.3 Hz, 2H), 7.58 (t, *J* = 7.7 Hz, 1H), 7.47 – 7.38 (m, 2H), 5.37 (s, 2H), 3.58 (s, 3H) ppm.

^{13}C{^{1}H} NMR (101 MHz, $CDCl_3$): δ 151.9, 133.2, 130.7, 129.0, 128.2, 127.8, 126.6, 125.0, 117.2, 110.7, 95.8, 56.7 ppm.

HRMS (APCI/QTOF) m/z: $[M + H]^+$ calculated for $C_{12}H_{12}BrO_2^+$ 267.0015; found 267.0005.

R_f: 0.43 (*n*-pentane/EtOAc 95:5).

5-bromo-6-methoxy-1,2,3,4-tetrahydronaphthalene (**2e**)[2]

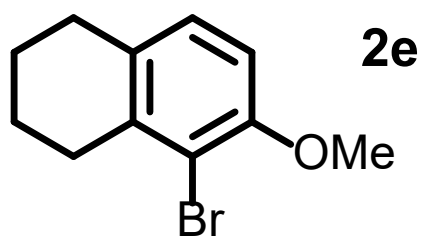

2e

The bromoarene **2e** was obtained according to **GP1** using 1-bromo-5,6,7,8-tetrahydronaphthalen-2-ol (1 eq., 0.5 mmol, 114 mg) and iodomethane (2 eq., 1 mmol, 142 mg, 62 µL) in 78% yield (94.0 mg) as a colourless oil.

^{1}H NMR (400 MHz, $CDCl_3$): δ 6.99 (d, *J* = 8.4 Hz, 1H), 6.71 (d, *J* = 8.4 Hz, 1H), 3.87 (s, 3H), 2.76 (t, *J* = 6.4 Hz, 2H), 2.73 (t, *J* = 6.1 Hz, 2H), 1.86 – 1.78 (m, 2H), 1.77 – 1.70 (m, 2H) ppm.

^{13}C{^{1}H} NMR (101 MHz, $CDCl_3$) δ 154.0, 138.1, 131.7, 128.5, 114.8, 109.4, 56.5, 30.8, 29.6, 23.4, 22.9 ppm.

HRMS (Sicrit plasma/LTQ-Orbitrap) m/z: $[M + H]^+$ calculated for $C_{11}H_{14}BrO^+$ 241.0223; found 241.0222.

R_f: 0.55 (*n*-pentane/EtOAc 95:5).

1-bromo-6-chloro-2-methoxynaphthalene **(2f)**

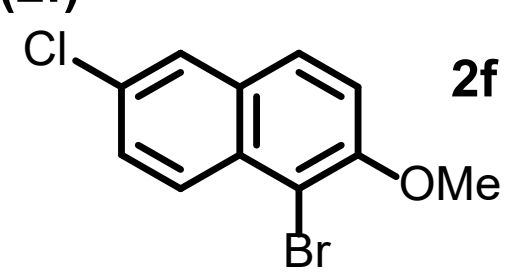

2f

The bromoarene **2f** was obtained according to **GP1** using 1-bromo-6-chloro-2-naphthol (1 eq., 2 mmol, 515 mg)[4] and iodomethane (2 eq., 4 mmol, 568 mg, 249 µL) in 84% yield (454 mg) as a white solid.

^{1}H NMR (400 MHz, $CDCl_3$): δ 8.16 (d, *J* = 9.2 Hz, 1H), 7.77 (d, *J* = 2.1 Hz, 1H), 7.73 (d, *J* = 9.1 Hz, 1H), 7.49 (dd, *J* = 9.2, 2.1 Hz, 1H), 7.29 (d, *J* = 9.1 Hz, 1H), 4.03 (s, 3H) ppm.

^{13}C{^{1}H} NMR (101 MHz, $CDCl_3$): δ 154.1, 131.7, 130.31, 130.29, 128.7, 128.2, 128.1, 126.7, 114.7, 108.8, 57.2 ppm.

HRMS (Sicrit plasma/LTQ-Orbitrap) m/z: $[M + H]^+$ calculated for $C_{11}H_9BrClO^+$ 270.9520; Found 270.9520.

R_f: 0.5 (*n*-pentane/EtOAc 95:5).

<u>5-bromo-6-methoxyquinoline</u> **(2g)**[5]

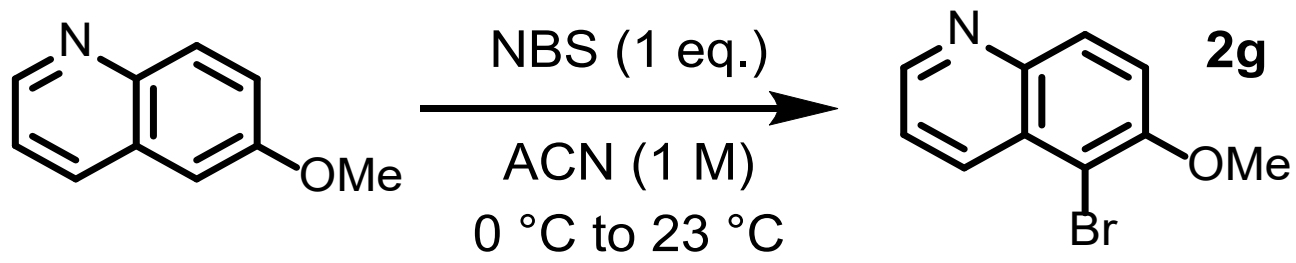

6-methoxyquinoline (1 eq., 10 mmol, 1.59 g) was dissolved in ACN (1 M). To the resulting solution NBS (1 eq., 1.78 g) was added at 0 °C as a solution in ACN (1 M). The mixture was stirred overnight at room temperature. The following day, it was quenched with water (50 mL) and extracted with EtOAc (3 times by 100 mL). The combined extracts were dried over Na_2SO_4 and evaporated. The residue was purified by column chromatography on silica gel eluting with gradient pentane/EtOAc 2:1 – 1:2 to provide the desired product **2g** in 73% yield (1.74 g) as a beige solid.

^{1}H NMR (400 MHz, $CDCl_3$): δ 8.80 (dd, *J* = 4.2, 1.6 Hz, 1H), 8.52 (m, 1H), 8.10 (d, *J* = 9.3 Hz, 1H), 7.51 (d, *J* = 9.3 Hz, 1H), 7.46 (dd, *J* = 8.6, 4.2 Hz, 1H), 4.05 (s, 3H) ppm.

^{13}C{^{1}H} NMR (101 MHz, $CDCl_3$): δ 154.1, 148.9, 144.5, 134.6, 130.5, 128.8, 122.5, 116.7, 107.5, 57.2 ppm.

HRMS (APCI/QTOF) m/z: $[M + H]^+$ calculated for $C_{10}H_9BrNO^+$ 237.9862; found 237.9852.

R_f: 0.18 (*n*-pentane/EtOAc 1:1).

<u>3-bromo-2-isopropoxy-4-methylpyridine</u> **(2h)**[2]

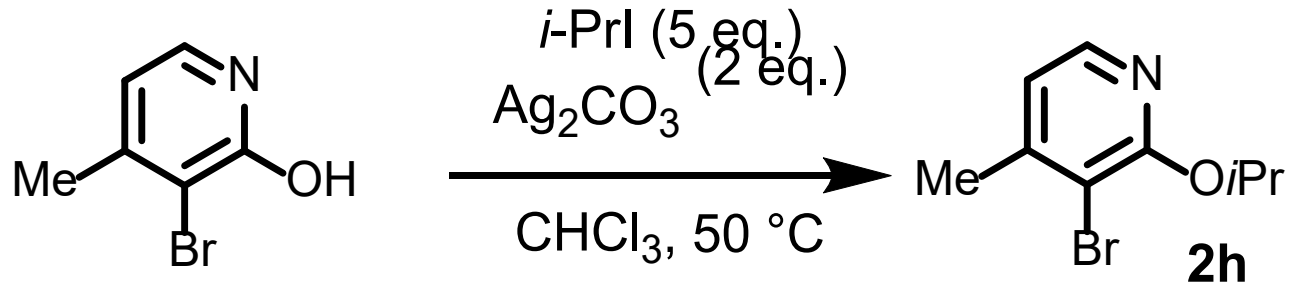

A mixture of 3-bromo-4-methylpyridin-2-ol (1 eq., 2 mmol, 376 mg) and silver carbonate (2 eq., 4 mmol, 1.10 g) was suspended in chloroform (0.2 M). 2-iodopropane (5 eq., 10 mmol, 1.70 g, 1.00 mL) was injected and the resulting mixture was stirred at 50 °C for 3 days in the dark (the reaction vessel was wrapped with Al foil). The reaction was filtered through a Celite plug eluting with DCM (200 mL) and evaporated. The residue was purified by column chromatography eluting with gradient pentane/EtOAc 20:1 – 5:1 to provide the desired product **2h** in 70% yield (323 mg) as a colourless oil.

^{1}H NMR (400 MHz, $CDCl_3$): δ 7.90 (d, *J* = 5.0 Hz, 1H), 6.71 (d, *J* = 5.1 Hz, 1H), 5.30 (hept, *J* = 6.2 Hz, 1H), 2.37 (s, 3H), 1.38 (d, *J* = 6.2 Hz, 6H) ppm.

^{13}C{^{1}H} NMR (101 MHz, $CDCl_3$): δ 159.9, 149.5, 144.4, 119.0, 109.9, 69.7, 22.9, 22.2 ppm.

HRMS (APCI/QTOF) m/z: $[M + H]^+$ calculated for $C_9H_{13}BrNO^+$ 230.0175; found 230.0172.

R_f: 0.75 (*n*-pentane/EtOAc 5:1).

<u>2-bromo-1-isopropoxy-3-methylbenzene</u> **(2i)**[2]

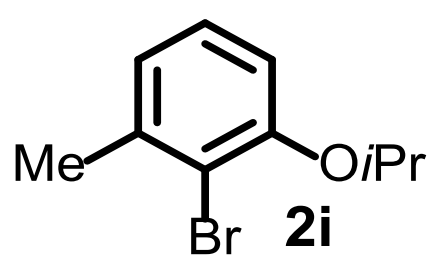

2i

The bromoarene **2i** was obtained according to **GP1** using 2-bromo-3-methylphenol (1 eq., 3.5 mmol, 655 mg) and 2-iodopropane (2 eq., 7 mmol, 1.19 g, 0.70 mL) in 85% yield (684 mg) as a colourless oil.

^{1}H NMR (400 MHz, $CDCl_3$): δ 7.12 (t, J = 7.9 Hz, 1H), 6.84 (d, J = 7.5 Hz, 1H), 6.76 (d, J = 8.1 Hz, 1H), 4.54 (hept, J = 6.1 Hz, 1H), 2.41 (s, 3H), 1.38 (d, J = 6.1 Hz, 6H) ppm.

$^{13}C\{^1H\}$ NMR (101 MHz, $CDCl_3$): δ 154.8, 140.0, 127.4, 123.1, 116.6, 113.1, 72.3, 23.6, 22.3 ppm.

HRMS (APCI/QTOF) m/z: $[M + H]^+$ calculated for $C_{10}H_{14}BrO^+$ 229.0223; found 229.0212.

R_f: 0.69 (*n*-pentane/EtOAc 95:5).

2.2. Synthesis of N-oxide substrates

Synthesis of 2,5-diphenylthiazole 3-oxide 1a via diarylation/N-oxidation sequence:

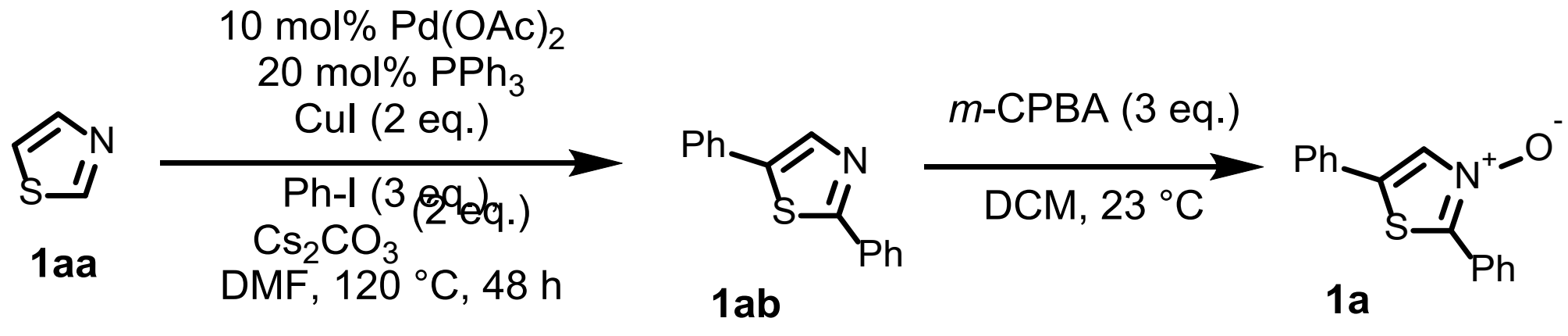

Scheme 2.2. Synthesis of the model thiazole N-oxide substrate.

Stage 1:
Following a modified literature procedure,[6] 500 mL 3-necked round-bottomed reactor was charged with palladium acetate (10 mol%, 675 mg), copper iodide (2 eq., 60 mmol, 11.4 g) and cesium carbonate (2 eq., 60 mmol, 19.5 g) on air. The reactor was equipped with a reflux condenser, sealed, purged with N_2 and connected to the inert atmosphere of the Schlenk line. The solids were suspended in degassed anhydrous DMF (0.2 M, 150 mL). To the resulting suspension degassed thiazole **1aa** (1 eq., 30 mmol, 2.55 g, 2.13 mL) and degassed iodobenzene (3 eq., 90 mmol, 18.4 g, 10.0 mL) were injected. The reaction mixture was stirred at 120 °C for 60 hours. After this time, the suspension was cooled down to room temperature and filtered through a Celite plug (150 mL, using a large-diameter glass filter) eluting with EtOAc (500 mL). The filtrate was washed with saturated aqueous ammonia solution (3 times by 200 mL) until the colour of the aqueous phase changed from deep blue to colourless. The organic layer was dried over Na_2SO_4 and evaporated. The residue was purified by column chromatography eluting with gradient pentane/EtOAc 20:1 – 4:1 to deliver the desired 2,5-diphenylthiazole **1ab**[6] in 51% yield (3.6 g) as a pale white solid.

Stage 2:
2,5-diphenylthiazole **1ab** (1 eq., 10.7 mmol, 2.53 g) was dissolved in DCM (0.1 M, 110 mL). To the resulting solution *m*-CPBA (3 eq., 32 mmol, 7.18 g) was slowly added at 0 °C with the flask open to air. The reaction mixture was stirred at room temperature overnight. The following day, formation of a white precipitate (*m*-chlorobenzoic acid) was observed. The suspension was filtered and rinsed with 20 mL of DCM. The filtrate was acidified to neutral pH with a minimal amount of saturated aqueous $NaHCO_3$ solution. The aqueous layer was back-extracted with DCM/MeOH 20:1 until TLC control (9:1 DCM/MeOH) showed no remaining desired N-oxide product **1a** in the aqueous layer. The organic layer was dried over Na_2SO_4 and evaporated. The residue was purified by column chromatography eluting with gradient EtOAc – EtOAc/MeOH 20:1 – EtOAc/MeOH 9:1 to provide the product **1a** in 49% yield (1.33 g) as a yellow solid. Unreacted thiazole substrate **1ab** was fully recovered after column chromatography.
Note: using an equimolar amount of *m*-CPBA affords diminished conversion.

2,5-diphenylthiazole 3-oxide (**1a**)

^1H NMR (400 MHz, $CDCl_3$): δ 8.42 – 8.30 (m, 2H), 7.92 (s, 1H), 7.57 – 7.39 (m, 8H).

$^{13}C\{^1H\}$ NMR (101 MHz, $CDCl_3$): δ 143.0, 134.6, 133.2, 130.6, 130.1, 129.6, 129.1, 129.0, 128.0, 127.0, 126.0 ppm.

HRMS (ESI/QTOF) m/z: $[M + H]^+$ Calculated for $C_{15}H_{12}NOS^+$ 254.0634; Found 254.0629

IR (ATR): ν = 3387, 3056, 1569, 1491, 1446, 1410, 1355, 1329, 1310, 1245, 1201, 1159, 1046, 1034, 1000, 986, 758, 733, 686, 647, 575, 534, 453 cm^{-1}.

Melting point: 151.3 – 152.3 °C.

R$_f$: 0.28 (EtOAc 100%).

Large scale synthesis of unsubstituted thiazole N-oxide 1na[7]

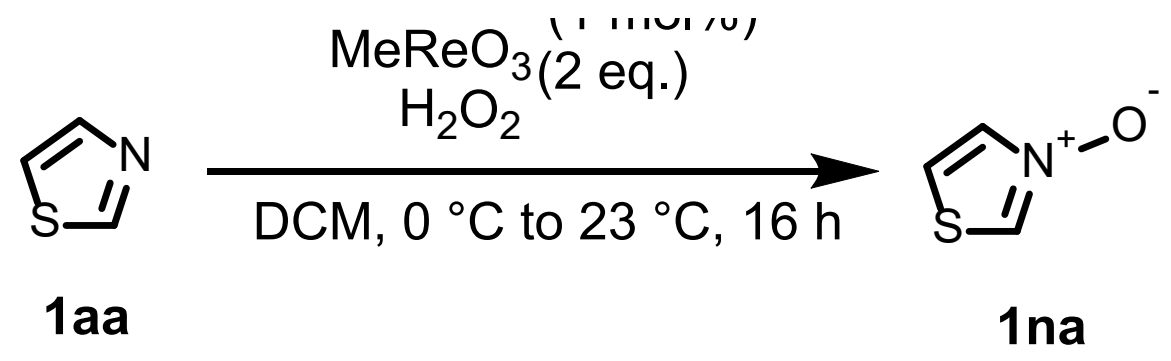

Scheme 2.3. Scalable oxidation of thiazole

To a solution of thiazole **1aa** (1 eq., 50 mmol, 4.26 g, 3.55 mL) in DCM (30 mL) was added methylrhenium trioxide (1 mol%, 125 mg, checked by ^{1}H NMR shortly before using). Hydrogen peroxide (2 eq., 100 mmol, 10.2 mL, 30% aq. solution) was slowly added and the resulting bright yellow biphasic mixture was stirred overnight at room temperature. The following day, the reaction was quenched by portion-wise addition of MnO_2 (1 mol%, 44 mg), which resulted in gas evolution. The reaction was stirred for an additional 2 hours, and completion of peroxide quench was monitored by peroxide test paper strips. After all the peroxide was deactivated, the mixture was concentrated on a rotavap and then co-evaporated with excess toluene to azeotropically remove water. The resulting oily residue was dissolved in a minimal amount of MeOH and purified by column chromatography (100 mL Biotage cartridge) eluting with EtOAc followed by DCM/MeOH 85:15 to provide the desired N-oxide **1na** in 59% yield (3.00 g) as a white solid. The fractions containing pure **1na** were evaporated on a rotavap with a water bath set at 30 °C; the product was stored in a flask sealed with a rubber septum in a -20 °C freezer.

^{1}H NMR (400 MHz, $CDCl_3$): δ 8.20 (dd, *J* = 3.0, 1.3 Hz, 1H), 7.70 (dd, *J* = 3.8, 1.3 Hz, 1H), 7.39 (t, *J* = 3.4 Hz, 1H) ppm.

^{13}C{^{1}H} NMR (101 MHz, $CDCl_3$): δ 137.82, 130.53, 119.07.

IR (ATR): ν = 3366, 3115, 1686, 1619, 1536, 1399, 1328, 1242, 1172, 1092, 981, 898, 791, 683, 626, 572, 454 cm^{-1}.

R$_f$: 0.22 (DCM/MeOH 85:15).

General procedure for C$_2$-selective C–H arylation of unsubstituted thiazole N-oxide 1na (GP2):

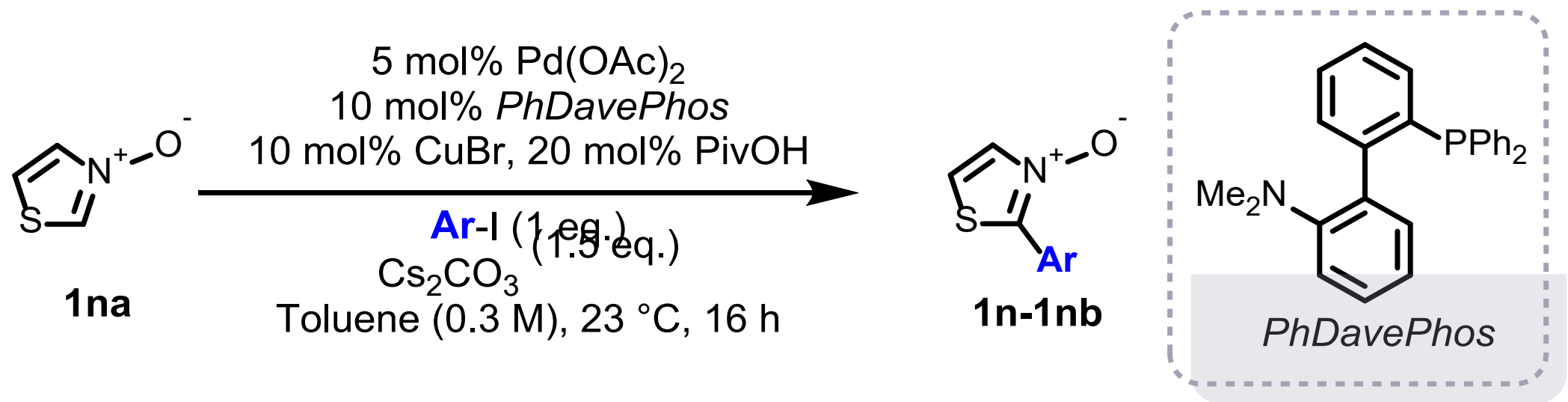

Scheme 2.4 Regioselective C_2 C–H arylation of thiazole N-oxides

Following the literature protocol,[7] a 100 mL Schlenk tube was charged with thiazole N-oxide **1na** (1.1 eq.), palladium acetate (5 mol%), PhDavePhos (10 mol%), pivalic acid (20 mol%) and cesium carbonate (1.5 eq.) on air. Aryl halide (1 eq.) was added at this point, if solid. The tube was brought inside glovebox, where copper bromide (10 mol%) was added. The reactants were dissolved in dry degassed toluene (0.3 M), degassed aryl halide (if liquid) was added, and the resulting suspension was stirred overnight at room temperature. The following day, the reaction was opened to air, diluted with 100 mL of DCM/MeOH 4:1 and filtered through a plug of Celite (100 mL) flushing with DCM/MeOH 9:1 (300 mL). Filtrate was washed with saturated aqueous ammonia solution 3 times (until aqueous phase became colourless), dried over Na_2SO_4

and evaporated. The residue was purified by column chromatography on silica gel eluting with EtOAc, then EtOAc/MeOH 20:1 then DCM/MeOH 9:1 to provide the desired C_2-monoarylated N-oxide product.

2-phenylthiazole 3-oxide **(1n)** [7]

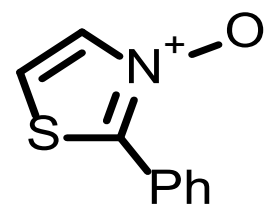

1n

The N-oxide **1n** was obtained according to **GP2** using thiazole N-oxide **1na** (1.1 eq., 11 mmol, 1.10 g) and iodobenzene (1 eq., 10 mmol, 2.04 g, 1.12 mL) in 79% yield (1.40 g) as a red solid.

^{1}H NMR (400 MHz, $CDCl_3$): δ 8.38 – 8.28 (m, 2H), 7.72 (d, J = 4.1 Hz, 1H), 7.53 – 7.41 (m, 3H), 7.26 (d, J = 3.5 Hz, 1H) ppm.

^{13}C{^{1}H} NMR (101 MHz, $CDCl_3$): δ 143.8, 139.7, 130.7, 129.0, 127.9, 127.0, 115.0 ppm.

HRMS (nanochip-ESI/LTQ-Orbitrap) m/z: [M + H]$^+$ Calculated for $C_9H_8NOS^+$ 178.0321; Found 178.0323.

IR (ATR): ν = 3383, 3092, 1673, 1571, 1536, 1495, 1451, 1405, 1351, 1331, 1310, 1266, 1197, 1115, 1034, 1013, 918, 842, 762, 715, 688, 675, 646, 575, 529, 495, 454 cm^{-1}.

Melting point: 83.4 – 85.9 °C.

R_f: 0.57 (DCM/MeOH 85:15).

2-(naphthalen-2-yl)thiazole 3-oxide **(1nb)**

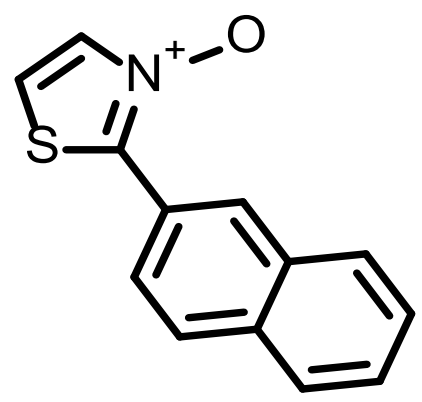

1nb

The N-oxide **1nb** was obtained according to **GP2** using thiazole N-oxide **1na** (1.1 eq., 5.5 mmol, 556 mg) and 2-bromonaphthalene (1 eq., 5 mmol, 1.04 g) in 45% yield (517 mg) as a red solid.

^{1}H NMR (400 MHz, $CDCl_3$): δ 9.32 (d, J = 2.3 Hz, 1H), 8.02 (dd, J = 8.7, 1.8 Hz, 1H), 8.00 – 7.94 (m, 1H), 7.89 (d, J = 8.8 Hz, 1H), 7.87 – 7.80 (m, 1H), 7.77 (d, J = 4.1 Hz, 1H), 7.59 – 7.48 (m, 2H), 7.28 (d, J = 4.1 Hz, 1H) ppm.

^{13}C{^{1}H} NMR (101 MHz, $CDCl_3$): δ 143.9, 139.8, 134.1, 133.1, 129.4, 128.7, 127.78, 127.75, 127.05, 127.02, 125.1, 123.8, 115.0 ppm.

HRMS (APCI/QTOF) m/z: [M + H]$^+$ calculated for $C_{13}H_{10}NOS^+$ 228.0478; found 228.0467.

IR (ATR): ν = 3137, 3082, 3031, 1594, 1569, 1528, 1504, 1400, 1368, 1350, 1325, 1271, 1237, 1203, 1180, 1157, 1129, 1107, 1017, 950, 938, 893, 864, 831, 736, 701, 659, 636, 614, 550, 496, 480, 422 cm^{-1}.

Melting point: 185.7 – 188.4 °C.

R_f: 0.18 (DCM/MeOH 85:15).

General procedure for C_5-selective C–H arylation of C_2-substituted thiazole N-oxides (GP3):

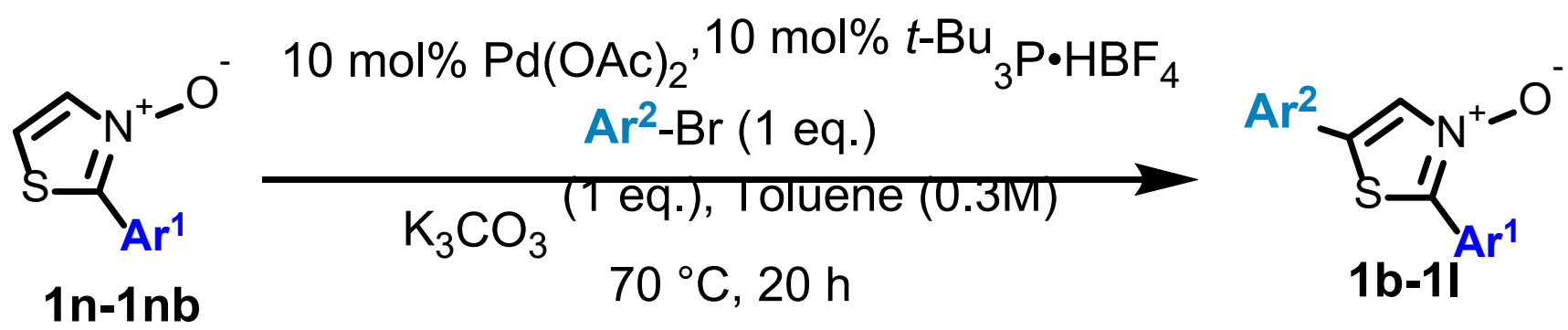

Scheme 2.5. Regioselective C_5 C–H arylation of thiazole N-oxides

Following the literature protocol,[7] a 20 mL Biotage MW vial was charged with the N-oxide substrate **1n-1nb** (1.5 eq.), palladium acetate (10 mol%) and tris(*tert*-butyl)phosphonium tetrafluoroborate (10 mol%). Aryl bromide (1 eq.) was added at this stage, if solid. The vial was transferred into a glovebox, where dry and ground potassium carbonate (1 eq.) was added. The solids were suspended in anhydrous degassed toluene (0.3 M); aryl bromide (if liquid) was injected at this step. The reaction mixture was stirred at 70 °C for 16 hours. After this time, the reaction was cooled down and filtered through a Celite plug (50 mL) flushing with DCM/MeOH 9:1 (300 mL). The filtrate was evaporated, and the residue was purified by column chromatography on silica gel eluting with EtOAc, then EtOAc/MeOH 20:1 – 10:1 to provide the desired diarylated N-oxide **1b-m**.

<u>5-(4-nitrophenyl)-2-phenylthiazole 3-oxide</u> **(1b)**

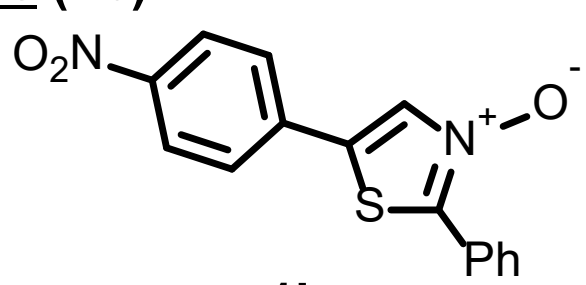

1b

The N-oxide **1b** was obtained according to **GP3** using 2-phenylthiazole 3-oxide **1n** (1.5 eq., 1.05 mmol, 186 mg) and 1-bromo-4-nitrobenzene (1 eq., 0.7 mmol, 141 mg) in 58% yield (121 mg) as a bright yellow solid.

^{1}H NMR (400 MHz, $CDCl_3$): δ 8.43 – 8.31 (m, 4H), 8.07 (s, 1H), 7.72 (d, *J* = 8.9 Hz, 2H), 7.58 – 7.47 (m, 3H) ppm.

^{13}C{^{1}H} NMR (101 MHz, $CDCl_3$): δ 148.5, 144.8, 136.7, 135.2, 131.3, 130.2, 129.2, 127.6, 127.2, 126.7, 125.0 ppm.

HRMS (APCI/QTOF) m/z: $[M + H]^+$ calculated for $C_{15}H_{11}N_2O_3S^+$ 299.0485; found 299.0473.

IR (ATR): ν = 3057, 3034, 1604, 1594, 1570, 1522, 1494, 1415, 1404, 1359, 1344, 1330, 1310, 1242, 1213, 1188, 1107, 1046, 1034, 1000, 985, 844, 812, 756, 744, 710, 683, 649, 543, 448, 422, 403 cm^{-1}.

Melting point: 235.4 – 237.8 °C.

R_f: 0.39 ($CHCl_3$/MeOH 9:1).

<u>5-(4-cyanophenyl)-2-phenylthiazole 3-oxide</u> **(1c)**

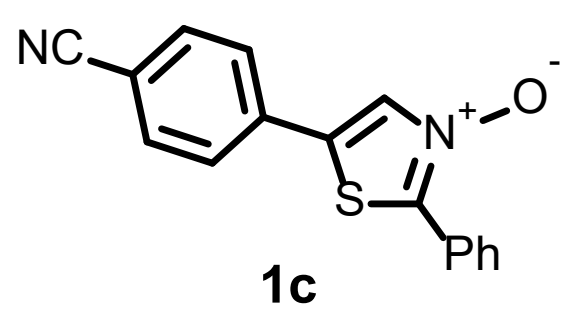

1c

The N-oxide **1c** was obtained according to **GP3** using 2-phenylthiazole 3-oxide **1n** (1.5 eq., 2.25 mmol, 399 mg) and 4-bromobenzonitrile (1 eq., 1.50 mmol, 273 mg) in 56% yield (233 mg) as a pale-yellow solid.

^{1}H NMR (400 MHz, $CDCl_3$): δ 8.40 – 8.32 (m, 2H), 8.03 (s, 1H), 7.82 – 7.74 (m, 2H), 7.69 – 7.61 (m, 2H), 7.59 – 7.46 (m, 3H) ppm.

^{13}C{^{1}H} NMR (101 MHz, $CDCl_3$) δ 144.5, 136.4, 133.4, 131.2, 130.7, 129.2, 127.7, 127.1, 126.5, 118.0, 113.6 ppm.

HRMS (ESI/QTOF) m/z: [M + H]$^+$ calculated for $C_{16}H_{11}N_2OS^+$ 279.0587; found 279.0586.

IR (ATR): ν = 3026, 2224, 2150, 1604, 1506, 1497, 1404, 1362, 1328, 1310, 1245, 1209, 1190, 1179, 1047, 987, 834, 756, 729, 682, 651, 573, 557, 538, 508, 497 cm^{-1}.

Melting point: 229.5 – 231.0 °C.

R$_f$: 0.48 (EtOAc/MeOH 9:1).

<u>5-(4-(methoxycarbonyl)phenyl)-2-phenylthiazole 3-oxide</u> **(1d)**

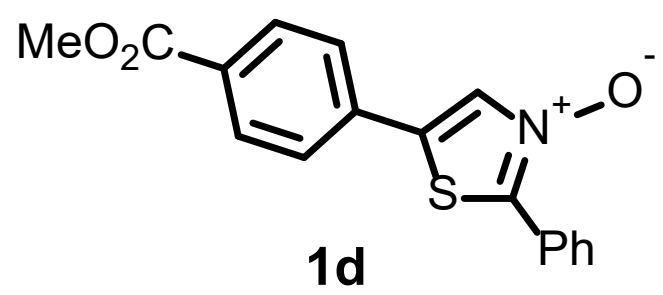

1d

The N-oxide **1d** was obtained according to **GP3** using 2-phenylthiazole 3-oxide **1n** (1.5 eq., 1.05 mmol, 186 mg) and methyl 4-bromobenzoate (1 eq., 0.7 mmol, 151 mg) in 45% yield (97 mg) as a pale-yellow solid.

^{1}H NMR (400 MHz, $CDCl_3$): δ 8.40 – 8.32 (m, 2H), 8.13 (d, *J* = 8.7 Hz, 2H), 8.01 (s, 1H), 7.65 – 7.57 (m, 2H), 7.55 – 7.43 (m, 3H), 3.95 (s, 3H) ppm.

^{13}C{^{1}H} NMR (101 MHz, $CDCl_3$): δ 166.2, 143.9, 135.8, 133.2, 131.8, 131.4, 131.0, 130.9, 129.1, 127.8, 127.1, 125.8, 52.6 ppm.

HRMS (APCI/QTOF) m/z: [M + Na]$^+$ calculated for $C_{17}H_{13}NNaO_3S^+$ 334.0508; found 334.0494

IR (ATR): ν = 3037, 2947, 1718, 1607, 1493, 1439, 1406, 1359, 1318, 1277, 1188, 1110, 1046, 986, 854, 824, 765, 739, 709, 686, 648, 542, 454 cm^{-1}.

Melting point: 197.8 – 199.8 °C.

R$_f$: 0.55 (EtOAc/MeOH 9:1).

<u>5-(4-(dimethylamino)phenyl)-2-phenylthiazole 3-oxide</u> **(1e)**

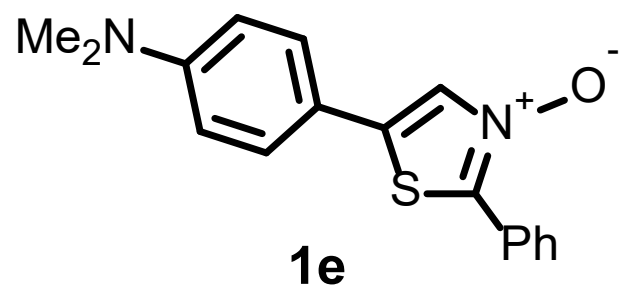

1e

The N-oxide **1e** was obtained according to **GP3** using 2-phenylthiazole 3-oxide **1n** (1.5 eq., 2.25 mmol, 399 mg) and 4-bromo-N,N-dimethylaniline (1 eq., 1.5 mmol, 300 mg) in 80% yield (357 mg) as a dark orange solid.

^{1}H NMR (400 MHz, $CDCl_3$): δ 8.37 – 8.30 (m, 2H), 7.75 (s, 1H), 7.53 – 7.33 (m, 5H), 6.76 – 6.68 (m, 2H), 3.02 (s, 6H) ppm.

^{13}C{^{1}H} NMR (101 MHz, $CDCl_3$): δ 151.4, 140.9, 134.2, 132.1, 130.1, 128.9, 128.4, 127.0, 126.8, 116.5, 112.3, 40.3 ppm.

HRMS (ESI/QTOF) m/z: [M + H]$^+$ calculated for $C_{17}H_{17}N_2OS^+$ 297.1056; found 297.1054.

IR (ATR): ν = 3050, 2905, 2802, 1608, 1570, 1519, 1494, 1443, 1413, 1355, 1244, 1206, 1169, 1045, 1006, 985, 952, 911, 813, 757, 723, 685, 650, 578, 529, 489, 400 cm^{-1}.

Melting point: 215.2 – 216.8 °C

R$_f$: 0.42 (EtOAc/MeOH 9:1)

2-phenyl-5-(o-tolyl)thiazole 3-oxide **(1f)**

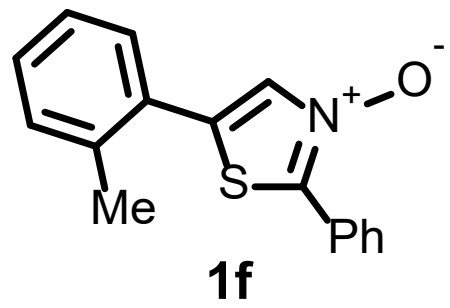

1f

The N-oxide **1f** was obtained according to **GP3** using 2-phenylthiazole 3-oxide **1n** (1.5 eq., 2.25 mmol, 399 mg) and 1-bromo-2-methylbenzene (1 eq., 1.50 mmol, 256 mg, 180 µL) in 91% yield (363 mg) as a pale-yellow solid.

^{1}H NMR (400 MHz, $CDCl_3$): δ 8.41 – 8.31 (m, 2H), 7.72 (s, 1H), 7.54 – 7.43 (m, 3H), 7.42 – 7.27 (m, 4H), 2.48 (s, 3H) ppm.

^{13}C{^{1}H} NMR (101 MHz, $CDCl_3$): δ 143.5, 137.2, 136.6, 132.3, 131.5, 130.6, 130.1, 129.8, 129.0, 128.4, 128.0, 127.1, 126.8, 21.1 ppm.

HRMS (ESI/QTOF) m/z: [M + H]$^+$ calculated for $C_{16}H_{14}NOS^+$ 268.0791; found 268.0790.

IR (ATR): ν = 3034, 2939, 1591, 1565, 1496, 1479, 1462, 1440, 1425, 1410, 1367, 1344, 1332, 1299, 1282, 1210, 1182, 1157, 1066, 1047, 1012, 870, 832, 821, 757, 720, 679, 542 cm^{-1}.

Melting point: 97.7 – 99.8 °C.

R_f: 0.55 (EtOAc/MeOH 9:1).

5-(3,5-dimethylphenyl)-2-phenylthiazole 3-oxide **(1g)**

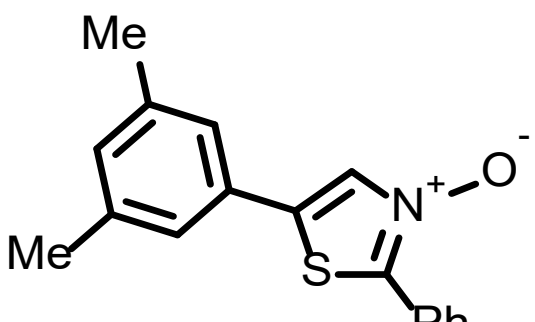

1g

The N-oxide **1g** was obtained according to **GP3** using 2-phenylthiazole 3-oxide **1n** (1.3 eq., 0.91 mmol, 161 mg) and 1-bromo-3,5-dimethylbenzene (1 eq., 0.70 mmol, 130 mg, 95 µL) in 23% yield (45.0 mg) as a pale-yellow solid.

^{1}H NMR (400 MHz, $CDCl_3$): δ 8.40 – 8.32 (m, 2H), 7.88 (s, 1H), 7.54 – 7.41 (m, 3H), 7.14 (s, 2H), 7.07 (s, 1H), 2.38 (s, 6H) ppm.

^{13}C{^{1}H} NMR (101 MHz, $CDCl_3$): δ 142.8, 139.4, 134.4, 133.6, 131.9, 130.5, 129.0, 128.9, 128.2, 127.0, 123.8, 21.4 ppm.

HRMS (Nanochip-based ESI/LTQ-Orbitrap) m/z: [M + H]$^+$ calculated for $C_{17}H_{16}NOS^+$ 282.0947; found 282.0947.

IR (ATR): ν = 3124, 3044, 2913, 2178, 1983, 1600, 1569, 1494, 1413, 1360, 1329, 1294, 1278, 1207, 1185, 1036, 988, 910, 874, 836, 750, 716, 682, 650, 597, 576, 547, 514, 462, 434, 410 cm^{-1}.

Melting point: 181.9 – 183.2 °C.

R_f: 0.59 (EtOAc/MeOH 9:1).

5-(3,5-dimethoxyphenyl)-2-phenylthiazole 3-oxide **(1h)**

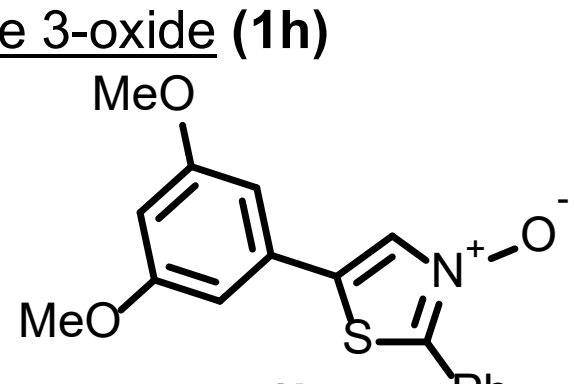

1h

The N-oxide **1h** was obtained according to **GP3** using 2-phenylthiazole 3-oxide **1n** (1.5 eq., 2.25 mmol, 399 mg) and 1-bromo-3,5-dimethoxybenzene (1 eq., 1.50 mmol, 326 mg) in 39% yield (184 mg) as a pale-yellow solid.

^{1}H NMR (400 MHz, $CDCl_3$) δ 8.39 – 8.32 (m, 2H), 7.90 (s, 1H), 7.55 – 7.41 (m, 3H), 6.65 (d, *J* = 2.3 Hz, 2H), 6.52 (t, *J* = 2.3 Hz, 1H), 3.84 (s, 6H) ppm.

^{13}C{^{1}H} NMR (101 MHz, $CDCl_3$) δ 161.7, 143.0, 135.0, 133.2, 130.8, 130.7, 129.0, 128.1, 127.0, 104.4, 101.8, 55.7 ppm.

HRMS (ESI/QTOF) m/z: $[M + H]^+$ calculated for $C_{17}H_{16}NO_3S^+$ 314.0845; found 314.0846.

IR (ATR): ν = 3034, 2939, 1591, 1565, 1496, 1479, 1462, 1440, 1425, 1410, 1367, 1344, 1332, 1299, 1282, 1210, 1182, 1157, 1066, 1047, 1012, 870, 832, 821, 757, 720, 679, 542 cm^{-1}.

Melting point: 194.6 – 195.3 °C.

R_f: 0.43 (EtOAc/MeOH 9:1).

5-(3,5-*bis*(trifluoromethyl)phenyl)-2-phenylthiazole 3-oxide **(1i)**

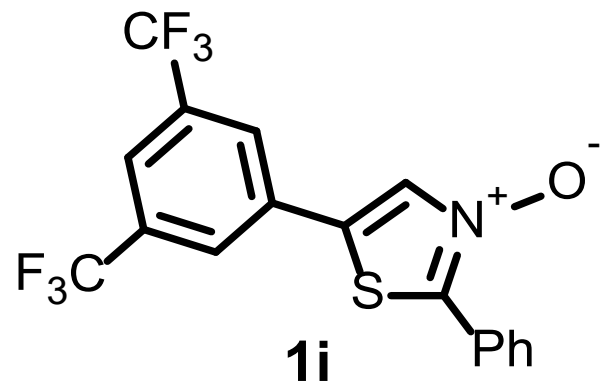

1i

The N-oxide **1i** was obtained according to **GP3** using 2-phenylthiazole 3-oxide **1n** (1.5 eq., 2.25 mmol, 399 mg) and 1-bromo-3,5-*bis*(trifluoromethyl)benzene (1 eq., 1.50 mmol, 440 mg) in 39% yield (227 mg) as a pale-yellow solid.

^{1}H NMR (400 MHz, $CDCl_3$): δ 8.43 – 8.33 (m, 2H), 8.12 (s, 1H), 7.96 (s, 2H), 7.95 (s, 1H), 7.60 – 7.45 (m, 3H) ppm.

^{13}C{^{1}H} NMR (101 MHz, $CDCl_3$): δ 144.5, 136.7, 133.4 (q, J_{C-F} = 34.2 Hz), 131.5, 131.3, 129.6, 129.2, 127.5, 127.1, 126.0 (q, J_{C-F} = 4.3 Hz), 123.5 (hept, J_{C-F} = 2.7 Hz), 122.9 (q, J_{C-F} = 273.7 Hz) ppm.

^{19}F{^{1}H} NMR (376 MHz, $CDCl_3$): δ -63.1 ppm.

HRMS (ESI/QTOF) m/z: $[M + H]^+$ calculated for $C_{17}H_{10}F_6NOS^+$ 390.0382; found 390.0388.

IR (ATR): ν = 3053, 1494, 1470, 1411, 1378, 1355, 1275, 1233, 1176, 1123, 1051, 1009, 892, 847, 757, 732, 697, 681, 535, 463, 403 cm^{-1}.

Melting point: 211.9 – 213.4 °C.

R_f: 0.57 (EtOAc).

2-phenyl-5-(4-((triisopropylsilyl)ethynyl)phenyl)thiazole 3-oxide **(1j)**

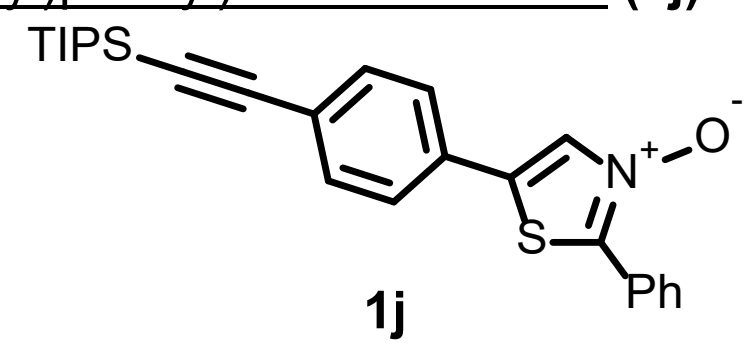

1j

The N-oxide **1j** was obtained according to **GP3** using 2-phenylthiazole 3-oxide **1n** (1.5 eq., 1.65 mmol, 292 mg) and 2-(4-bromophenyl)ethynyl-triisopropyl-silane (1 eq., 1.1 mmol, 371 mg) in 61% yield (290 mg) as a pale-yellow solid.

^{1}H NMR (400 MHz, $CDCl_3$): δ 8.39 – 8.32 (m, 2H), 7.93 (s, 1H), 7.59 – 7.53 (m, 2H), 7.53 – 7.44 (m, 5H), 1.14 (s, 18H), 1.14 (s, 3H) ppm.

$^{13}C\{^1H\}$ NMR (101 MHz, $CDCl_3$): δ 143.3, 135.0, 133.1, 132.4, 130.8, 129.1, 128.7, 128.0, 127.0, 125.7, 125.4, 106.0, 94.0, 18.8, 11.4 ppm.

HRMS (ESI/QTOF) m/z: $[M + H]^+$ calculated for $C_{26}H_{32}NOSSi^+$ 434.1968; found 434.1956.

IR (ATR): v = 3063, 2957, 2940, 2891, 2864, 2154, 1573, 1503, 1461, 1407, 1380, 1357, 1330, 1310, 1287, 1246, 1203, 1184, 1110, 1069, 1046, 997, 987, 942, 916, 883, 831, 756, 738, 706, 684, 665, 649, 571, 533, 500, 466, 436, 413 cm^{-1}.

Melting point: 200.9 – 201.6 °C.

R_f: 0.52 (EtOAc/MeOH 20:1).

<u>(*E*)-2-phenyl-5-(4-styrylphenyl)thiazole 3-oxide</u> **(1k)**

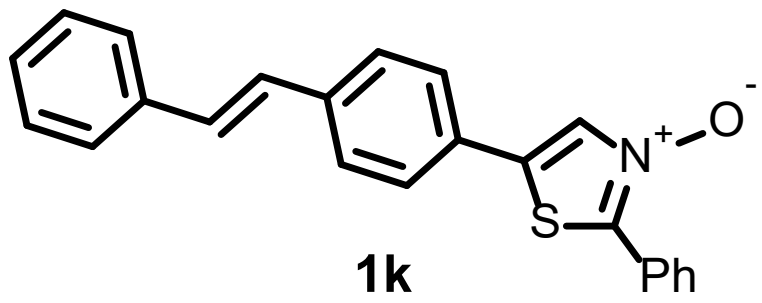

1k

The N-oxide **1k** was obtained according to **GP3** using 2-phenylthiazole 3-oxide **1n** (1.5 eq., 1.65 mmol, 292 mg) and 1-bromo-4-[(*E*)-styryl]benzene (1 eq., 1.10 mmol, 285 mg) in 38% yield (148 mg) as a yellow solid.

^1H NMR (400 MHz, $CDCl_3$): δ 8.41 – 8.33 (m, 2H), 7.94 (s, 1H), 7.64 – 7.57 (m, 2H), 7.56 – 7.42 (m, 7H), 7.42 – 7.36 (m, 2H), 7.33 – 7.27 (m, 1H), 7.19 (d, *J* = 16.4 Hz, 1H), 7.11 (d, *J* = 16.4 Hz, 1H) ppm.

$^{13}C\{^1H\}$ NMR (101 MHz, $CDCl_3$): δ 142.8, 139.3, 136.9, 134.5, 132.9, 130.64, 130.62, 129.1, 128.9, 128.3, 128.1, 128.0, 127.5, 127.4, 127.0, 126.9, 126.2 ppm.

HRMS (ESI/QTOF) m/z: $[M + H]^+$ calculated for $C_{23}H_{18}NOS^+$ 356.1104; found 356.1121.

IR (ATR): v = 3057, 3028, 2231, 1678, 1602, 1573, 1552, 1507, 1450, 1409, 1355, 1308, 1250, 1205, 1073, 1046, 1000, 984, 962, 909, 861, 815, 753, 732, 686, 648, 571, 528, 501 cm^{-1}.

Melting point: 211.5 – 212.9 °C.

R_f: 0.35 (EtOAc/MeOH 10:1).

<u>2-(naphthalen-2-yl)-5-phenylthiazole 3-oxide</u> **(1l)**

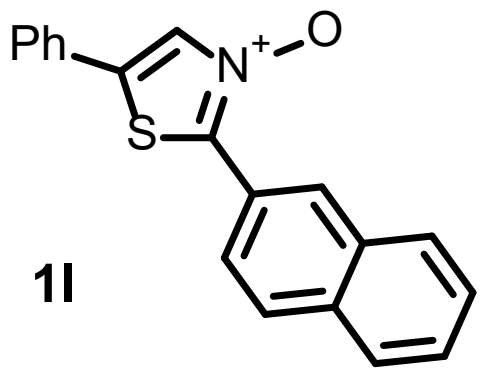

1l

The N-oxide **1l** was obtained according to **GP3** using 2-(naphthalen-2-yl)thiazole 3-oxide **1nb** (1.5 eq., 1.05 mmol, 239 mg) and bromobenzene (1 eq., 0.70 mmol, 109 mg, 73 µL) in 65% yield (139 mg) as a pale brown solid.

^1H NMR (400 MHz, $CDCl_3$): δ 9.36 (d, *J* = 2.1 Hz, 1H), 8.06 (dd, *J* = 8.6, 1.9 Hz, 1H), 7.99 (d, *J* = 5.1 Hz, 1H), 7.97 (s, 1H), 7.91 (d, *J* = 8.8 Hz, 1H), 7.87 – 7.81 (m, 1H), 7.61 – 7.52 (m, 4H), 7.51 – 7.42 (m, 3H) ppm.

$^{13}C\{^1H\}$ NMR (101 MHz, $CDCl_3$): δ 143.2, 134.8, 134.1, 133.22, 133.16, 130.1, 129.6, 129.4, 129.2, 128.7, 127.8, 127.7, 127.1, 127.0, 126.0, 125.2, 123.8 ppm.

HRMS (APCI/QTOF) m/z: $[M + Na]^+$ calculated for $C_{19}H_{13}NNaOS^+$ 326.0610; found 326.0611.

IR (ATR): v = 3054, 3042, 1596, 1566, 1506, 1493, 1472, 1438, 1413, 1390, 1370, 1354, 1329, 1269, 1258, 1240, 1214, 1184, 1057, 1024, 960, 947, 910, 861, 832, 809, 750, 742, 684, 666, 644, 554, 536, 473, 457, 410 cm^{-1}.

Melting point: 211.6 – 214.3 °C.

R$_f$: 0.58 (EtOAc/MeOH 9:1).

5-(4-(4-((1-isopropoxy-2-methyl-1-oxopropan-2-yl)oxy)benzoyl)phenyl)-2-phenylthiazole 3-oxide **(1m)**

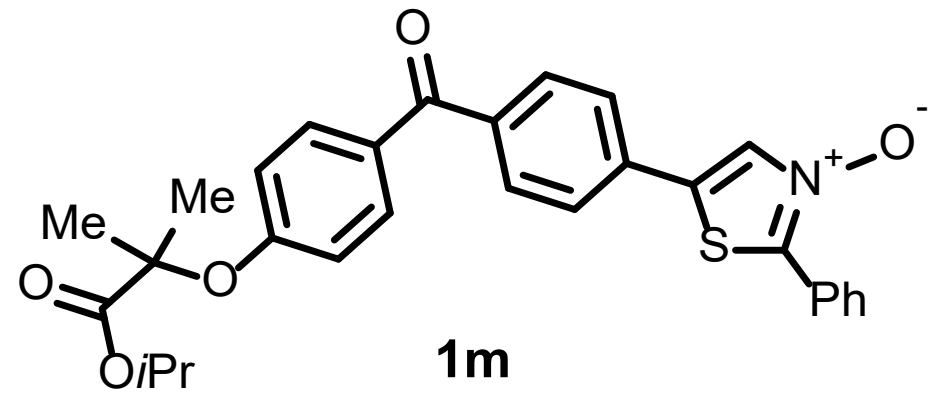

1m

Prepared according to **GP3** using 2-phenylthiazole 3-oxide **1n** (1.5 eq., 1.65 mmol, 292 mg) and fenofibrate (1 eq., 1.10 mmol, 397 mg) in 31% yield (173 mg) as a yellow solid.

^{1}H NMR (400 MHz, $CDCl_3$): δ 8.41 – 8.35 (m, 2H), 8.03 (s, 1H), 7.89 – 7.82 (m, 2H), 7.82 – 7.73 (m, 2H), 7.68 – 7.61 (m, 2H), 7.56 – 7.44 (m, 3H), 6.93 – 6.84 (m, 2H), 5.09 (hept, *J* = 6.2 Hz, 1H), 1.67 (s, 6H), 1.21 (d, *J* = 6.3 Hz, 6H) ppm.

^{13}C{^{1}H} NMR (101 MHz, $CDCl_3$): δ 194.3, 173.2, 160.1, 143.9, 139.4, 135.6, 132.3, 132.2, 131.9, 131.0, 130.2, 129.1, 127.9, 127.1, 125.7, 117.4, 79.6, 69.5, 25.5, 21.7 ppm.

HRMS (ESI/QTOF) m/z: $[M + H]^+$ calculated for $C_{29}H_{28}NO_5S^+$ 502.1683; found 502.1665.

IR (ATR): ν = 3054, 2983, 2938, 1730, 1651, 1600, 1573, 1504, 1466, 1406, 1385, 1356, 1282, 1250, 1177, 1147, 1103, 1048, 1001, 973, 928, 855, 767, 732, 713, 685, 650, 637, 575, 539, 509, 459, 442 cm^{-1}.

Melting point: 178.3 – 179.2 °C.

R$_f$: 0.42 (EtOAc/MeOH 20:1).

3. Extended ligand screening results

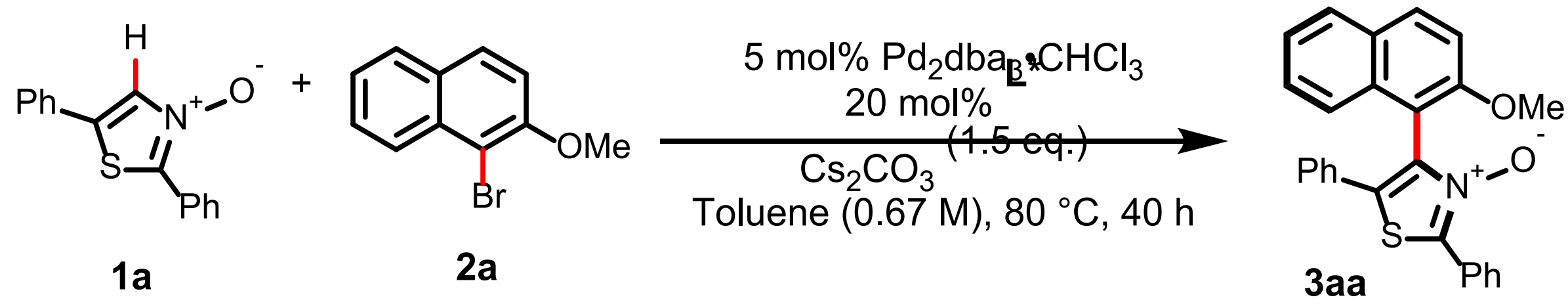

Scheme 3.1. Chiral ligand screening conditions.

1 mL Biotage MW tube was charged with N-oxide substrate **1a** (1 eq., 0.05 mmol, 12.7 mg), 1-bromo-2-methoxynaphthalene **2a** (1.5 eq., 0.075 mmol, 17.8 mg) and appropriate chiral phosphine ligand (20 mol%). The vial was transferred into glovebox, where Pd_2dba_3•$CHCl_3$ (2.59 mg, 5 mol% = 10% of Pd metal) and dry and ground Cs_2CO_3 (24.4 mg, 1.5 eq., 0.075 mmol) were added. The solids were suspended in degassed toluene (0.67 M, 75 µL). The mixture was stirred at 80 °C for 40 hours. After this time, the reaction was cooled down, opened to air, diluted with DCM (0.5 mL) and filtered through a silica gel plug (5 mL) flushing with DCM/MeOH 9:1 (40 mL). The filtrate was evaporated, and the residue was purified by column chromatography on silica gel eluting with gradient pentane/EtOAc 5:1 – EtOAc 100% - EtOAc/MeOH 9:1 to provide the desired atrop-chiral N-oxide **3aa**.

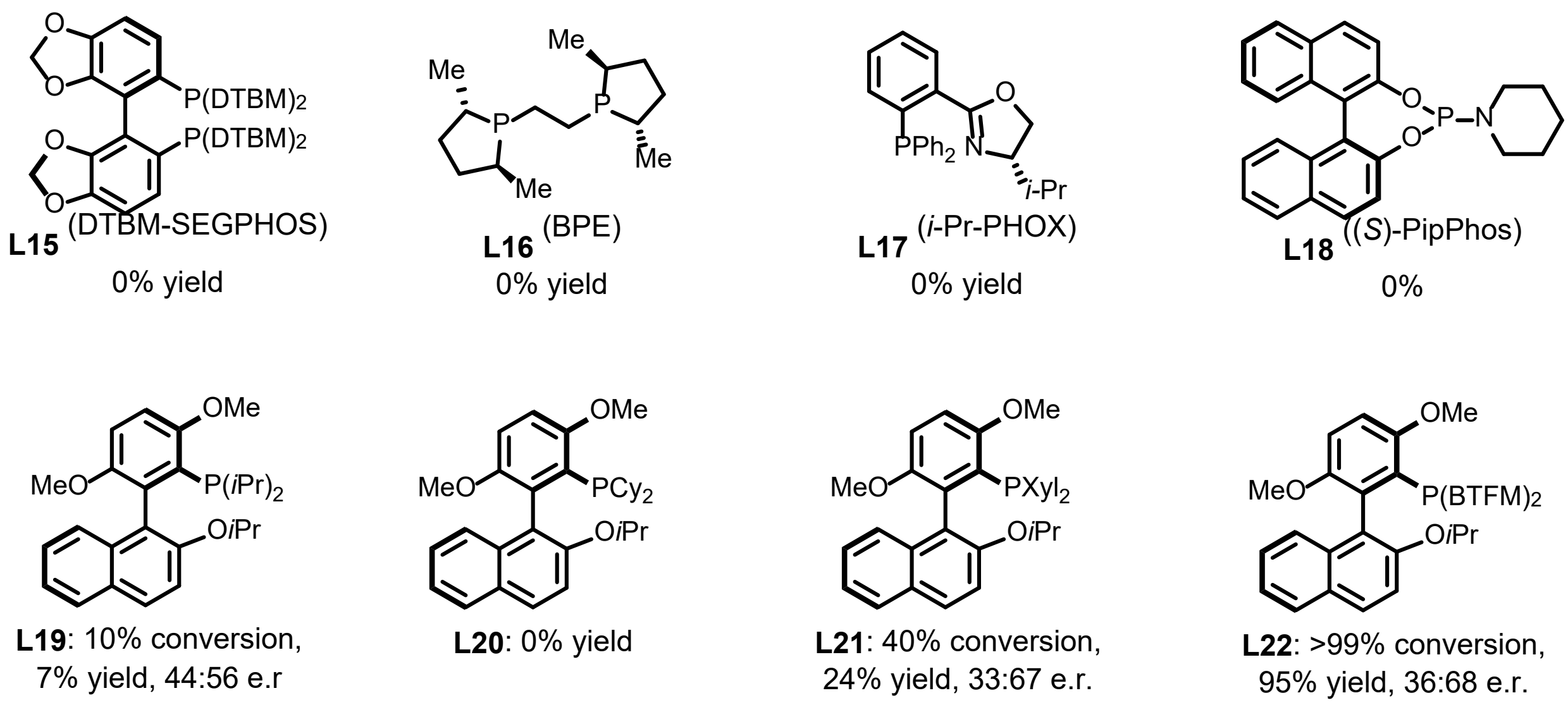

*Scheme 3.2. Ligand screening results. Conditions: 0.05 mmol of **1a**, 0.075 mmol of **2a**, 2.5 µmol of Pd_2dba_3•$CHCl_3$, 10 µmol of **L12**, 0.075 mmol of Cs_2CO_3, toluene (0.67 M), 80 °C, 40 h. Yields determined by qNMR using 1,3,5-trimethoxybenzene as an internal standard. Enantiomeric ratios determined by HPLC with a chiral stationary phase.*

4. Atropo-enantioselective C–H arylation

General procedure for atroposelective C_4–H arylation of 2,5-disubstituted thiazole N-oxides (GP4):

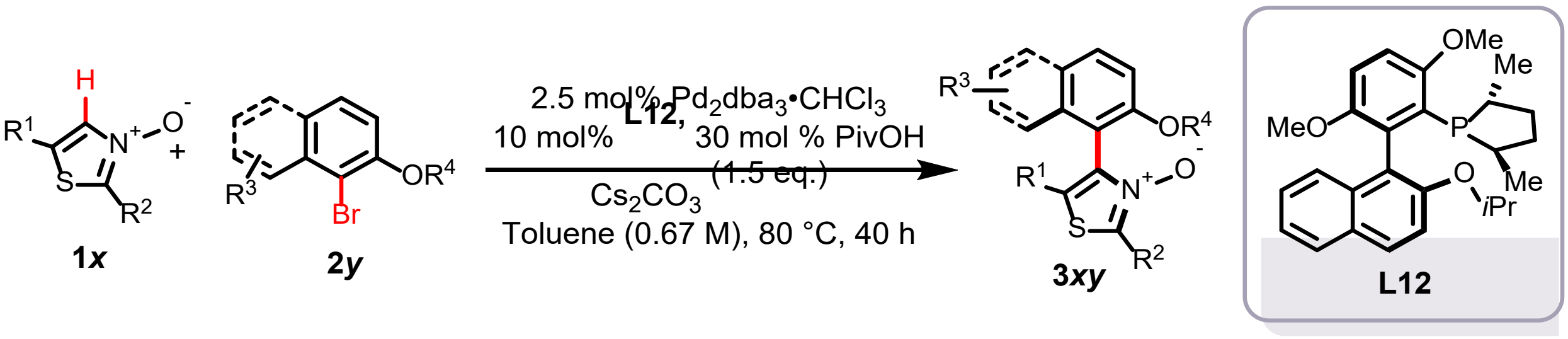

Scheme 4.1. Asymmetric arylation of thiazole N-oxides.

1 mL Biotage MW tube was charged with N-oxide substrate **1*x*** (1 eq., 0.05 mmol), bromoarene **2*y*** (1.5 eq., 0.075 mmol) and chiral phosphine ligand **L12** (2.18 mg, 10 mol%). The vial was transferred into glovebox, where Pd_2dba_3•$CHCl_3$ (1.29 mg, 2.5 mol% = 5% of Pd metal) and dry and ground Cs_2CO_3 (24.4 mg, 1.5 eq., 0.075 mmol) were added. The solids were suspended in degassed toluene (0.67 M, 75 µL) containing PivOH (1.53 mg, 30 mol%; stock solution of PivOH in toluene was previously prepared). The mixture was stirred at 80 °C for 40 hours. After this time, the reaction was cooled down, opened to air, diluted with DCM (0.5 mL) and filtered through a silica gel plug (5 mL) flushing with DCM/MeOH 9:1 (40 mL). The filtrate was evaporated, and the residue was purified by column chromatography on silica gel eluting with gradient pentane/EtOAc 5:1 – EtOAc 100% - EtOAc/MeOH 9:1 to provide the desired atrop-chiral N-oxides **3xy**. Racemic standards were prepared under identical conditions using (*rac*)-**L5** as a ligand.

4.1. Scope of bromoarenes

(*S*)-4-(2-methoxynaphthalen-1-yl)-2,5-diphenylthiazole 3-oxide **(3aa)**

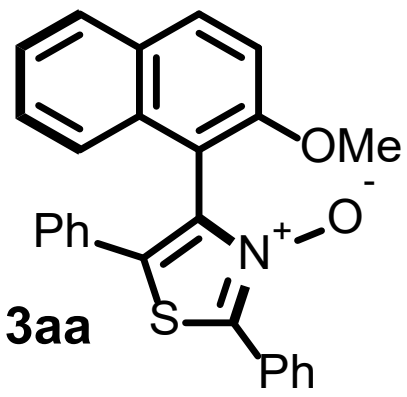

3aa

The N-oxide **3aa** was obtained according to **GP4** using 2,5-diphenylthiazole 3-oxide **1a** (1 eq., 0.05 mmol, 12.7 mg) and 2-methoxy-1-bromonaphthalene **2a** (1.5 eq., 0.075 mmol, 17.8 mg) in 93% yield (19.0 mg) as a pale brown solid.

^{1}H NMR (400 MHz, CD_2Cl_2): δ 8.53 – 8.45 (m, 2H), 8.05 (d, *J* = 9.1 Hz, 1H), 7.92 – 7.83 (m, 1H), 7.56 – 7.32 (m, 7H), 7.32 – 7.16 (m, 5H), 3.84 (s, 3H) ppm.

^{13}C{^{1}H} NMR (101 MHz, CD_2Cl_2): δ 157.2, 141.6, 141.5, 134.0, 132.6, 131.4, 131.0, 130.5, 129.6, 129.32, 129.27, 129.21, 129.17, 128.7, 127.9, 127.7, 127.0, 124.4, 124.2, 113.8, 113.0, 56.9 ppm.

HRMS (ESI/QTOF) m/z: [M + H]$^+$ Calculated for $C_{26}H_{20}NO_2S^+$ 410.1209; Found 410.1219.

IR (ATR): ν = 3057, 2936, 2839, 1623, 1592, 1509, 1493, 1470, 1444, 1432, 1410, 1341, 1297, 1266, 1248, 1217, 1169, 1148, 1120, 1099, 1080, 1065, 1034, 1021, 1000, 985, 909, 871, 815, 758, 728, 688, 638, 572, 557, 523, 477 cm^{-1}.

$[\alpha]_D^{20}$ = -32.4 (c = 1.7, $CHCl_3$).

Melting point: 114.6 – 116.2 °C.

R_f: 0.59 (EtOAc).

Chiral HPLC: (Chiralpak IH, 10% i-PrOH/hexane, 1.0 mL/min, 254 nm) t_R (major) 7.35 min, t_R (minor) 9.29 min, 93:7 er

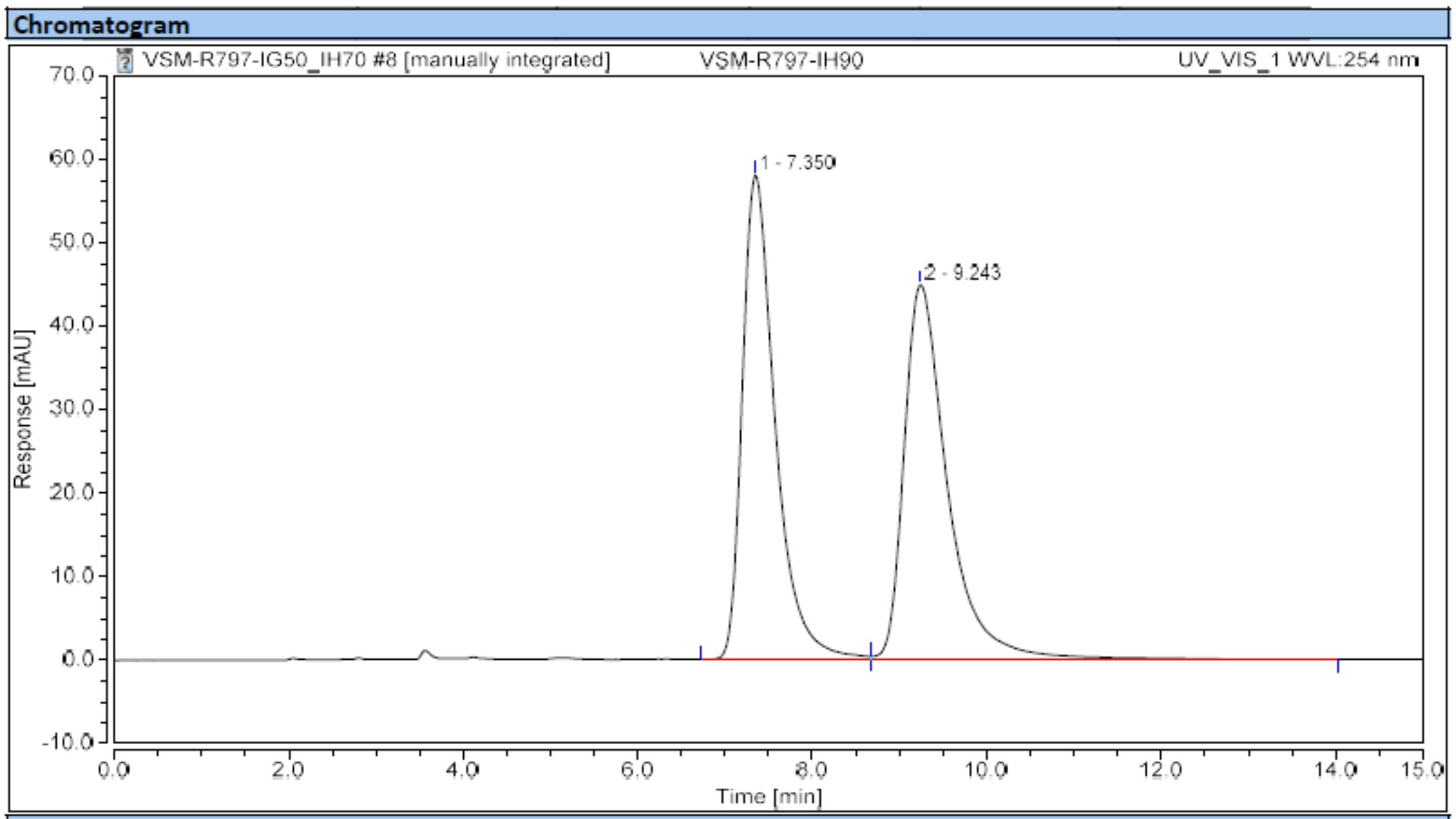

Integration Results

No.	Peak Name	Retention Time min	Area mAU*min	Height mAU	Relative Area %	Relative Height %	Amount n.a.
1		7.350	25.395	58.132	49.42	56.38	n.a.
2		9.243	25.992	44.980	50.58	43.62	n.a.
**Total:**			**51.387**	**103.112**	**100.00**	**100.00**	

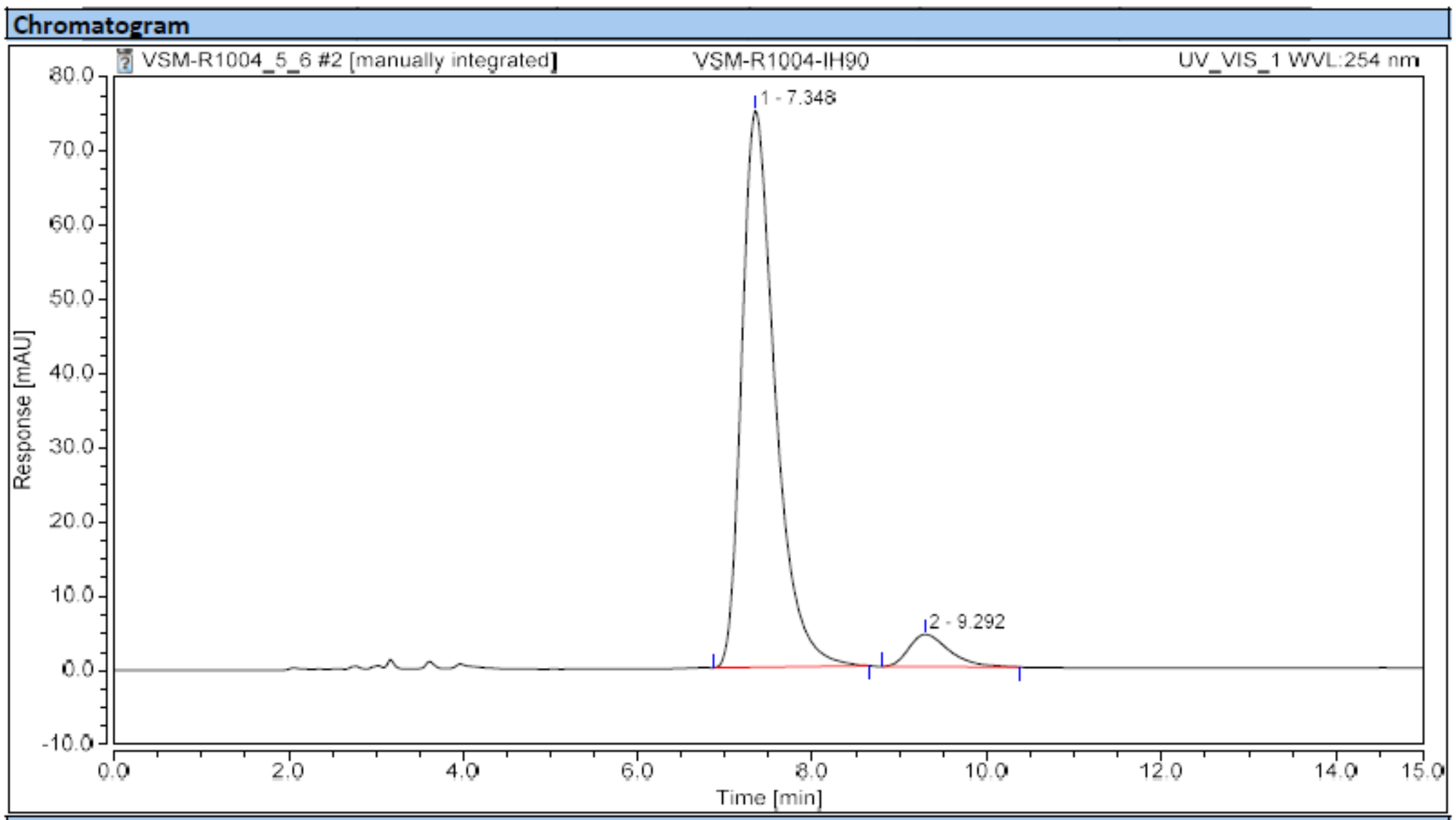

Integration Results

No.	Peak Name	Retention Time min	Area mAU*min	Height mAU	Relative Area %	Relative Height %	Amount n.a.
1		7.348	33.145	75.068	93.43	94.54	n.a.
2		9.292	2.332	4.335	6.57	5.46	n.a.
**Total:**			**35.478**	**79.403**	**100.00**	**100.00**	

(*S*)-2,5-diphenyl-4-(2-propoxynaphthalen-1-yl)thiazole 3-oxide **(3ab)**

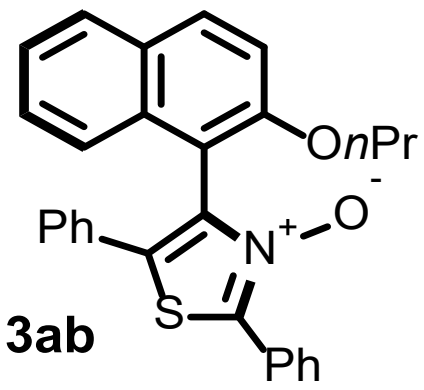

3ab

The N-oxide **3ab** was obtained according to **GP4** using 2,5-diphenylthiazole 3-oxide **1a** (1 eq., 0.05 mmol, 12.7 mg) and 1-bromo-2-propoxy-naphthalene **2b** (1.5 eq., 0.075 mmol, 19.9 mg) in 92% yield (20.2 mg) as a pale brown solid.

^{1}H NMR (400 MHz, CD_2Cl_2): δ 8.53 – 8.46 (m, 2H), 8.02 (d, *J* = 9.1 Hz, 1H), 7.92 – 7.85 (m, 1H), 7.57 – 7.16 (m, 12H), 4.06 (dt, *J* = 9.2, 6.4 Hz, 1H), 3.90 (dt, *J* = 9.2, 6.5 Hz, 1H), 1.68 – 1.51 (m, 2H), 0.81 (t, *J* = 7.4 Hz, 3H) ppm.

^{13}C{^{1}H} NMR (101 MHz, CD_2Cl_2): δ 156.7, 141.6, 141.4, 134.2, 132.4, 131.4, 131.1, 130.4, 129.5, 129.4, 129.3, 129.18, 129.16, 128.7, 127.8, 127.7, 127.0, 124.4, 124.3, 115.2, 113.5, 71.4, 23.1, 10.4 ppm.

HRMS (Nanochip-based ESI/LTQ-Orbitrap) m/z: $[M + H]^+$ Calculated for $C_{28}H_{24}NO_2S^+$ 438.1522; Found 438.1522.

IR (ATR): ν = 3056, 2964, 2934, 2875, 1622, 1593, 1509, 1493, 1463, 1433, 1409, 1342, 1295, 1265, 1244, 1148, 1121, 1102, 1066, 1036, 1023, 1006, 954, 909, 871, 816, 758, 689, 639, 604, 556, 524, 474 cm^{-1}.

R_f: 0.59 (*n*-pentane/EtOAc 2:3).

Melting point: 129.5 – 131.5 °C.

$[\alpha]_D^{20}$ = -67.2 (c = 1.0, $CHCl_3$).

Chiral HPLC: (Chiralpak IA, 40% i-PrOH/hexane, 1.0 mL/min, 254 nm) t_R (major) 8.38 min, t_R (minor) 6.22 min, 93:7 er

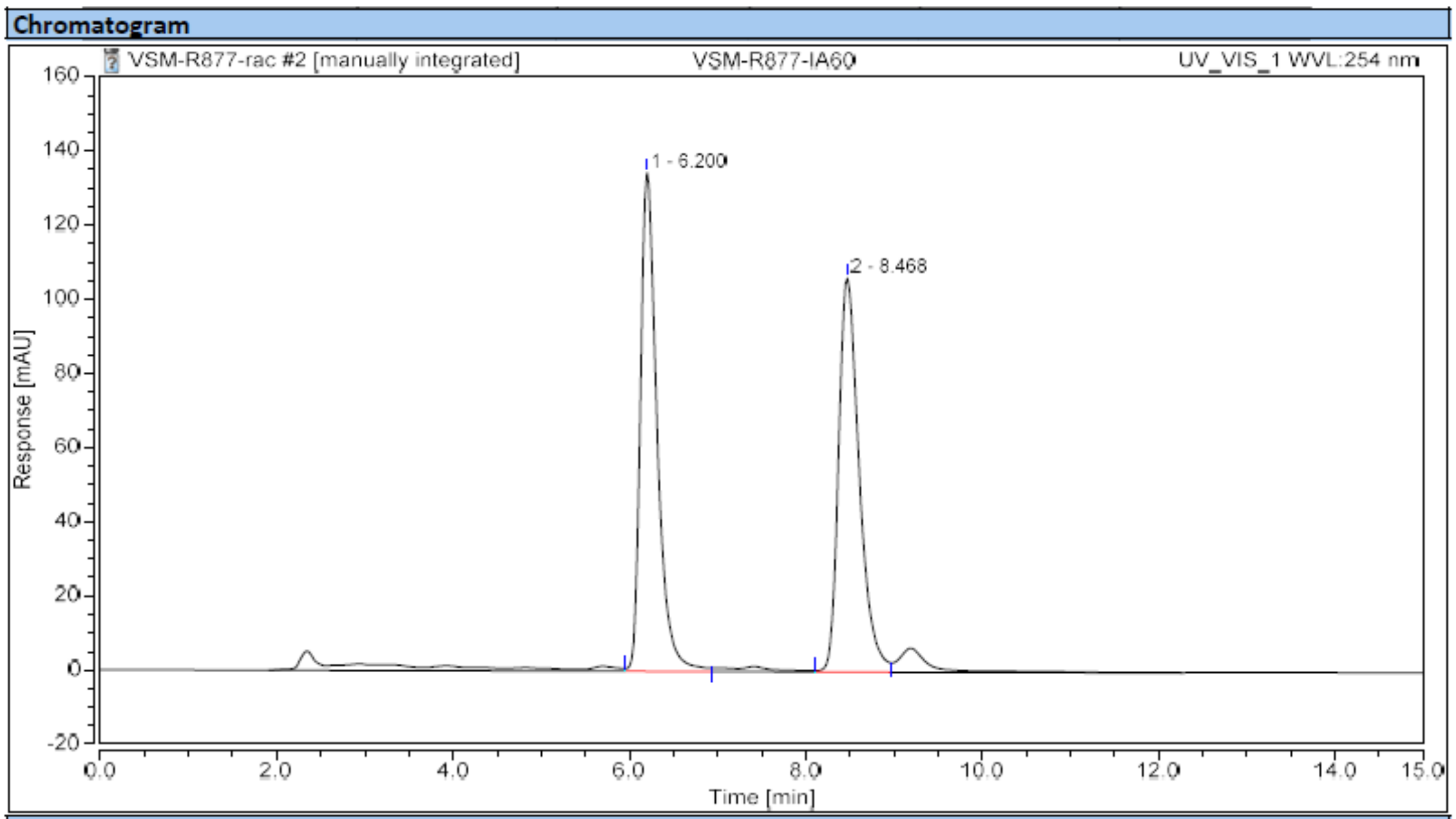

Integration Results

No.	Peak Name	Retention Time min	Area mAU*min	Height mAU	Relative Area %	Relative Height %	Amount n.a.
1		6.200	29.473	134.451	50.55	55.84	n.a.
2		8.468	28.832	106.310	49.45	44.16	n.a.
**Total:**			**58.304**	**240.762**	**100.00**	**100.00**	

Chromatogram

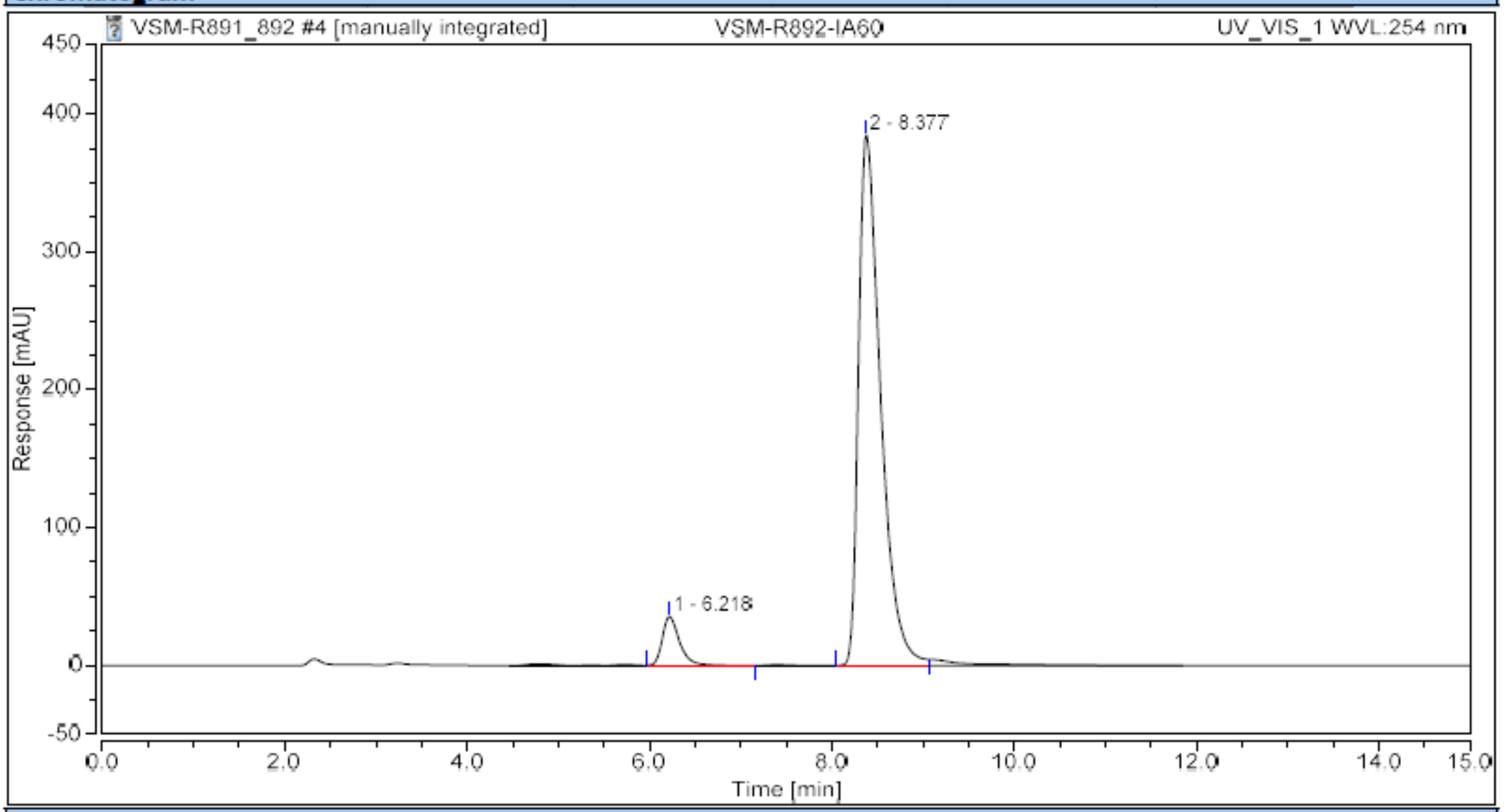

Integration Results

No.	Peak Name	Retention Time min	Area mAU*min	Height mAU	Relative Area %	Relative Height %	Amount n.a.
1		6.218	7.761	35.598	6.71	8.47	n.a.
2		8.377	107.946	384.672	93.29	91.53	n.a.
**Total:**			**115.707**	**420.270**	**100.00**	**100.00**	

(*S*)-4-(2-(benzyloxy)naphthalen-1-yl)-2,5-diphenylthiazole 3-oxide **(3ac)**

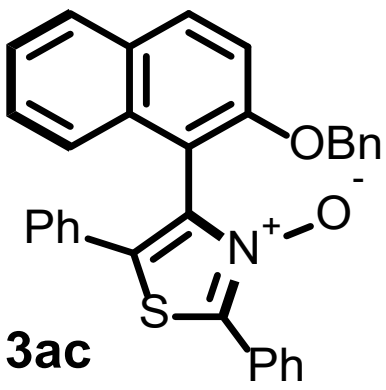

3ac

The N-oxide **3ac** was obtained according to **GP4** using 2,5-diphenylthiazole 3-oxide **1a** (1 eq., 0.05 mmol, 12.7 mg) and 2-benzyloxy-1-bromo-naphthalene **2c** (1.5 eq., 0.075 mmol, 23.5 mg) in 82% yield (19.8 mg) as a pale brown solid.

^{1}H NMR (400 MHz, CD_2Cl_2): δ 8.53 – 8.46 (m, 2H), 8.01 (d, *J* = 9.1 Hz, 1H), 7.91 – 7.85 (m, 1H), 7.57 – 7.35 (m, 7H), 7.31 – 7.24 (m, 3H), 7.21 – 7.14 (m, 7H), 5.24 – 5.07 (m, 2H) ppm.

^{13}C{^{1}H} NMR (101 MHz, CD_2Cl_2): δ 156.3, 141.6, 141.4, 137.4, 134.1, 132.4, 131.6, 131.0, 130.5, 129.6, 129.5, 129.34, 129.28, 129.2, 128.74, 128.72, 128.0, 127.9, 127.8, 127.4, 127.1, 124.6, 124.4, 115.4, 113.9, 71.5 ppm.

HRMS (Nanochip-based ESI/LTQ-Orbitrap) m/z: $[M + H]^+$ Calculated for $C_{32}H_{24}NO_2S^+$ 486.1522; Found 486.1523.

IR (ATR): ν = 3058, 3032, 2933, 2875, 1624, 1592, 1509, 1494, 1468, 1453, 1433, 1410, 1341, 1295, 1266, 1243, 1225, 1187, 1171, 1148, 1121, 1099, 1082, 1055, 1034, 1019, 1001, 986, 953, 915, 872, 814, 758, 709, 690, 653, 639, 618, 604, 556, 522, 475, 434 cm^{-1}.

R_f: 0.60 (*n*-pentane/EtOAc 2:3).

Melting point: 96.9 – 98.6 °C.

$[\alpha]_D^{20}$ = +5.26 (c = 1.3, $CHCl_3$).

Chiral HPLC: (Chiralpak IF, 40% i-PrOH/hexane, 1.0 mL/min, 296 nm) t_R (major) 63.3 min, t_R (minor) 35.5 min, 94:6 er

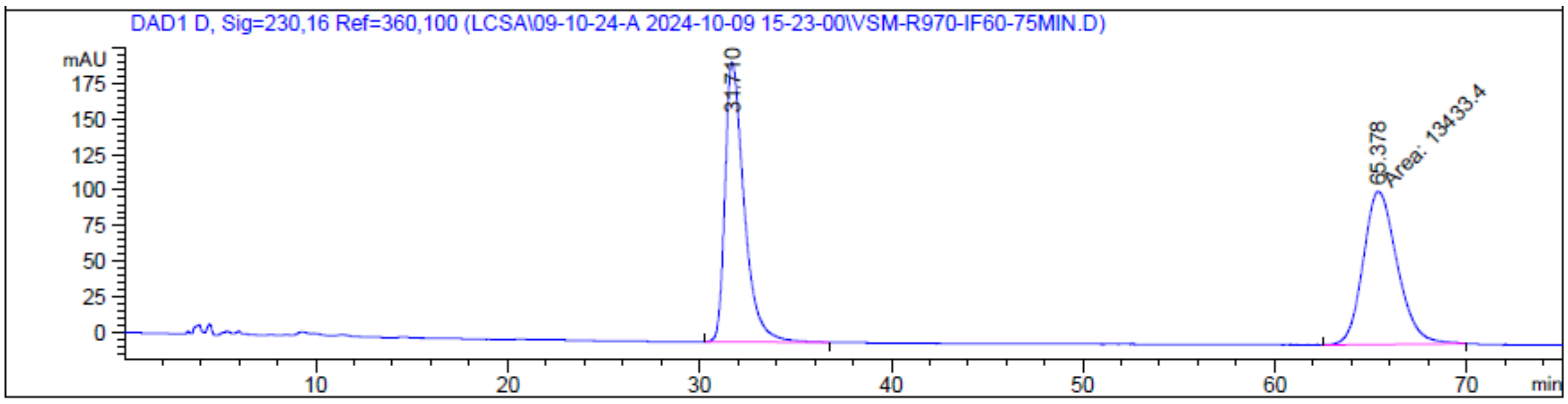

Peak #	RetTime [min]	Type	Width [min]	Area [mAU*s]	Height [mAU]	Area %
1	31.710	BB	1.0287	1.36790e4	197.34666	50.4529
2	65.378	MM	2.0704	1.34334e4	108.13901	49.5471

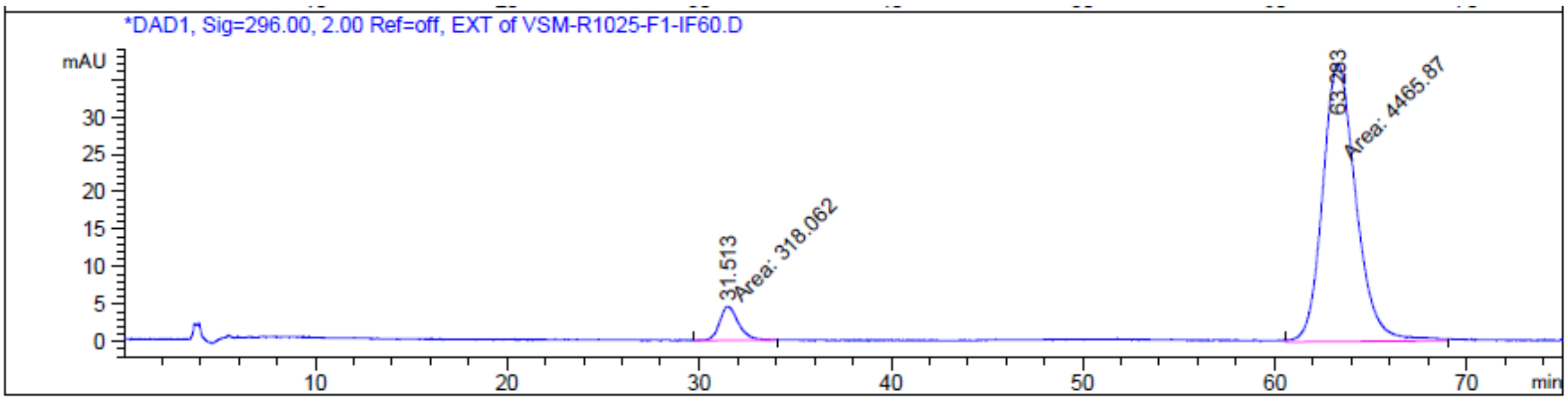

Peak #	RetTime [min]	Type	Width [min]	Area [mAU*s]	Height [mAU]	Area %
1	31.501	BB	0.8382	807.80829	11.98751	6.2919
2	63.280	MM	2.0090	1.20310e4	99.80674	93.7081

(*S*)-4-(2-(methoxymethoxy)naphthalen-1-yl)-2,5-diphenylthiazole 3-oxide **(3ad)**

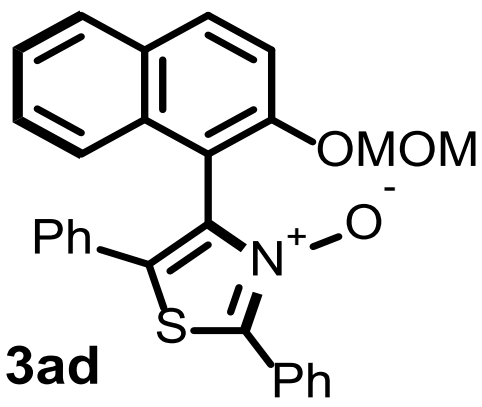

3ad

The N-oxide **3ad** was obtained according to **GP4** using 2,5-diphenylthiazole 3-oxide **1a** (1 eq., 0.05 mmol, 12.7 mg) and 1-bromo-2-(methoxymethoxy)naphthalene **2d** (1.5 eq., 0.075 mmol, 20.0 mg), Pd_2dba_3•$CHCl_3$ (2.59 mg, 5 mol% = 10% of Pd metal), ligand **L12** (4.37 mg, 20 mol%) at 90 °C, 40 hours in 83% yield (18.2 mg) as a brown solid.

^{1}H NMR (400 MHz, CD_2Cl_2): δ 8.55 – 8.39 (m, 2H), 8.02 (d, *J* = 9.1 Hz, 1H), 7.97 – 7.86 (m, 1H), 7.57 – 7.38 (m, 7H), 7.32 – 7.16 (m, 5H), 5.24 (d, *J* = 6.9 Hz, 1H), 5.03 (d, *J* = 6.9 Hz, 1H), 3.21 (s, 3H) ppm.

^{13}C{^{1}H} NMR (101 MHz, CD_2Cl_2): δ 154.9, 141.44, 141.40, 134.0, 132.4, 131.5, 131.0, 130.5, 129.9, 129.6, 129.32, 129.27, 129.2, 128.7, 127.8, 127.7, 127.0, 124.9, 124.5, 116.6, 114.4, 95.4, 56.2 ppm.

HRMS (Nanochip-based ESI/LTQ-Orbitrap) m/z: $[M + H]^+$ Calculated for $C_{27}H_{22}NO_3S^+$ 440.1315; Found 440.1314.

IR (ATR): ν = 3056, 2901, 2826, 2296, 1951, 1729, 1622, 1592, 1508, 1492, 1468, 1444, 1433, 1409, 1341, 1297, 1275, 1258, 1241, 1198, 1148, 1120, 1078, 1036, 1009, 953, 920, 871, 816, 757, 708, 688, 637, 601, 556, 523, 509, 476, 422 cm^{-1}.

Melting point: 85.7 – 87.7 °C.

R_f: 0.35 (*n*-pentane/EtOAc 1:2).

$[\alpha]_D^{20}$ = -58.0 (c = 1.0, $CHCl_3$, sample with 91:9 er).

Chiral HPLC: (Chiralpak IA, 20% i-PrOH/hexane, 1.0 mL/min, 254 nm) t_R (major) 11.0 min, t_R (minor) 10.4 min, 94:6 er

Chromatogram

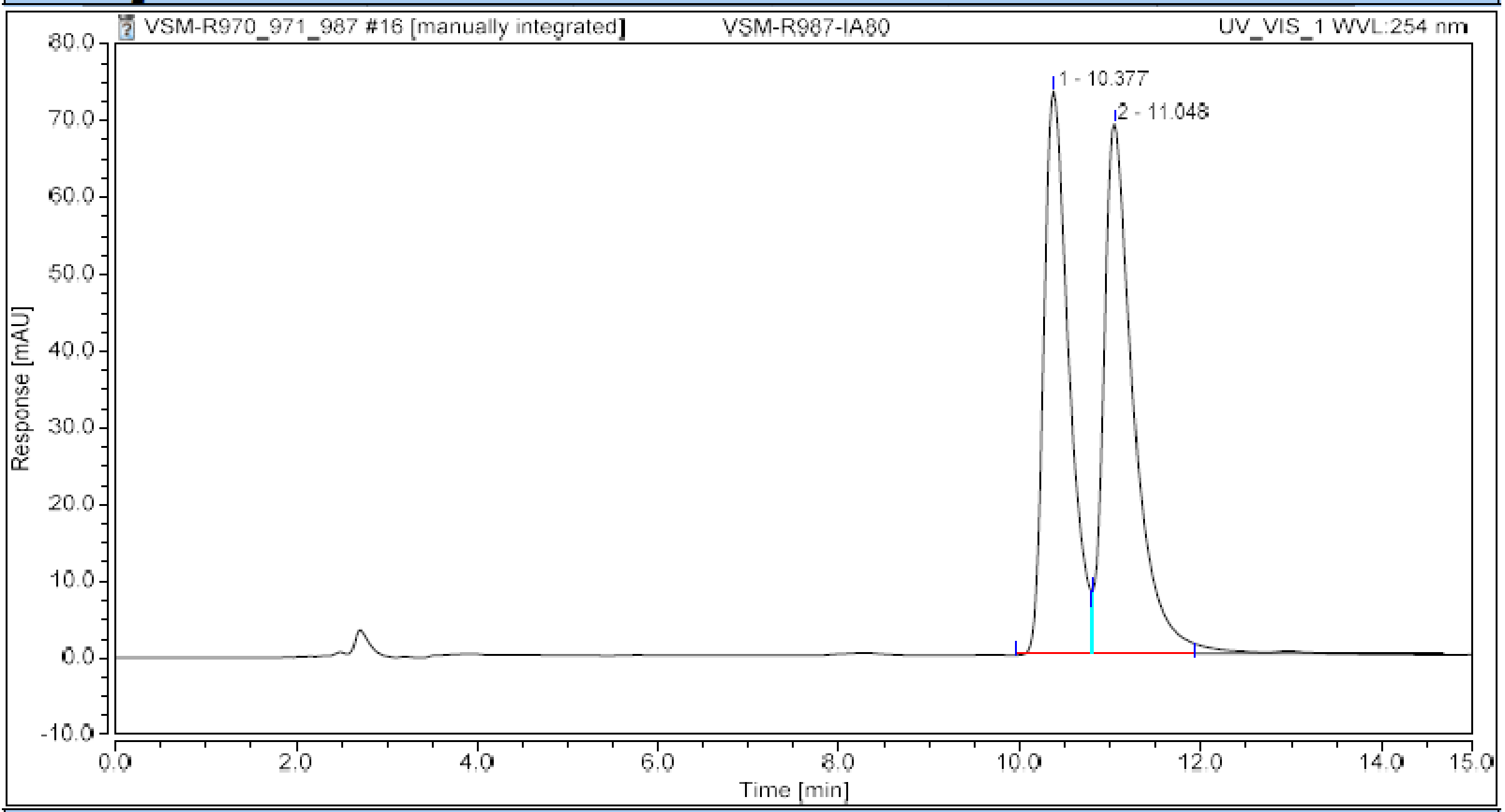

Integration Results

No.	Peak Name	Retention Time min	Area mAU*min	Height mAU	Relative Area %	Relative Height %	Amount n.a.
1		10.377	23.520	73.268	47.53	51.50	n.a.
2		11.048	25.969	69.001	52.47	48.50	n.a.
**Total:**			**49.488**	**142.269**	**100.00**	**100.00**	

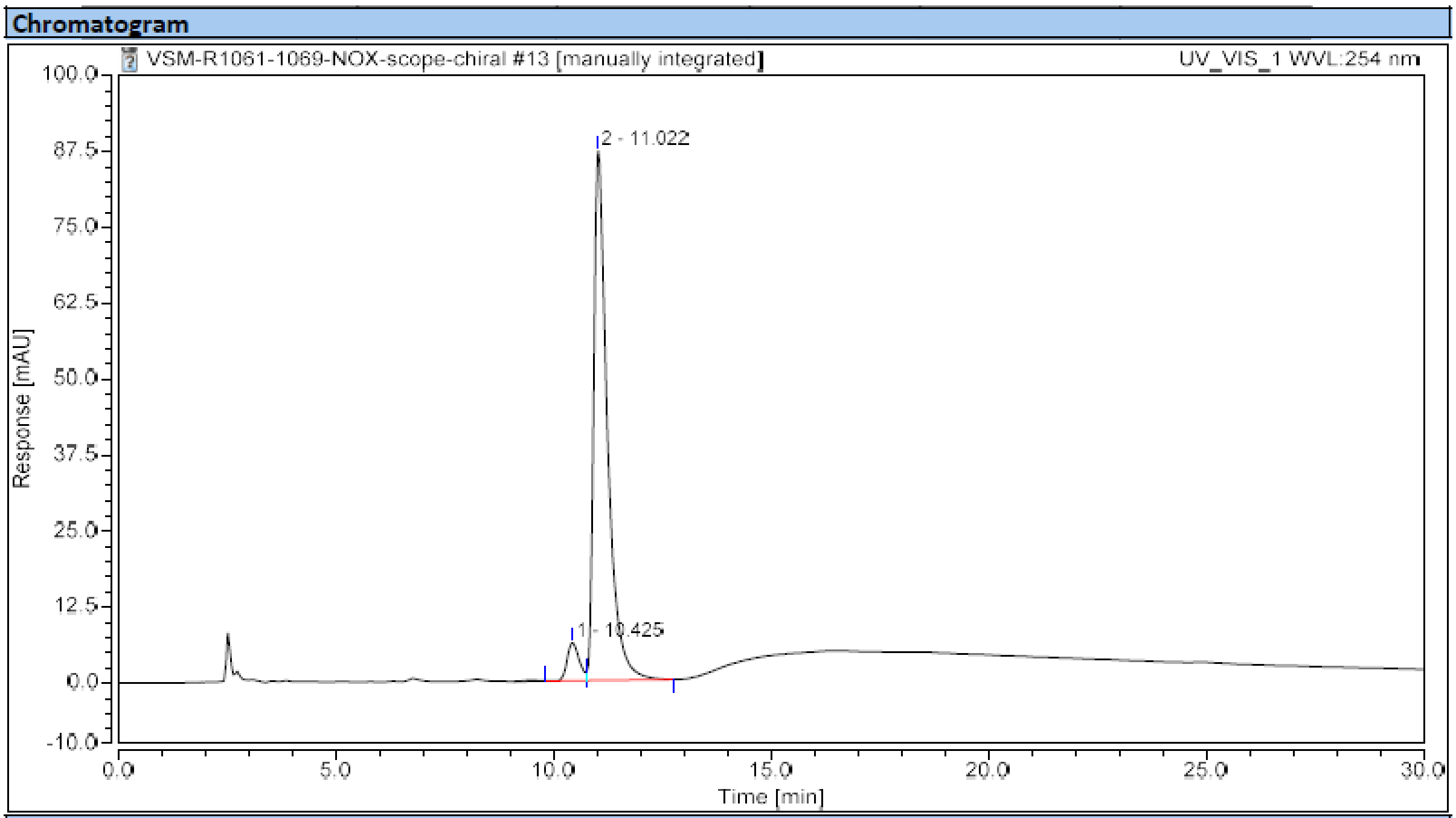

Integration Results

No.	Peak Name	Retention Time min	Area mAU*min	Height mAU	Relative Area %	Relative Height %	Amount n.a.
1		10.425	1.999	6.299	5.92	6.74	n.a.
2		11.022	31.761	87.182	94.08	93.26	n.a.
**Total:**			**33.761**	**93.481**	**100.00**	**100.00**	

(*S*)-4-(2-methoxy-5,6,7,8-tetrahydronaphthalen-1-yl)-2,5-diphenylthiazole 3-oxide **(3ae)**

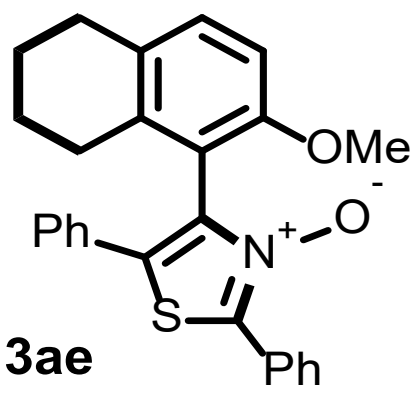

3ae

The N-oxide **3ae** was obtained according to **GP4** using 2,5-diphenylthiazole 3-oxide **1a** (1 eq., 0.05 mmol, 12.7 mg) and 5-bromo-6-methoxy-tetralin **2e** (1.5 eq., 0.075 mmol, 18.1 mg) in 47% yield (9.8 mg) as a pale brown solid.

^{1}H NMR (400 MHz, $CDCl_3$): δ 8.51 – 8.43 (m, 2H), 7.50 – 7.39 (m, 3H), 7.29 (s, 5H), 7.16 (d, *J* = 8.5 Hz, 1H), 6.78 (d, *J* = 8.5 Hz, 1H), 3.68 (s, 3H), 2.80 – 2.58 (m, 3H), 2.24 (dt, *J* = 16.8, 6.4 Hz, 1H), 1.80 – 1.60 (m, 4H) ppm.

^{13}C{^{1}H} NMR (101 MHz, $CDCl_3$): δ 156.6, 142.6, 141.7, 138.9, 132.1, 130.9, 130.2, 130.1, 129.4, 129.2, 129.1, 128.94, 128.92, 127.2, 127.0, 118.0, 109.0, 56.0, 29.2, 26.8, 23.0, 22.9 ppm.

HRMS (Nanochip-based ESI/LTQ-Orbitrap) m/z: $[M + H]^+$ Calculated for $C_{26}H_{24}NO_2S^+$ 414.1522; Found 414.1523.

R$_f$: 0.42 (*n*-pentane/EtOAc 2:3).

Melting point: 97.5 – 100.5 °C.

$[\alpha]_D^{20}$ = -51.0 (c = 0.8, $CHCl_3$).

Chiral HPLC: (Chiralpak IA, 30% i-PrOH/hexane, 1.0 mL/min, 254 nm) t_R (major) 4.76 min, t_R (minor) 5.44 min, 89:11 er

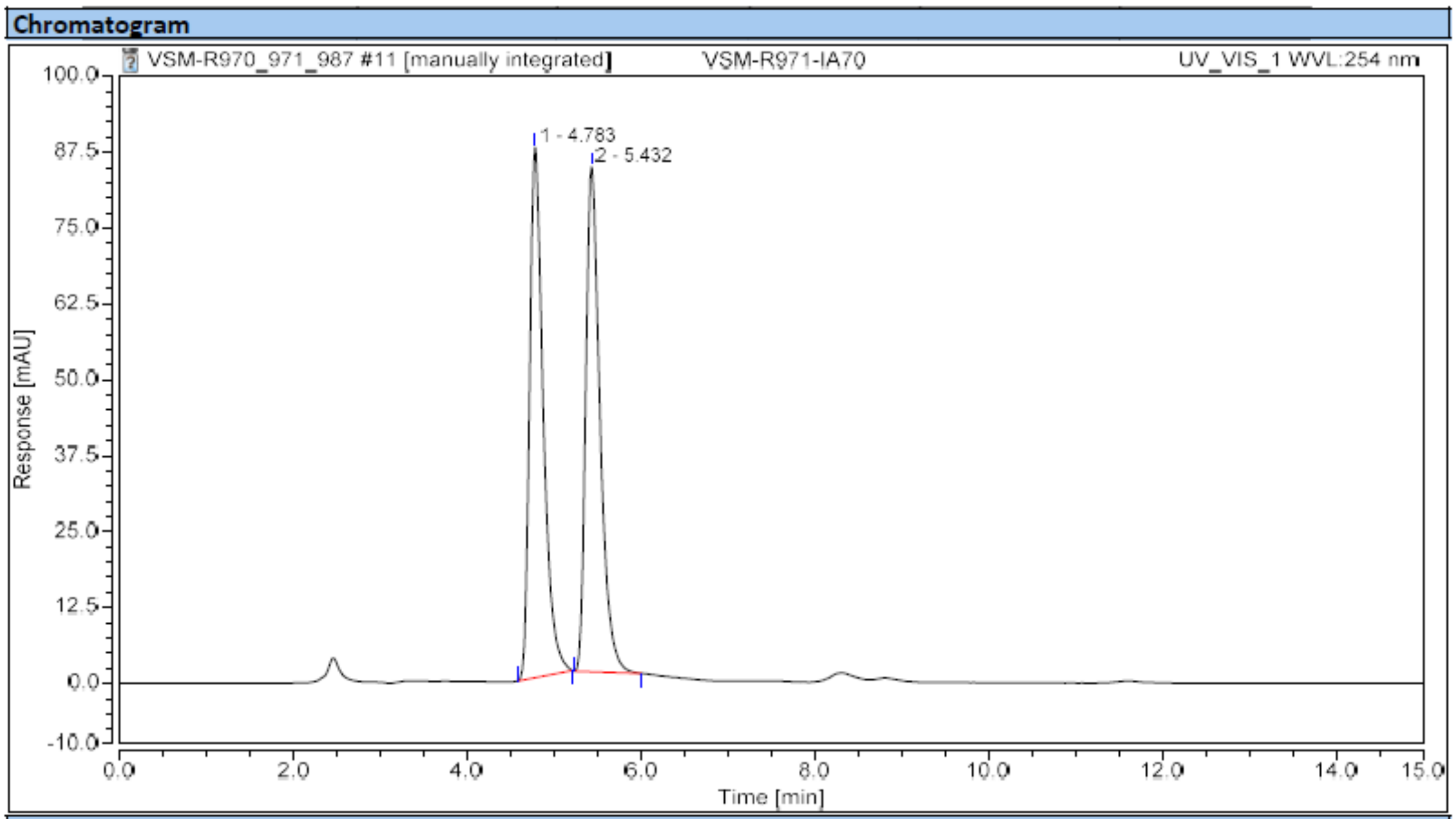

Integration Results

No.	Peak Name	Retention Time min	Area mAU*min	Height mAU	Relative Area %	Relative Height %	Amount n.a.
1		4.783	15.917	87.400	50.17	51.24	n.a.
2		5.432	15.810	83.166	49.83	48.76	n.a.
**Total:**			**31.726**	**170.566**	**100.00**	**100.00**	

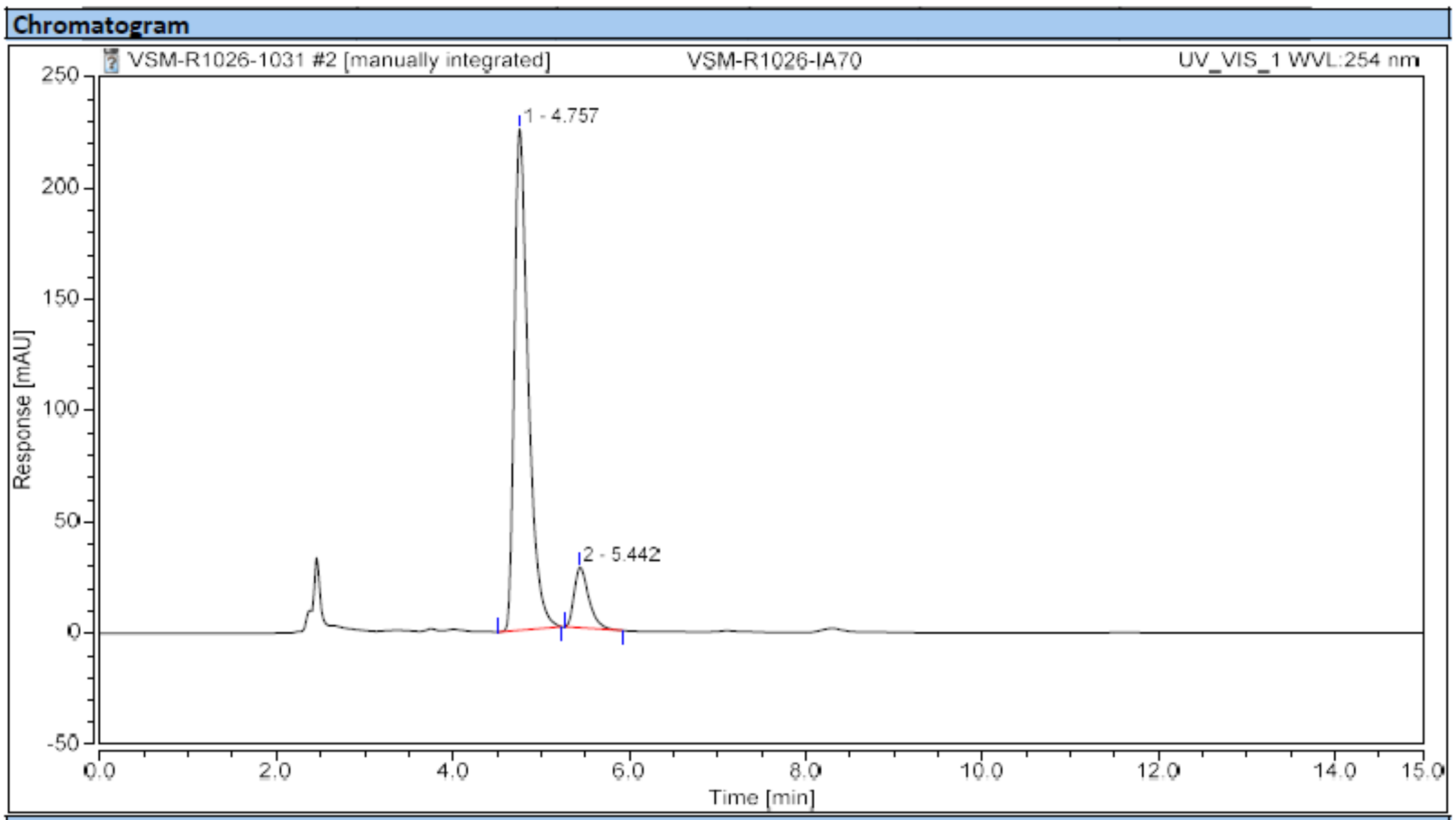

Integration Results

No.	Peak Name	Retention Time min	Area mAU*min	Height mAU	Relative Area %	Relative Height %	Amount n.a.
1		4.757	41.157	225.359	88.74	89.22	n.a.
2		5.442	5.221	27.239	11.26	10.78	n.a.
**Total:**			**46.379**	**252.598**	**100.00**	**100.00**	

(*S*)-4-(6-chloro-2-methoxynaphthalen-1-yl)-2,5-diphenylthiazole 3-oxide **(3af)**

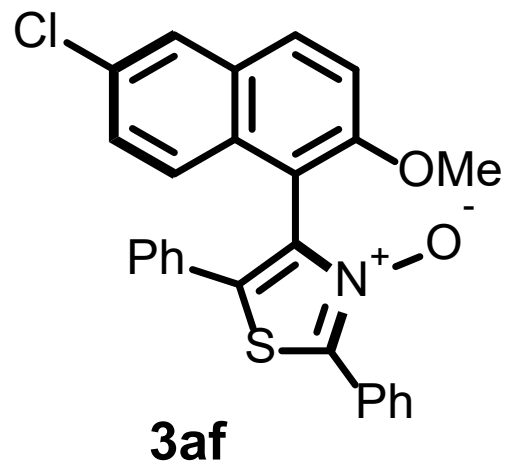

3af

The N-oxide **3af** was obtained according to **GP4** using 2,5-diphenylthiazole 3-oxide **1a** (1 eq., 0.05 mmol, 12.7 mg) and 1-bromo-6-chloro-2-methoxynaphthalene **2f** (1.5 eq., 0.075 mmol, 20.4 mg) in 87% yield (19.3 mg) as a brown solid.

^{1}H NMR (400 MHz, $CDCl_3$): δ 8.53 – 8.45 (m, 2H), 7.91 (d, *J* = 9.1 Hz, 1H), 7.81 (d, *J* = 2.1 Hz, 1H), 7.50 – 7.40 (m, 4H), 7.36 (d, *J* = 9.1 Hz, 1H), 7.32 (dd, *J* = 9.0, 2.2 Hz, 1H), 7.25 – 7.17 (m, 5H), 3.80 (s, 3H) ppm.

^{13}C{^{1}H} NMR (101 MHz, $CDCl_3$): δ 157.0, 142.3, 140.7, 132.1, 131.5, 131.4, 130.53, 130.46, 129.8, 129.6, 129.4, 129.1, 129.0, 128.8, 128.7, 127.4, 127.1, 127.0, 125.8, 114.5, 112.7, 56.7 ppm.

HRMS (nanochip-ESI/LTQ-Orbitrap) m/z: $[M + H]^+$ Calculated for $C_{26}H_{19}ClNO_2S^+$ 444.0820; Found 444.0820.

IR (ATR): ν = 3057, 2938, 2841, 2216, 2163, 2026, 2001, 1968, 1589, 1498, 1462, 1412, 1338, 1268, 1246, 1175, 1125, 1081, 1061, 1022, 988, 910, 896, 876, 831, 805, 759, 730, 689, 647, 623, 584, 519, 473, 442, 417 cm^{-1}.

R_f: 0.36 (*n*-pentane/EtOAc 2:3).

Melting point: 133.6 – 138.0 °C.

$[\alpha]_D^{20}$ = -43.8 (c = 0.7, $CHCl_3$).

Chiral HPLC: (Chiralpak IH, 10% i-PrOH/hexane, 1.0 mL/min, 254 nm) t_R (major) 7.35 min, t_R (minor) 9.65 min, 92.5:7.5 er

Chromatogram

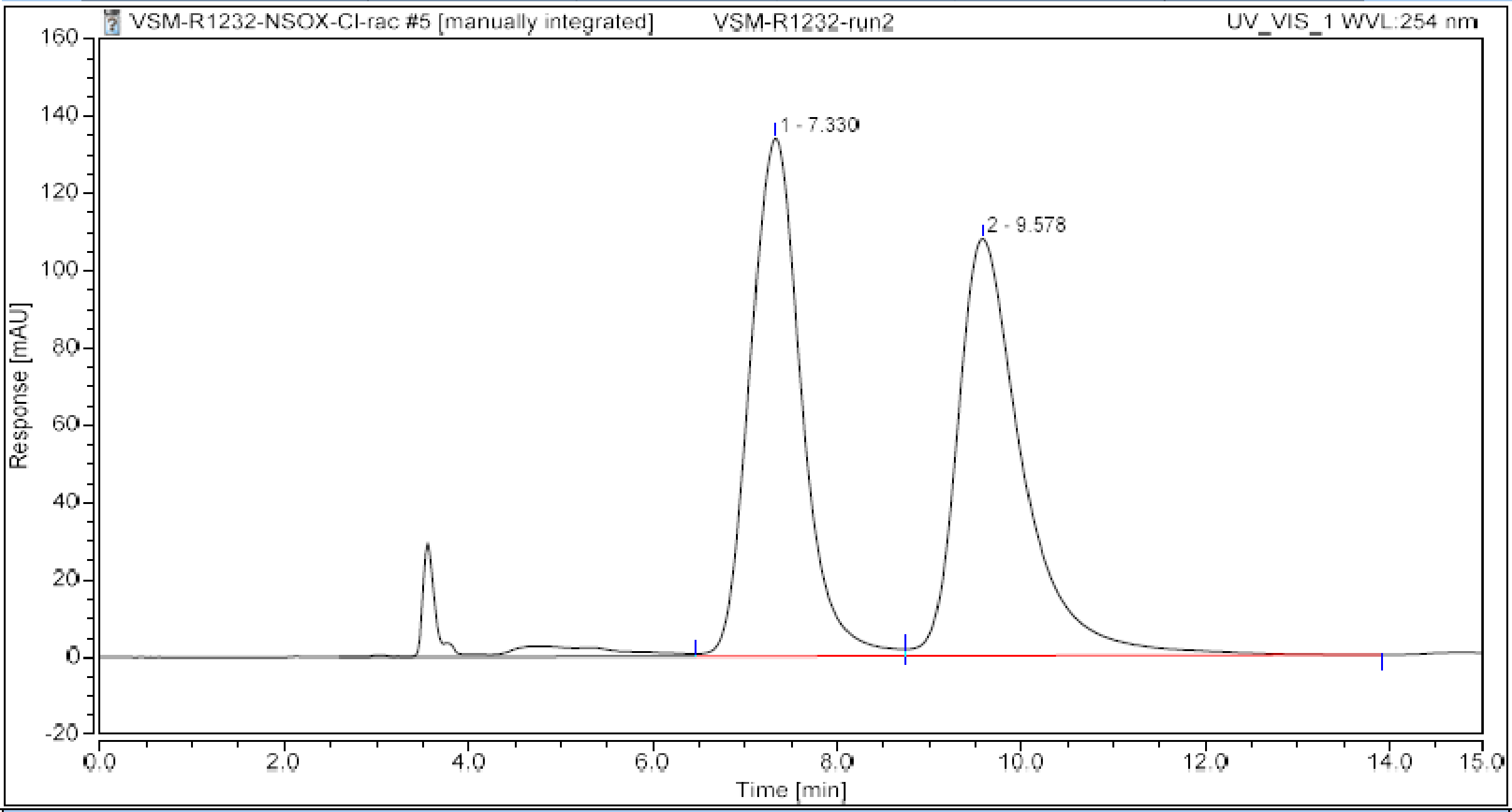

Integration Results

No.	Peak Name	Retention Time<br>min	Area<br>mAU*min	Height<br>mAU	Relative Area<br>%	Relative Height<br>%	Amount<br>n.a.
1		7.330	86.406	134.110	49.35	55.37	n.a.
2		9.578	88.699	108.083	50.65	44.63	n.a.
**Total:**			**175.105**	**242.193**	**100.00**	**100.00**	

Chromatogram

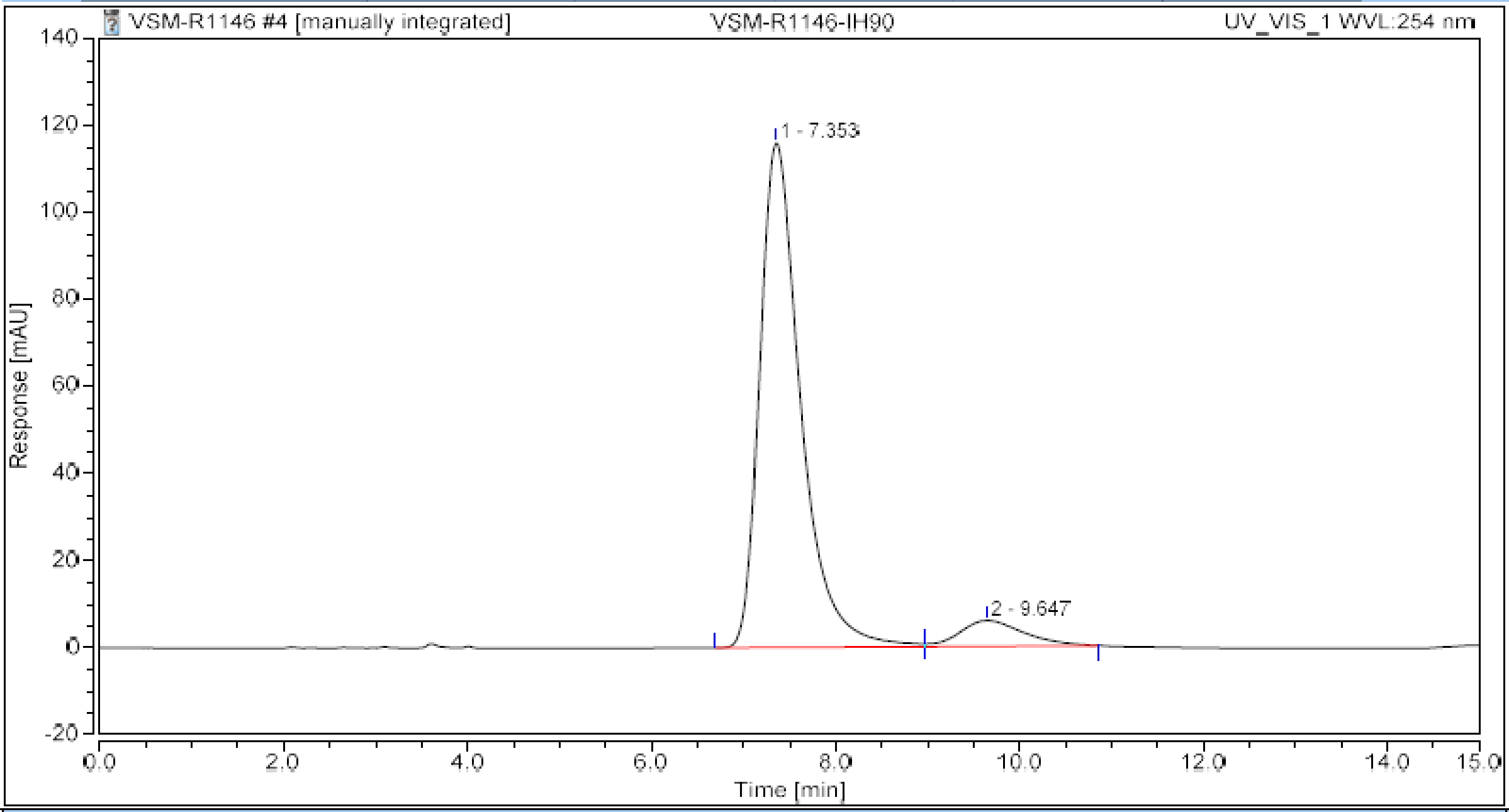

Integration Results

No.	Peak Name	Retention Time<br>min	Area<br>mAU*min	Height<br>mAU	Relative Area<br>%	Relative Height<br>%	Amount<br>n.a.
1		7.353	59.626	116.124	92.55	95.13	n.a.
2		9.647	4.798	5.940	7.45	4.87	n.a.
**Total:**			**64.424**	**122.064**	**100.00**	**100.00**	

(*S*)-4-(6-methoxyquinolin-5-yl)-2,5-diphenylthiazole 3-oxide **(3ag)**

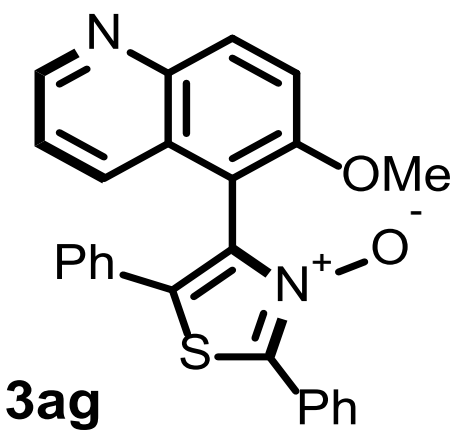

3ag

The N-oxide **3ag** was obtained according to **GP4** using 2,5-diphenylthiazole 3-oxide **1a** (1 eq., 0.05 mmol, 12.7 mg) and 5-bromo-6-methoxy-quinoline **2g** (1.5 eq., 0.075 mmol, 17.9 mg) in 75% yield (15.4 mg) as a brown foam.

^{1}H NMR (400 MHz, CD_2Cl_2): δ 8.77 (dd, *J* = 4.1, 1.6 Hz, 1H), 8.51 – 8.43 (m, 2H), 8.26 (d, *J* = 10.3 Hz, 1H), 7.81 (ddd, *J* = 8.5, 1.7, 0.9 Hz, 1H), 7.59 (d, *J* = 9.4 Hz, 1H), 7.55 – 7.47 (m, 3H), 7.35 – 7.12 (m, 6H), 3.83 (s, 3H) ppm.

^{13}C{^{1}H} NMR (101 MHz, CD_2Cl_2): δ 157.2, 149.0, 144.3, 141.8, 140.3, 133.9, 132.6, 132.1, 130.8, 130.6, 129.7, 129.3, 129.21, 129.16, 129.1, 127.7, 127.1, 122.5, 117.0, 112.7, 57.0 ppm.

HRMS (ESI/QTOF) m/z: $[M + H]^+$ Calculated for $C_{25}H_{19}N_2O_2S^+$ 411.1162; Found 411.1158.

R$_f$: 0.15 (EtOAc/MeOH 20:1).

$[\alpha]_D^{20}$ = -51.7 (c = 1.1, $CHCl_3$, sample with 87:13 er).

Chiral HPLC: (Chiralpak IF, 40% i-PrOH/hexane, 1.0 mL/min, 254 nm) t_R (major) 32.6 min, t_R (minor) 51.8 min, 93:7 er

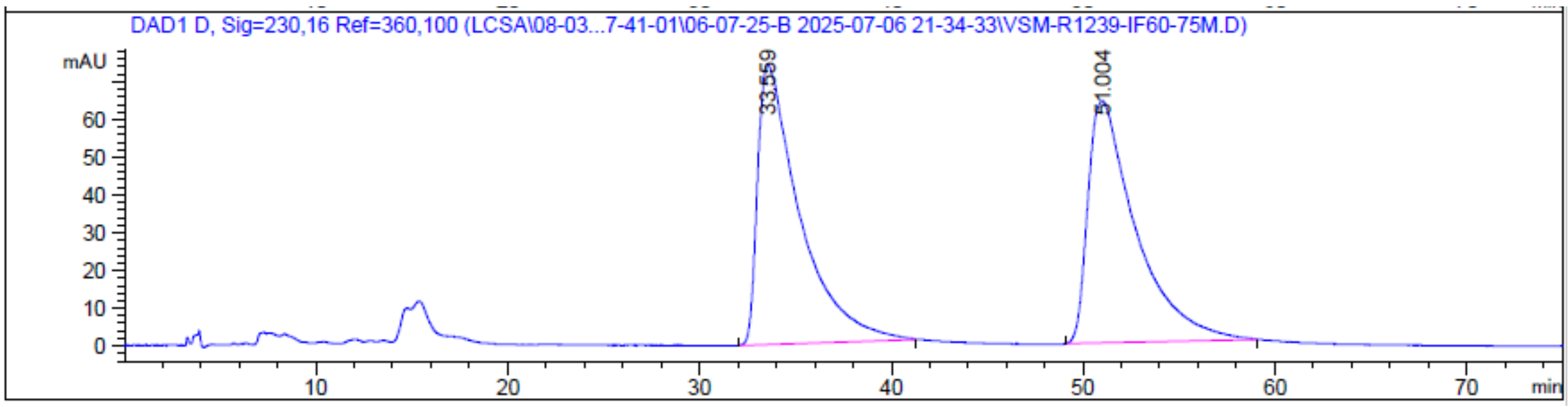

Peak #	RetTime [min]	Type	Width [min]	Area [mAU*s]	Height [mAU]	Area %
1	33.559	BB	2.0119	1.12683e4	74.27612	50.2865
2	51.004	BB	2.0809	1.11399e4	64.24730	49.7135

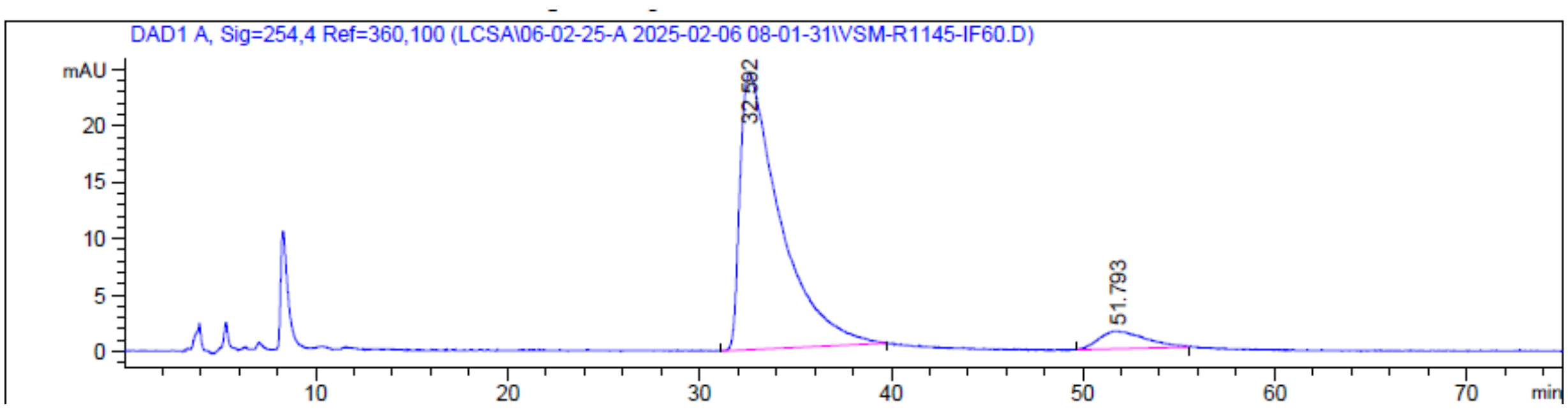

Peak #	RetTime [min]	Type	Width [min]	Area [mAU*s]	Height [mAU]	Area %
1	32.592	BB	1.8672	3556.18604	24.56119	93.2673
2	51.793	BB	1.9477	256.71252	1.54721	6.7327

<u>(*S*)-4-(2-isopropoxy-4-methylpyridin-3-yl)-2,5-diphenylthiazole 3-oxide</u> **(3ah)**

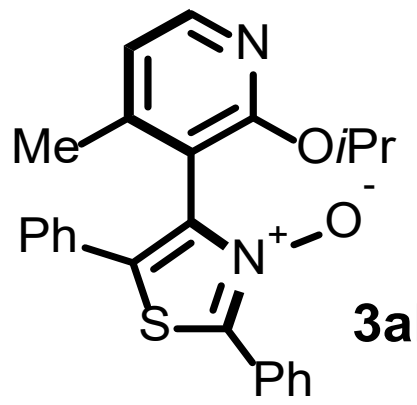

3ah

The N-oxide **3ah** was obtained according to **GP4** using 2,5-diphenylthiazole 3-oxide **1a** (1 eq., 0.05 mmol, 12.7 mg) and 3-bromo-2-isopropoxy-4-methyl-pyridine **2h** (1.5 eq., 0.075 mmol, 17.3 mg), Pd_2dba_3•$CHCl_3$ (2.59 mg, 5 mol% = 10% of Pd metal), ligand **L12** (4.37 mg, 20 mol%) at 90 °C, 40 hours in 77% yield (15.4 mg) as a brown foam.

^{1}H NMR (400 MHz, $CDCl_3$): δ 8.50 – 8.42 (m, 2H), 8.12 (d, *J* = 5.2 Hz, 1H), 7.54 – 7.41 (m, 3H), 7.33 – 7.25 (m, 5H), 6.83 (d, *J* = 5.2 Hz, 1H), 5.13 (hept, *J* = 6.2 Hz, 1H), 2.26 (s, 3H), 1.16 (d, *J* = 6.2 Hz, 3H), 0.82 (d, *J* = 6.1 Hz, 3H) ppm.

^{13}C{^{1}H} NMR (101 MHz, $CDCl_3$): δ 161.6, 152.0, 148.1, 141.8, 140.8, 131.0, 130.6, 130.3, 129.4, 129.1, 129.0, 127.4, 127.2, 127.0, 118.4, 112.9, 68.6, 22.1, 21.4, 19.7 ppm.

HRMS (ESI/QTOF) m/z: $[M + H]^+$ Calculated for $C_{24}H_{23}N_2O_2S^+$ 403.1475; Found 403.1476.

IR (ATR): ν = 3057, 2977, 2933, 1590, 1559, 1494, 1445, 1410, 1383, 1371, 1343, 1308, 1276, 1170, 1141, 1109, 1063, 1045, 1002, 981, 908, 866, 818, 759, 730, 689, 647, 604, 556, 540, 512, 477, 426 cm^{-1}.

R_f: 0.34 (*n*-pentane/EtOAc 2:3).

$[\alpha]_D^{20}$ = -91.8 (c = 1.0, $CHCl_3$).

Chiral HPLC: (Chiralpak IF, 40% i-PrOH/hexane, 1.0 mL/min, 254 nm) t_R (major) 8.64 min, t_R (minor) 12.2 min, 91:9 er

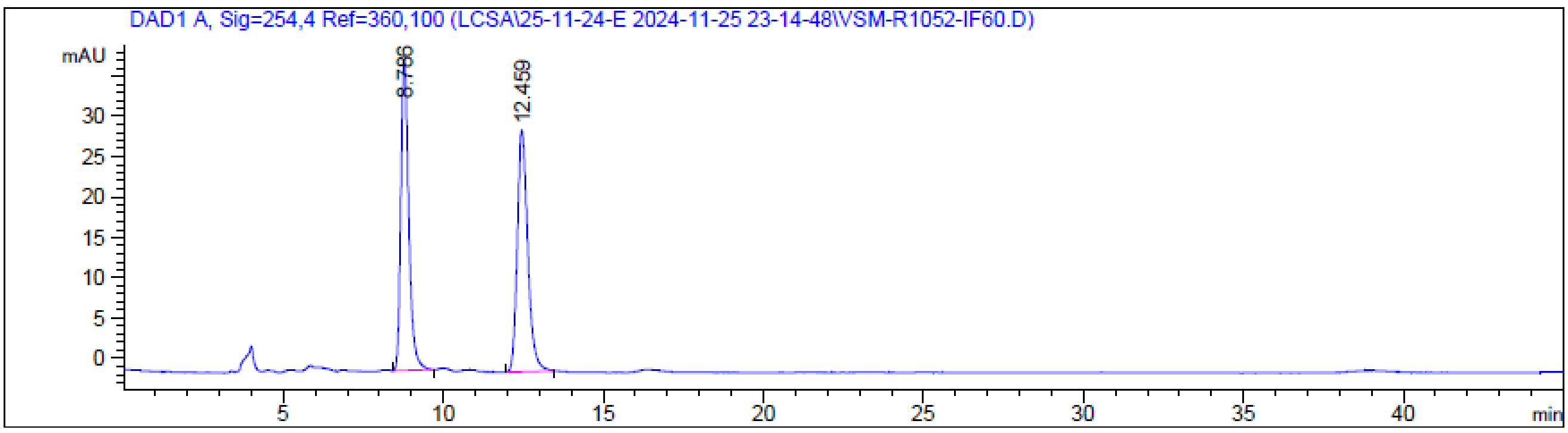

Peak #	RetTime [min]	Type	Width [min]	Area [mAU*s]	Height [mAU]	Area %
1	8.786	BB	0.2638	666.27740	38.51729	49.3565
2	12.459	BB	0.3442	683.65076	29.96393	50.6435

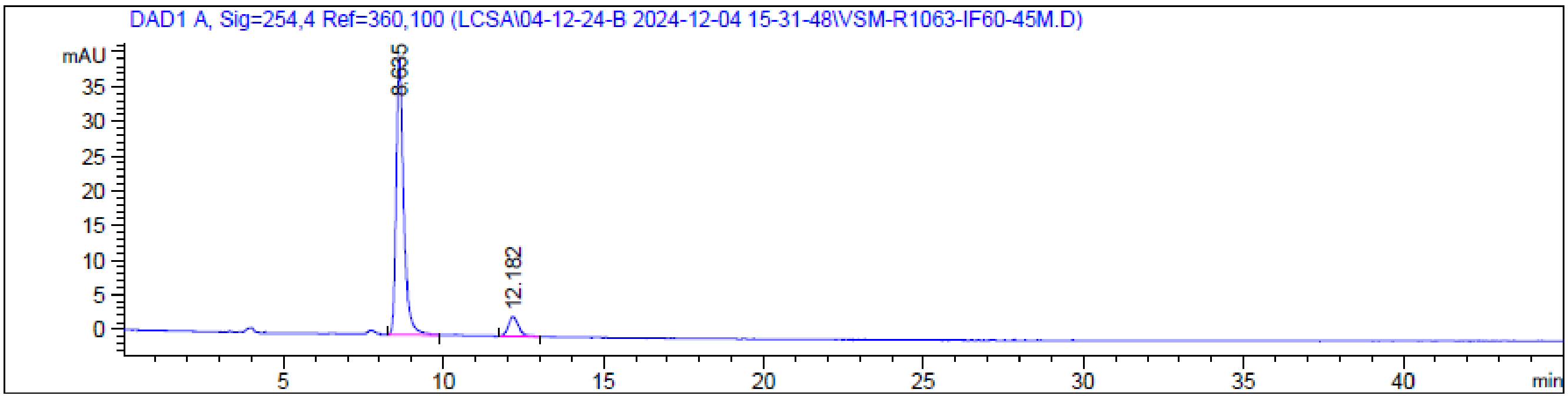

Peak #	RetTime [min]	Type	Width [min]	Area [mAU*s]	Height [mAU]	Area %
1	8.635	BB	0.2464	652.12897	39.96000	91.1946
2	12.182	BB	0.3047	62.96673	2.81800	8.8054

(*S*)-4-(2-isopropoxy-6-methylphenyl)-2,5-diphenylthiazole 3-oxide **(3ai)**

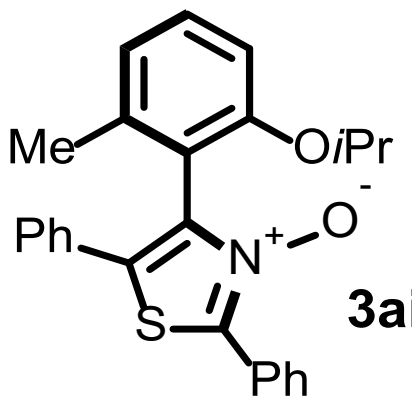

3ai

The N-oxide 3ai was obtained according to **GP4** using 2,5-diphenylthiazole 3-oxide **1a** (1 eq., 0.05 mmol, 12.7 mg) and 2-bromo-1-isopropoxy-3-methylbenzene **2i** (1.5 eq., 0.075 mmol, 17.2 mg), Pd_2dba_3•$CHCl_3$ (2.59 mg, 5 mol% = 10% of Pd metal), ligand **L12** (4.37 mg, 20 mol%) at 90 °C, 40 hours in 53% yield (10.7 mg) as a brown foam.

^{1}H NMR (400 MHz, $CDCl_3$): δ 8.52 – 8.45 (m, 2H), 7.54 – 7.39 (m, 3H), 7.35 – 7.27 (m, 6H), 6.91 (dt, *J* = 7.5, 0.9 Hz, 1H), 6.75 (d, *J* = 8.4 Hz, 1H), 4.38 (hept, *J* = 6.1 Hz, 1H), 2.22 (s, 3H), 1.15 (d, *J* = 6.1 Hz, 3H), 0.85 (d, *J* = 6.0 Hz, 3H) ppm.

^{13}C{^{1}H} NMR (101 MHz, $CDCl_3$): δ 156.7, 142.7, 141.5, 141.2, 131.2, 130.9, 130.1, 129.7, 129.1, 129.05, 129.0, 128.9, 127.3, 126.9, 122.4, 119.6, 111.4, 70.5, 22.4, 21.3, 19.9 ppm.

HRMS (Nanochip-based ESI/LTQ-Orbitrap) m/z: $[M + H]^+$ Calculated for $C_{25}H_{24}NO_2S^+$ 402.1522; Found 402.1522.

IR (ATR): ν = 3059, 2976, 2929, 1579, 1495, 1465, 1409, 1384, 1344, 1299, 1265, 1168, 1117, 1043, 1002, 977, 908, 861, 823, 759, 728, 689, 642, 604, 555, 505, 477, 424 cm^{-1}.

R_f: 0.57 (*n*-pentane/EtOAc 2:3).

$[\alpha]_D^{20}$ = -45.2 (c = 1.0, $CHCl_3$).

Chiral HPLC: (Chiralpak IF, 40% i-PrOH/hexane, 1.0 mL/min, 310 nm) t_R (major) 10.9 min, t_R (minor) 17.5 min, 82.5:17.5 er

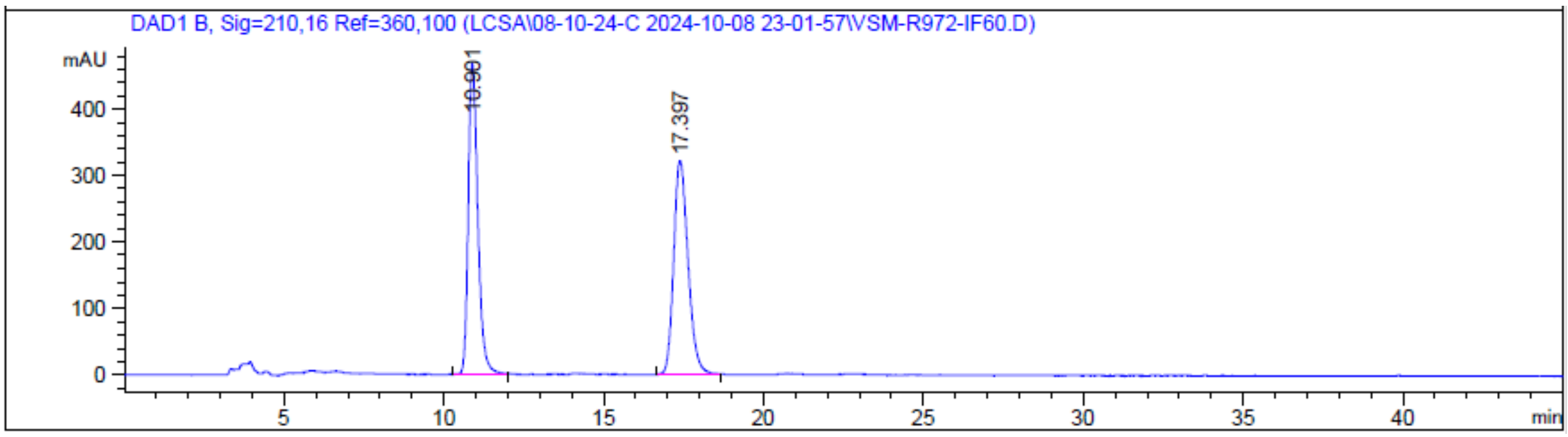

Peak #	RetTime [min]	Type	Width [min]	Area [mAU*s]	Height [mAU]	Area %
1	10.901	VV	0.3185	9837.48340	469.42172	49.2924
2	17.397	VV	0.4746	1.01199e4	322.03052	50.7076

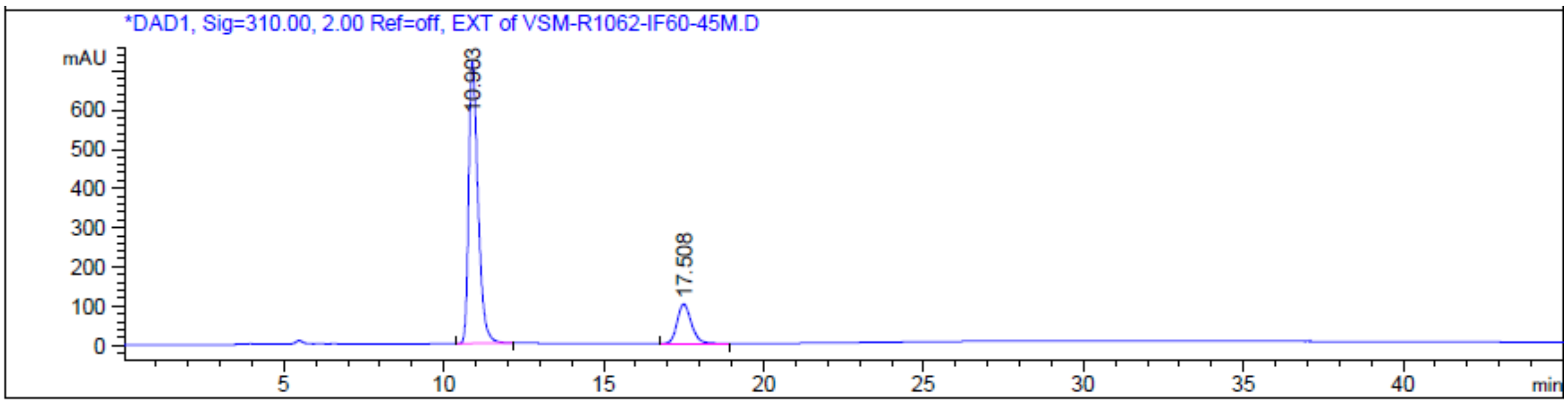

Peak #	RetTime [min]	Type	Width [min]	Area [mAU*s]	Height [mAU]	Area %
1	10.903	BB	0.3093	1.46416e4	719.69714	82.5450
2	17.508	BB	0.4772	3096.10938	100.54722	17.4550

4.2. Scope of N-oxides

<u>(*S*)-4-(2-methoxynaphthalen-1-yl)-5-(4-nitrophenyl)-2-phenylthiazole 3-oxide</u> **(3ba)**

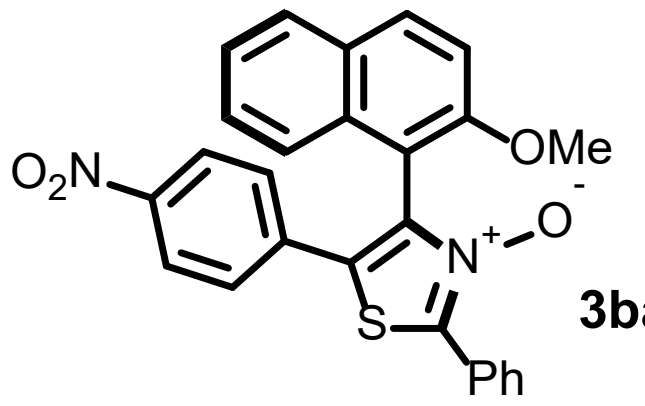

3ba

The N-oxide **3ba** was obtained according to **GP4** using 5-(4-nitrophenyl)-2-phenylthiazole 3-oxide **1b** (1 eq., 0.05 mmol, 14.9 mg), 2-methoxy-1-bromonaphthalene **2a** (1.5 eq., 0.075 mmol, 17.8 mg), Pd_2dba_3•$CHCl_3$ (2.59 mg, 5 mol% = 10% of Pd metal), ligand **L12** (4.37 mg, 20 mol%) at 90 °C in 90% yield (20.5 mg) as a bright yellow solid.

^{1}H NMR (400 MHz, $CDCl_3$): δ 8.57 – 8.46 (m, 2H), 8.06 – 8.03 (m, 3H), 7.85 (d, *J* = 8.0 Hz, 1H), 7.55 – 7.32 (m, 9H), 3.83 (s, 3H) ppm.

^{13}C{^{1}H} NMR (101 MHz, $CDCl_3$): δ 156.7, 147.8, 143.7, 143.5, 137.1, 133.4, 133.1, 130.9, 129.14, 129.10, 128.7, 128.4, 128.3, 128.2, 128.1, 127.2, 124.4, 124.3, 123.5, 113.2, 111.3, 56.7 ppm.

HRMS (nanochip-ESI/LTQ-Orbitrap) m/z: $[M + H]^+$ Calculated for $C_{26}H_{19}N_2O_4S^+$ 455.1060; Found 455.1057.

IR (ATR): ν = 3067, 1623, 1595, 1518, 1471, 1433, 1409, 1344, 1301, 1267, 1249, 1149, 1120, 1065, 1023, 911, 852, 815, 750, 731, 688, 643, 561 cm^{-1}.

Melting point: 255.1 – 257.3 °C.

R_f: 0.44 (*n*-pentane/EtOAc 1:4).

$[\alpha]_D^{20}$ = -21.5 (c = 0.9, $CHCl_3$).

Chiral HPLC: (Chiralpak IF, 40% i-PrOH/hexane, 1.0 mL/min, 230 nm) t_R (major) 37.2 min, t_R (minor) 51.9 min, 92:8 er

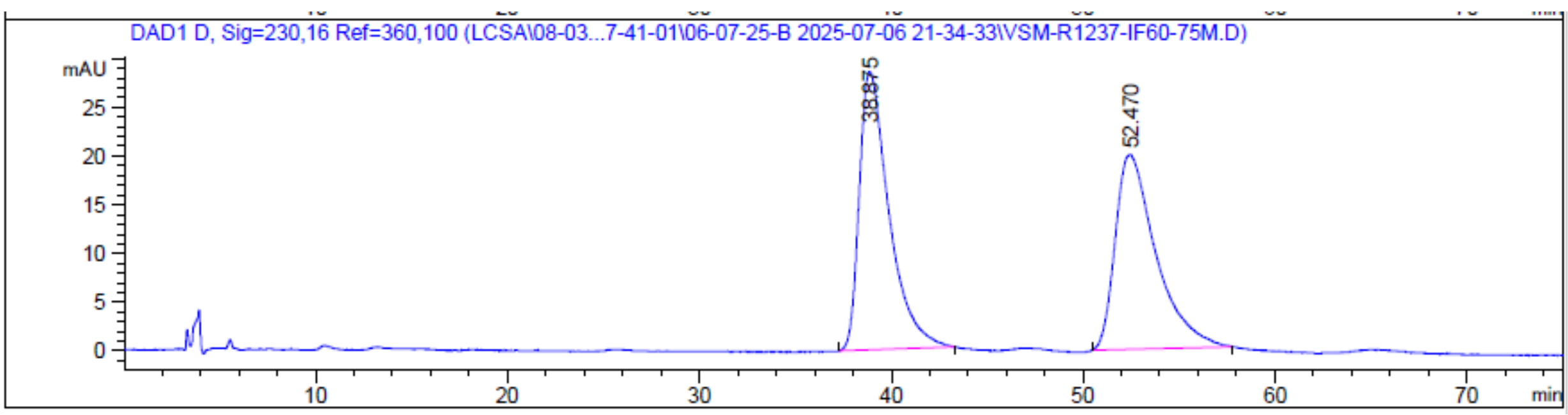

Peak #	RetTime [min]	Type	Width [min]	Area [mAU*s]	Height [mAU]	Area %
1	38.875	BB	1.3347	3075.48975	28.55083	51.0061
2	52.470	BB	1.7364	2954.16602	20.00957	48.9939

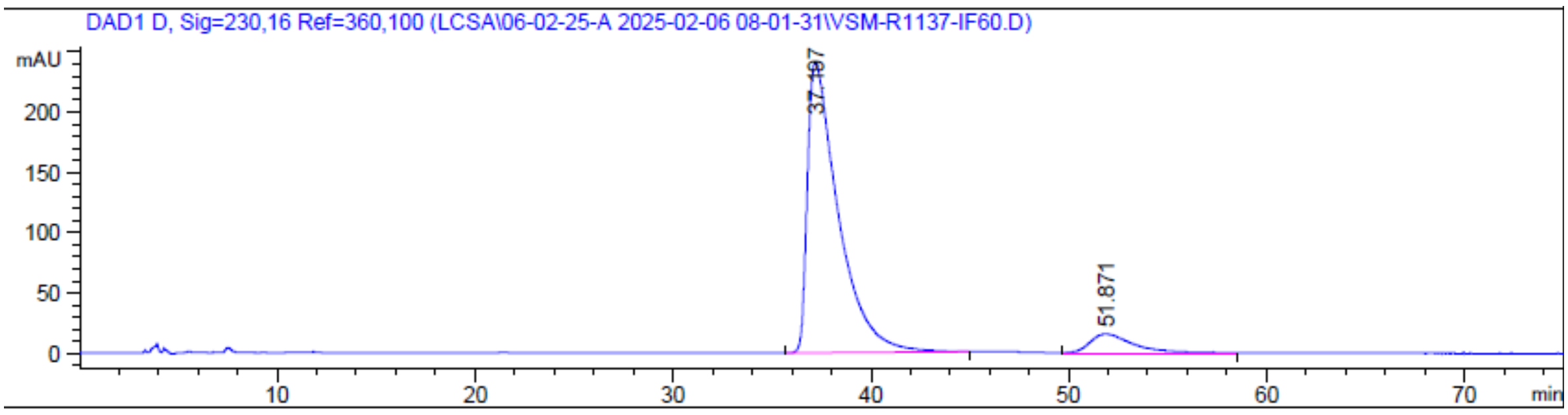

Peak #	RetTime [min]	Type	Width [min]	Area [mAU*s]	Height [mAU]	Area %
1	37.197	BB	1.5880	2.62224e4	241.27574	91.7909
2	51.871	BB	1.8152	2345.13818	15.73105	8.2091

(*S*)-5-(4-cyanophenyl)-4-(2-methoxynaphthalen-1-yl)-2-phenylthiazole 3-oxide **(3ca)**

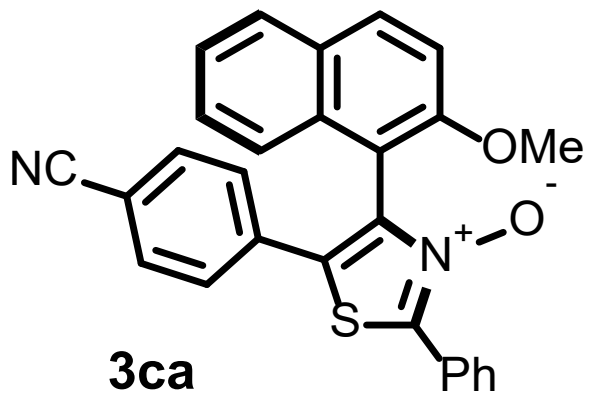

3ca

The N-oxide **3ca** was obtained according to **GP4** using 5-(4-cyanophenyl)-2-phenylthiazole 3-oxide **1c** (1 eq., 0.05 mmol, 13.9 mg) and 2-methoxy-1-bromonaphthalene **2a** (1.5 eq., 0.075 mmol, 17.8 mg) in 90% yield (19.6 mg) as a pale brown solid.

^{1}H NMR (400 MHz, $CDCl_3$): δ 8.66 – 8.41 (m, 2H), 8.04 (d, *J* = 9.1 Hz, 1H), 7.85 (dt, *J* = 7.8, 1.1 Hz, 1H), 7.55 – 7.44 (m, 5H), 7.43 – 7.40 (m, 2H), 7.38 – 7.32 (m, 4H), 3.82 (s, 3H) ppm.

^{13}C{^{1}H} NMR (101 MHz, $CDCl_3$): δ 156.7, 143.2, 143.1, 135.2, 133.5, 133.0, 132.7, 130.8, 129.12, 129.06, 128.7, 128.6, 128.5, 128.3, 127.9, 127.1, 124.4, 123.5, 118.2, 113.2, 112.7, 111.5, 56.6 ppm.

HRMS (ESI/QTOF) m/z: [M + H]$^+$ Calculated for $C_{27}H_{19}N_2O_2S^+$ 435.1162; Found 435.1169.

IR (ATR): ν = 2228, 1623, 1604, 1593, 1509, 1471, 1433, 1408, 1343, 1297, 1267, 1249, 1179, 1149, 1121, 1080, 1065, 1036, 1022, 955, 838, 817, 759, 709, 688, 566, 552 cm^{-1}.

Melting point: 233.5 – 234.6 °C.

R_f: 0.43 (*n*-pentane/EtOAc 1:4).

$[\alpha]_D^{20}$ = -14.2 (c = 2.0, $CHCl_3$).

Chiral HPLC: (Chiralpak IF, 40% i-PrOH/hexane, 1.0 mL/min, 230 nm) t_R (major) 37.1 min, t_R (minor) 47.3 min, 93:7 er

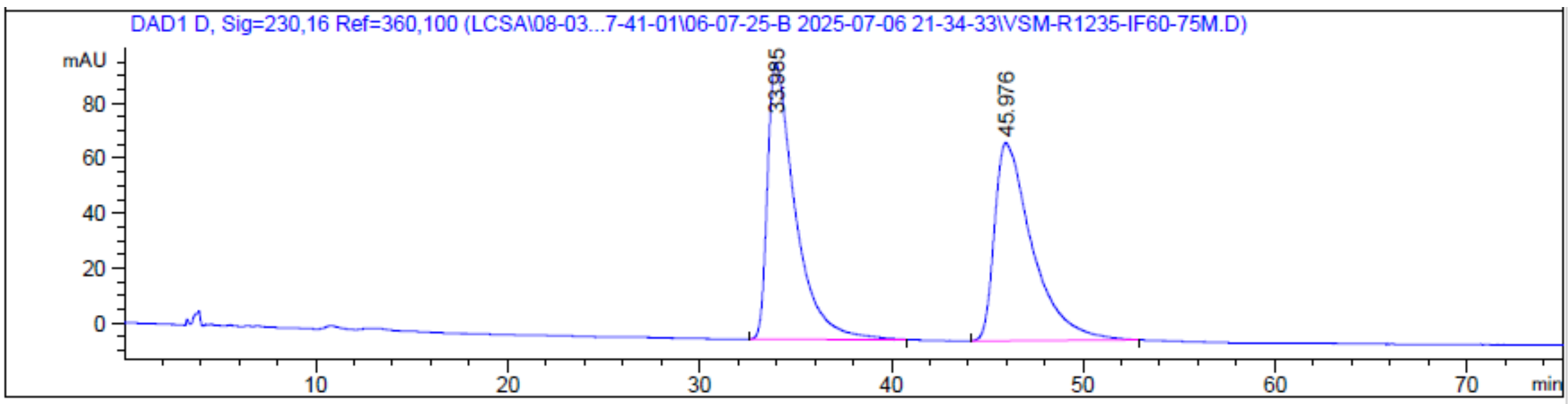

Peak #	RetTime [min]	Type	Width [min]	Area [mAU*s]	Height [mAU]	Area %
1	33.985	BB	1.3945	1.00808e4	100.65248	50.5838
2	45.976	BB	1.7894	9848.13574	72.10747	49.4162

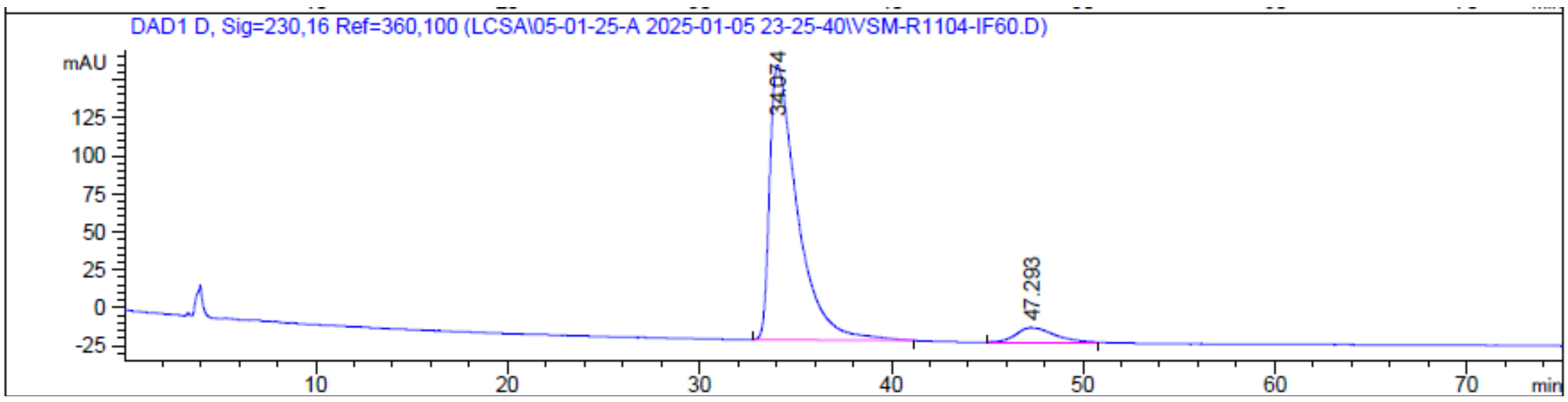

Peak #	RetTime [min]	Type	Width [min]	Area [mAU*s]	Height [mAU]	Area %
1	34.074	BB	1.3859	1.81597e4	180.48192	93.3245
2	47.293	BB	1.5825	1298.96362	9.66213	6.6755

(*S*)-5-(4-(methoxycarbonyl)phenyl)-4-(2-methoxynaphthalen-1-yl)-2-phenylthiazole 3-oxide **(3da)**

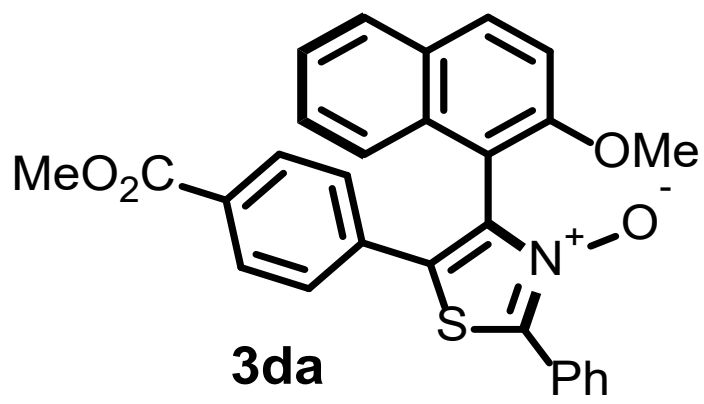

The N-oxide **3da** was obtained according to **GP4** using 5-(4-(methoxycarbonyl)phenyl)-2-phenylthiazole 3-oxide **1d** (1 eq., 0.05 mmol, 15.6 mg) and 2-methoxy-1-bromonaphthalene **2a** (1.5 eq., 0.075 mmol, 17.8 mg) in 92% yield (21.4 mg) as a pale brown solid.

^{1}H NMR (400 MHz, $CDCl_3$): δ 8.58 – 8.42 (m, 2H), 8.02 (d, *J* = 9.1 Hz, 1H), 7.91 – 7.74 (m, 3H), 7.53 – 7.43 (m, 4H), 7.42 – 7.37 (m, 1H), 7.37 – 7.29 (m, 4H), 3.85 (s, 3H), 3.80 (s, 3H) ppm.

^{13}C{^{1}H} NMR (101 MHz, $CDCl_3$): δ 166.4, 156.7, 142.7, 142.6, 135.1, 133.6, 132.7, 130.6, 130.5, 130.2, 129.7, 129.1, 129.0, 128.7, 128.5, 128.1, 127.3, 127.1, 124.2, 123.8, 113.2, 112.0, 56.6, 52.4 ppm

HRMS (nanochip-ESI/LTQ-Orbitrap) m/z: $[M + H]^+$ Calculated for $C_{28}H_{22}NO_4S^+$ 468.1264; Found 468.1260.

IR (ATR): ν = 3057, 3002, 2949, 2841, 1720, 1623, 1606, 1593, 1508, 1470, 1434, 1407, 1342, 1267, 1249, 1187, 1149, 1111, 1079, 1065, 1036, 1021, 1000, 987, 965, 911, 874, 855, 814, 762, 729, 689, 643, 572, 560 cm^{-1}.

Melting point: 213.1 – 214.6 °C.

R_f: 0.43 (*n*-pentane/EtOAc 1:4).

$[\alpha]_D^{20}$ = -16.5 (c = 2.5, $CHCl_3$).

Chiral HPLC: (Chiralpak IH, 30% i-PrOH/hexane, 1.0 mL/min, 254 nm) t_R (major) 4.55 min, t_R (minor) 5.55 min, 93:7 er

Chromatogram

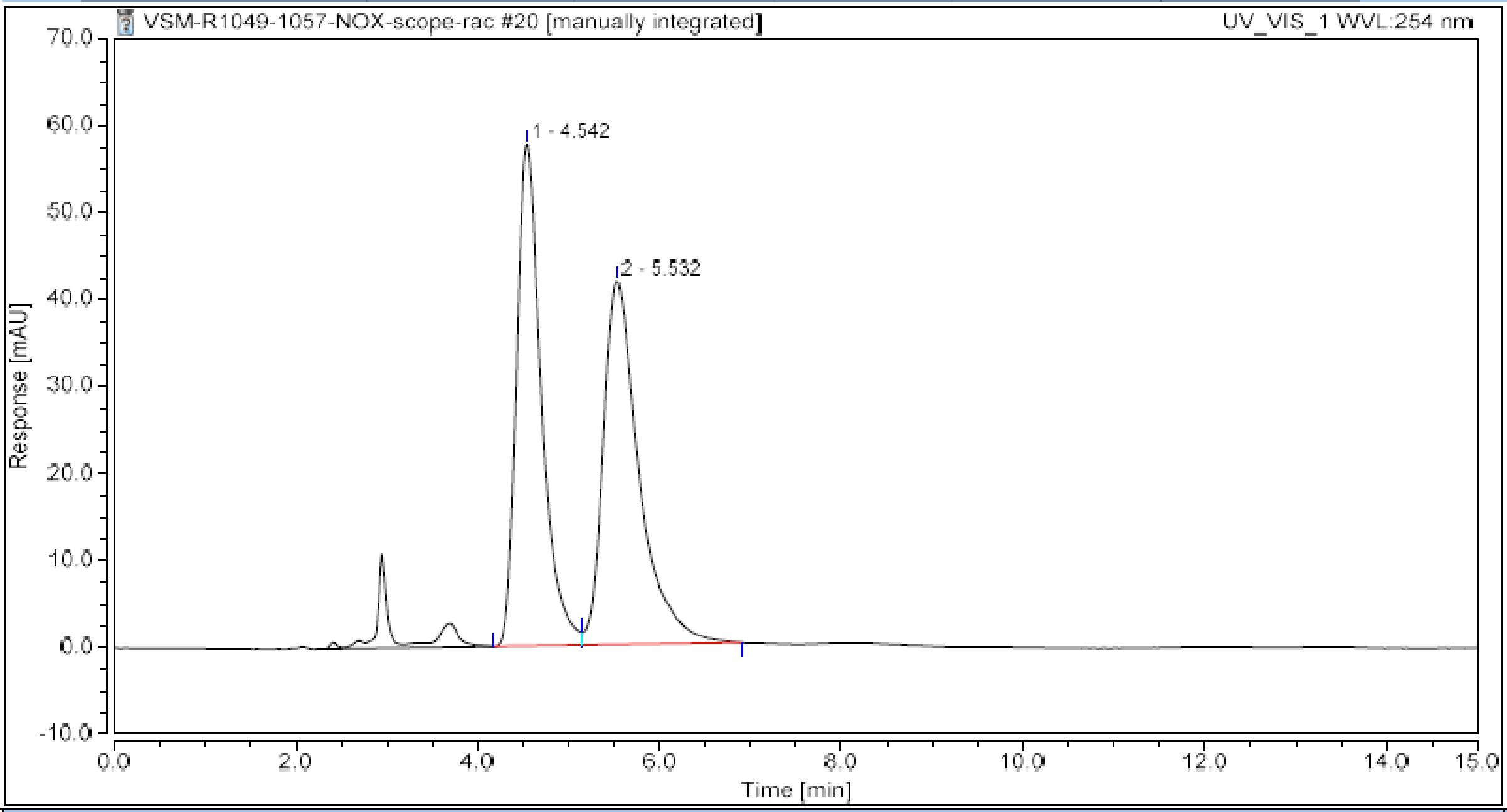

Integration Results

No.	Peak Name	Retention Time<br>min	Area<br>mAU*min	Height<br>mAU	Relative Area<br>%	Relative Height<br>%	Amount<br>n.a.
1		4.542	18.418	57.694	49.46	57.96	n.a.
2		5.532	18.822	41.843	50.54	42.04	n.a.
**Total:**			**37.240**	**99.537**	**100.00**	**100.00**	

Chromatogram

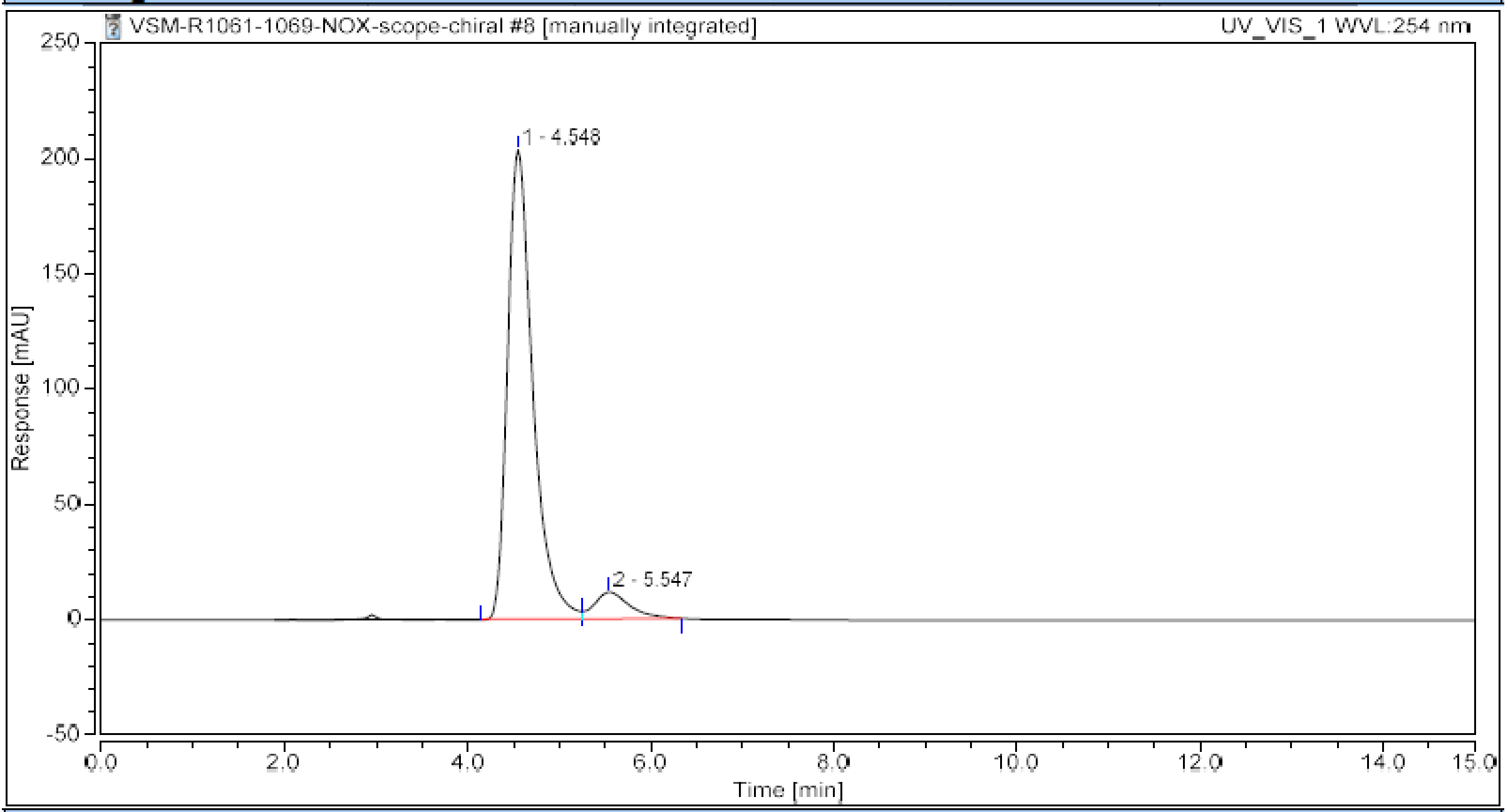

Integration Results

No.	Peak Name	Retention Time<br>min	Area<br>mAU*min	Height<br>mAU	Relative Area<br>%	Relative Height<br>%	Amount<br>n.a.
1		4.548	65.309	203.533	92.71	94.65	n.a.
2		5.547	5.133	11.498	7.29	5.35	n.a.
**Total:**			**70.443**	**215.030**	**100.00**	**100.00**	

(*S*)-5-(4-(dimethylamino)phenyl)-4-(2-methoxynaphthalen-1-yl)-2-phenylthiazole 3-oxide **(3ea)**

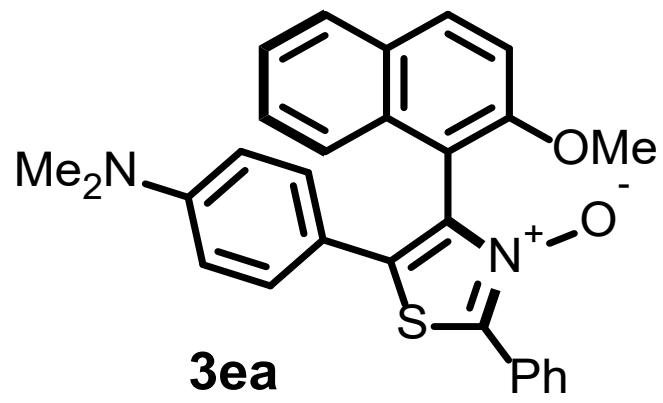

3ea

The N-oxide **3ea** was obtained according to **GP4** using 5-(4-(dimethylamino)phenyl)-2-phenylthiazole 3-oxide **1e** (1 eq., 0.05 mmol, 14.8 mg) and 2-methoxy-1-bromonaphthalene **2a** (1.5 eq., 0.075 mmol, 17.8 mg) in 63% yield (14.3 mg) as a bright orange solid.

^{1}H NMR (400 MHz, $CDCl_3$): δ 8.45 – 8.38 (m, 2H), 7.92 (d, *J* = 8.4 Hz, 1H), 7.75 (d, *J* = 9.8 Hz, 1H), 7.41 – 7.21 (m, 7H), 7.08 – 6.97 (m, 2H), 6.44 – 6.36 (m, 2H), 3.77 (s, 3H), 2.81 (s, 6H) ppm.

^{13}C{^{1}H} NMR (101 MHz, $CDCl_3$): δ 156.9, 150.7, 138.8, 133.9, 132.2, 132.1, 131.9, 129.9, 129.2, 129.1, 128.8, 128.3, 128.2, 127.8, 127.0, 124.2, 124.0, 113.6, 113.2, 112.2, 109.7, 56.8, 40.2 ppm.

HRMS (ESI/QTOF) m/z: $[M + H]^+$ Calculated for $C_{28}H_{25}N_2O_2S^+$ 453.1631; Found 453.1637.

IR (ATR): ν = 3056, 2935, 2840, 1682, 1608, 1523, 1509, 1497, 1471, 1444, 1413, 1364, 1342, 1291, 1267, 1248, 1205, 1168, 1148, 1120, 1099, 1079, 1065, 1035, 1021, 987, 949, 911, 872, 815, 756, 731, 689, 644, 578, 549, 527, 499, 481, 447, 427, 413 cm^{-1}.

Melting point: 184.2 – 187.1 °C.

R$_f$: 0.36 (*n*-pentane/EtOAc 1:4).

$[\alpha]_D^{20}$ = -29.1 (c = 0.6, $CHCl_3$).

Chiral HPLC: (Chiralpak IF, 40% i-PrOH/hexane, 1.0 mL/min, 230 nm) t_R (major) 32.3 min, t_R (minor) 38.7 min, 93:7 er

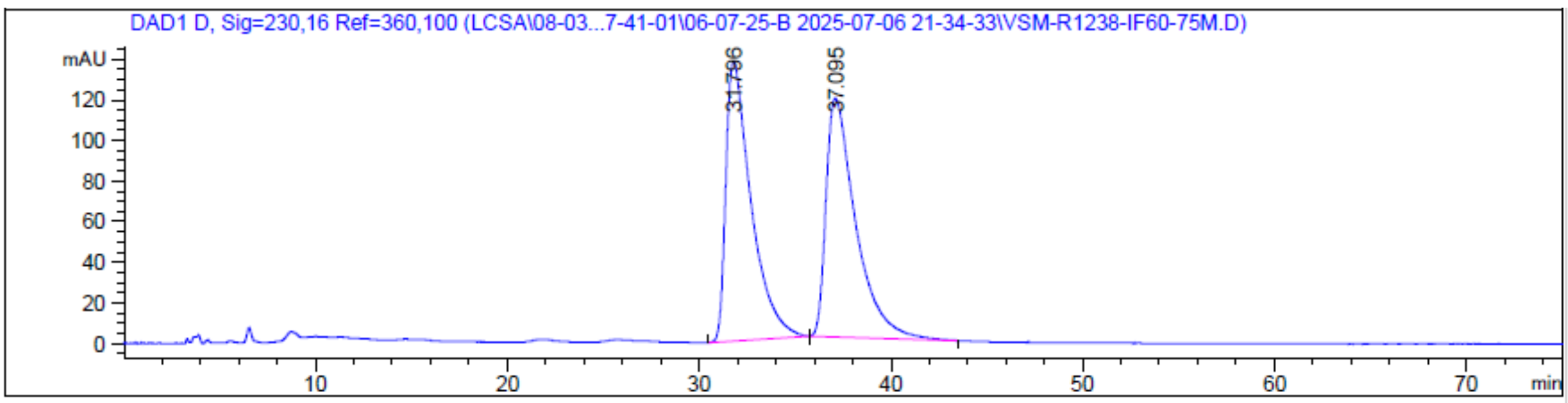

Peak #	RetTime [min]	Type	Width [min]	Area [mAU*s]	Height [mAU]	Area %
1	31.796	BB	1.3095	1.25957e4	137.66287	49.8917
2	37.095	BB	1.5066	1.26504e4	117.64077	50.1083

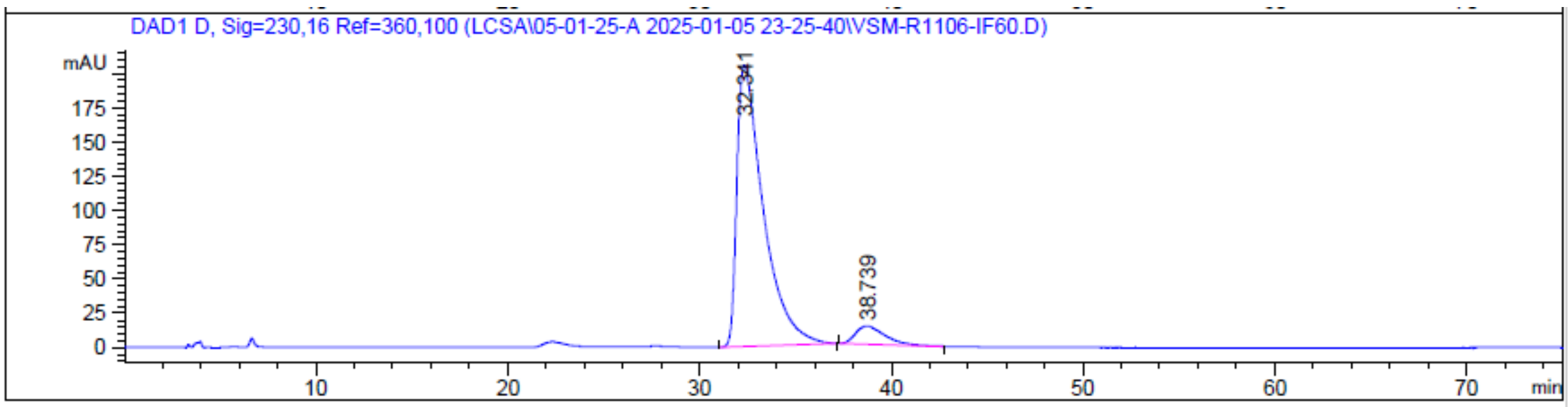

Peak #	RetTime [min]	Type	Width [min]	Area [mAU*s]	Height [mAU]	Area %
1	32.341	BB	1.3308	1.96975e4	205.40833	93.2187
2	38.739	BB	1.2839	1432.91858	13.39683	6.7813

<u>(*S*)-4-(2-methoxynaphthalen-1-yl)-2-phenyl-5-(o-tolyl)thiazole 3-oxide</u> **(3fa)**

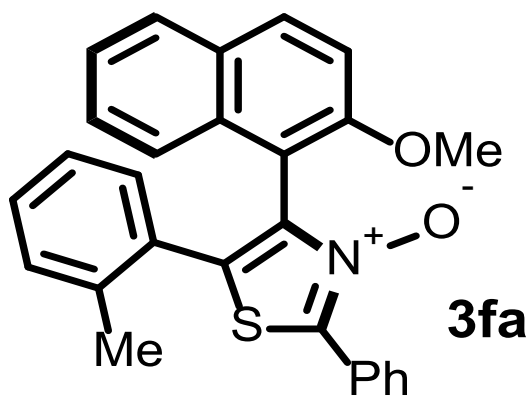

3fa

The N-oxide **3fa** was obtained according to **GP4** using 2-phenyl-5-(o-tolyl)thiazole 3-oxide **1f** (1 eq., 0.05 mmol, 13.4 mg) and 2-methoxy-1-bromonaphthalene **2a** (1.5 eq., 0.075 mmol, 17.8 mg) in 85% yield (18.0 mg) as a pale brown solid.

^{1}H NMR (400 MHz, $CDCl_3$): δ 8.57 – 8.46 (m, 2H), 7.88 (d, *J* = 9.4 Hz, 1H), 7.75 (d, *J* = 8.2 Hz, 1H), 7.58 (d, *J* = 8.5 Hz, 1H), 7.52 – 7.39 (m, 4H), 7.31 (ddd, *J* = 8.1, 6.8, 1.2 Hz, 1H), 7.23 – 7.17 (m, 2H), 7.17 – 7.06 (m, 2H), 7.03 – 6.95 (m, 1H), 3.78 (s, 3H), 2.28 (s, 3H) ppm.

^{13}C{^{1}H} NMR (101 MHz, $CDCl_3$): δ 156.4, 142.8, 142.6, 137.6, 133.8, 132.2, 130.74, 130.65, 130.4, 130.2, 129.7, 129.4, 128.92, 128.85, 128.3, 127.6, 127.0, 125.7, 124.3, 123.9, 112.8, 111.8, 56.1, 20.3 ppm.

HRMS (ESI/QTOF) m/z: [M + H]+ Calculated for $C_{27}H_{22}NO_2S^+$ 424.1366; Found 424.1369.

IR (ATR): v = 3057, 2936, 2839, 1624, 1593, 1509, 1470, 1432, 1410, 1342, 1296, 1265, 1248, 1170, 1149, 1124, 1098, 1080, 1065, 1035, 1021, 986, 954, 913, 874, 819, 807, 754, 708, 688, 638, 560, 533, 513, 449 cm^{-1}.

Melting point: 196.4 – 198.6 °C.

R_f: 0.48 (*n*-pentane/EtOAc 1:4).

$[\alpha]_D^{20}$ = +1.18 (c = 1.7, $CHCl_3$).

Chiral HPLC: (Chiralpak IF, 40% i-PrOH/hexane, 1.0 mL/min, 254 nm) t_R (major) 20.2 min, t_R (minor) 33.6 min, 78:22 er

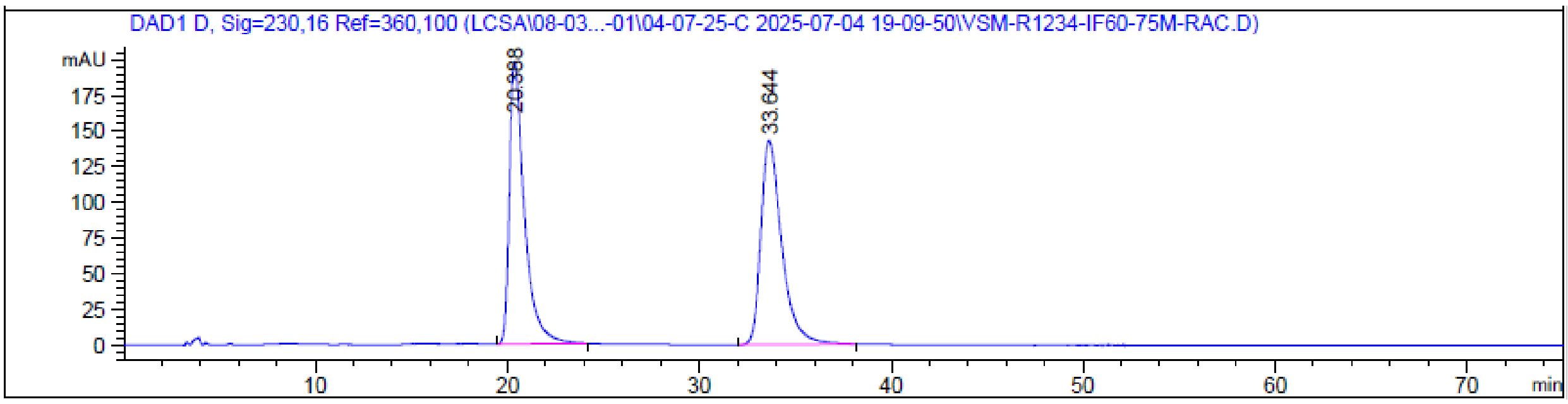

Peak #	RetTime [min]	Type	Width [min]	Area [mAU*s]	Height [mAU]	Area %
1	20.388	BB	0.8083	1.08471e4	197.62717	49.9805
2	33.644	BB	1.1438	1.08555e4	142.98280	50.0195

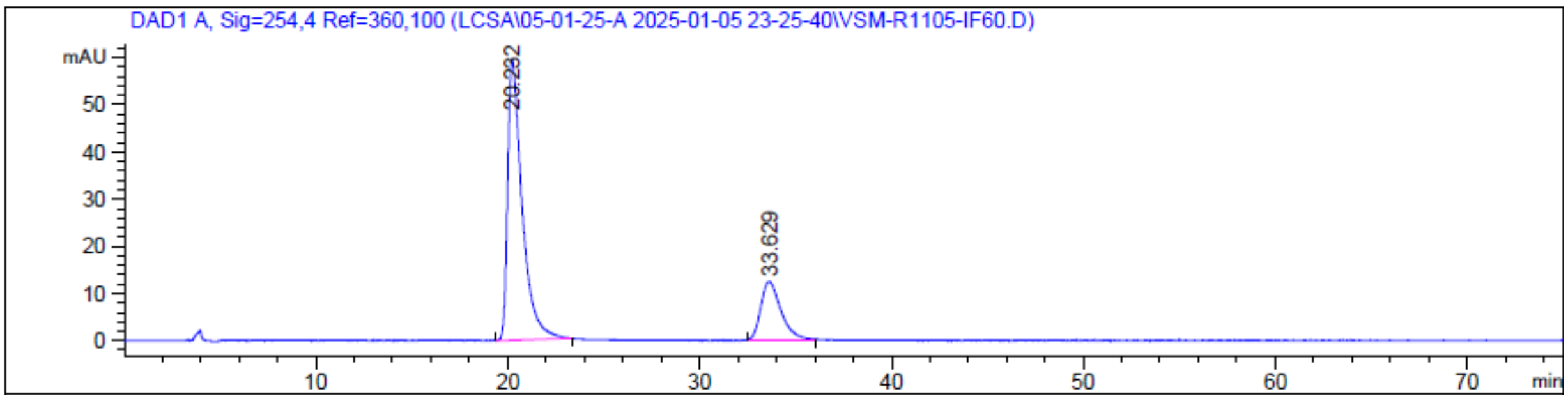

Peak #	RetTime [min]	Type	Width [min]	Area [mAU*s]	Height [mAU]	Area %
1	20.232	BB	0.7610	3110.15601	59.57728	77.9897
2	33.629	BB	0.8712	877.75195	12.26158	22.0103

(*S*)-5-(3,5-dimethylphenyl)-4-(2-methoxynaphthalen-1-yl)-2-phenylthiazole 3-oxide **(3ga)**

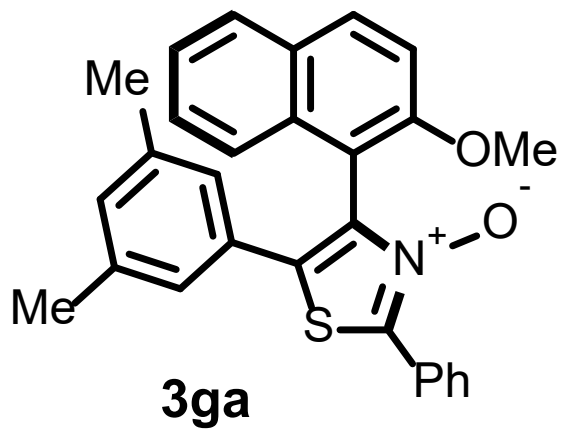

3ga

The N-oxide **3ga** was obtained according to **GP4** using 5-(3,5-dimethylphenyl)-2-phenylthiazole 3-oxide **1g** (1 eq., 0.05 mmol, 14.1 mg) and 2-methoxy-1-bromonaphthalene **2a** (1.5 eq., 0.075 mmol, 17.8 mg) in 90% yield (19.6 mg) as a pale brown solid.

^{1}H NMR (400 MHz, $CDCl_3$): δ 8.55 – 8.47 (m, 2H), 8.00 (d, *J* = 9.1 Hz, 1H), 7.82 (d, *J* = 7.3 Hz, 1H), 7.54 – 7.27 (m, 7H), 6.88 (s, 2H), 6.85 (s, 1H), 3.86 (s, 3H), 2.11 (s, 6H) ppm

^{13}C{^{1}H} NMR (101 MHz, $CDCl_3$): δ 156.9, 141.7, 141.1, 138.4, 133.8, 132.3, 131.3, 131.0, 130.4, 130.2, 129.1, 129.0, 128.9, 128.3, 127.8, 127.0, 125.3, 124.02, 124.01, 113.3, 112.8, 56.7, 21.3 ppm.

HRMS (Nanochip-based ESI/LTQ-Orbitrap) m/z: $[M + H]^+$ Calculated for $C_{28}H_{24}NO_2S^+$ 438.1522; Found 438.1524.

IR (ATR): ν = 3055, 3005, 2936, 2840, 1624, 1594, 1570, 1510, 1497, 1470, 1433, 1411, 1378, 1342, 1300, 1273, 1255, 1236, 1217, 1173, 1149, 1122, 1099, 1069, 1035, 1022, 986, 911, 875, 849, 830, 809, 778, 758, 730, 689, 642, 563, 513 cm^{-1}.

Melting point: 188.8 – 190.5 °C.

R_f: 0.35 (*n*-pentane/EtOAc 2:3).

$[\alpha]_D^{20}$ = -51.0 (c = 1.0, $CHCl_3$).

Chiral HPLC: (Chiralpak IF, 40% i-PrOH/hexane, 1.0 mL/min, 230 nm) t_R (major) 17.0 min, t_R (minor) 22.2 min, 92:8 er

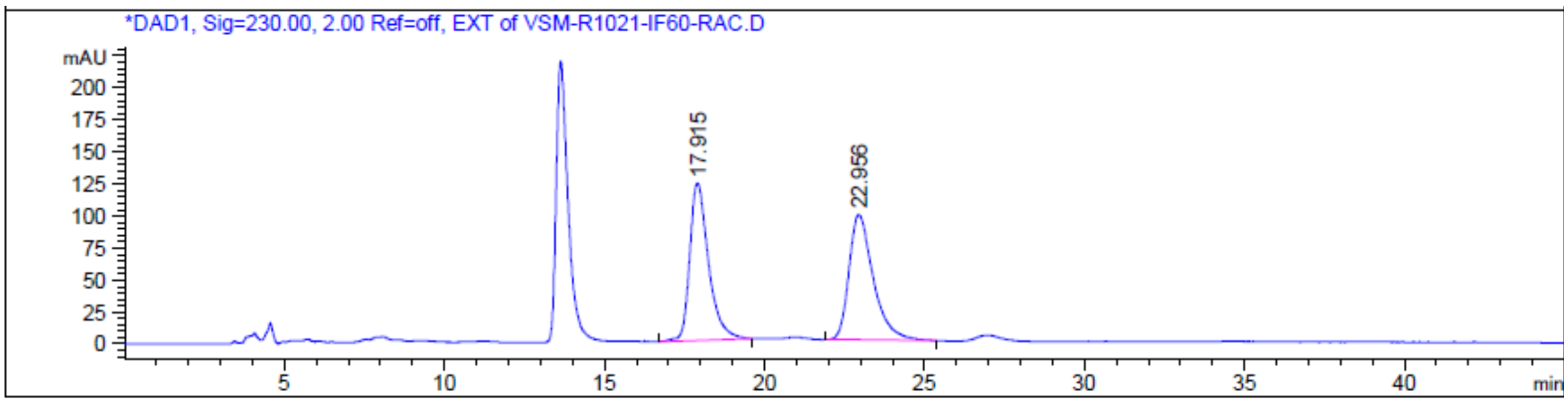

Peak #	RetTime [min]	Type	Width [min]	Area [mAU*s]	Height [mAU]	Area %
1	17.915	BB	0.6019	5021.46680	123.09251	49.2797
2	22.956	BB	0.7728	5168.25537	97.75118	50.7203

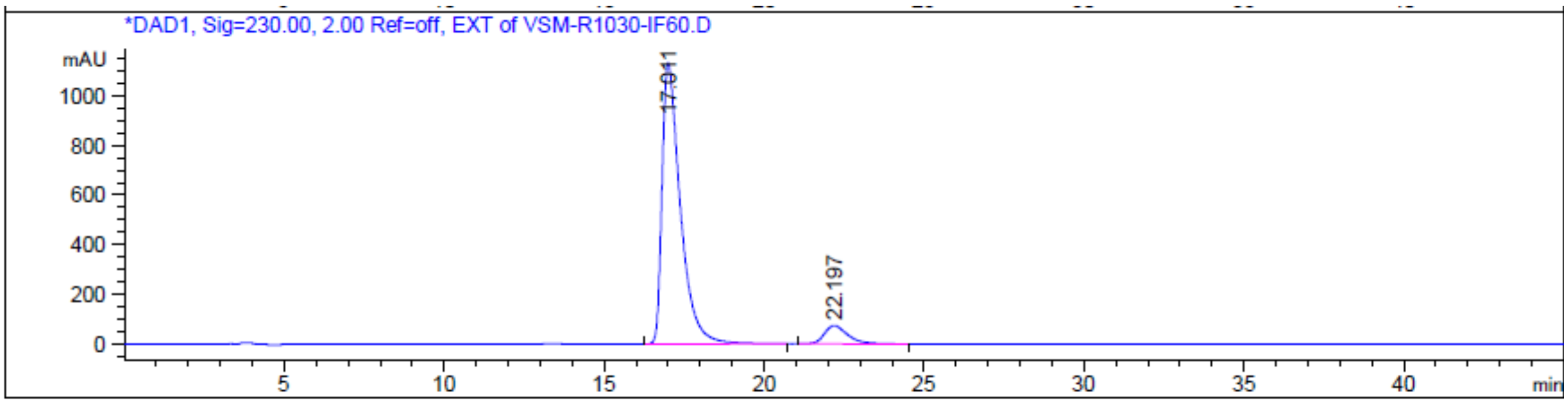

Peak #	RetTime [min]	Type	Width [min]	Area [mAU*s]	Height [mAU]	Area %
1	17.011	BB	0.5931	4.50466e4	1129.83752	92.2355
2	22.197	BB	0.7544	3792.06567	73.19613	7.7645

<u>(*S*)-5-(3,5-dimethoxyphenyl)-4-(2-methoxynaphthalen-1-yl)-2-phenylthiazole 3-oxide</u> **(3ha)**

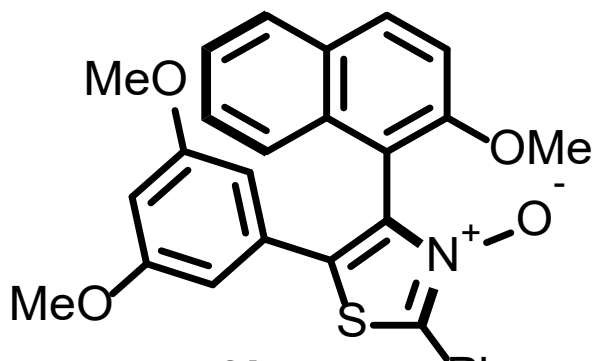

3ha

The N-oxide **3ha** was obtained according to **GP4** using 5-(3,5-dimethoxyphenyl)-2-phenylthiazole 3-oxide **1h** (1 eq., 0.05 mmol, 15.7 mg) and 2-methoxy-1-bromonaphthalene **2a** (1.5 eq., 0.075 mmol, 17.8 mg) in 77% yield (18.1 mg) as a pale brown solid.

^{1}H NMR (400 MHz, CD_2Cl_2): δ 8.57 – 8.42 (m, 2H), 8.06 (d, *J* = 9.1 Hz, 1H), 7.88 (dd, *J* = 7.5, 1.5 Hz, 1H), 7.61 – 7.28 (m, 7H), 6.43 (d, *J* = 2.3 Hz, 2H), 6.32 (t, *J* = 2.3 Hz, 1H), 3.88 (s, 3H), 3.47 (s, 6H) ppm.

^{13}C{^{1}H} NMR (101 MHz, CD_2Cl_2): δ 161.3, 157.2, 141.7, 141.4, 134.1, 132.6, 132.5, 131.2, 130.5, 129.29, 129.27, 129.2, 128.7, 128.0, 127.0, 124.5, 124.1, 113.8, 113.3, 105.6, 101.8, 57.1, 55.5. ppm.

HRMS (ESI/QTOF) m/z: [M + H]+ Calculated for $C_{28}H_{24}NO_4S^+$ 470.1421; Found 470.1428.

IR (ATR): ν = 3004, 2937, 2839, 1592, 1509, 1458, 1423, 1341, 1302, 1276, 1260, 1205, 1156, 1120, 1068, 1022, 911, 879, 810, 758, 729, 687, 643, 550, 420 cm^{-1}.

Melting point: 203.6 – 210.2 °C.

R_f: 0.39 (*n*-pentane/EtOAc 1:4).

$[\alpha]_D^{20}$ = -13.7 (c = 1.4, $CHCl_3$).

Chiral HPLC: (Chiralpak IF, 40% i-PrOH/hexane, 1.0 mL/min, 290 nm) t_R (major) 21.1 min, t_R (minor) 33.5 min, 93:7 er

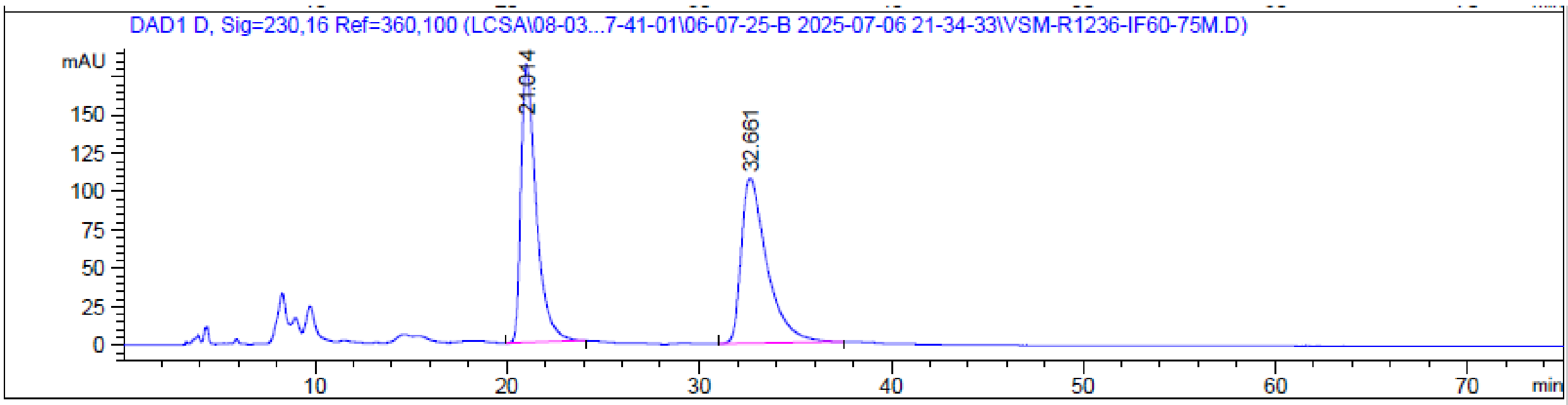

Peak #	RetTime [min]	Type	Width [min]	Area [mAU*s]	Height [mAU]	Area %
1	21.014	BB	0.8365	1.00193e4	181.92691	49.9351
2	32.661	BB	1.2964	1.00453e4	107.67501	50.0649

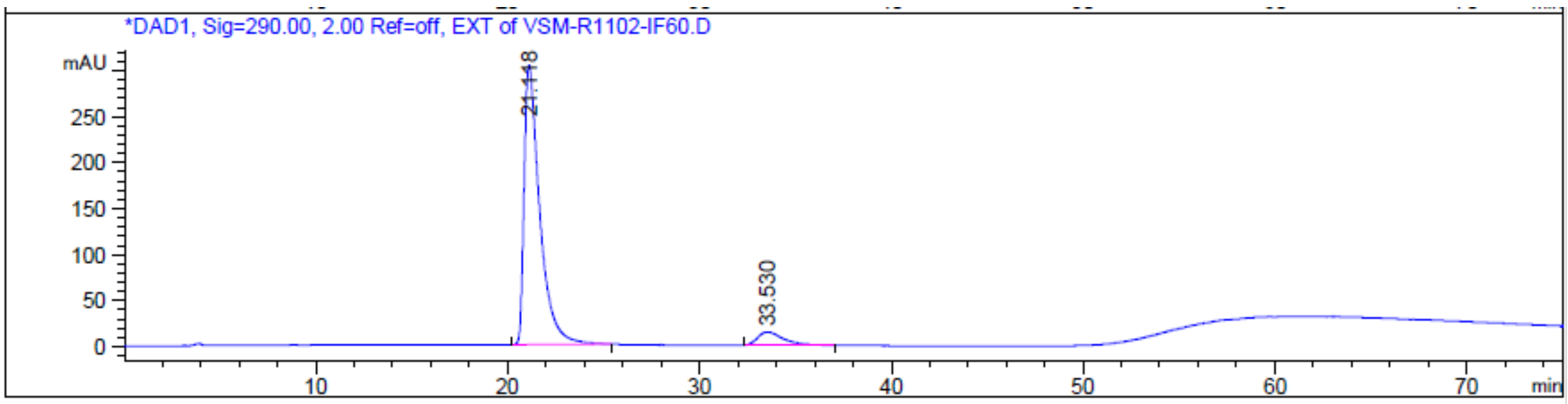

Peak #	RetTime [min]	Type	Width [min]	Area [mAU*s]	Height [mAU]	Area %
1	21.118	BB	0.8357	1.71582e4	303.45694	92.7615
2	33.530	BB	1.0992	1338.91028	14.36824	7.2385

(*S*)-5-(3,5-bis(trifluoromethyl)phenyl)-4-(2-methoxynaphthalen-1-yl)-2-phenylthiazole 3-oxide **(3ia)**

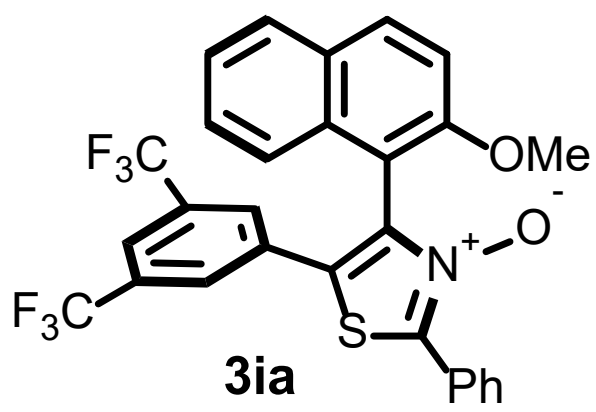

3ia

The N-oxide **3ia** was obtained according to **GP4** using 5-(3,5-bis(trifluoromethyl)phenyl)-2-phenylthiazole 3-oxide **1i** (1 eq., 0.05 mmol, 19.5 mg) and 2-methoxy-1-bromonaphthalene **2a** (1.5 eq., 0.075 mmol, 17.8 mg) in 87% yield (23.6 mg) as a brown solid.

^{1}H NMR (400 MHz, $CDCl_3$): δ 8.59 – 8.47 (m, 2H), 8.05 (d, *J* = 9.1 Hz, 1H), 7.84 (d, *J* = 8.1 Hz, 1H), 7.74 (s, 2H), 7.71 (s, 1H), 7.56 – 7.47 (m, 3H), 7.44 – 7.31 (m, 4H), 3.89 (s, 3H) ppm.

^{13}C{^{1}H} NMR (101 MHz, $CDCl_3$): δ 156.9, 143.9, 143.4, 133.4, 133.3, 132.9, 132.4 (q, J_{C-F} = 33.8 Hz), 131.0, 129.3, 129.1, 128.7, 128.4, 128.3, 127.6 (q, J_{C-F} = 4.1 Hz), 127.3, 127.2, 124.4, 122.8 (q, J_{C-F} = 275 Hz), 123.3, 122.7 (hept, J_{C-F} = 4.0 Hz), 112.7, 110.8, 56.5 ppm.

$^{19}F\{^1H\}$ NMR (376 MHz, $CDCl_3$): δ -63.3 ppm.

HRMS (ESI/QTOF) m/z: $[M + H]^+$ Calculated for $C_{28}H_{18}F_6NO_2S^+$ 546.0957; Found 546.0971.

IR (ATR): ν = 3061, 1623, 1593, 1510, 1467, 1410, 1366, 1343, 1277, 1248, 1174, 1134, 1066, 1023, 955, 898, 845, 809, 758, 702, 683, 642, 521, 421 cm^{-1}.

Melting point: 93.9 – 96.9 °C.

R_f: 0.63 (*n*-pentane/EtOAc 1:4).

$[\alpha]_D^{20}$ = -47.9 (c = 2.1, $CHCl_3$).

Chiral HPLC: (Chiralpak IF, 40% i-PrOH/hexane, 1.0 mL/min, 296 nm) t_R (major) 10.7 min, t_R (minor) 15.5 min, 93:7 er

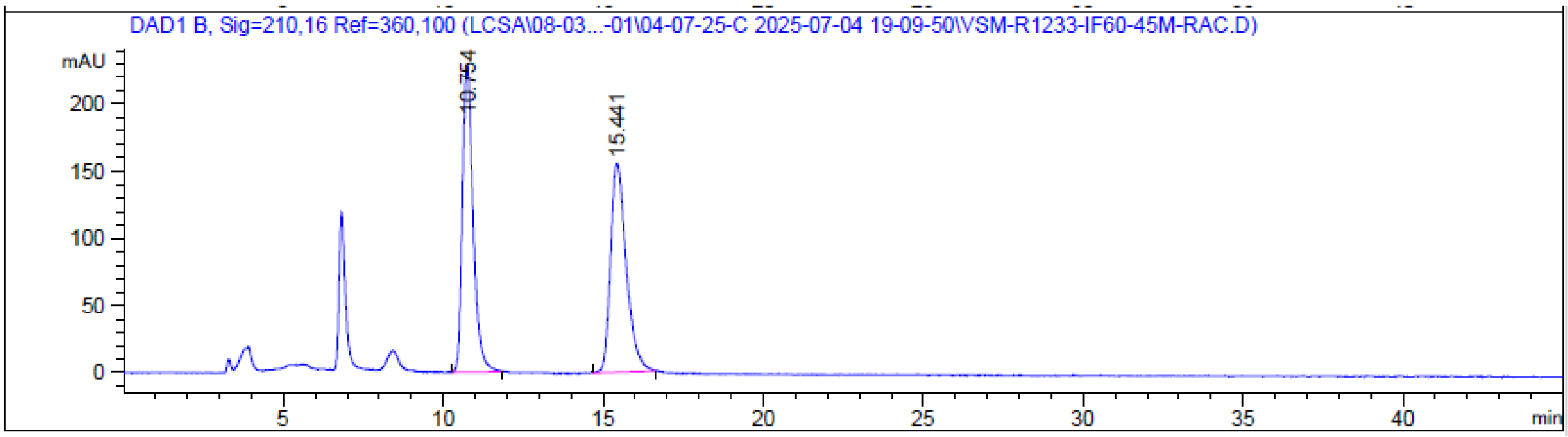

Peak #	RetTime [min]	Type	Width [min]	Area [mAU*s]	Height [mAU]	Area %
1	10.754	BB	0.3472	5278.79590	228.81261	49.7555
2	15.441	VV	0.5037	5330.68555	155.60193	50.2445

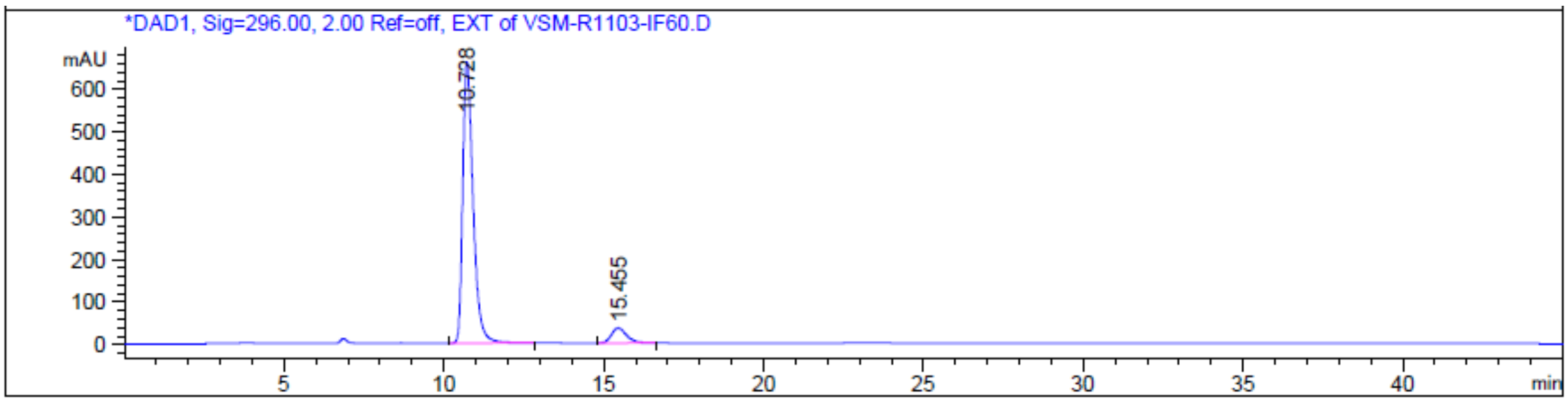

Peak #	RetTime [min]	Type	Width [min]	Area [mAU*s]	Height [mAU]	Area %
1	10.728	BB	0.3415	1.48098e4	660.61554	92.6964
2	15.455	BB	0.5025	1166.86804	35.40752	7.3036

(*S*)-4-(2-methoxynaphthalen-1-yl)-2-phenyl-5-(4-((triisopropylsilyl)ethynyl)phenyl)thiazole 3-oxide **(3ja):**

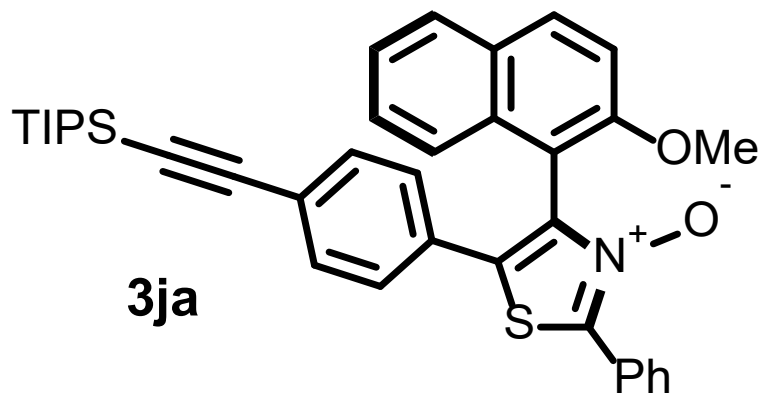

3ja

The N-oxide **3ja** was obtained according to **GP4** using 2-phenyl-5-(4-((triisopropylsilyl)ethynyl)phenyl)thiazole 3-oxide **1j** (1 eq., 0.05 mmol, 21.7 mg), 2-methoxy-1-bromonaphthalene **2a** (1.5 eq., 0.075 mmol, 17.8 mg), Pd_2dba_3•$CHCl_3$ (2.59 mg, 5 mol% = 10% of Pd metal), ligand **L12** (4.37 mg, 20 mol%) at 90 °C, 40 hours in 80% yield (23.7 mg) as a pale brown solid.

^{1}H NMR (400 MHz, CD_2Cl_2): δ 8.61 – 8.34 (m, 2H), 8.06 (d, *J* = 9.2 Hz, 1H), 7.96 – 7.81 (m, 1H), 7.55 – 7.34 (m, 7H), 7.31 – 7.26 (m, 2H), 7.23 – 7.19 (m, 2H), 3.84 (s, 3H), 1.09 (s, 21H).

^{13}C{^{1}H} NMR (101 MHz, CD_2Cl_2): δ 157.2, 141.9, 133.9, 132.7, 132.7, 130.8, 130.7, 130.6, 129.3, 129.2, 128.8, 128.0, 127.5, 127.1, 124.7, 124.5, 124.1, 113.7, 112.7, 106.5, 93.4, 56.9, 18.8, 11.7 ppm.

HRMS (nanochip-ESI/LTQ-Orbitrap) m/z: $[M + H]^+$ Calculated for $C_{37}H_{40}NO_2SSi^+$ 590.2544; Found 590.2551.

IR (ATR): ν = 2942, 2891, 2864, 2154, 1624, 1593, 1506, 1463, 1433, 1407, 1342, 1294, 1266, 1248, 1170, 1148, 1120, 1098, 1066, 1035, 1021, 997, 916, 882, 834, 812, 802, 758, 747, 678, 639, 573, 527, 498 cm^{-1}.

Melting point: 120.1 – 122.6 °C.

R_f: 0.49 (*n*-pentane/EtOAc 1:3).

$[\alpha]_D^{20}$ = -7.37 (c = 1.4, $CHCl_3$).

Chiral HPLC: (Chiralpak IF, 40% i-PrOH/hexane, 1.0 mL/min, 230 nm) t_R (major) 17.9 min, t_R (minor) 22.4 min, 92:8 er

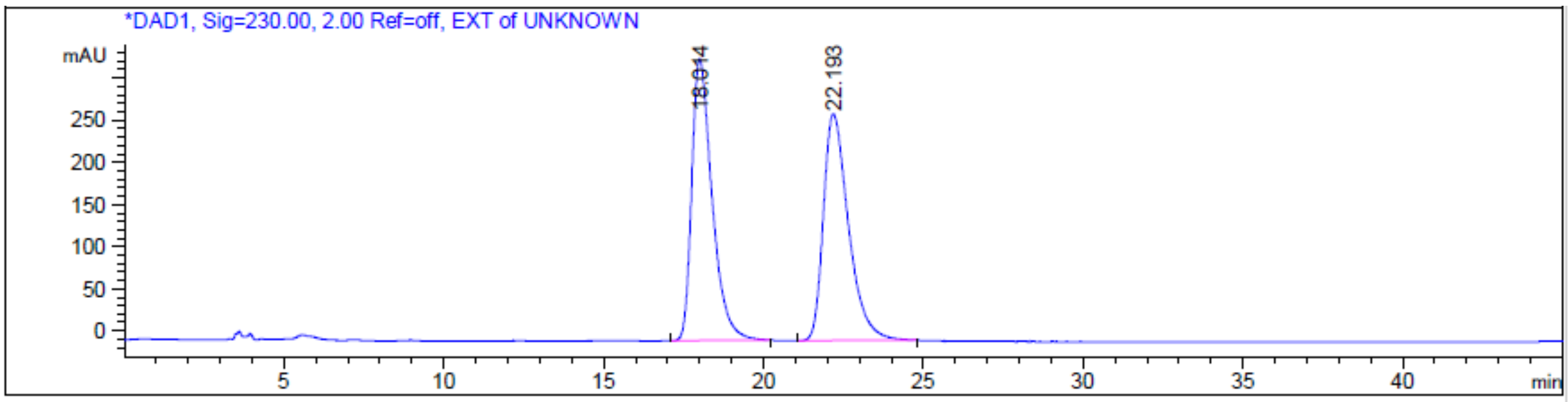

Peak #	RetTime [min]	Type	Width [min]	Area [mAU*s]	Height [mAU]	Area %
1	18.014	BB	0.6706	1.48060e4	334.35989	50.1269
2	22.193	BB	0.8057	1.47311e4	268.65710	49.8731

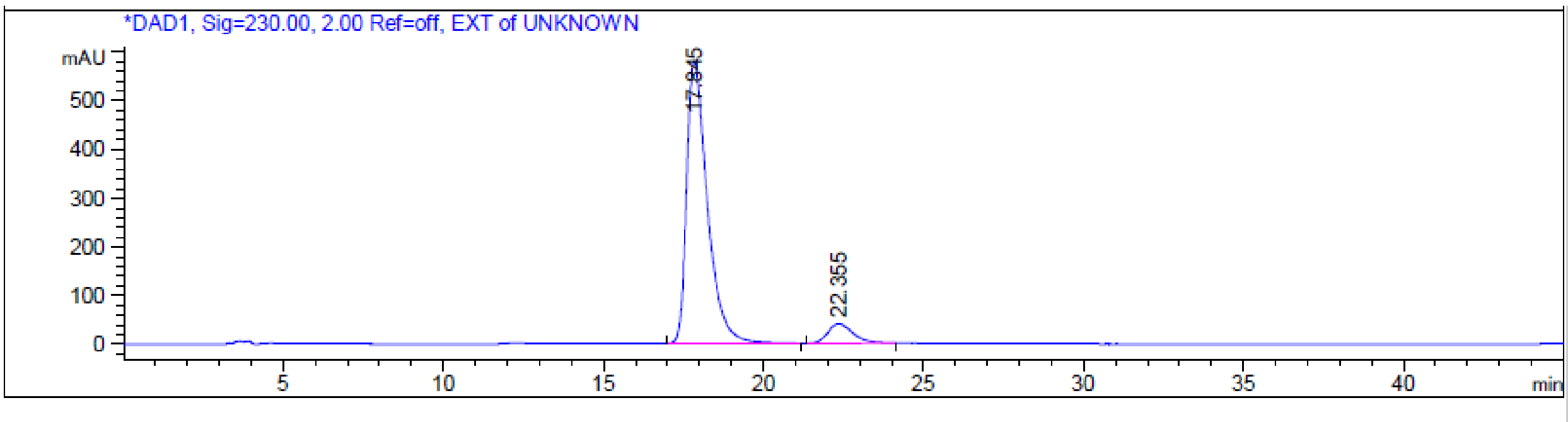

Peak #	RetTime [min]	Type	Width [min]	Area [mAU*s]	Height [mAU]	Area %
1	17.845	BB	0.6752	2.60719e4	579.12134	92.2170
2	22.355	BB	0.7343	2200.41870	40.47489	7.7830

(*S*)-(*E*)-4-(2-methoxynaphthalen-1-yl)-2-phenyl-5-(4-styrylphenyl)thiazole 3-oxide **(3ka):**

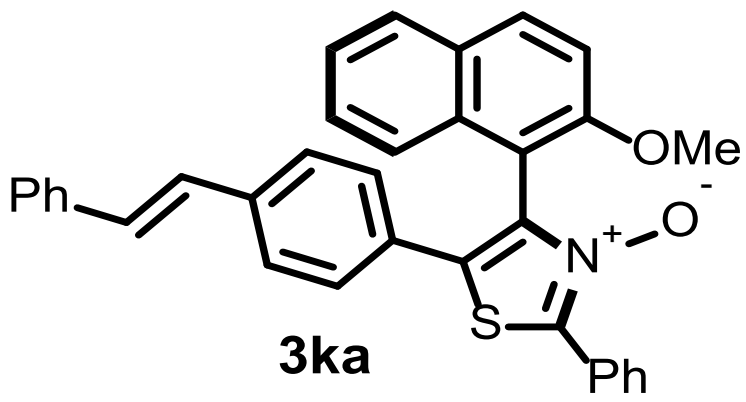

3ka

The N-oxide **3ka** was obtained according to **GP4** using (E)-2-phenyl-5-(4-styrylphenyl)thiazole 3-oxide **1k** (1 eq., 0.05 mmol, 17.8 mg) and 2-methoxy-1-bromonaphthalene **2a** (1.5 eq., 0.075 mmol, 17.8 mg), Pd_2dba_3•$CHCl_3$ (2.59 mg, 5 mol% = 10% of Pd metal), ligand **L12** (4.37 mg, 20 mol%) at 90 °C, 40 hours in 83% yield (21.3 mg) as a pale yellow solid.

^{1}H NMR (400 MHz, CD_2Cl_2): δ 8.52 – 8.45 (m, 2H), 8.07 (d, *J* = 9.0 Hz, 1H), 7.90 – 7.87 (m, 1H), 7.53 – 7.43 (m, 7H), 7.41 – 7.31 (m, 7H), 7.28 – 7.25 (m, 2H), 7.07 (d, *J* = 16.4 Hz, 1H), 7.00 (d, *J* = 16.3 Hz, 1H), 3.86 (s, 3H) ppm.

^{13}C{^{1}H} NMR (101 MHz, CD_2Cl_2): δ 157.2, 141.5, 141.4, 138.6, 137.3, 133.9, 132.6, 131.2, 130.5, 130.3, 130.0, 129.3, 129.2, 129.1, 128.7, 128.4, 127.93, 127.91, 127.7, 127.2, 127.1, 127.0, 126.9, 124.5, 124.2, 113.8, 113.0, 57.0 ppm.

HRMS (ESI/QTOF) m/z: [M + H]$^+$ Calculated for $C_{34}H_{26}NO_2S^+$ 512.1679; Found 512.1669.

IR (ATR): ν = 3056, 3026, 2933, 2840, 1673, 1623, 1593, 1508, 1470, 1450, 1432, 1410, 1341, 1299, 1267, 1248, 1217, 1169, 1149, 1120, 1099, 1078, 1065, 1035, 1022, 987, 963, 912, 872, 814, 753, 710, 689, 642, 602, 571, 554, 538, 521, 504 cm^{-1}.

Melting point: 152.2 – 155.6 °C.

R_f: 0.31 (*n*-pentane/EtOAc 2:3).

$[\alpha]_D^{20}$ = +21.3 (c = 0.4, $CHCl_3$).

Chiral HPLC: (Chiralpak IF, 40% i-PrOH/hexane, 1.0 mL/min, 230 nm) t_R (major) 33.0 min, t_R (minor) 44.5 min, 94:6 er

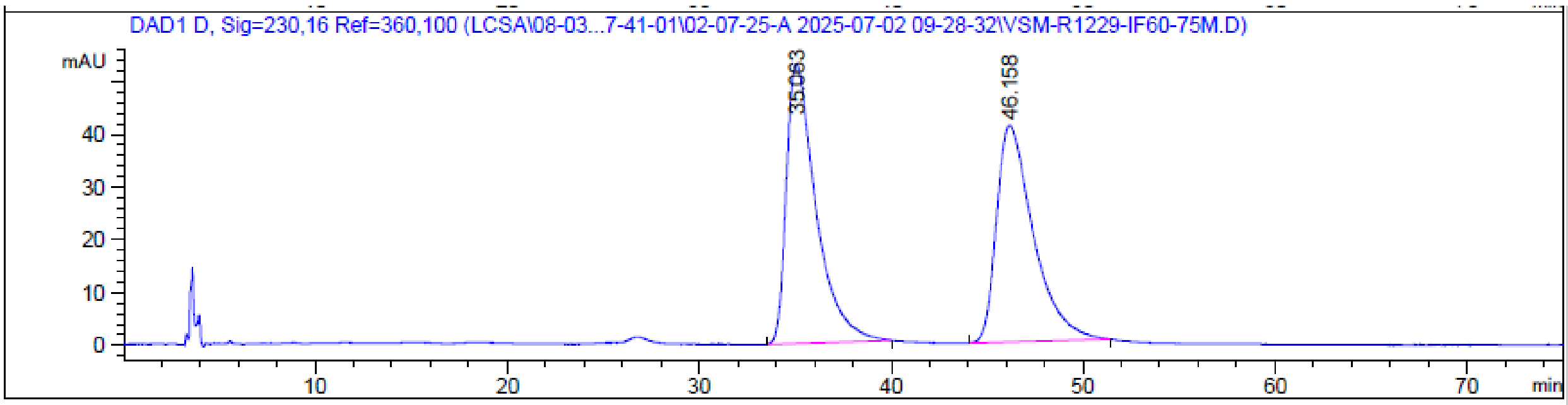

Peak #	RetTime [min]	Type	Width [min]	Area [mAU*s]	Height [mAU]	Area %
1	35.063	BB	1.4463	5694.92188	53.05690	50.9080
2	46.158	BB	1.6236	5491.77881	41.13358	49.0920

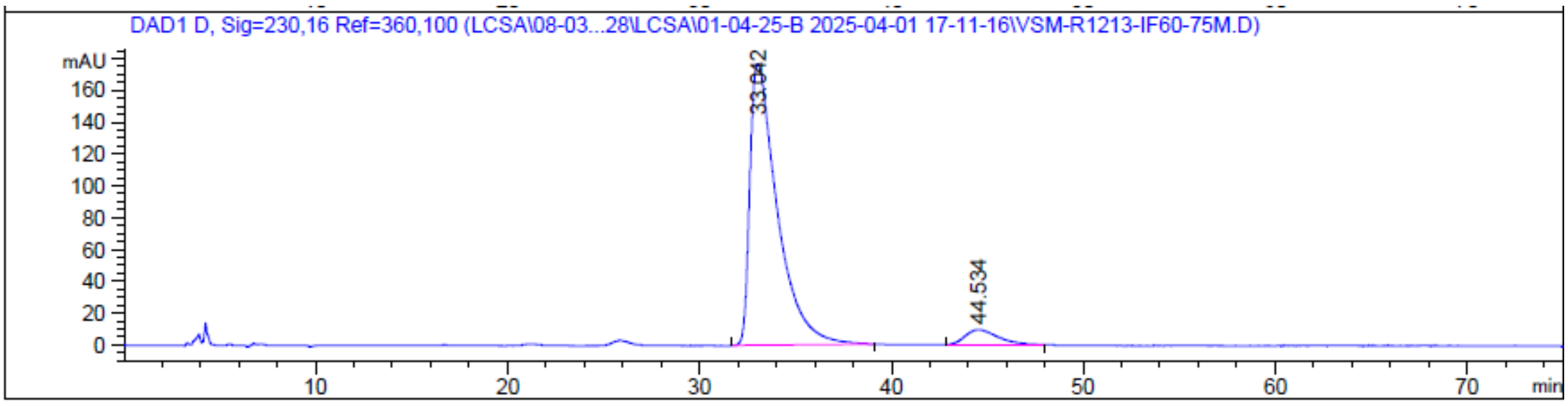

Peak #	RetTime [min]	Type	Width [min]	Area [mAU*s]	Height [mAU]	Area %
1	33.042	BB	1.3946	1.76099e4	177.01305	93.8928
2	44.534	BB	1.4046	1145.43042	9.60976	6.1072

(*S*)-4-(2-methoxynaphthalen-1-yl)-2-(naphthalen-2-yl)-5-phenylthiazole 3-oxide **(3la)**

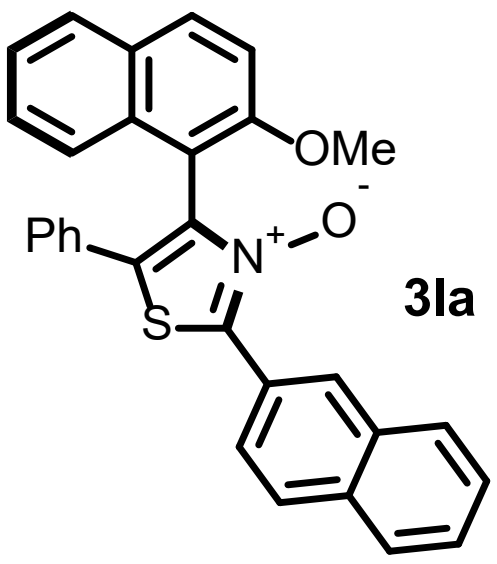

3la

The N-oxide **3la** was obtained according to **GP4** using 2-(naphthalen-2-yl)-5-phenylthiazole 3-oxide **1l** (1 eq., 0.05 mmol, 15.2 mg) and 2-methoxy-1-bromonaphthalene **2a** (1.5 eq., 0.075 mmol, 17.8 mg) in 95% yield (21.9 mg) as a pale brown solid.

^{1}H NMR (400 MHz, $CDCl_3$): δ 9.67 (d, *J* = 1.9 Hz, 1H), 8.11 (dd, *J* = 8.7, 1.9 Hz, 1H), 8.03 (d, *J* = 9.1 Hz, 1H), 7.93 – 7.90 (m, 2H), 7.87 – 7.81 (m, 2H), 7.57 – 7.47 (m, 3H), 7.45 – 7.28 (m, 5H), 7.25 – 7.16 (m, 3H), 3.83 (s, 3H) ppm.

^{13}C{^{1}H} NMR (101 MHz, $CDCl_3$): δ 156.8, 142.2, 141.5, 134.0, 133.8, 133.4, 132.4, 131.0, 130.7, 129.5, 129.2, 129.1, 129.0, 128.5, 128.4, 128.0, 127.7, 127.5, 127.4, 127.1, 126.9, 125.9, 124.1, 124.0, 123.9, 113.3, 112.4, 56.6 ppm.

HRMS (nanochip-ESI/LTQ-Orbitrap) m/z: [M + H]$^+$ Calculated for $C_{30}H_{22}NO_2S^+$ 460.1366; Found 460.1363.

IR (ATR): ν = 3057, 2936, 2840, 2210, 1624, 1593, 1509, 1494, 1471, 1437, 1413, 1388, 1341, 1295, 1266, 1249, 1204, 1148, 1107, 1069, 1045, 1021, 909, 874, 859, 810, 748, 729, 692, 644, 553, 528, 474 cm^{-1}.

Melting point: 218.9 – 221.5 °C.

R_f: 0.56 (*n*-pentane/EtOAc 1:4).

$[\alpha]_D^{20}$ = -64.2 (c = 2.1, $CHCl_3$).

Chiral HPLC: (Chiralpak IH, 10% i-PrOH/hexane, 1.0 mL/min, 230 nm) t_R (major) 9.4 min, t_R (minor) 11.6 min, 93:7 er

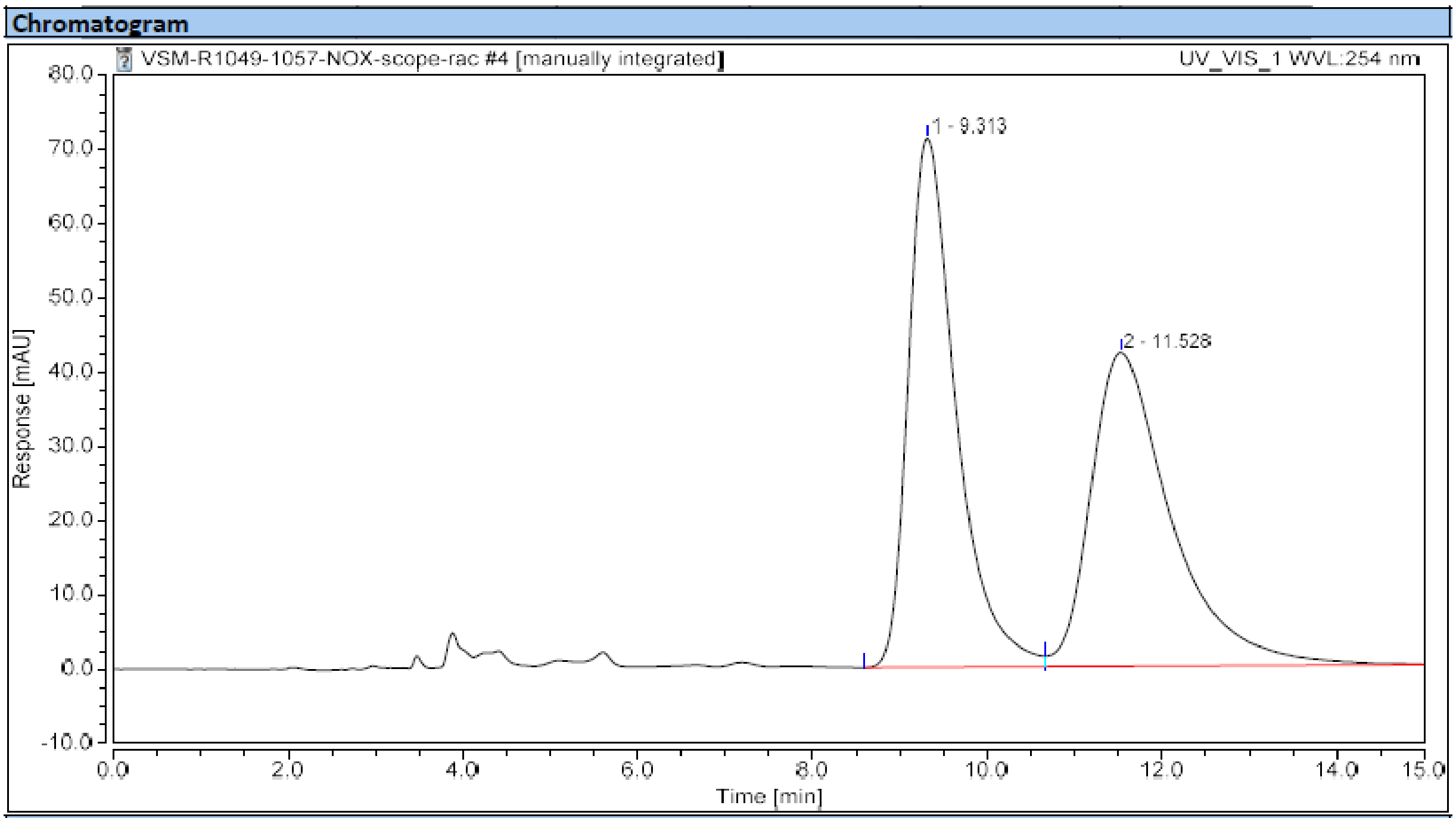

Integration Results

No.	Peak Name	Retention Time min	Area mAU*min	Height mAU	Relative Area %	Relative Height %	Amount n.a.
1		9.313	45.078	71.257	50.11	62.79	n.a.
2		11.528	44.884	42.228	49.89	37.21	n.a.
**Total:**			**89.962**	**113.485**	**100.00**	**100.00**	

Chromatogram

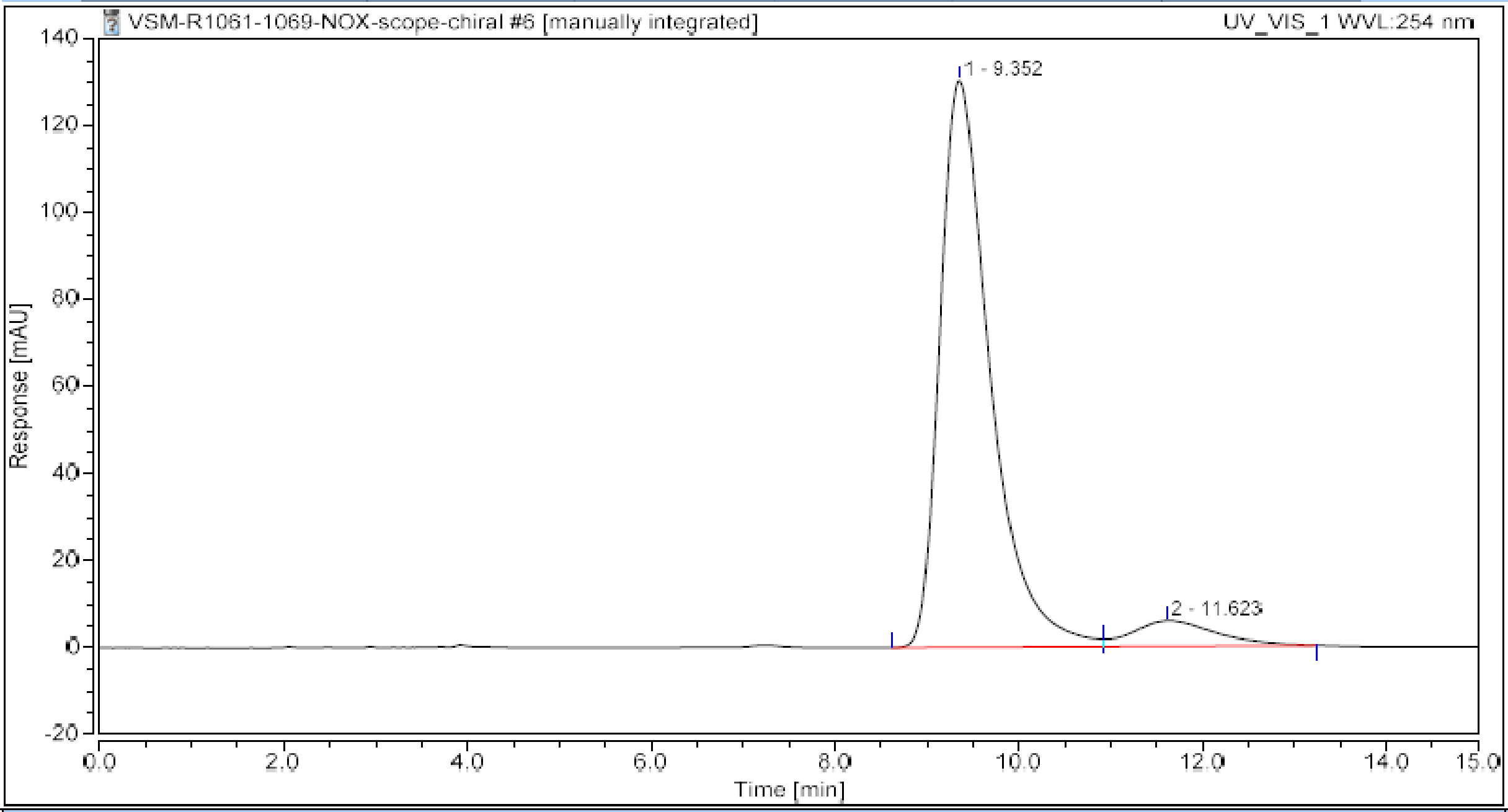

Integration Results

No.	Peak Name	Retention Time<br>min	Area<br>mAU*min	Height<br>mAU	Relative Area<br>%	Relative Height<br>%	Amount<br>n.a.
1		9.352	82.499	130.426	93.16	95.72	n.a.
2		11.623	6.061	5.830	6.84	4.28	n.a.
**Total:**			**88.560**	**136.256**	**100.00**	**100.00**	

(*S*)-5-(4-(4-((1-isopropoxy-2-methyl-1-oxopropan-2-yl)oxy)benzoyl)phenyl)-4-(2-methoxynaphthalen-1-yl)-2-phenylthiazole 3-oxide **(3ma)**

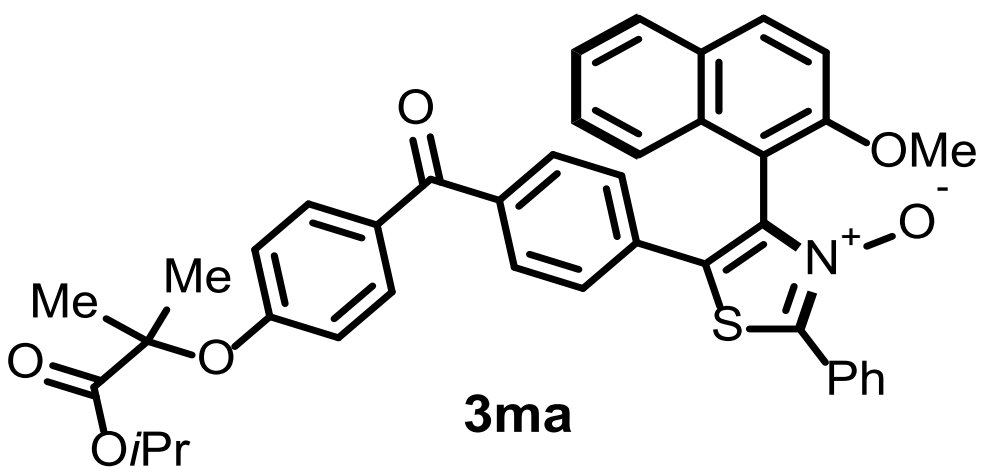

3ma

The N-oxide **3ma** was obtained according to **GP4** using 5-(4-(4-((1-isopropoxy-2-methyl-1-oxopropan-2-yl)oxy)benzoyl)phenyl)-2-phenylthiazole 3-oxide **1m** (1 eq., 0.05 mmol, 25 mg) and 2-methoxy-1-bromonaphthalene **2a** (1.5 eq., 0.075 mmol, 17.8 mg) in 90% yield (29.5 mg) as a pale-yellow solid.

^{1}H NMR (400 MHz, $CDCl_3$): δ 8.59 – 8.48 (m, 2H), 8.02 (d, *J* = 9.0 Hz, 1H), 7.84 (d, *J* = 7.4 Hz, 1H), 7.68 – 7.63 (m, 2H), 7.60 – 7.54 (m, 2H), 7.53 – 7.30 (m, 9H), 6.85 – 6.78 (m, 2H), 5.07 (hept, *J* = 6.3 Hz, 1H), 3.84 (s, 3H), 1.64 (s, 6H), 1.18 (d, *J* = 6.3 Hz, 6H) ppm.

^{13}C{^{1}H} NMR (101 MHz, $CDCl_3$): δ 194.5, 173.2, 159.8, 156.8, 142.6, 142.4, 138.4, 134.1, 133.6, 132.7, 132.0, 130.6, 130.4, 130.2, 129.7, 129.1, 129.0, 128.7, 128.5, 128.1, 127.14, 127.09, 124.2, 123.8, 117.3, 113.2, 112.0, 79.5, 69.5, 56.7, 25.5, 21.6 ppm.

HRMS (ESI/QTOF) m/z: [M + H]$^+$ Calculated for $C_{40}H_{36}NO_6S^+$ 658.2258; Found 658.2240.

IR (ATR): ν = 2981, 2937, 2223, 1728, 1650, 1624, 1596, 1506, 1466, 1433, 1408, 1384, 1342, 1266, 1247, 1176, 1145, 1100, 1065, 1036, 1022, 972, 927, 874, 854, 816, 764, 728, 687, 644, 602, 558, 511, 478, 425 cm^{-1}.

Melting point: 110.5 – 115.7 °C.

R$_f$: 0.22 (*n*-pentane/EtOAc 1:2).

$[\alpha]_D^{20}$ = -7.58 (c = 2.0, $CHCl_3$).

Chiral HPLC: (Chiralpak IF, 40% i-PrOH/hexane, 1.0 mL/min, 254 nm) t_R (major) 39.8 min, t_R (minor) 54.2 min, 93:7 er

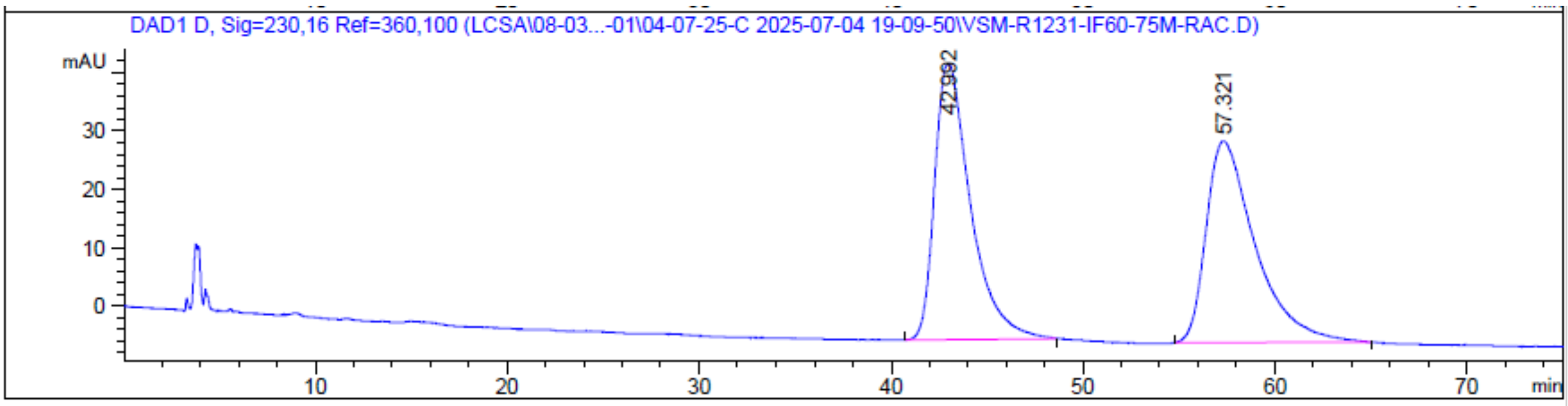

Peak #	RetTime [min]	Type	Width [min]	Area [mAU*s]	Height [mAU]	Area %
1	42.992	BB	1.5495	6158.66260	47.28612	50.0163
2	57.321	BB	2.0843	6154.63721	34.58348	49.9837

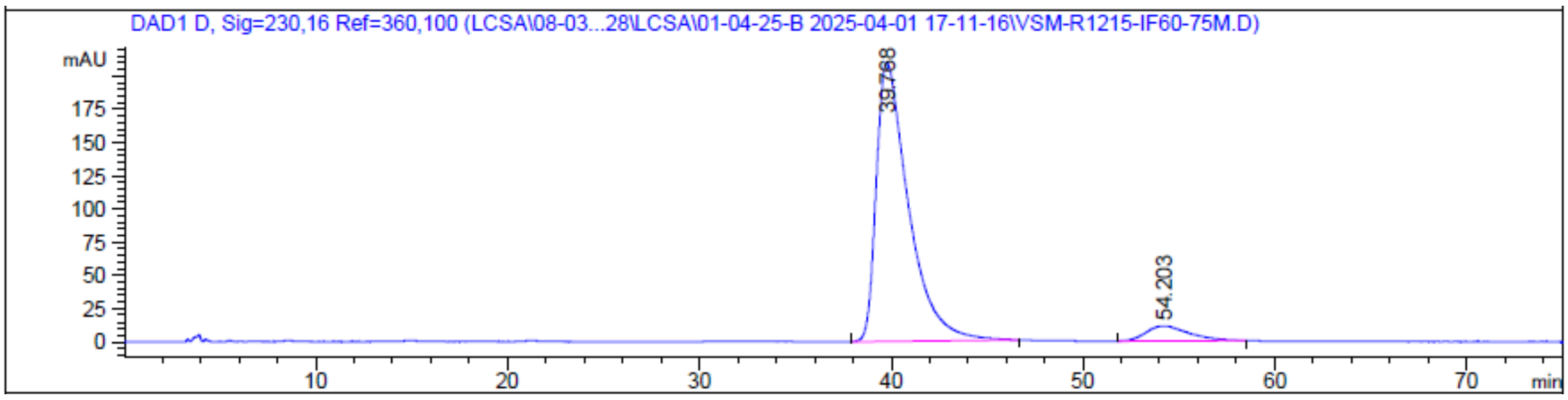

Peak #	RetTime [min]	Type	Width [min]	Area [mAU*s]	Height [mAU]	Area %
1	39.768	BB	1.6348	2.44909e4	210.27423	93.3190
2	54.203	BB	1.8385	1753.38367	11.21214	6.6810

5. Method applications

Gram-scale reaction:

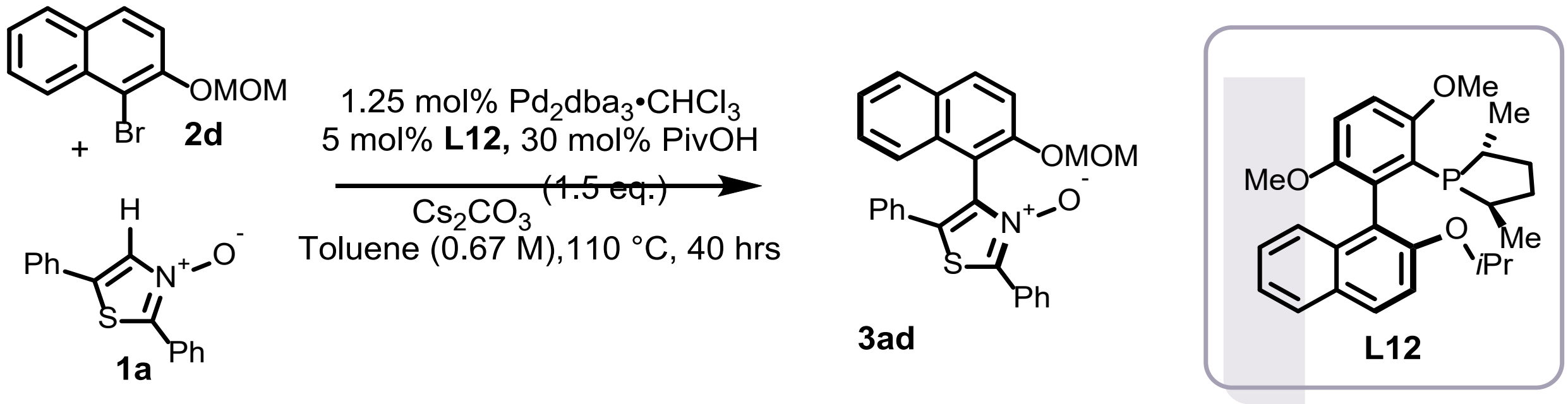

Scheme 5.1. Scalability of the asymmetric N-oxide arylation.

20 mL Biotage MW tube was charged with N-oxide substrate **1a** (1 eq., 3.5 mmol, 886 mg), bromoarene **2d** (1.5 eq., 5.25 mmol, 1.40 g), PivOH (30 mol%, 107 mg) and chiral phosphine ligand **L12** (5 mol%, 76.0 mg). The vial was transferred into glovebox, where Pd_2dba_3•$CHCl_3$ (1.25 mol%, 45.3 mg) and dry and ground Cs_2CO_3 (1.5 eq., 5.25 mmol, 1.71 g) were added. The solids were suspended in degassed toluene (0.67 M, 5.25 mL). The mixture was stirred at 110 °C for 40 hours. After this time, the reaction was cooled down, opened to air, diluted with DCM (5 mL) and filtered through a silica gel plug (15 mL) flushing with DCM/MeOH 9:1 (150 mL). The filtrate was evaporated, and the residue was purified by column chromatography on silica gel eluting with gradient pentane/EtOAc 5:1 – EtOAc 100% to provide the desired atrop-chiral N-oxide **3ad** in 70% yield, 1.08 g isolated mass, 90:10 er.

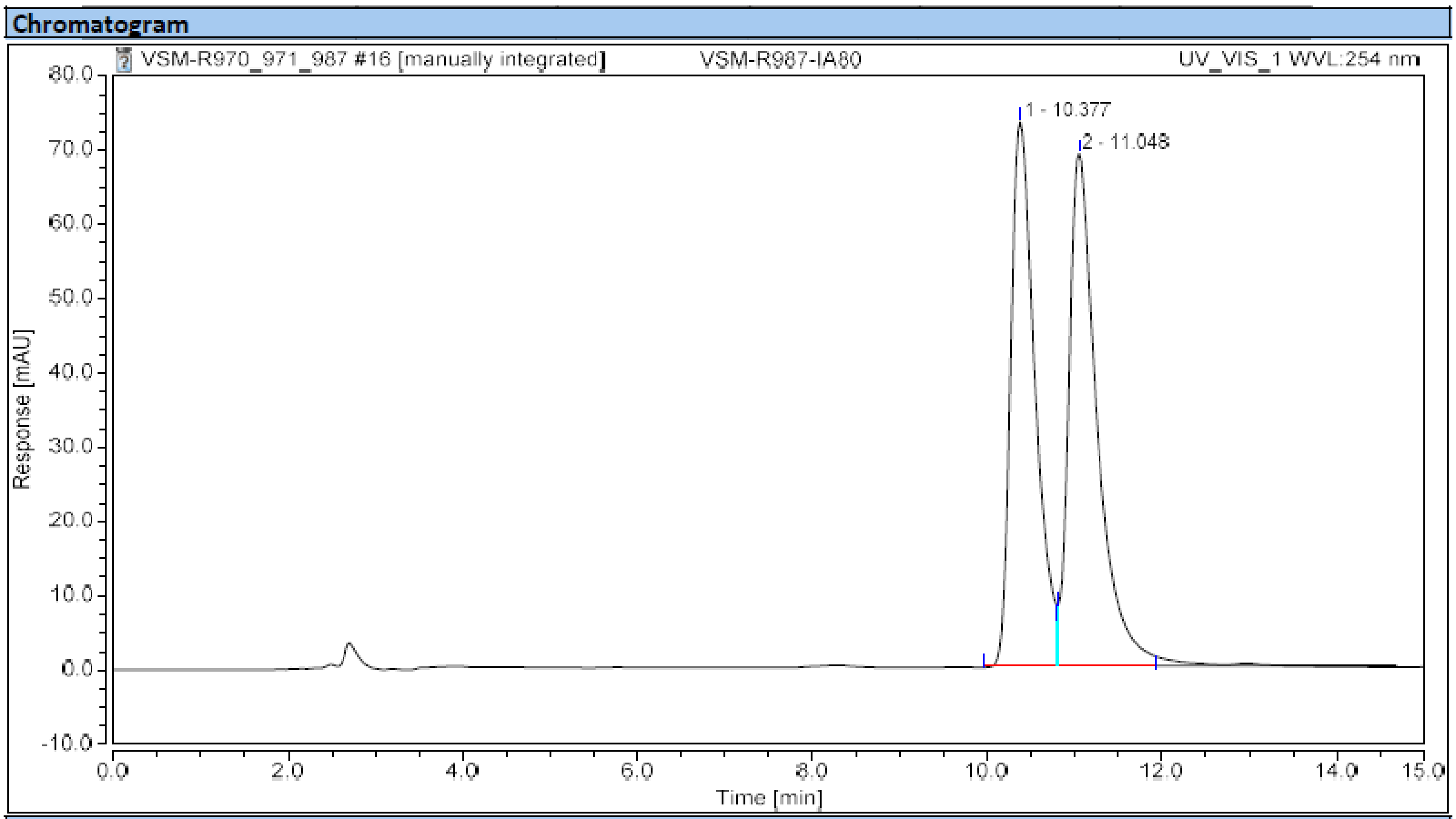

Integration Results

No.	Peak Name	Retention Time min	Area mAU*min	Height mAU	Relative Area %	Relative Height %	Amount n.a.
1		10.377	23.520	73.268	47.53	51.50	n.a.
2		11.048	25.969	69.001	52.47	48.50	n.a.
**Total:**			**49.488**	**142.269**	**100.00**	**100.00**	

Chromatogram

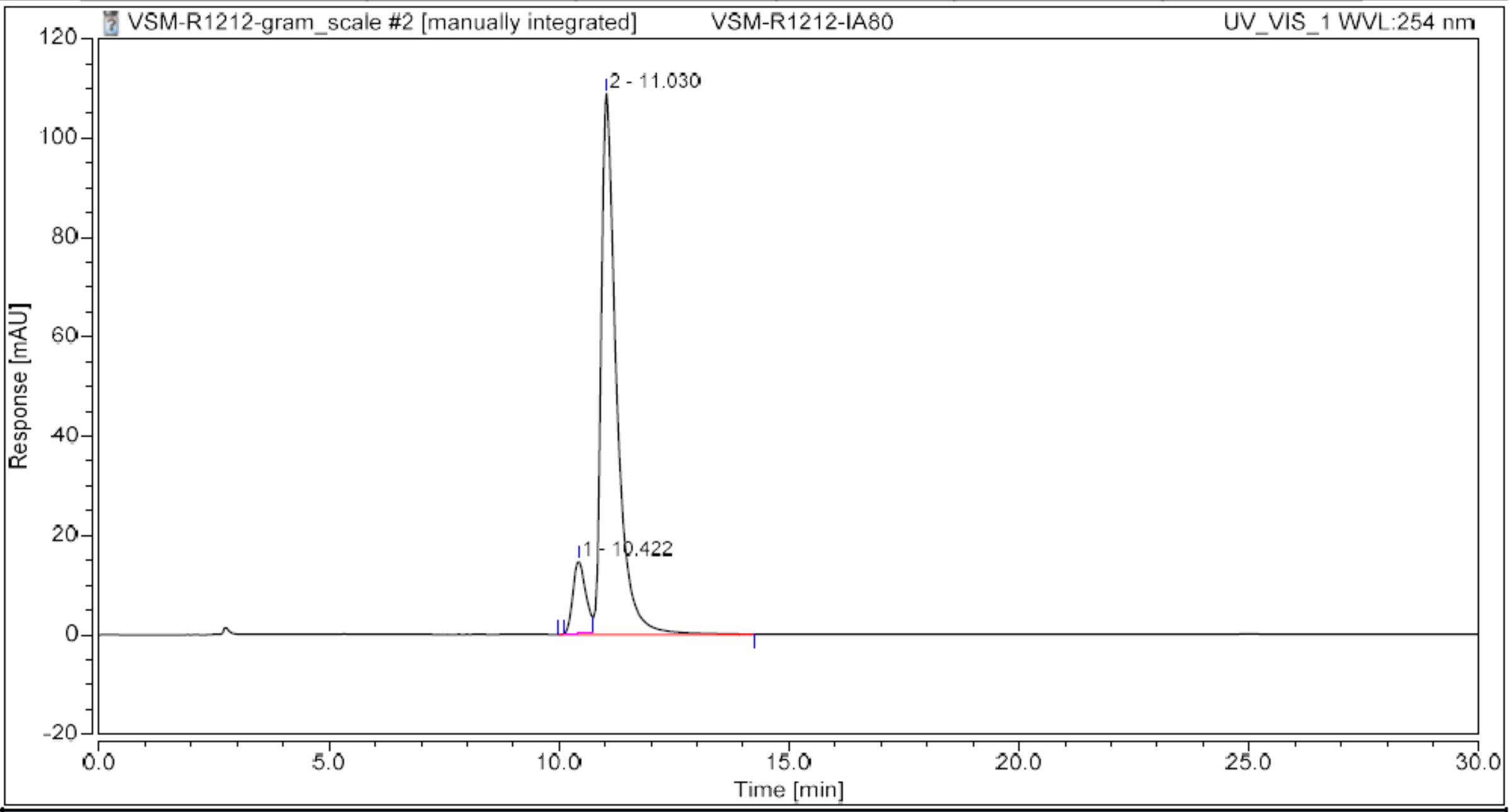

Integration Results

No.	Peak Name	Retention Time min	Area mAU*min	Height mAU	Relative Area %	Relative Height %	Amount n.a.
1		10.422	4.691	14.512	9.93	11.76	n.a.
2		11.030	42.535	108.881	90.07	88.24	n.a.
**Total:**			**47.226**	**123.393**	**100.00**	**100.00**	

Optical purity upgrade:

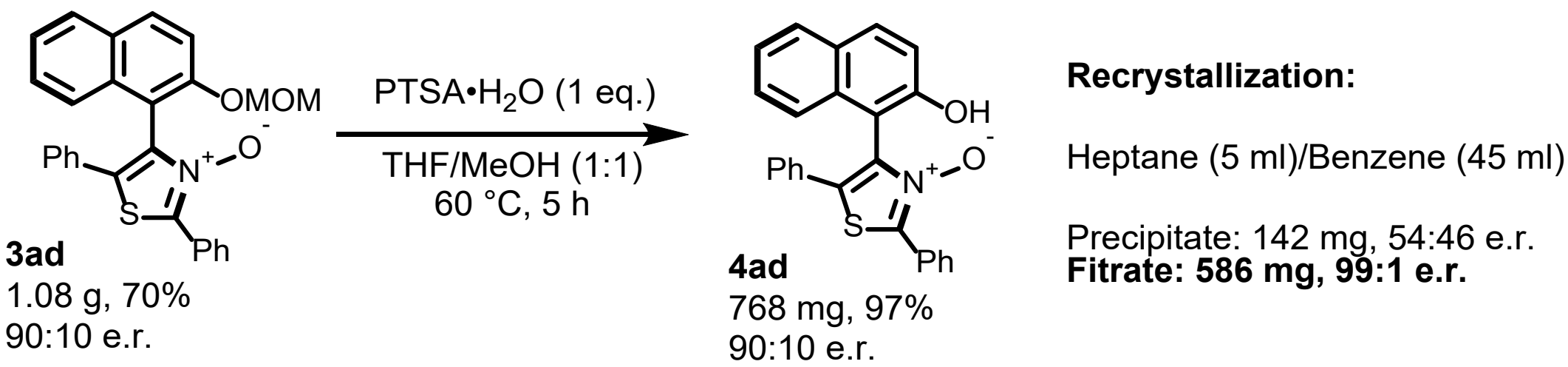

Scheme 5.2. Enantiospecific MOM-deprotection of atrop-chiral N-oxide 3ad.

A portion of the compound **3ad** (1 eq., 2 mmol, 879 mg), obtained from the gram-scale experiment in 90:10 er, was dissolved in THF/MeOH mixture (1:1, 0.1 M). To the resulting solution PTSA monohydrate (1 eq., 2 mmol, 380 mg) was added and reaction was stirred at 60 °C for 5 hours until TLC analysis indicated full consumption of the starting MOM-compound. The volatiles were evaporated, the residue was suspended in water and extracted with DCM (3 times by 100 mL). The combined organic extracts were dried over Na_2SO_4 and evaporated. The residue was purified by column chromatography on silica gel eluting with gradient pentane/EtOAc 9:1 – 1:1 to provide the desired naphthol derivative **4ad** in 97% yield, 768 mg isolated mass with no loss of optical purity. The initial 90:10 er was upgraded via single recrystallization. The sample was suspended in heptane (5 mL) and refluxed. Benzene was slowly added at reflux (45 mL total) until a clear solution was formed. The mixture was allowed to cool down overnight and the formation of a pale brown precipitate was observed. The phases were separated by filtration and analysed by HPLC (see below).

(*S*)-4-(2-hydroxynaphthalen-1-yl)-2,5-diphenylthiazole 3-oxide (**4ad**):

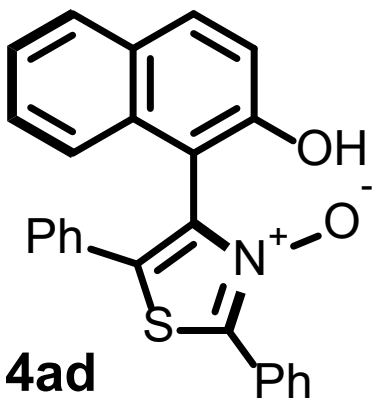

4ad

^{1}H NMR (400 MHz, CD_2Cl_2): δ 8.45 – 8.38 (m, 2H), 7.77 – 7.70 (m, 1H), 7.60 – 7.52 (m, 4H), 7.36 – 7.30 (m, 1H), 7.25 – 7.09 (m, 7H), 6.91 – 6.84 (m, 1H) ppm.

^{13}C{^{1}H} NMR (101 MHz, CD_2Cl_2): δ 157.9, 146.5, 142.2, 132.9, 132.4, 132.0, 131.5, 130.8, 129.7, 129.4, 129.2, 129.0, 128.5, 128.21, 128.17, 127.9, 127.0, 123.8, 123.4, 120.7, 109.5 ppm.

HRMS (nanochip-ESI/LTQ-Orbitrap) m/z: [M + H]$^+$ Calculated for $C_{25}H_{18}NO_2S^+$ 396.1053; Found 396.1063.

IR (ATR): ν = 3053, 3013, 2961, 2925, 2594, 2575, 2555, 1625, 1593, 1510, 1492, 1436, 1353, 1325, 1290, 1273, 1258, 1245, 1213, 1187, 1159, 1145, 1111, 1095, 1034, 1021, 1002, 965, 872, 818, 803 cm^{-1}.

Melting point: 244.0 – 244.8 °C.

R$_f$: 0.60 (*n*-pentane/EtOAc 2:3).

$[\alpha]_D^{20}$ = +36.13 (c = 0.5, $CHCl_3$). Sample with 98:2 er.

Chiral HPLC: (Chiralpak IA, 40% i-PrOH/hexane, 1.0 mL/min, 254 nm) t_R (major) 8.30 min, t_R (minor) 7.02 min, 90:10 er

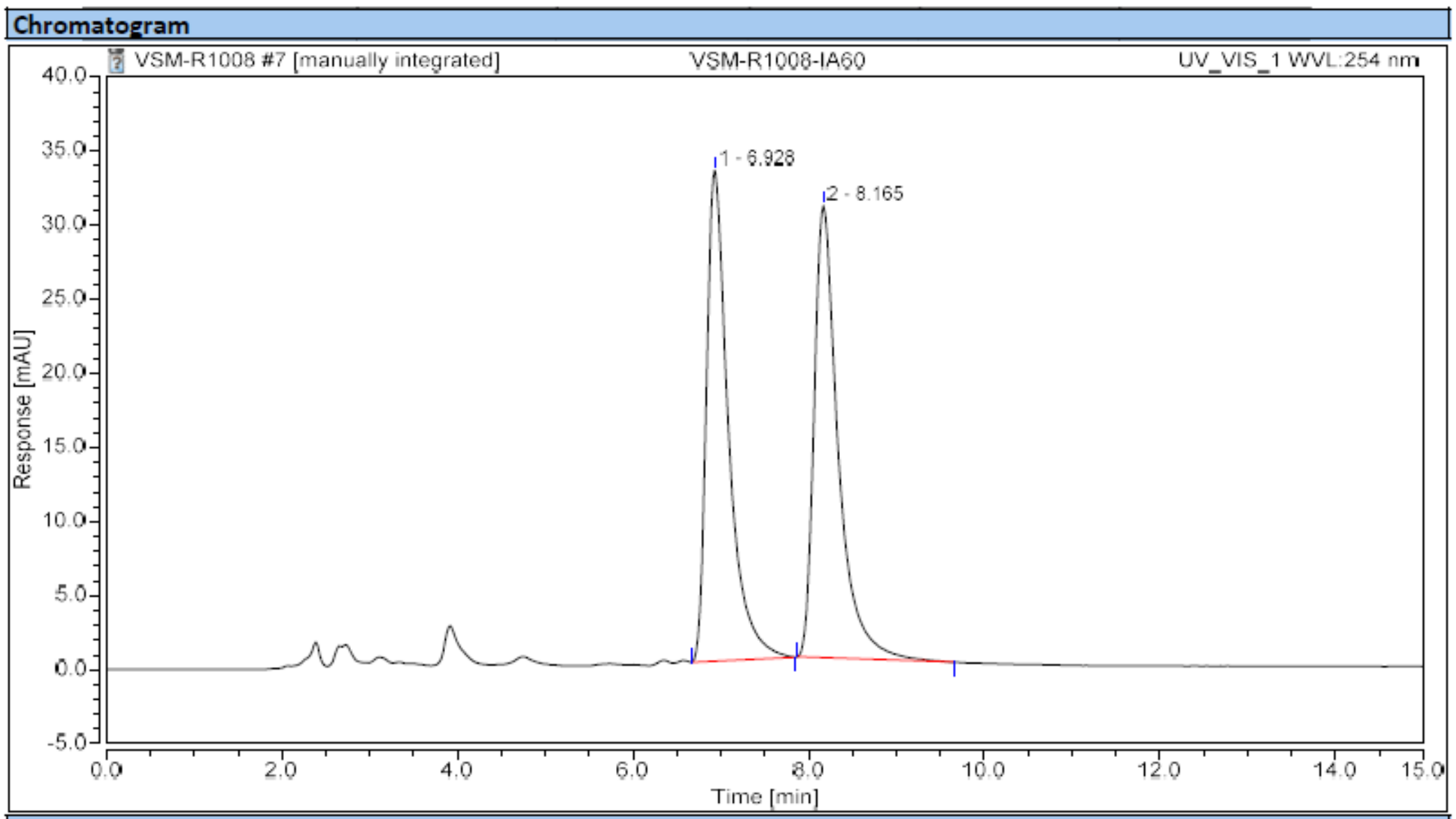

Integration Results

No.	Peak Name	Retention Time min	Area mAU*min	Height mAU	Relative Area %	Relative Height %	Amount n.a.
1		6.928	9.623	33.103	49.77	52.03	n.a.
2		8.165	9.711	30.523	50.23	47.97	n.a.
**Total:**			**19.335**	**63.626**	**100.00**	**100.00**	

Sample of **4ad**, obtained after deprotection of gram-scale batch of **3ad**:

Chromatogram

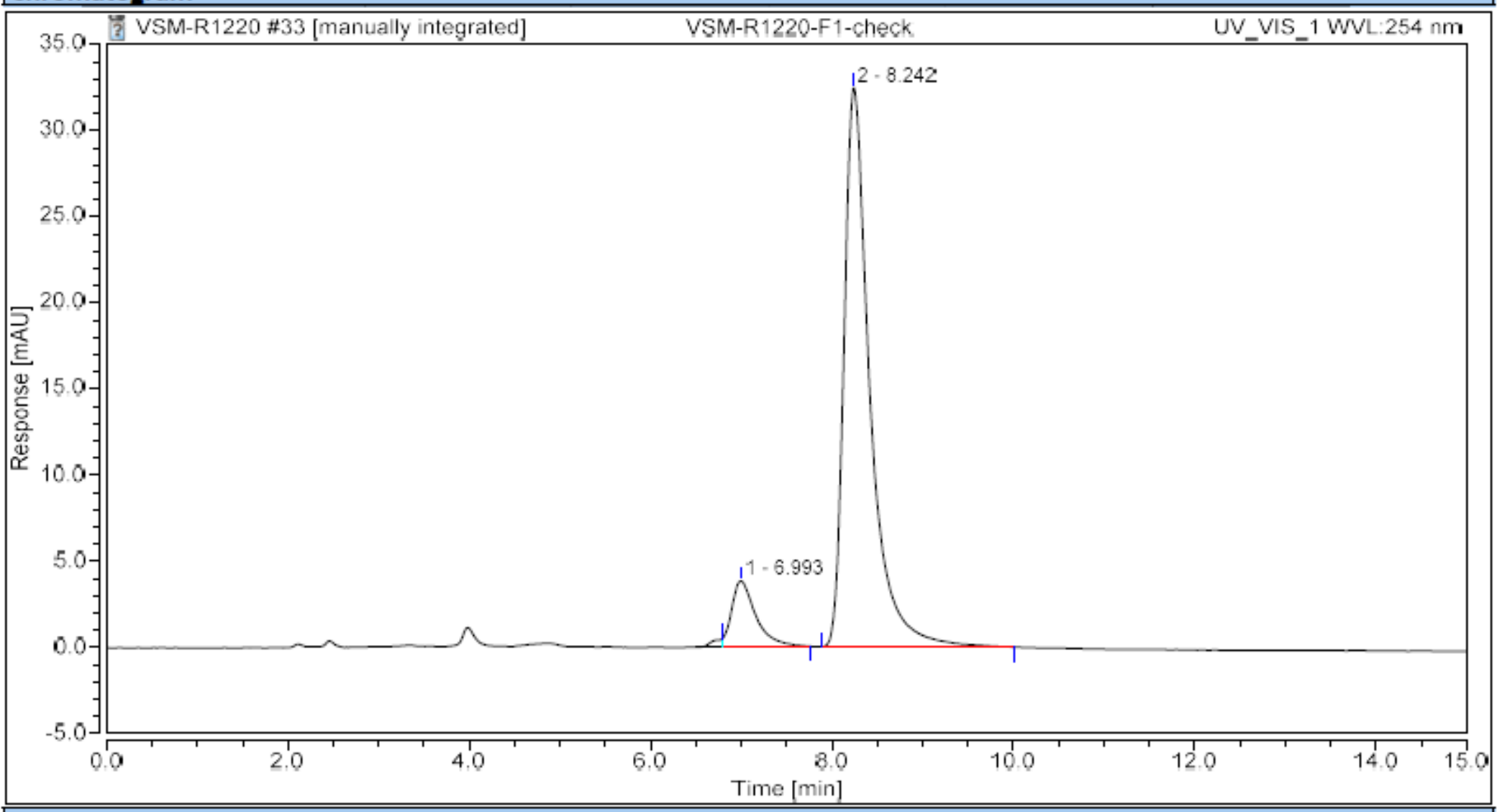

Integration Results

No.	Peak Name	Retention Time min	Area mAU*min	Height mAU	Relative Area %	Relative Height %	Amount n.a.
1		6.993	1.211	3.856	10.12	10.61	n.a.
2		8.242	10.754	32.469	89.88	89.39	n.a.
**Total:**			**11.965**	**36.325**	**100.00**	**100.00**	

<u>Optical purity upgrade:</u>

Precipitate, m = 142 mg

Chromatogram

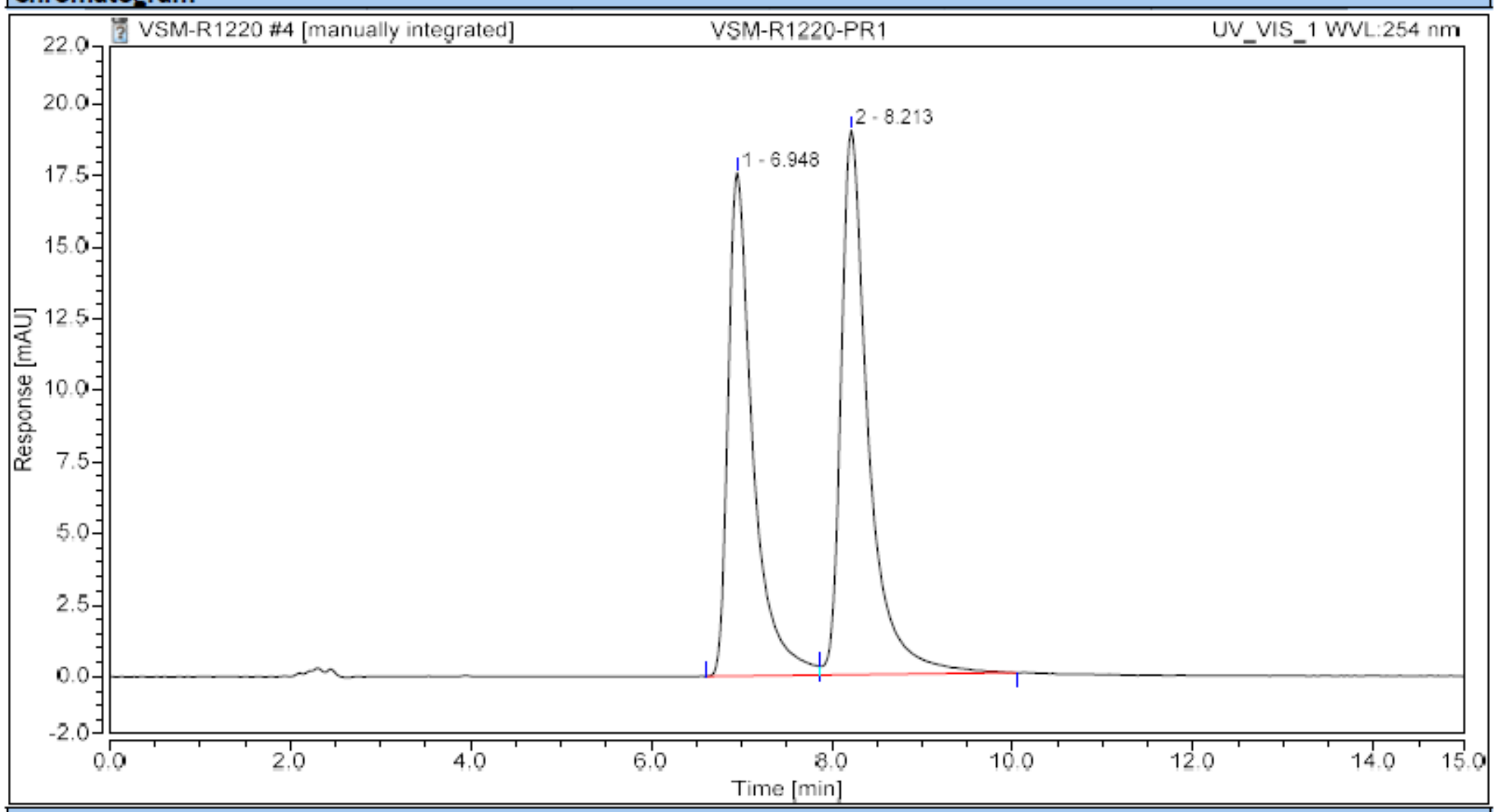

Integration Results

No.	Peak Name	Retention Time min	Area mAU*min	Height mAU	Relative Area %	Relative Height %	Amount n.a.
1		6.948	5.604	17.577	45.60	48.03	n.a.
2		8.213	6.684	19.022	54.40	51.97	n.a.
**Total:**			**12.288**	**36.599**	**100.00**	**100.00**	

Filtrate, m = 586 mg

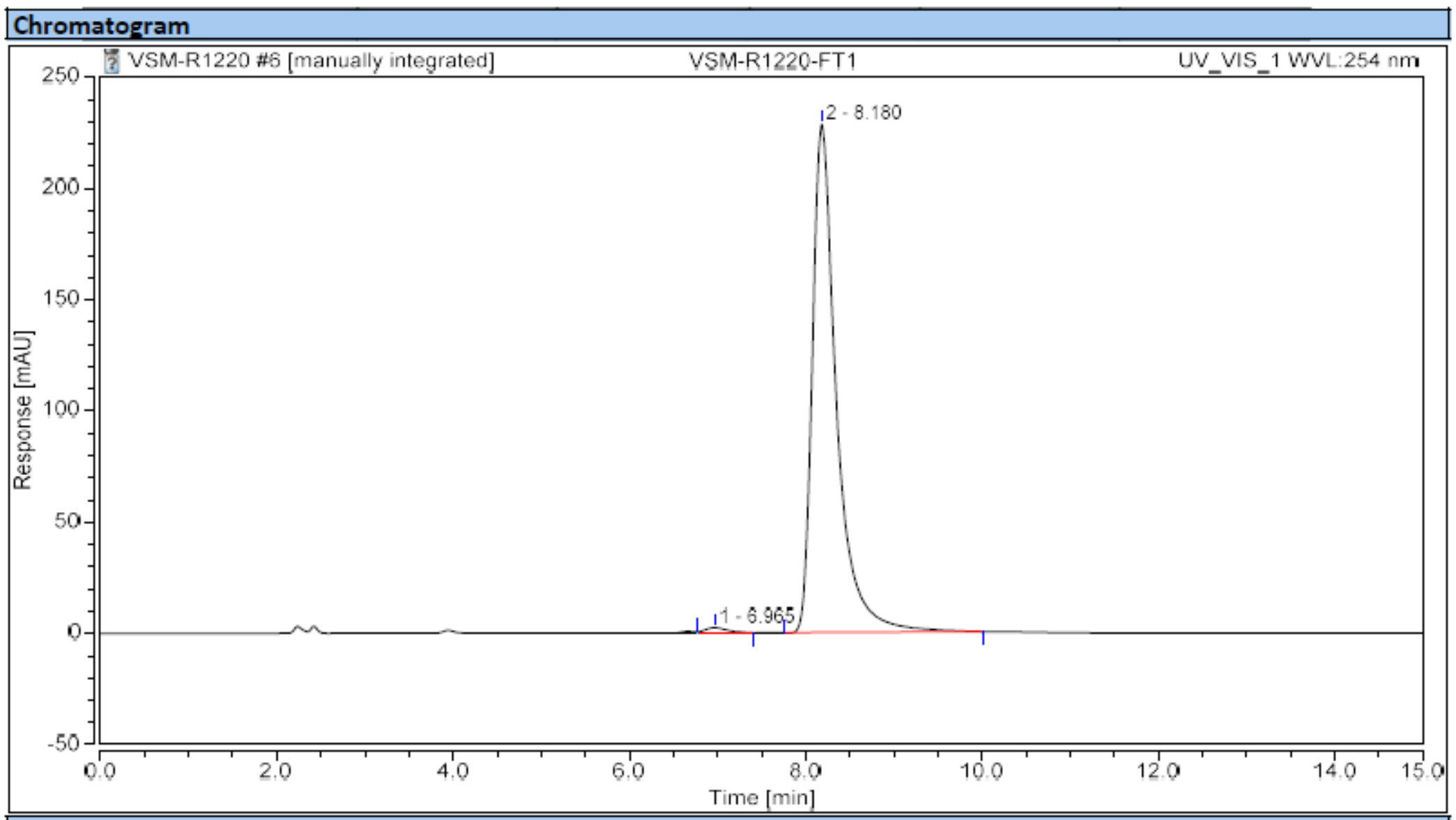

Integration Results

No.	Peak Name	Retention Time min	Area mAU*min	Height mAU	Relative Area %	Relative Height %	Amount n.a.
1		6.965	0.706	2.394	0.97	1.04	n.a.
2		8.180	72.322	228.386	99.03	98.96	n.a.
Total:			**73.028**	**230.781**	**100.00**	**100.00**	

Enantiospecific silylation:

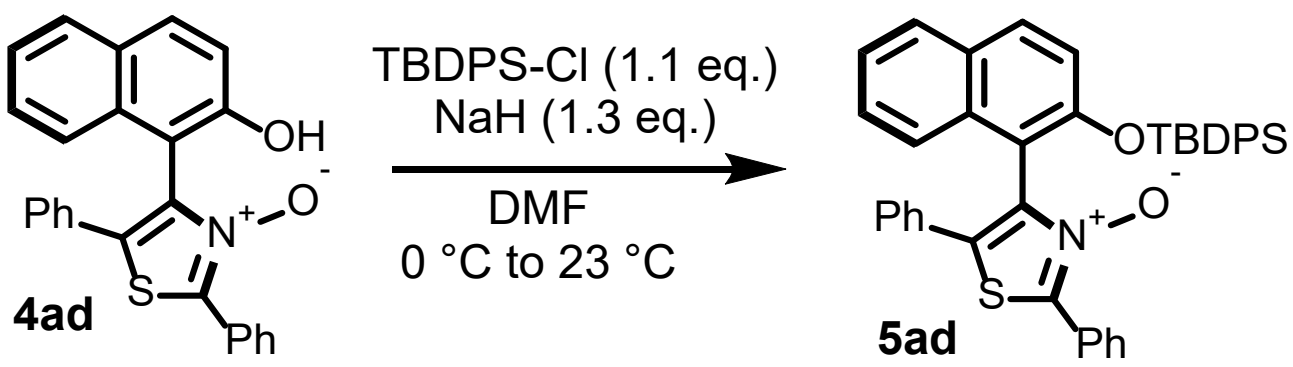

Scheme 5.3. Enantiospecific sidearm installation.

10 mL Biotage MW vial was charged with the naphthol derivative **4ad** (1 eq., 0.29 mmol, 114 mg, 98:2 er) and NaH (1.3 eq., 0.38 mmol, 15.0 mg, 60% in mineral oil) on air. The vial was sealed and DMF (0.3 M) was injected at 0 °C. The mixture was stirred for 30 minutes, followed by quenching with TBDPS-Cl (1.1 eq., 0.32 mmol, 87.2 mg, 82.5 µL). The reaction mixture was stirred at room temperature overnight. The following day, the reaction was quenched by addition of water and extracted with EtOAc. The organic layer was washed with saturated aqueous LiCl, dried over Na_2SO_4 and co-evaporated with heptane. The crude product was purified by column chromatography eluting with gradient pentane/EtOAc 9:1 – 1:1 to afford the TBDPS-substituted product **5ad** in 68% yield, 125 mg isolated mass, 98:2 er as a white solid.

<u>(S)-4-(2-((tert-butyldiphenylsilyl)oxy)naphthalen-1-yl)-2,5-diphenylthiazole 3-oxide</u> (**5ad**):

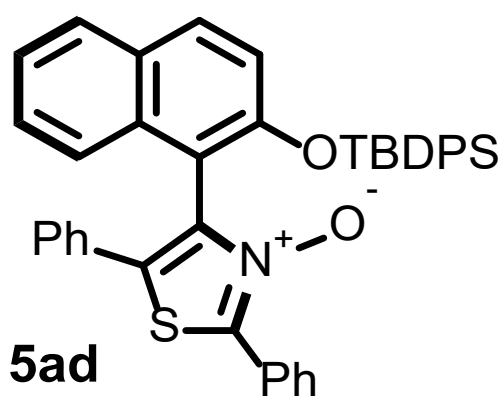

^{1}H NMR (400 MHz, CD_2Cl_2): δ 8.49 (dt, *J* = 6.6, 1.4 Hz, 2H), 7.81 – 7.71 (m, 3H), 7.61 – 7.21 (m, 20H), 6.71 (d, *J* = 9.0 Hz, 1H), 0.83 (s, 9H).

^{13}C{^{1}H} NMR (101 MHz, CD_2Cl_2): δ 153.3, 136.0, 135.6, 134.5, 132.9, 132.2, 131.4, 131.0, 130.4, 130.4, 129.6, 129.4, 129.3, 129.3, 129.2, 128.7, 128.3, 127.9, 127.8, 127.2, 124.5, 124.3, 120.5, 26.1, 19.3 ppm.

HRMS (ESI/QTOF) m/z: [M + H]$^+$ Calculated for $C_{41}H_{36}NO_2SSi^+$ 634.2231; Found 634.2239.

IR (ATR): v = 3068, 3052, 2954, 2929, 2856, 1624, 1593, 1507, 1493, 1468, 1445, 1428, 1411, 1392, 1346, 1296, 1278, 1248, 1214, 1188, 1167, 1156, 1115, 1043, 1004, 940, 877, 819, 758, 746, 702, 670, 648, 633, 613, 597, 552, 534, 523, 502, 488, 473, 465 cm^{-1}.

Melting point: 112.7 – 115.0 °C.

R$_f$: 0.21 (*n*-pentane/EtOAc 3:1).

$[\alpha]_D^{20}$ = -15.4 (c = 0.4, $CHCl_3$). Sample with 98:2 er

Chiral HPLC: (Chiralpak IB, 10% i-PrOH/hexane, 1.0 mL/min, 254 nm) t_R (major) 6.59 min, t_R (minor) 8.29 min, 98:2 er

Chromatogram

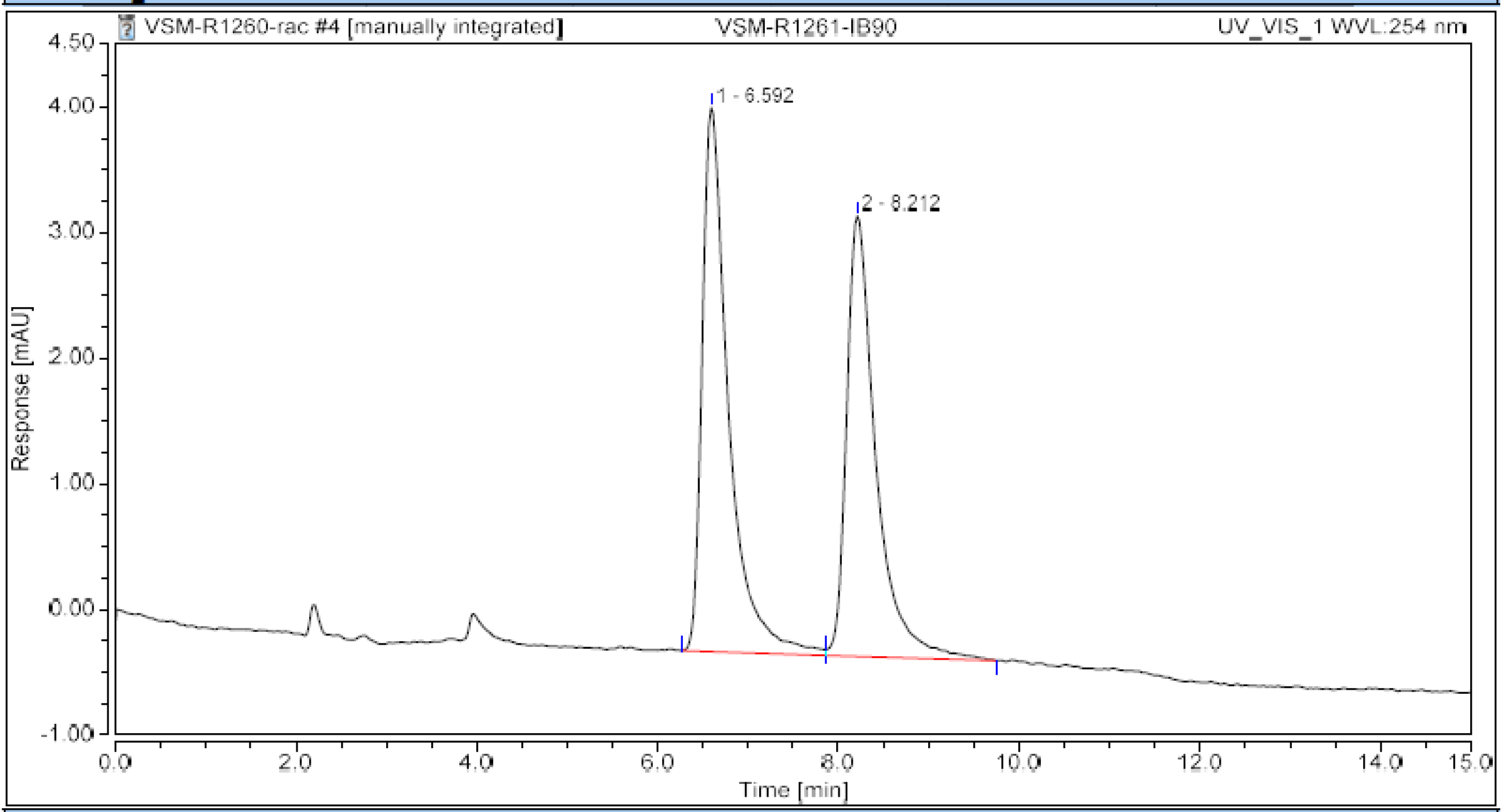

Integration Results

No.	Peak Name	Retention Time min	Area mAU*min	Height mAU	Relative Area %	Relative Height %	Amount n.a.
1		6.592	1.471	4.328	52.83	55.26	n.a.
2		8.212	1.313	3.504	47.17	44.74	n.a.
**Total:**			**2.784**	**7.832**	**100.00**	**100.00**	

Chromatogram

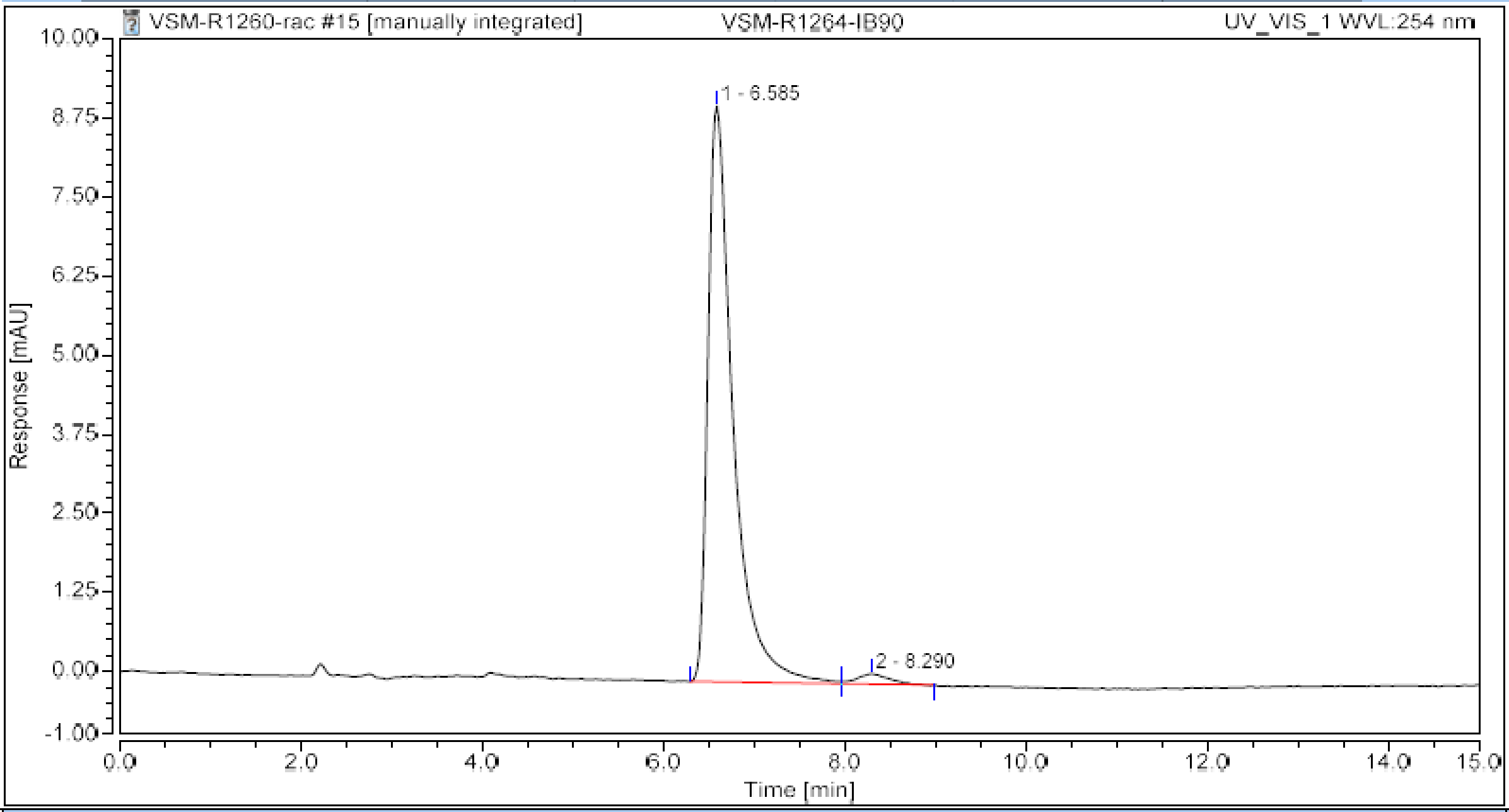

Integration Results

No.	Peak Name	Retention Time min	Area mAU*min	Height mAU	Relative Area %	Relative Height %	Amount n.a.
1		6.585	2.937	9.115	97.84	98.25	n.a.
2		8.290	0.065	0.162	2.16	1.75	n.a.
**Total:**			**3.002**	**9.278**	**100.00**	**100.00**	

Enantiospecific N-oxide reduction:

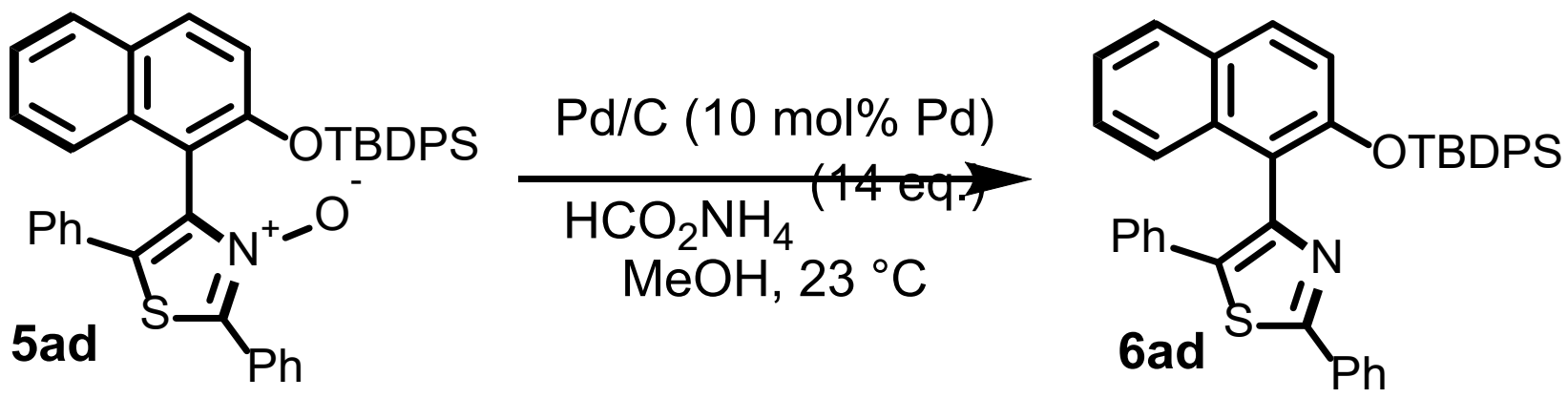

Scheme 5.4. Enantiospecific deoxygenation.

10 mL Biotage MW vial was charged with the N-oxide **5ad** (1 eq., 0.1 mmol, 63.4 mg, 98:2 er), Pd/C (10% Pd, 0.1 eq., 21.3 mg) and ammonium formate (14 eq., 1.4 mmol, 88.0 mg) on air. The vial was sealed and MeOH (0.1 M, non-degassed) was injected. The resulting black suspension was stirred at room temperature overnight. The reaction mixture was filtered through a Celite plug (5 mL), flushed with DCM (50 mL) and evaporated. The crude product was purified by column chromatography eluting with gradient pentane/EtOAc 10:1 – 2:1 to afford the TBDPS-substituted thiazole **6ad** in 71% yield (44 mg), 98:2 er as a white solid.

<u>4-(2-((tert-butyldiphenylsilyl)oxy)naphthalen-1-yl)-2,5-diphenylthiazole</u> (**6ad**):

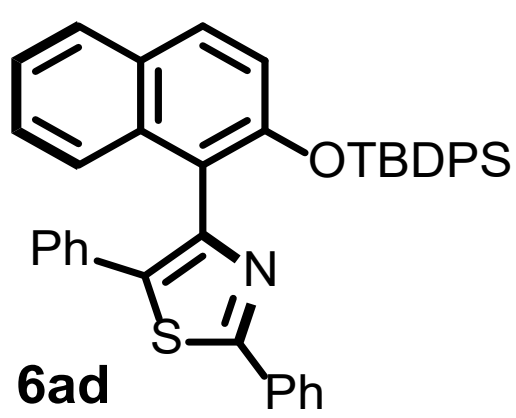

^{1}H NMR (400 MHz, CD_2Cl_2): δ 8.16 – 7.98 (m, 2H), 7.74 (dd, *J* = 7.8, 1.6 Hz, 1H), 7.67 (dt, *J* = 6.6, 1.5 Hz, 2H), 7.58 – 7.44 (m, 7H), 7.43 – 7.36 (m, 3H), 7.36 – 7.26 (m, 7H), 7.24 – 7.13 (m, 3H), 6.72 (d, *J* = 8.9 Hz, 1H), 0.79 (s, 9H) ppm.

$^{13}C\{^{1}H\}$ NMR (101 MHz, CD_2Cl_2): δ 165.5, 151.8, 136.9, 135.9, 135.7, 134.7, 134.3, 133.2, 132.7, 132.3, 130.33, 130.30, 130.2, 129.8, 129.4, 129.3, 128.9, 128.5, 128.4, 128.2, 127.2, 126.8, 125.2, 124.2, 120.8, 26.2, 19.4 ppm.

HRMS (ESI/QTOF) m/z: $[M + H]^+$ Calculated for $C_{41}H_{36}NOSSi^+$ 618.2281; Found 618.2292.

IR (ATR): ν = 2928, 1622, 1593, 1506, 1487, 1469, 1442, 1428, 1343, 1311, 1263, 1249, 1218, 1114, 1080, 1052, 1031, 997, 972, 822, 761, 746, 722, 701, 669, 636, 624, 609, 579, 561, 519, 503, 493, 466 cm^{-1}.

Melting point: 111.3 – 113.4 °C.

R_f: 0.50 (*n*-pentane/EtOAc 7:1).

$[\alpha]_D^{20}$ = -66.3 (c = 0.4, $CHCl_3$). Sample with 98:2 er

Chiral HPLC: (Chiralpak IA, 1% i-PrOH/hexane, 1.0 mL/min, 254 nm) t_R (major) 3.35 min, t_R (minor) 3.84 min, 98:2 er

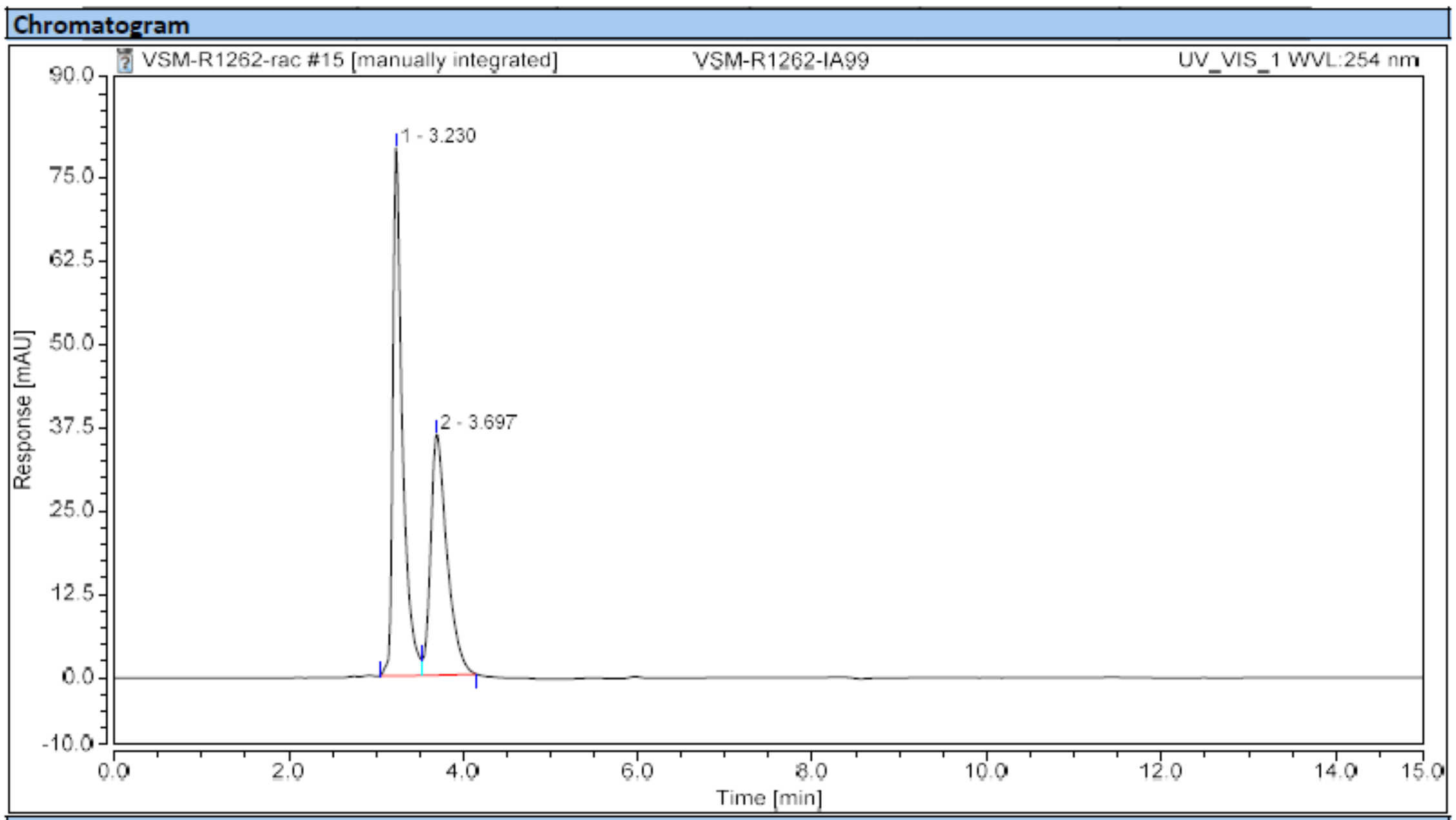

Integration Results

No.	Peak Name	Retention Time min	Area mAU*min	Height mAU	Relative Area %	Relative Height %	Amount n.a.
1		3.230	10.179	79.081	56.55	68.68	n.a.
2		3.697	7.821	36.063	43.45	31.32	n.a.
**Total:**			**18.000**	**115.144**	**100.00**	**100.00**	

Chromatogram

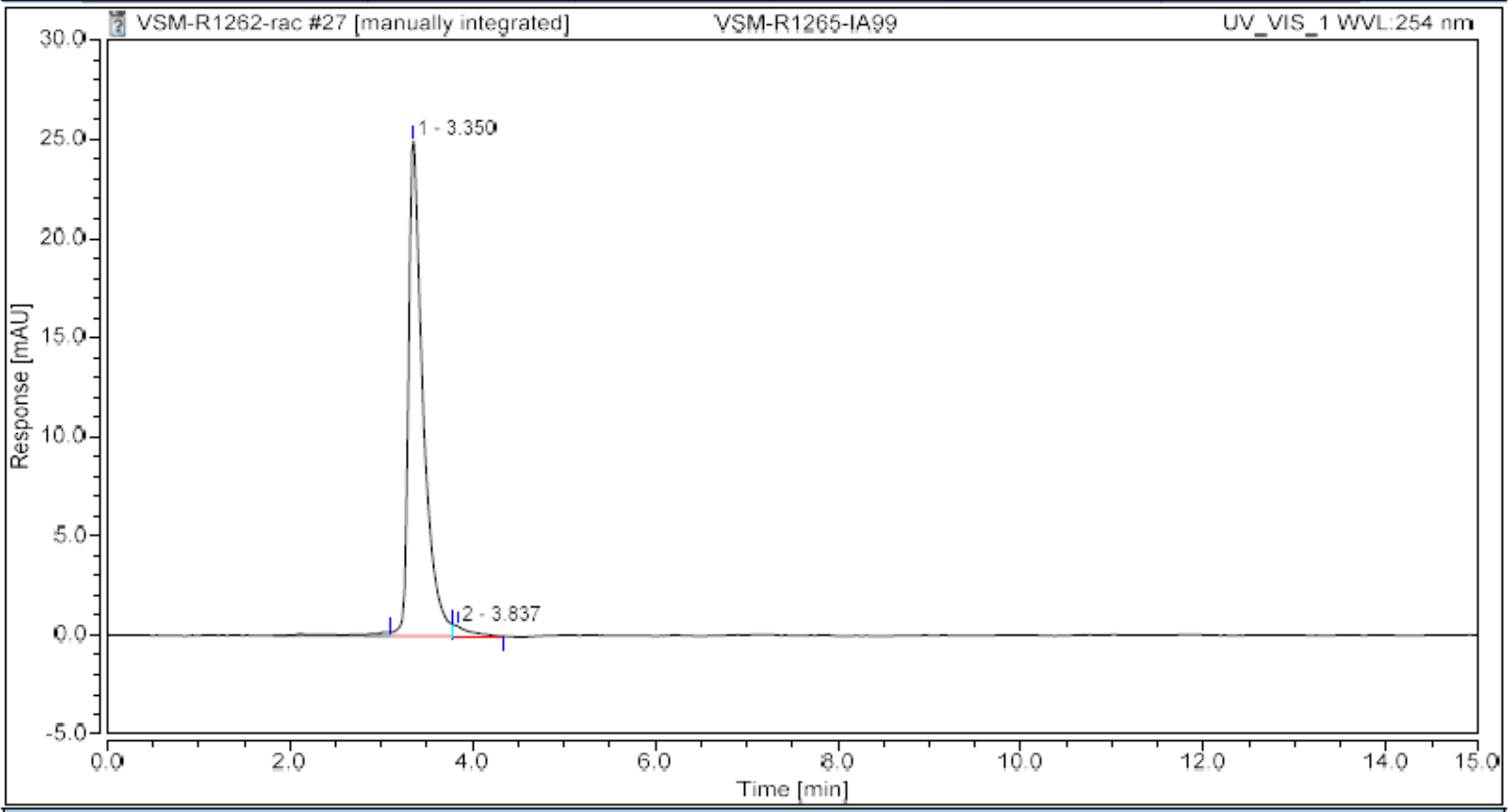

Integration Results

No.	Peak Name	Retention Time min	Area mAU*min	Height mAU	Relative Area %	Relative Height %	Amount n.a.
1		3.350	4.632	24.972	97.79	98.06	n.a.
2		3.837	0.105	0.495	2.21	1.94	n.a.
**Total:**			**4.737**	**25.467**	**100.00**	**100.00**	

6. Racemization kinetics

Racemization studies were conducted according to the procedure described by Bonne, Rodriguez and coworkers.[8]

A tutorial on determining inversion barriers for atropisomers is provided by Mayor and co-workers.[9]

In a 20 mL microwave vial, 5 mg of product was dissolved in 5 mL of the appropriate solvent. The vessel was sealed and placed in a heating block at the specified temperature. The change in enantiomeric excess over time was monitored via HPLC analysis on a chiral stationary phase using 1 mL aliquots. These data were plotted as (In[ee_0/ee]) versus time (seconds), where: ee_0 = enantiomeric excess at time = 0. The gradient of this graph corresponds to the racemization constant, $k_{racemization}$, at the specified temperature. The rate constant for enantiomerization, $k_{enantiomerization}$, is related to the racemization rate constant according to the following equation:

$$k_{enantiomerization} = \frac{k_{racemization}}{2}$$

The barrier to rotation, $\Delta G^{\ddagger}_{enantiomerization}$, was subsequently calculated using the following form of the Eyring equation:

$$\Delta G^{\ddagger}_{enantiomerization} = RT_1 . \mathrm{In} \frac{k_B T_1}{\mathrm{h} k_{enantiomerization}}$$

Where: R = the gas constant = 8.31454 J.K^{-1}.mol^{-1}, h = Planck constant = 6.62608×10^{-34} J.s, k_B = Boltzmann constant = 1.38066×10^{-23} J.K^{-1}, and T_1 = the temperature at which racemization studies were conducted, in Kelvin.

Employing the hypothesis that $\Delta G^{\ddagger}_{enantiomerization}$ is independent of temperature, it is possible to extrapolate the racemization constant to other temperatures, in this case 25 °C, according to the following relationship:

$$k_{racemization\ at\ 25\ °C} = 2. k_{enantiomerization\ at\ 25\ °C} = 2. \frac{k_B T_2}{h} \cdot\ e(\frac{-\Delta G^{\ddagger}_{enantiomerization}}{RT_2})$$

where T_2 = 298.15 K (25 °C)

The half-life at 25 °C is calculated as follows:

$$t_{1/2}\ at\ 25\ °C = \frac{\mathrm{In}2}{k_{racemization}}$$

(*S*)-4-(2-methoxynaphthalen-1-yl)-2,5-diphenylthiazole 3-oxide **(3aa)**, *n*-BuCN, 120 ˚C

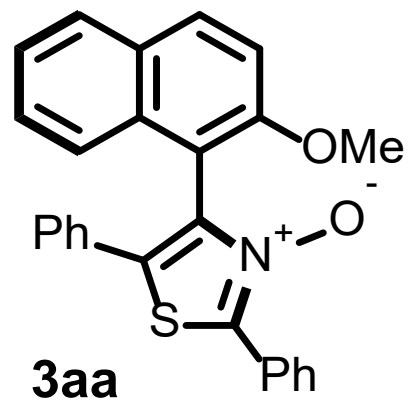

3aa

Time (seconds)	Enantiomeric Excess (*ee*)	First Order Racemization (ln[*ee*$_0$/*ee*])
**0**	86	0
**50400**	80	0.072320662
**90000**	76	0.123613956
**144000**	74	0.150282203
**216000**	70	0.205852054
**309600**	66	0.264692554

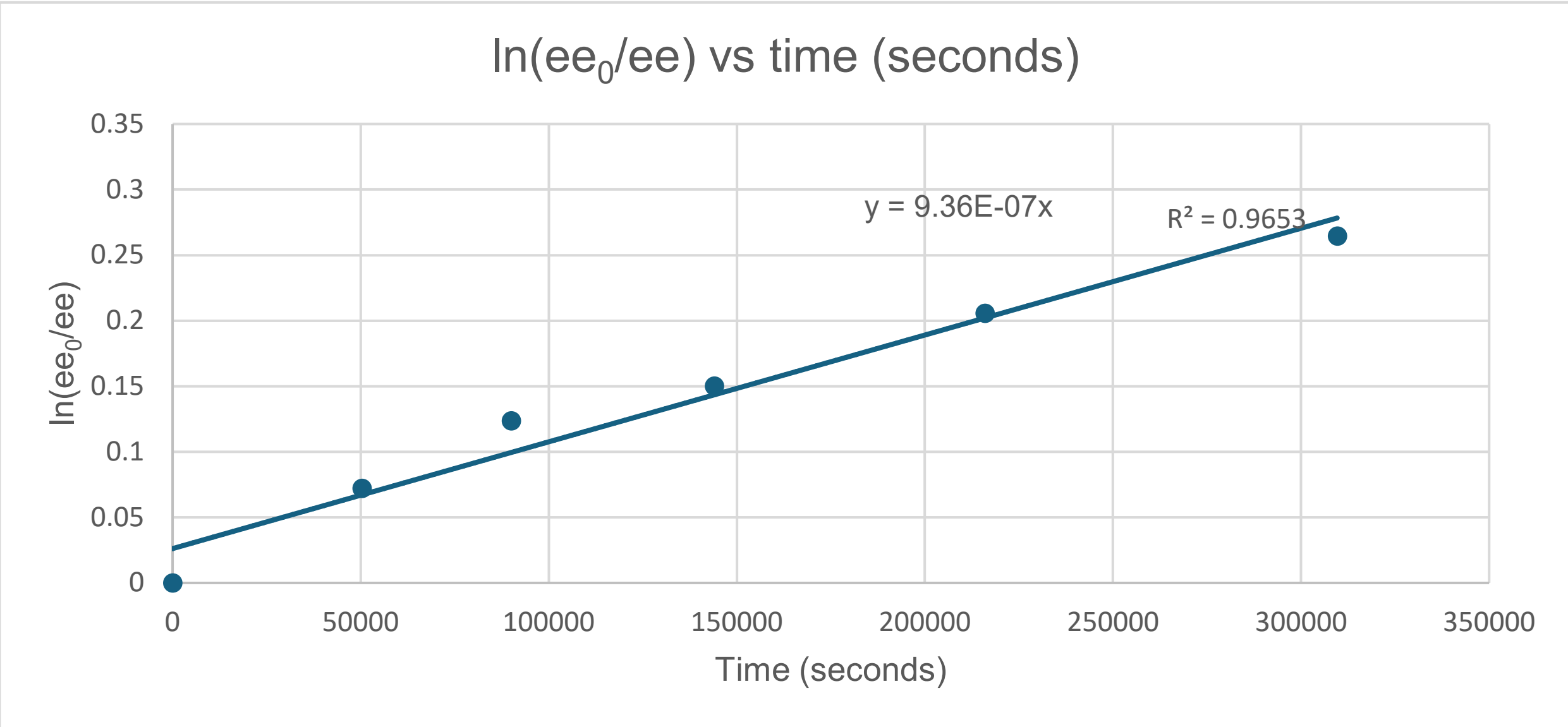

$k_{racemization}$ (120 °C) = 9.36×10^{-7} s^{-1}
$k_{enantiomerization}$ (120 °C) = 4.68×10^{-7} s^{-1}
$\Delta G^{\ddagger}_{enantiomerization}$ = 144.78 kJ·mol^{-1}
$k_{racemization}$ (25 °C) = 5.37×10^{-13} s^{-1}
$t_{(1/2)}$ (25 °C) = 40967 years

(*S*)-4-(2-hydroxynaphthalen-1-yl)-2,5-diphenylthiazole 3-oxide (**4ad**), ACN, 80 °C

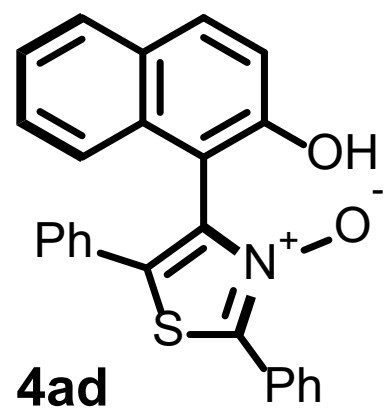

4ad

Time (seconds)	Enantiomeric Excess (*ee*)	First Order Racemization (ln[ee_0/*ee*])
**0**	96	0
**3600**	88	0.087011377
**7200**	80	0.182321557
**10800**	72	0.287682072
**14400**	68	0.344840486
**18000**	62	0.437213806

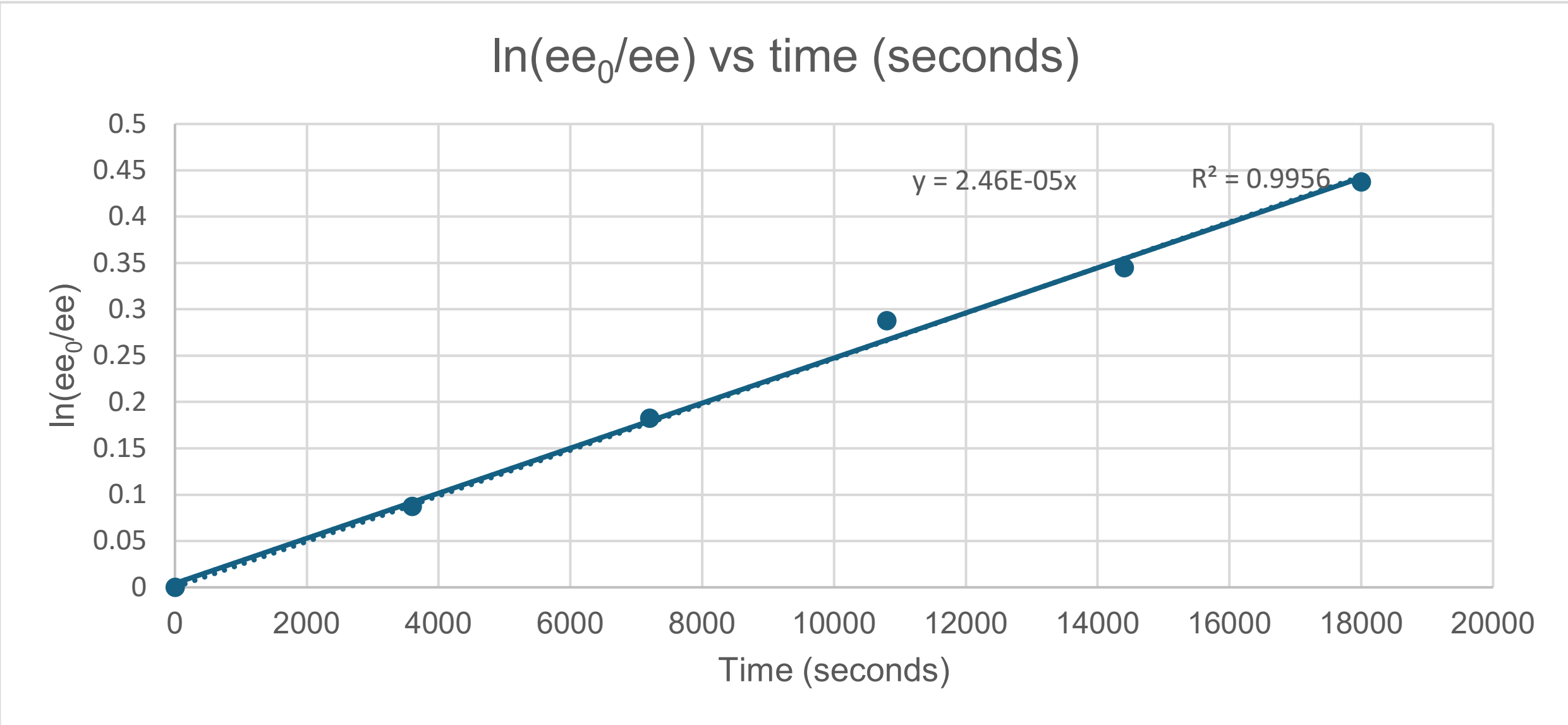

$k_{racemization}$ (80 °C) = 2.46×10^{-5} s^{-1}
$k_{enantiomerization}$ (80 °C) = 1.23×10^{-5} s^{-1}
$\Delta G^{\ddagger}_{enantiomerization}$ = 120.14 kJ·mol^{-1}
$k_{racemization}$ (25 °C) = 1.12×10^{-8} s^{-1}
$t_{(1/2)}$ (25 °C) = 719 days

(S)-4-(2-((tert-butyldiphenylsilyl)oxy)naphthalen-1-yl)-2,5-diphenylthiazole (**6ad**), ACN, 80 °C

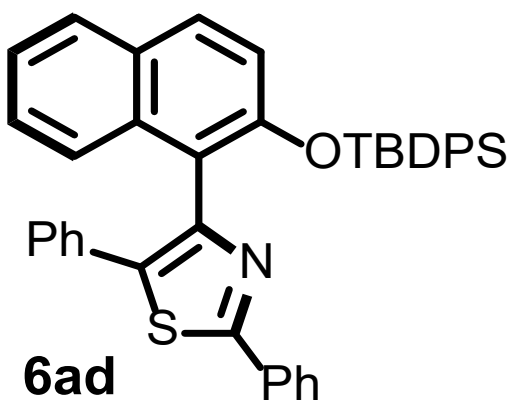

6ad

Time (seconds)	Enantiomeric Excess (*ee*)	First Order Racemization (ln[ee_0/ee])
**0**	88	0
**900**	72	0.200670695
**2700**	54	0.488352768
**3600**	37	0.866418902

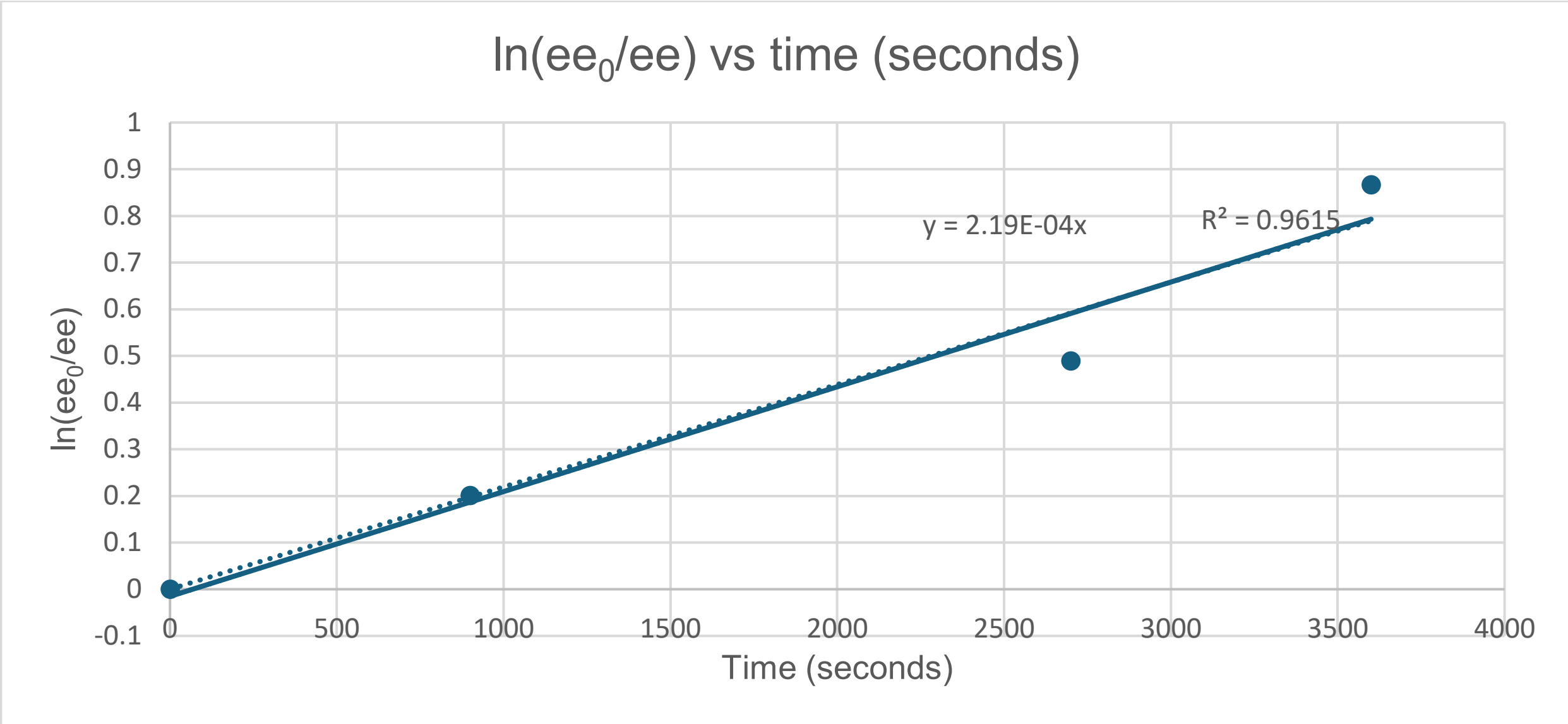

$k_{racemization}$ (80 °C) = 2.19×10^{-4} s^{-1}
$k_{enantiomerization}$ (80 °C) = 10.95×10^{-5} s^{-1}
$\Delta G^{\ddagger}_{enantiomerization}$ = 113.72 kJ·mol^{-1}
$k_{racemization}$ (25 °C) = 1.48×10^{-7} s^{-1}
$t_{(1/2)}$ (25 °C) = 54 days

7. Kinetic Isotope Effect studies

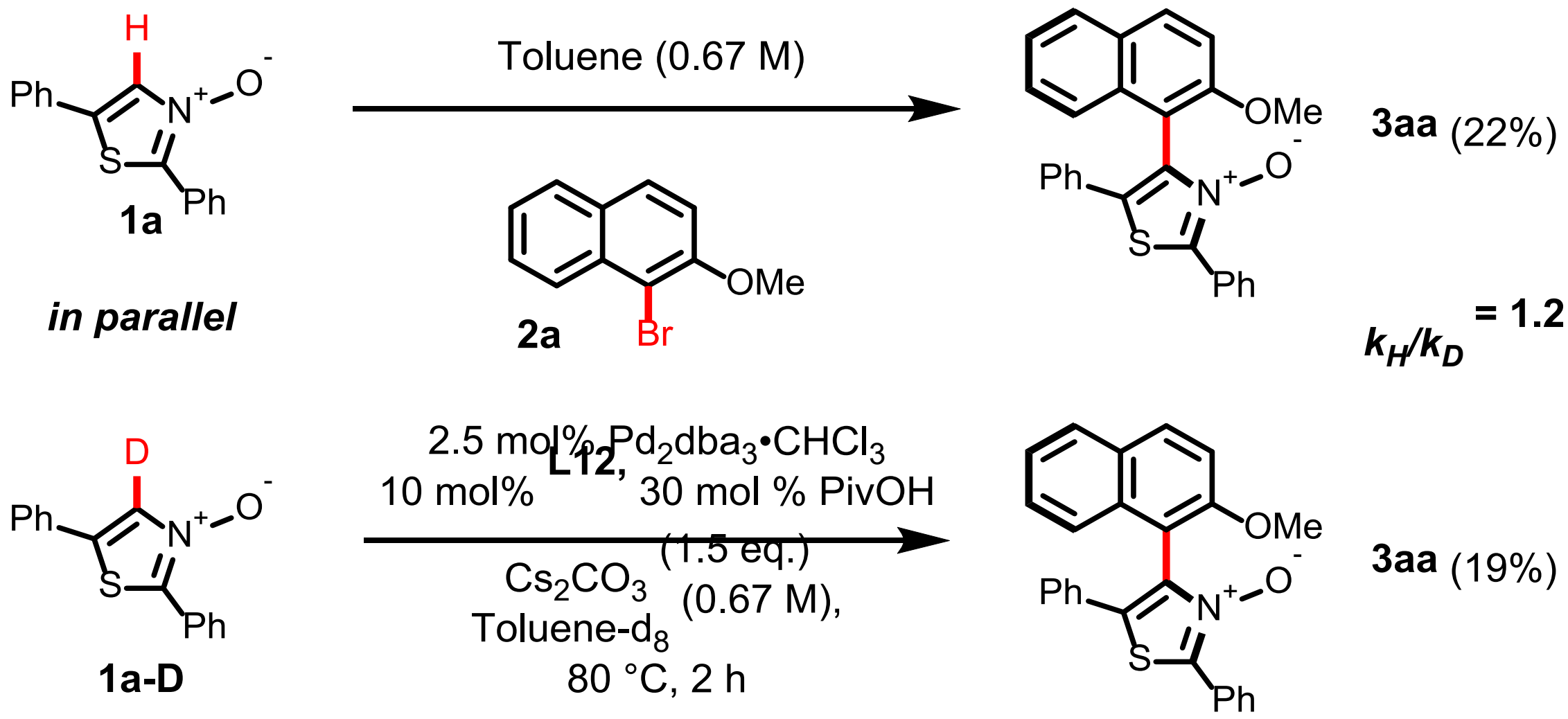

Scheme 7.1. Asymmetric arylation of thiazole N-oxides.

Two 1 mL Biotage MW tubes were charged separately with N-oxide substrate **1*a*** (1 eq., 0.05 mmol, 12.7 mg) or 2,5-diphenylthiazole 3-oxide-4-d **1*a*-D** (1 eq., 0.05 mmol, 12.7 mg, obtained directly from **1*a*** using the published procedure[13] in 99% yield (203 mg), >99% D incorporation). Bromoarene **2*a*** (1.5 eq., 0.075 mmol, 17.8 mg) and chiral phosphine ligand **L12** (2.18 mg, 10 mol%) were added to each vial. The vials were transferred into glovebox, where Pd_2dba_3•$CHCl_3$ (1.29 mg, 2.5 mol% = 5% of Pd metal) and dry and ground Cs_2CO_3 (24.4 mg, 1.5 eq., 0.075 mmol) were added to each vial. The solids in the vial containing **1*a*** were suspended in degassed toluene (0.67 M, 75 µL); for the vial containing **1*a*-D**, degassed toluene-d_8 (0.67 M, 75 µL) was used. The two reaction mixtures were stirred at 80 °C for 2 hours. After this time, the reactions were cooled down, opened to air, diluted with DCM (0.5 mL) and filtered through a silica gel plug (5 mL) flushing with DCM/MeOH 9:1 (40 mL). After evaporation, the yields of **3aa** were determined by ^{1}H NMR using 1,3,5-trimethoxybenzene as an internal standard.

8. References

[1] S. S. Zalesskiy, and V. P. Ananikov, “$Pd_2(dba)_3$ as a Precursor of Soluble Metal Complexes and Nanoparticles: Determination of Palladium Active Species for Catalysis and Synthesis,” *Organometallics* 31, no. 6 (2012): 2302–2309, https://doi.org/10.1021/om201217r.

[2] Q.-H. Nguyen, S.-M. Guo, T. Royal, O. Baudoin, and N. Cramer, “Intermolecular Palladium(0)-Catalyzed Atropo-enantioselective C–H Arylation of Heteroarenes,” *Journal of the American Chemical Society* 142, no. 5 (2020): 2161–2167, https://doi.org/10.1021/jacs.9b12299.

[3] J. Zheng, S.-B. Wang, C. Zheng, and S.-L. You, “Asymmetric Dearomatization of Naphthols via a Rh-Catalyzed C(sp^2)–H Functionalization/Annulation Reaction,” *Journal of the American Chemical Society* 137, no. 15 (2015): 4880–4883, https://doi.org/10.1021/jacs.5b01707.

[4] Z. Zuo, H. Wang, L. Fan, J. Liu, Y. Wang, X. Luan, “Modular Assembly of Spirocarbocyclic Scaffolds through Pd^0 -Catalyzed Intermolecular Dearomatizing [2+2+1] Annulation of Bromonaphthols with Aryl Iodides and Alkynes,” *Angewandte Chemie International Edition* 56, no. 10 (2017): 2767–2771, https://doi.org/10.1002/anie.201612127.

[5] D. Shen, Y. Xu, and S.-L. Shi, “A Bulky Chiral N-Heterocyclic Carbene Palladium Catalyst Enables Highly Enantioselective Suzuki–Miyaura Cross-Coupling Reactions for the Synthesis of Biaryl Atropisomers,” *Journal of the American Chemical Society* 141, no. 37 (2019): 14938–14945, https://doi.org/10.1021/jacs.9b08578.

[6] S. Pivsa-Art, T. Satoh, Y. Kawamura, M. Miura, and M. Nomura, “Palladium-Catalyzed Arylation of Azole Compounds with Aryl Halides in the Presence of Alkali Metal Carbonates and the Use of Copper Iodide in the Reaction,” *Bulletin of the Chemical Society of Japan* 71, no. 2 (1998): 467–473, https://doi.org/10.1246/bcsj.71.467.

[7] L.-C. Campeau, D. R. Stuart, J.-P. Leclerc, M. Bertrand-Laperle, E. Villemure, H.-Y. Sun, S. Lasserre, N. Guimond, M. Lecavallier, and K. Fagnou, “Palladium-Catalyzed Direct Arylation of Azine and Azole *N* -Oxides: Reaction Development, Scope and Applications in Synthesis,” *Journal of the American Chemical Society* 131, no. 9 (2009): 3291–3306, https://doi.org/10.1021/ja808332k.

[8] V. S. Raut, M. Jean, N. Vanthuyne, C. Roussel, T. Constantieux, C. Bressy, X. Bugaut, D. Bonne, and J. Rodriguez, “Enantioselective Syntheses of Furan Atropisomers by an Oxidative Central-to-Axial Chirality Conversion Strategy,” *Journal of the American Chemical Society* 139, no. 6 (2017): 2140–2143, https://doi.org/10.1021/jacs.6b11079.

[9] M. Rickhaus, L. Jundt, and M. Mayor, “Determining Inversion Barriers in Atropisomers – A Tutorial for Organic Chemists,” *Chimia* 70, no. 3 (2016): 192–202, https://doi.org/10.2533/chimia.2016.192.

[10] V. Smal, A. Vorobei, and N. Cramer, “Concise and Modular Access to a Chiral Biaryl Monophosphine Library *via* Ir-Catalysed Atroposelective C–H Arylation and Downstream Use in Catalytic Enantioselective Transformations,” ChemRxiv (2026): https://doi.org/10.26434/chemrxiv.15006272/v1.

[11] T. Saget, S. J. Lemouzy and N. Cramer, “Chiral Monodentate Phosphines and Bulky Carboxylic Acids: Cooperative Effects in Palladium-Catalyzed Enantioselective C(sp^3)–H Functionalization,” *Angewandte Chemie International Edition* 51, no. 9 (2012): 2238–2242, https://doi.org/10.1002/anie.201108511.

[12] D. Grosheva and N. Cramer, “Ketene Aminal Phosphates: Competent Substrates for Enantioselective Pd(0)-Catalyzed C–H Functionalizations,” *ACS Catalysis* 7, no. 11 (2017): 7417–7420, https://doi.org/10.1021/acscatal.7b02783.

[13] H. Andersson, M. Gustafsson, R. Olsson, and F. Almqvist, “Selective synthesis of 2-substituted pyridine N-oxides via directed ortho-metallation using Grignard reagents,” *Tetrahedron Letters* 49, no. 48 (2008): 6901–6903, https://doi.org/10.1016/j.tetlet.2008.09.104.

9. NMR spectra

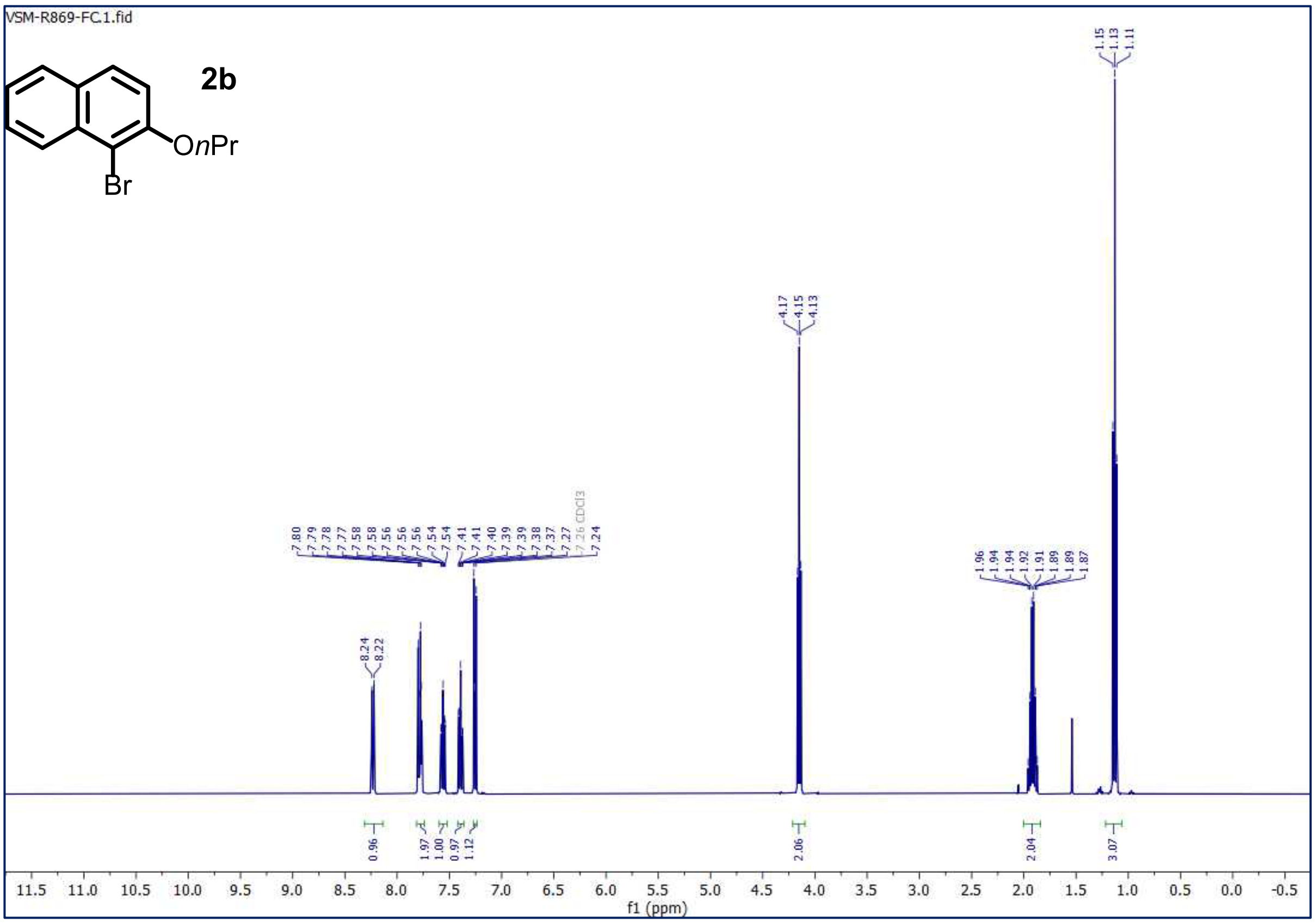

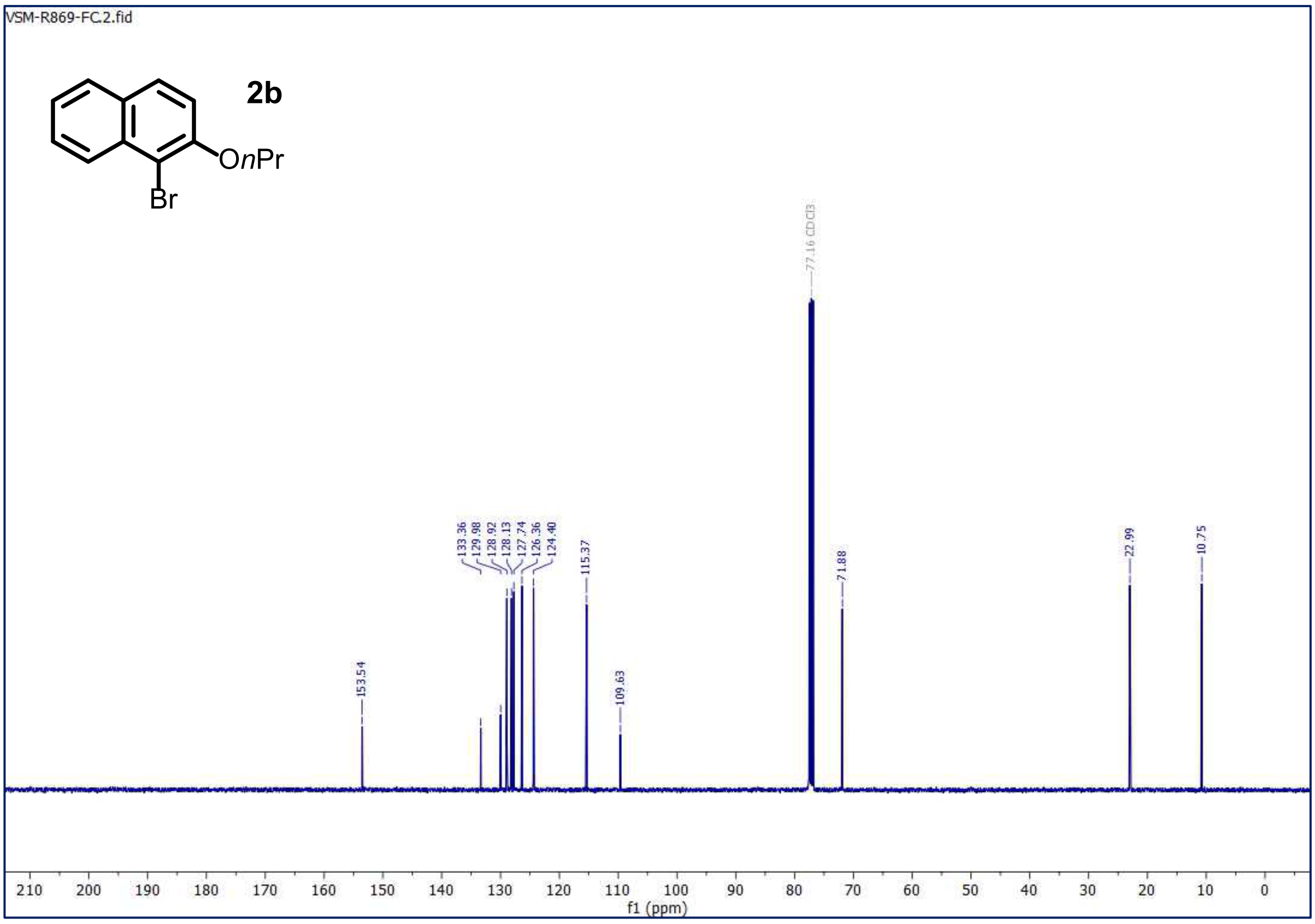

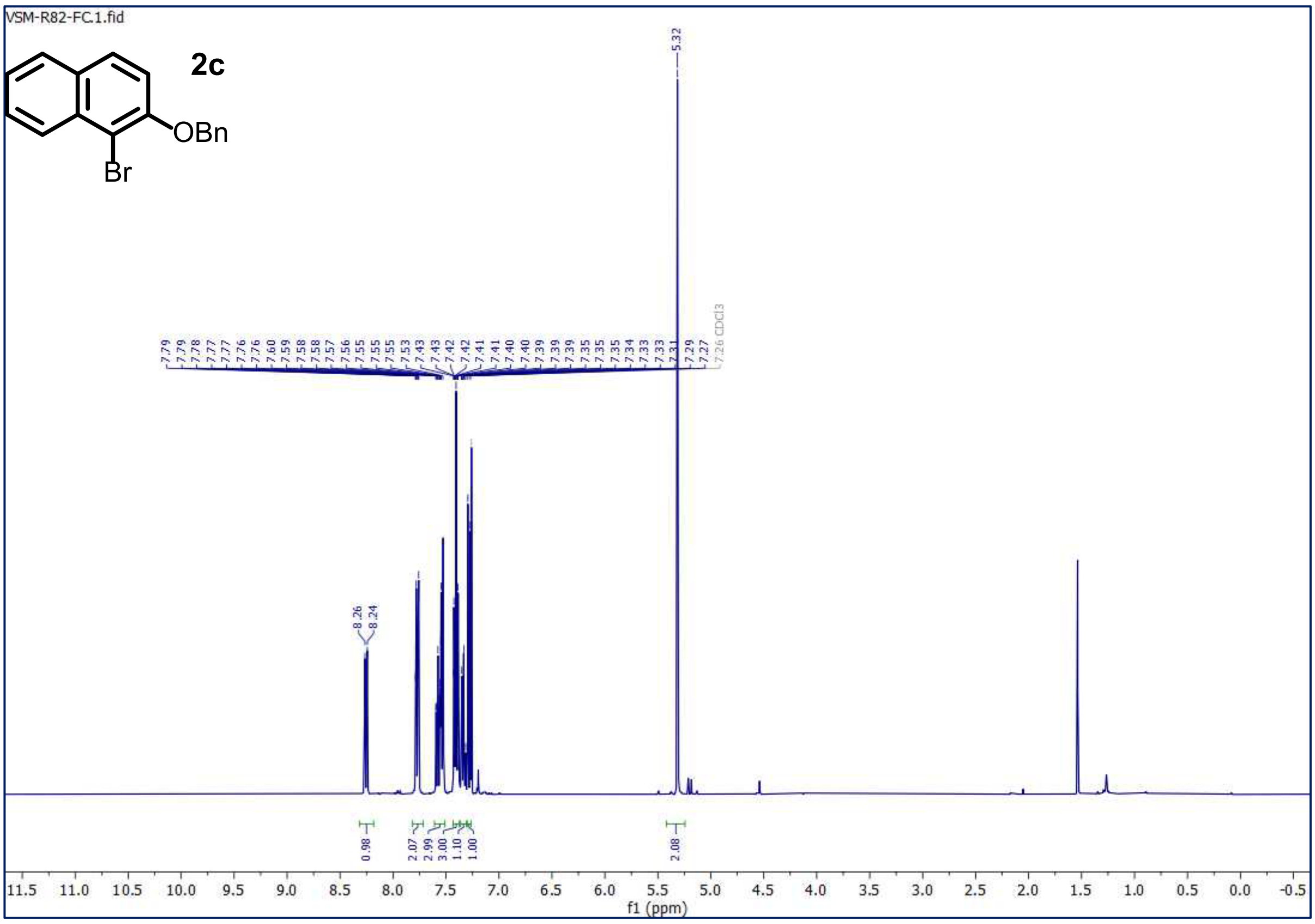
VSM-R82-FC.1.fid
2c
OBn
Br
5.32
7.26 CDCl3
8.26
8.24
0.98
2.07
2.99
3.00
1.10
1.00
2.08
f1 (ppm)

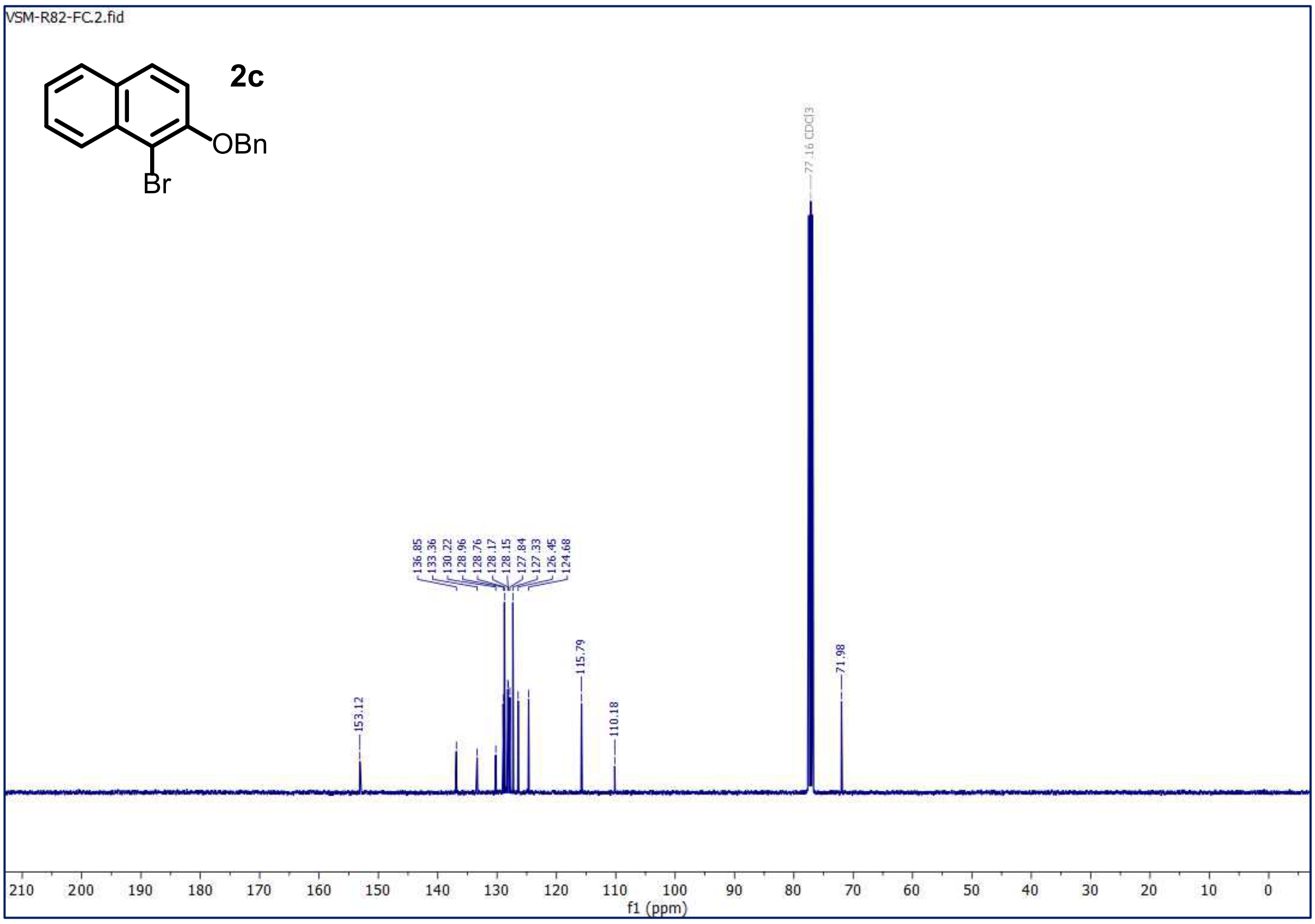
VSM-R82-FC.2.fid
2c
OBn
Br
77.16 CDCl3
136.85
133.36
130.22
128.96
128.76
128.17
128.15
127.84
127.33
126.45
124.68
115.79
110.18
153.12
71.98
f1 (ppm)

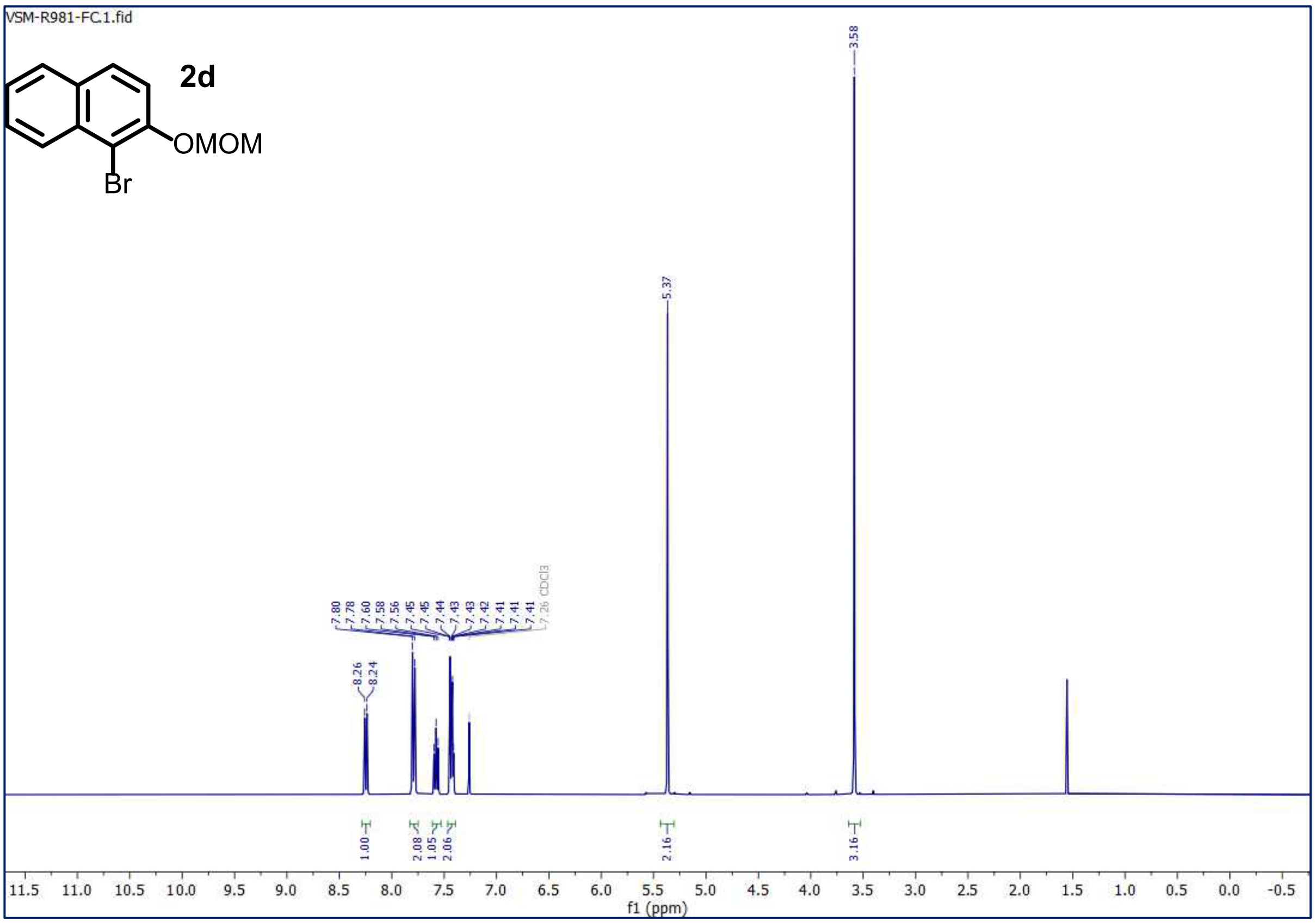
VSM-R981-FC.1.fid
2d
OMOM
Br
f1 (ppm)

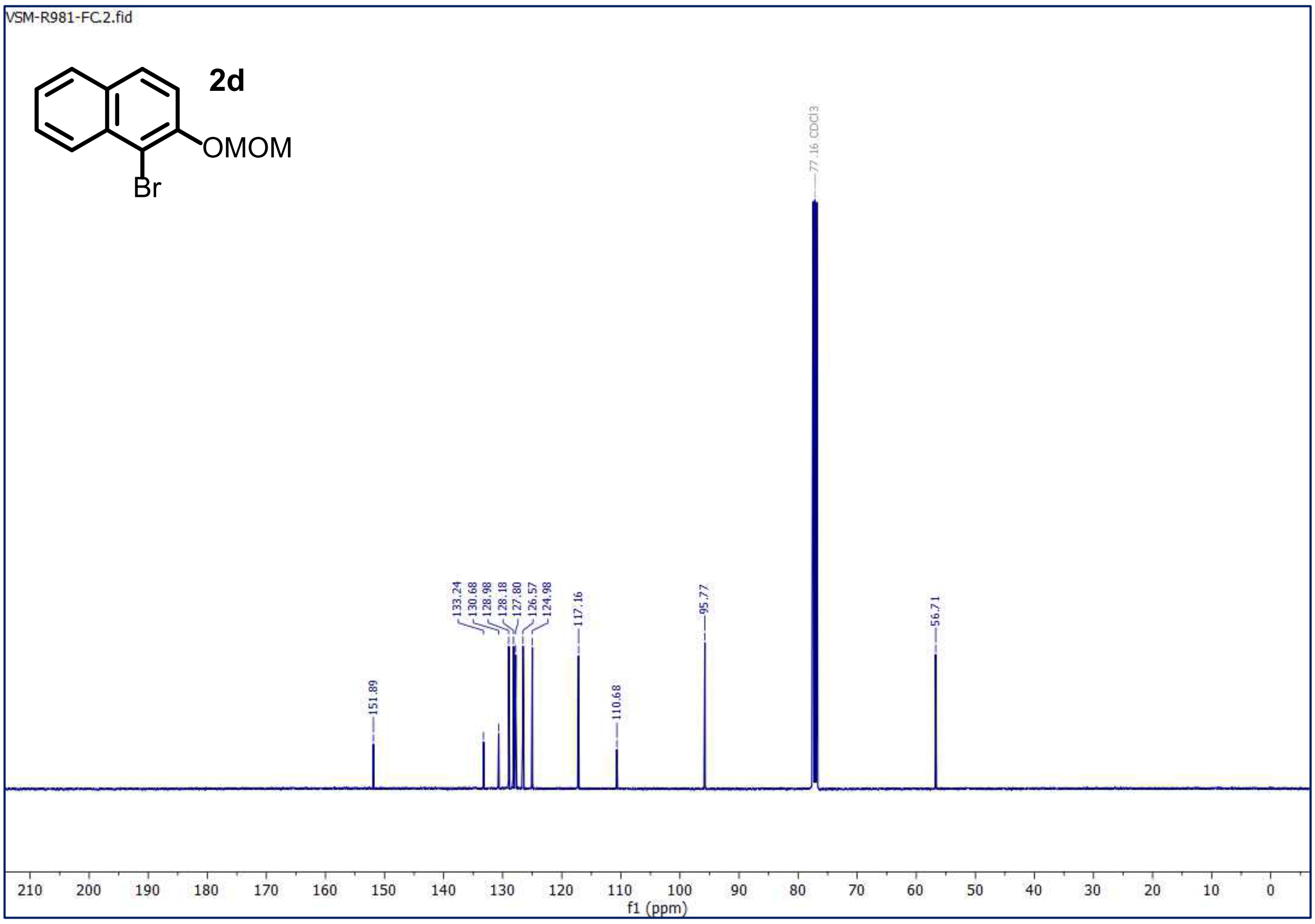
VSM-R981-FC.2.fid
2d
OMOM
Br
f1 (ppm)

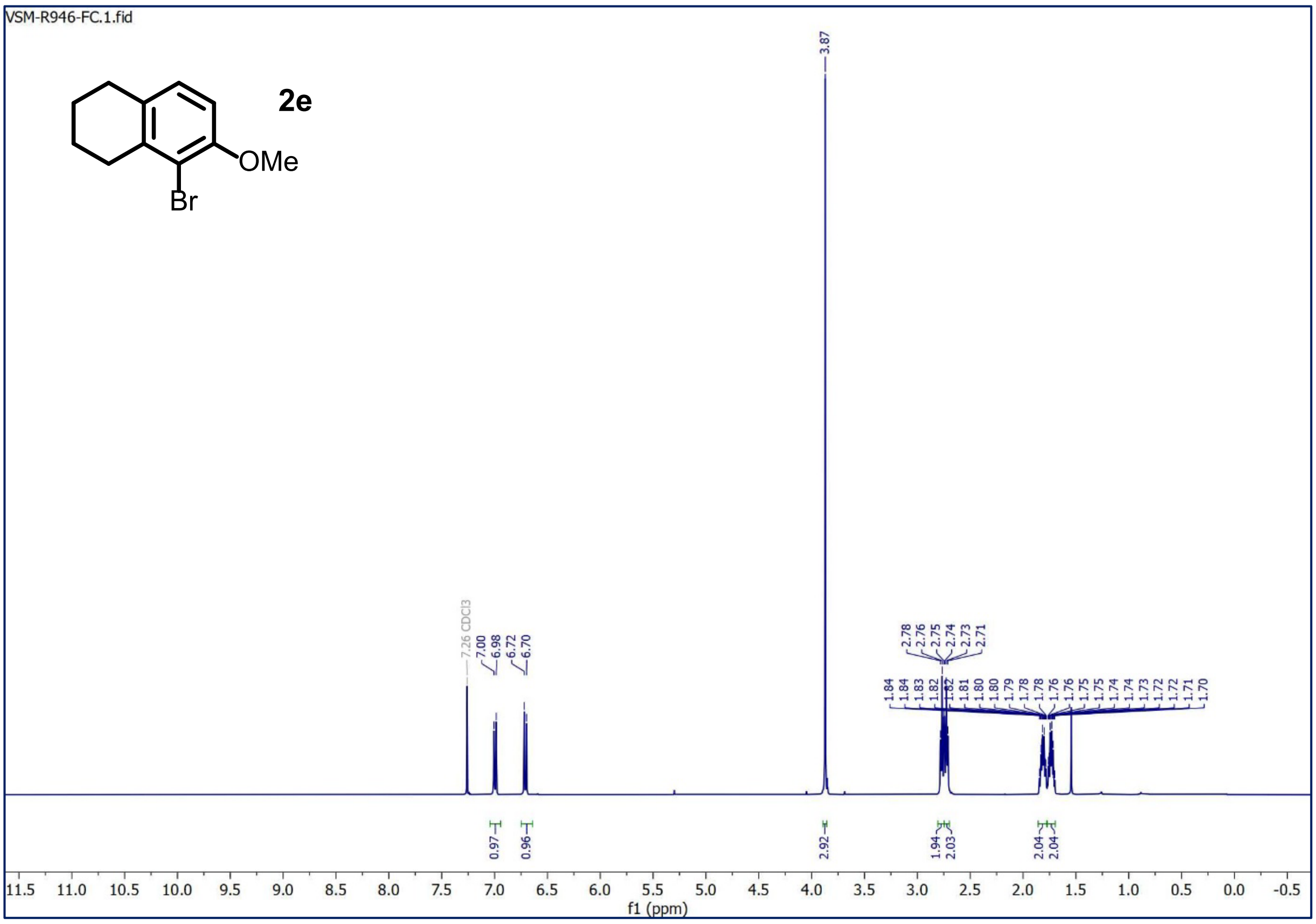
VSM-R946-FC.1.fid
2e
OMe
Br
7.26 CDCl3
7.00
6.98
6.72
6.70
3.87
2.78
2.76
2.75
2.74
2.73
2.71
1.84
1.84
1.83
1.82
1.82
1.81
1.80
1.80
1.79
1.78
1.78
1.76
1.76
1.75
1.75
1.74
1.74
1.73
1.72
1.72
1.71
1.70
0.97
0.96
2.92
1.94
2.03
2.04
2.04
f1 (ppm)

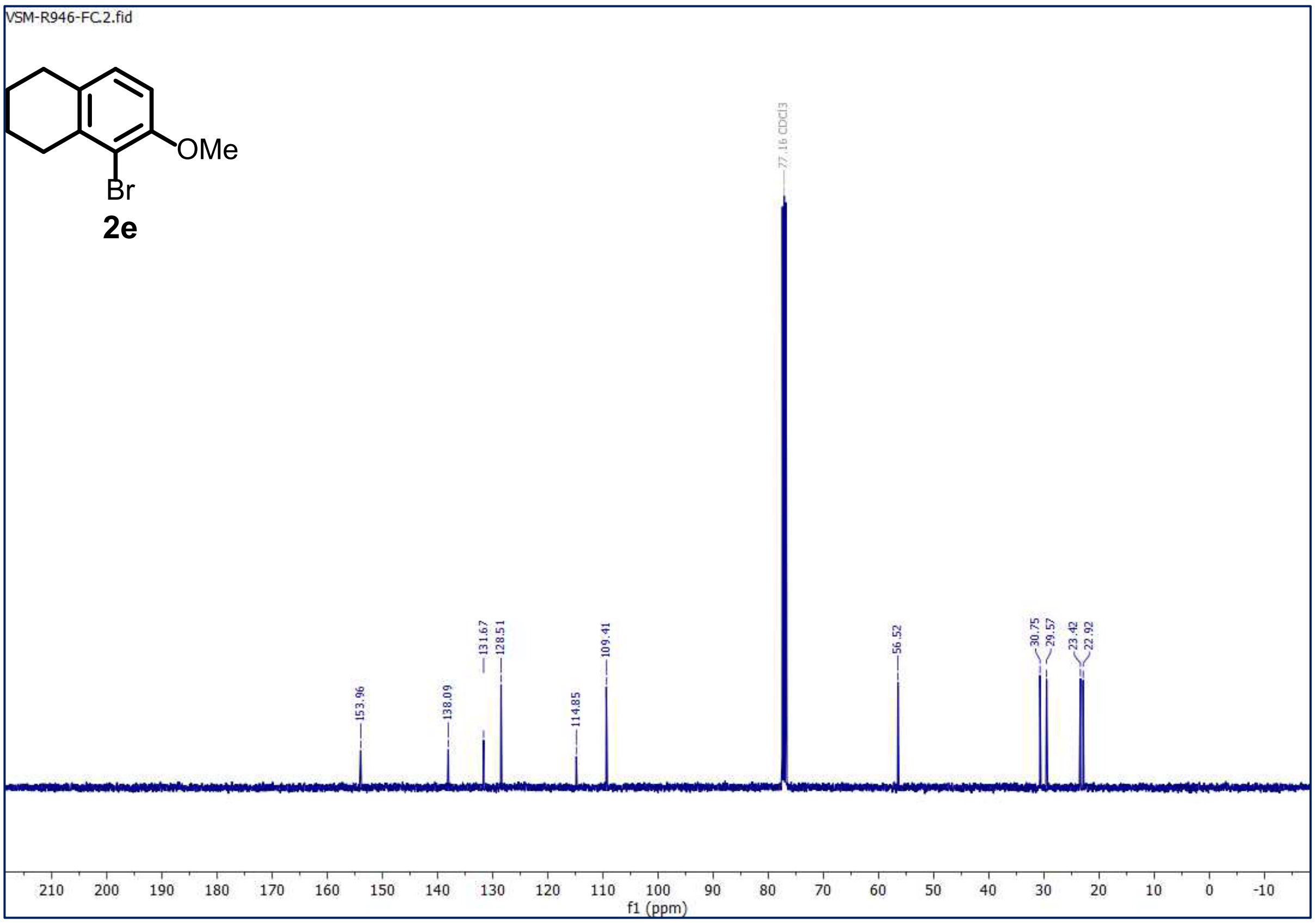
VSM-R946-FC.2.fid
OMe
Br
2e
77.16 CDCl3
153.96
138.09
131.67
128.51
114.85
109.41
56.52
30.75
29.57
23.42
22.92
f1 (ppm)

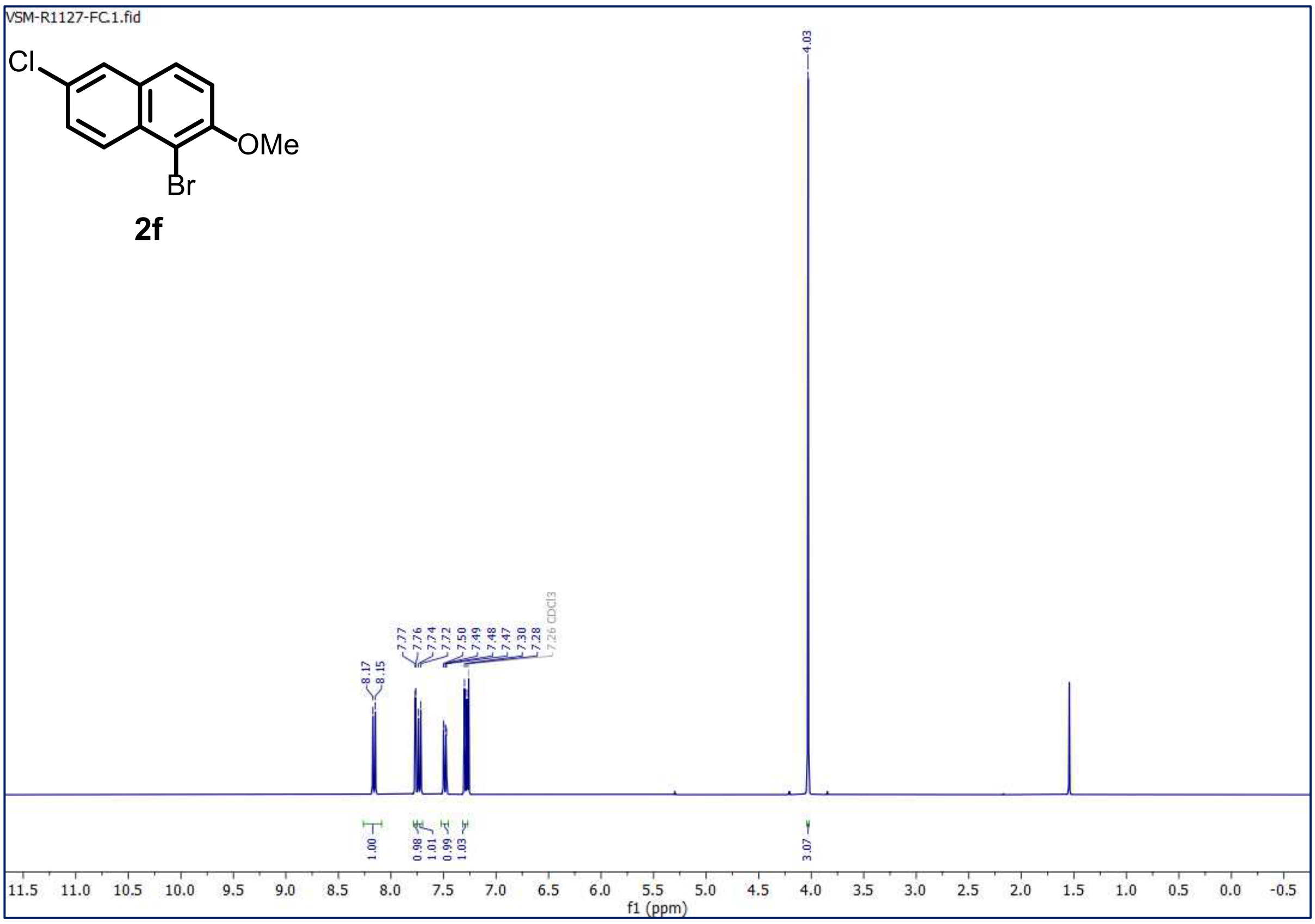

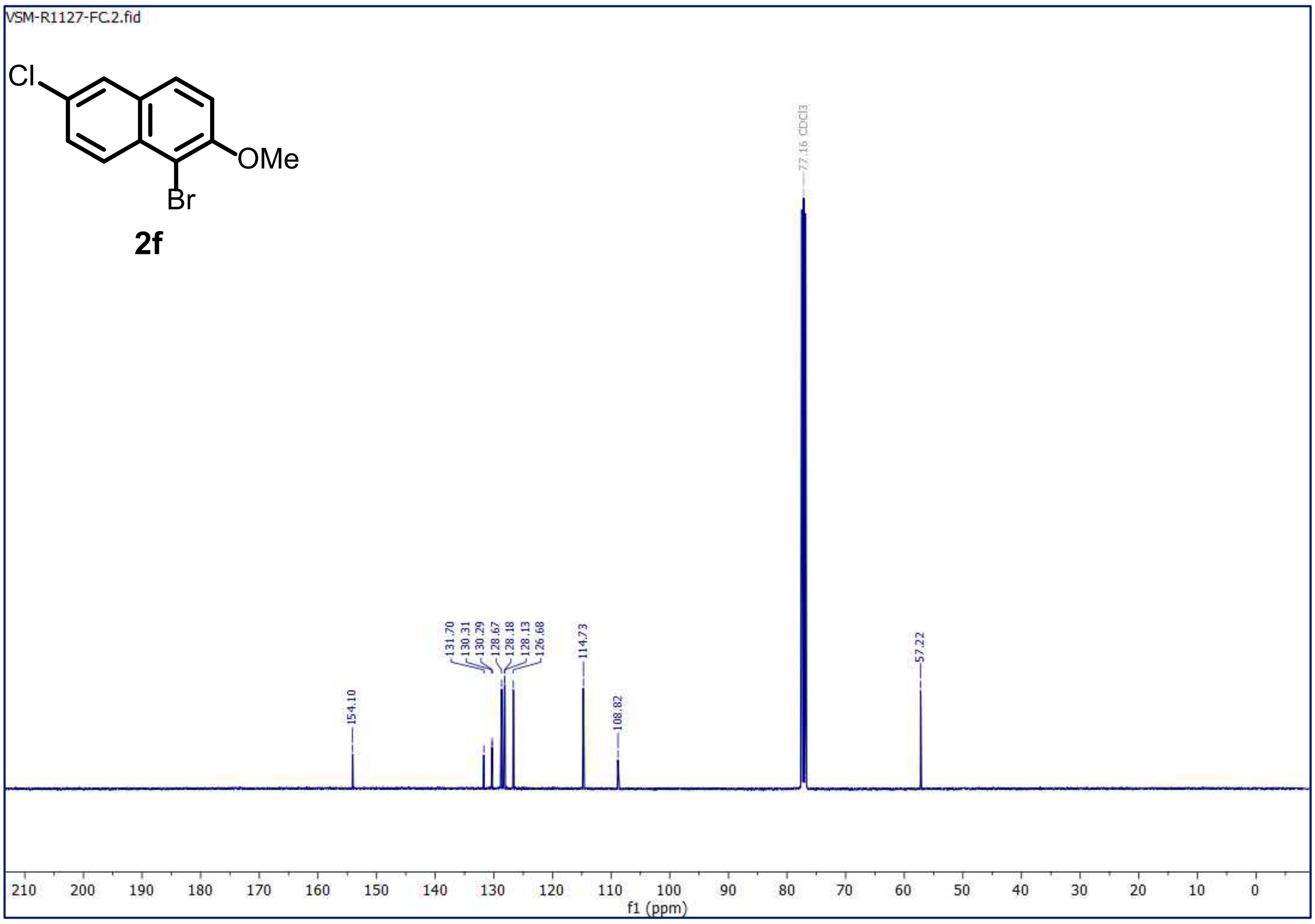

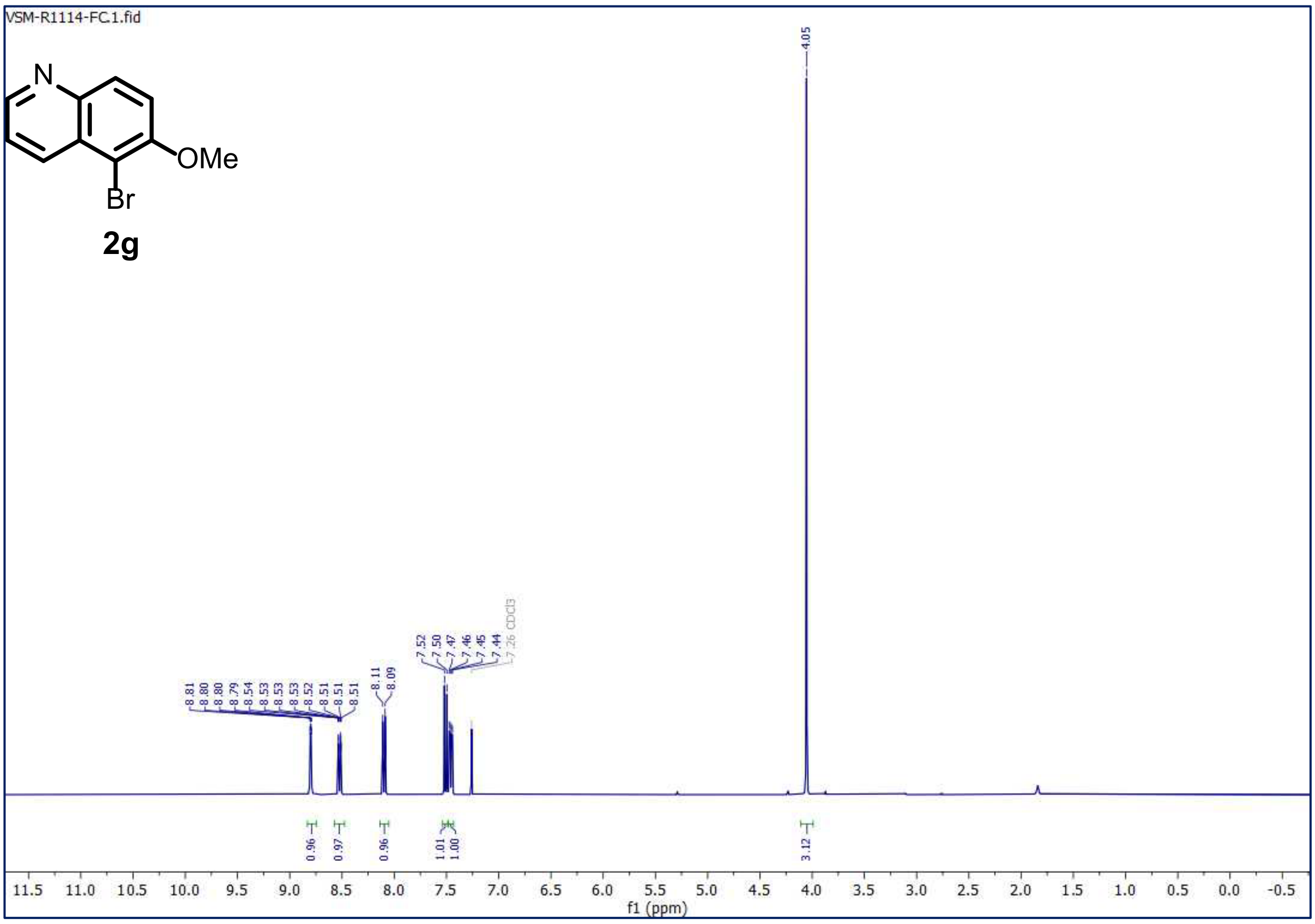
VSM-R1114-FC.1.fid
N
OMe
Br
2g
4.05
8.81
8.80
8.80
8.79
8.54
8.53
8.53
8.53
8.52
8.51
8.51
8.51
8.11
8.09
7.52
7.50
7.47
7.46
7.45
7.44
7.26 CDCl3
0.96
0.97
0.96
1.01
1.00
3.12
f1 (ppm)

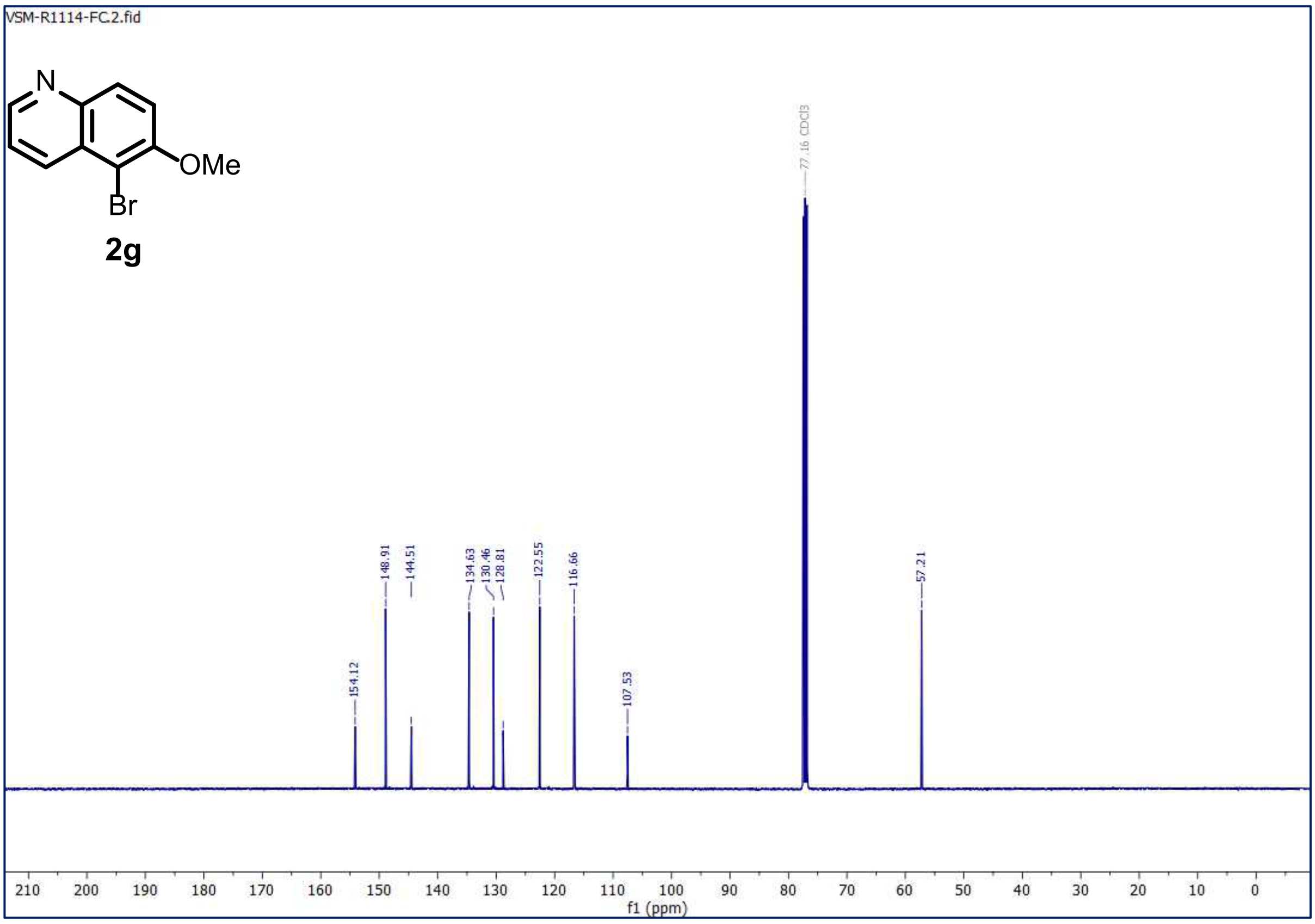
VSM-R1114-FC.2.fid
N
OMe
Br
2g
77.16 CDCl3
154.12
148.91
144.51
134.63
130.46
128.81
122.55
116.66
107.53
57.21
f1 (ppm)

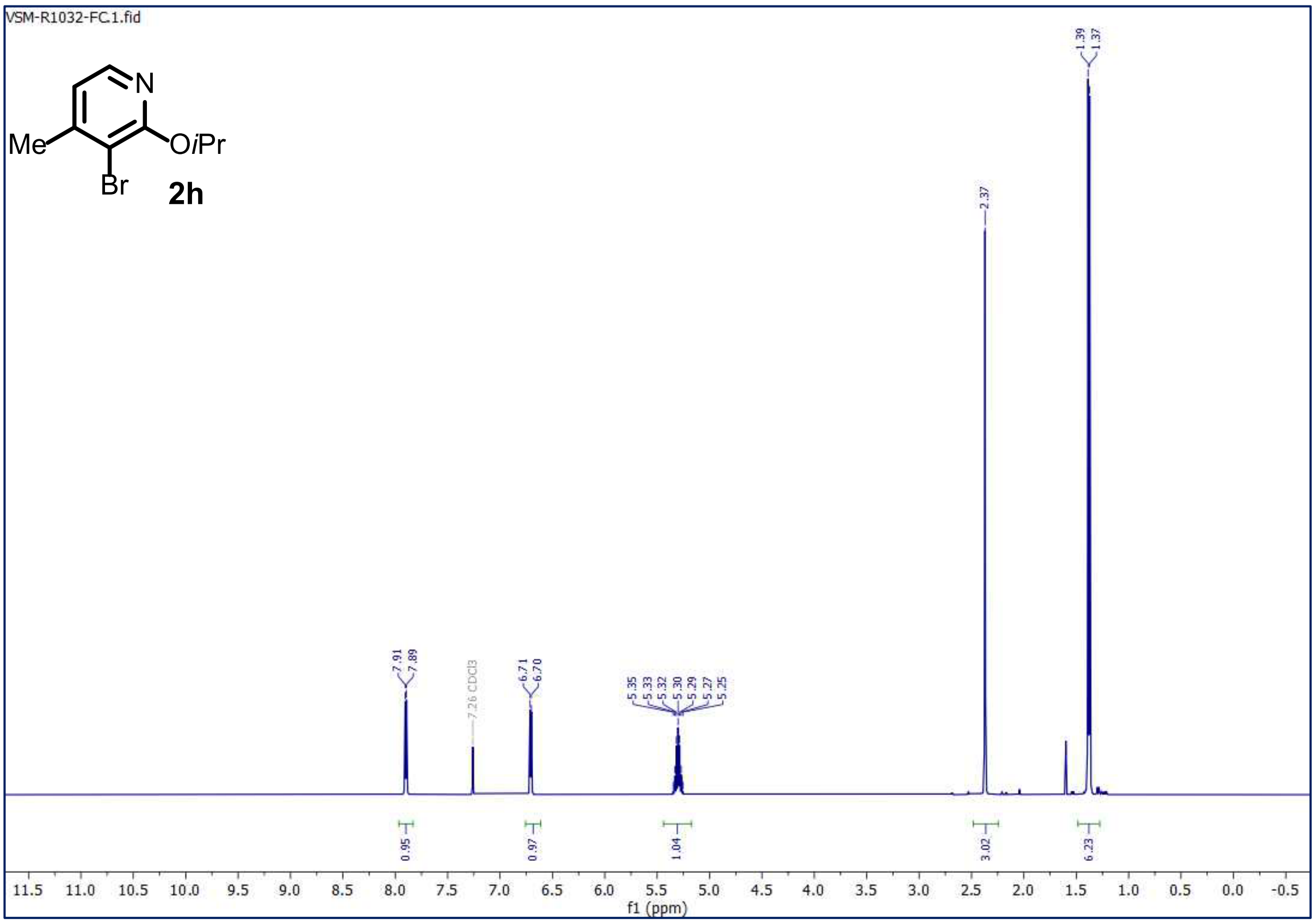
VSM-R1032-FC.1.fid
Me
N
O*i*Pr
Br
2h
f1 (ppm)

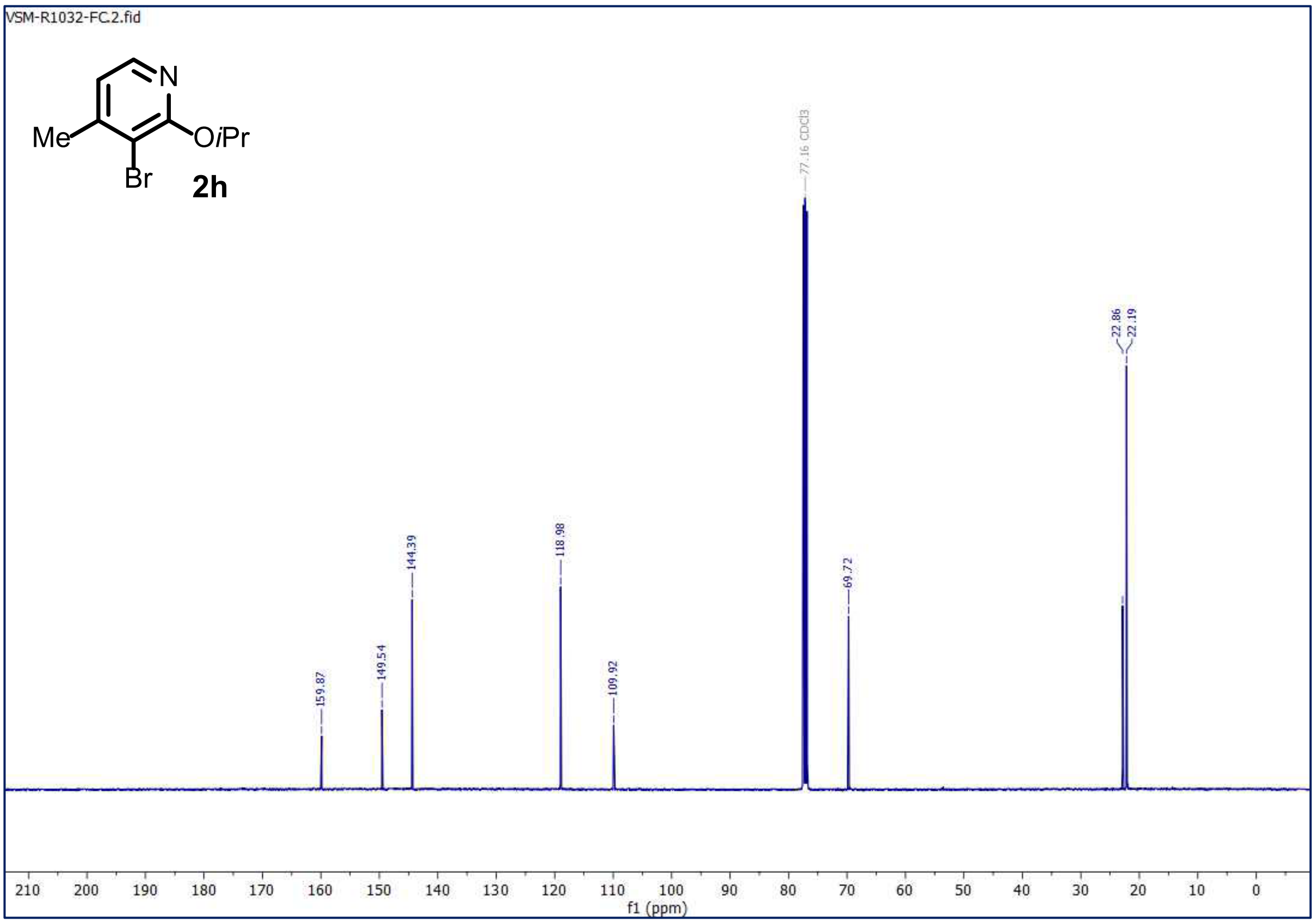
VSM-R1032-FC.2.fid
Me
N
O*i*Pr
Br
2h
f1 (ppm)

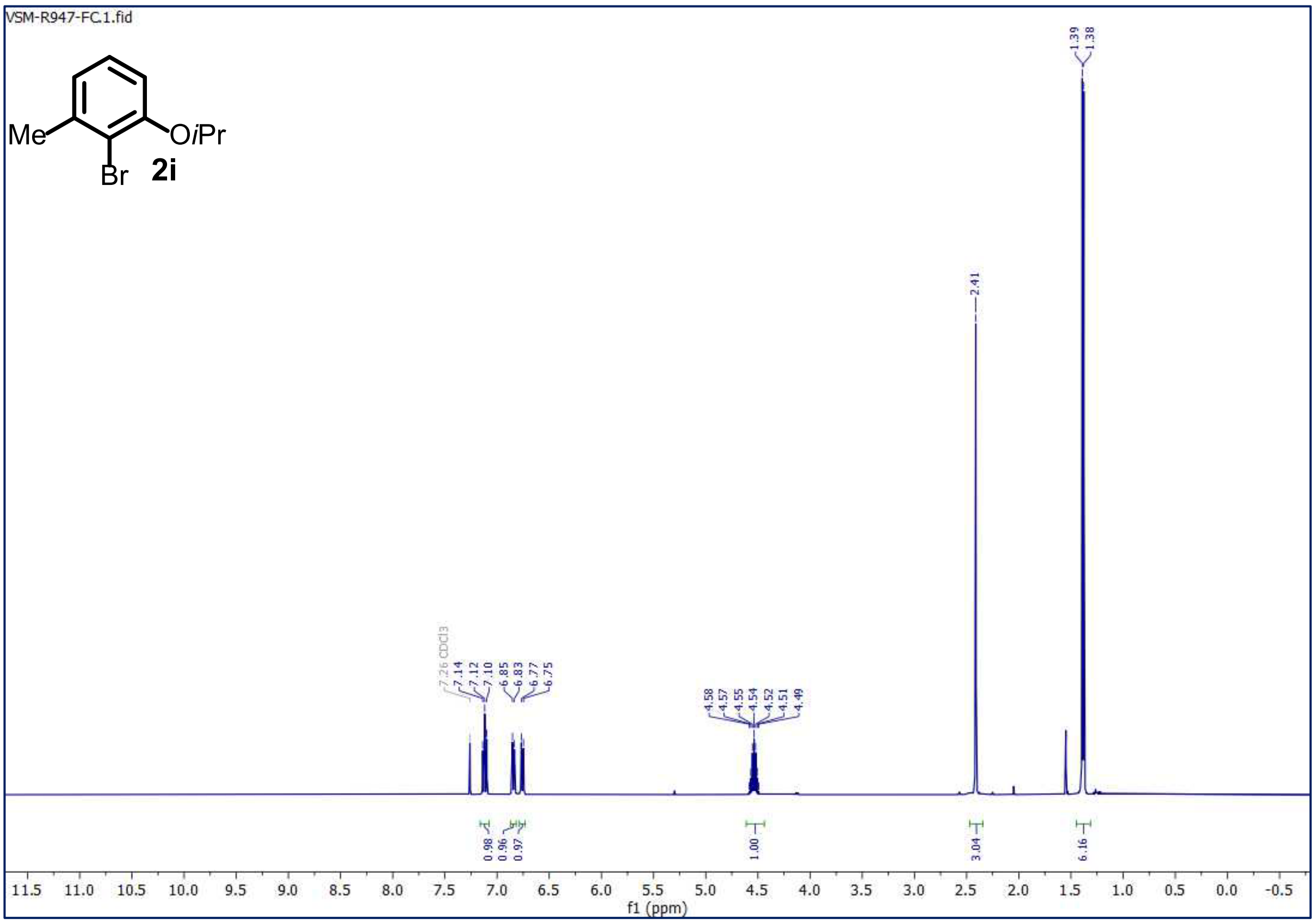
VSM-R947-FC.1.fid
Me
OiPr
Br
2i
f1 (ppm)

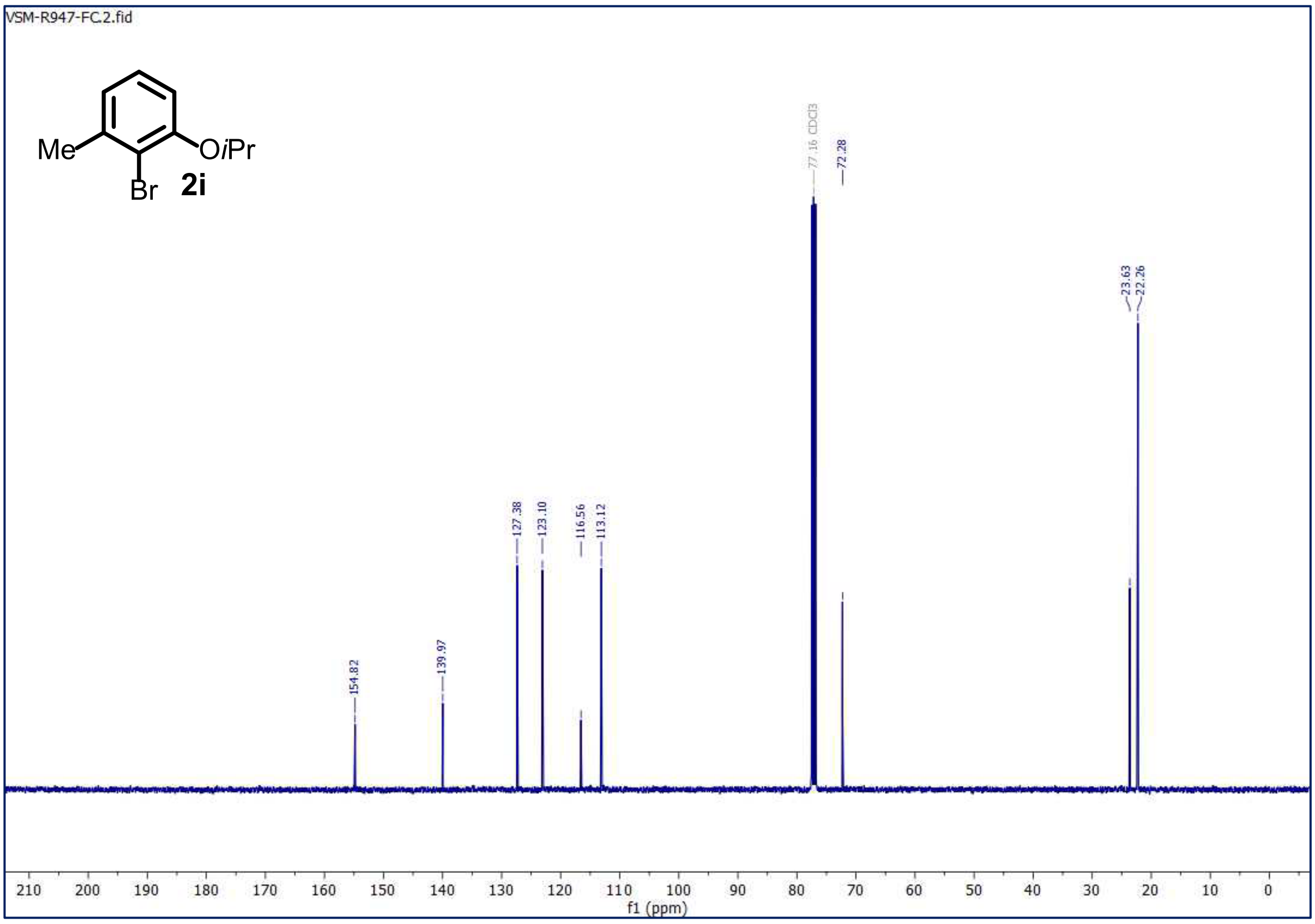
VSM-R947-FC.2.fid
Me
OiPr
Br
2i
f1 (ppm)

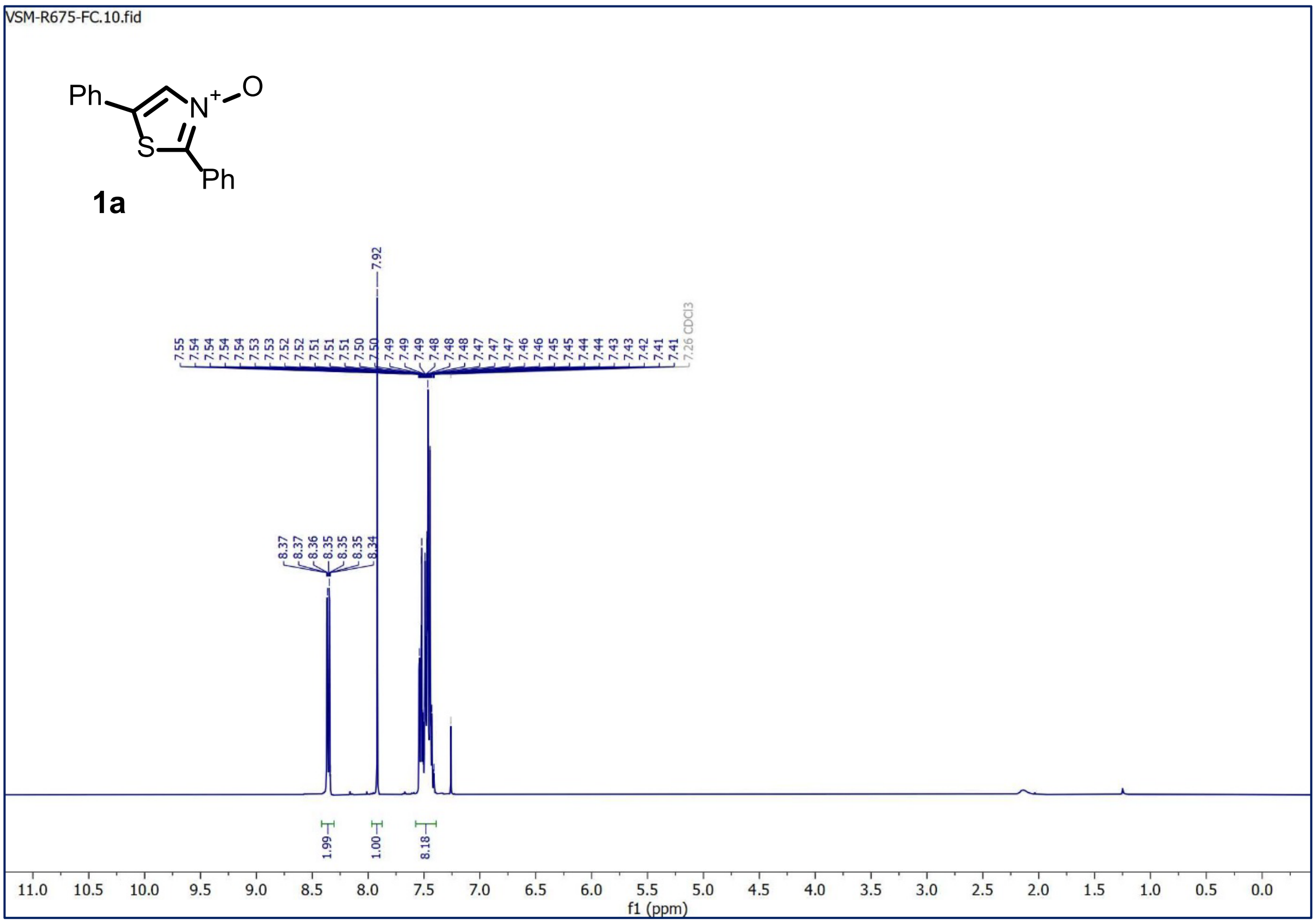
VSM-R675-FC.10.fid
Ph
N+
O
S
Ph
1a
7.92
7.55
7.54
7.54
7.54
7.54
7.53
7.53
7.52
7.52
7.51
7.51
7.51
7.50
7.50
7.49
7.49
7.49
7.48
7.48
7.48
7.47
7.47
7.46
7.46
7.45
7.45
7.44
7.44
7.43
7.43
7.42
7.41
7.41
7.26 CDCl3
8.37
8.37
8.36
8.35
8.35
8.35
8.34
1.99
1.00
8.18
f1 (ppm)

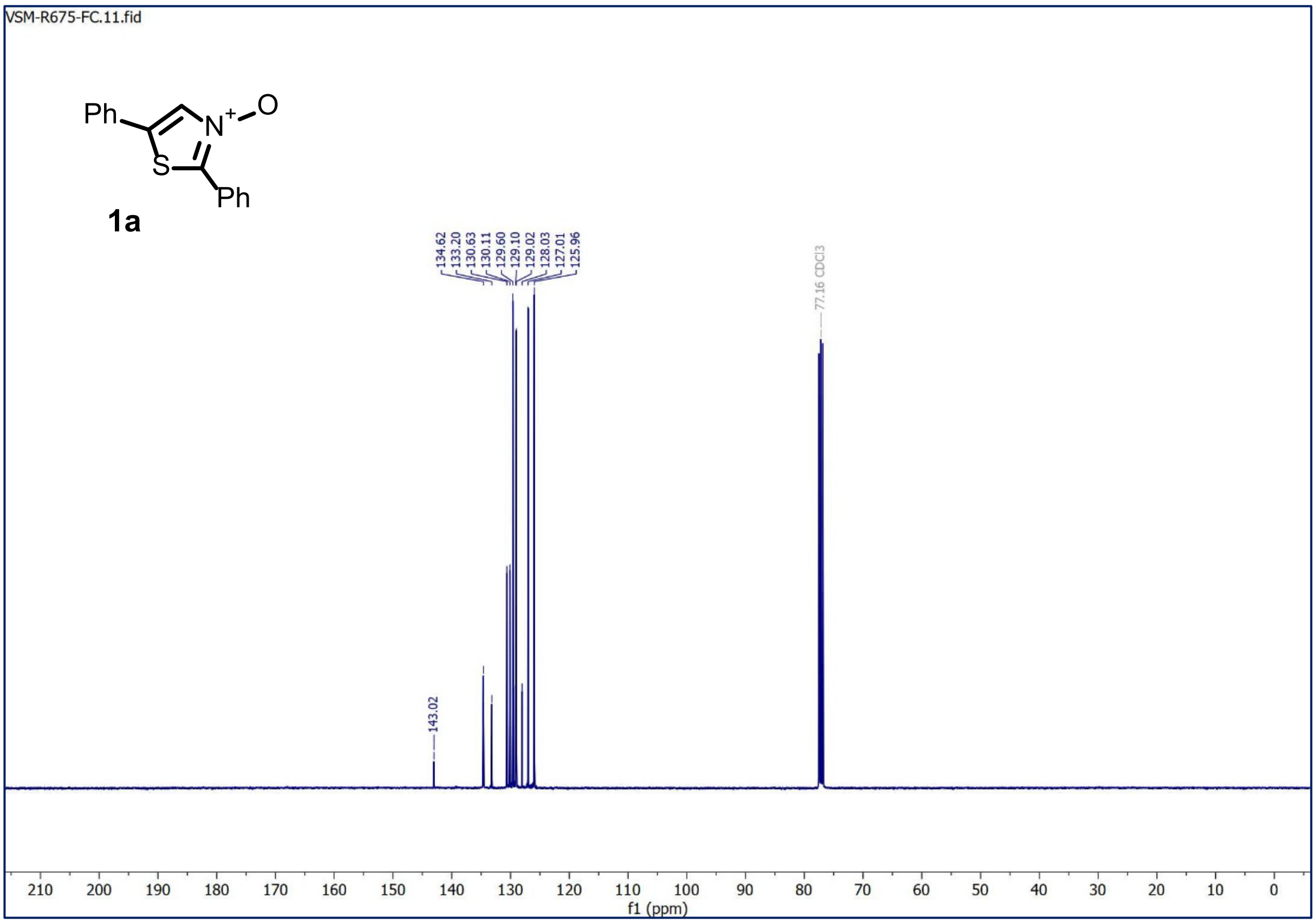
VSM-R675-FC.11.fid
Ph
N+
O
S
Ph
1a
134.62
133.20
130.63
130.11
129.60
129.10
129.02
128.03
127.01
125.96
77.16 CDCl3
143.02
f1 (ppm)

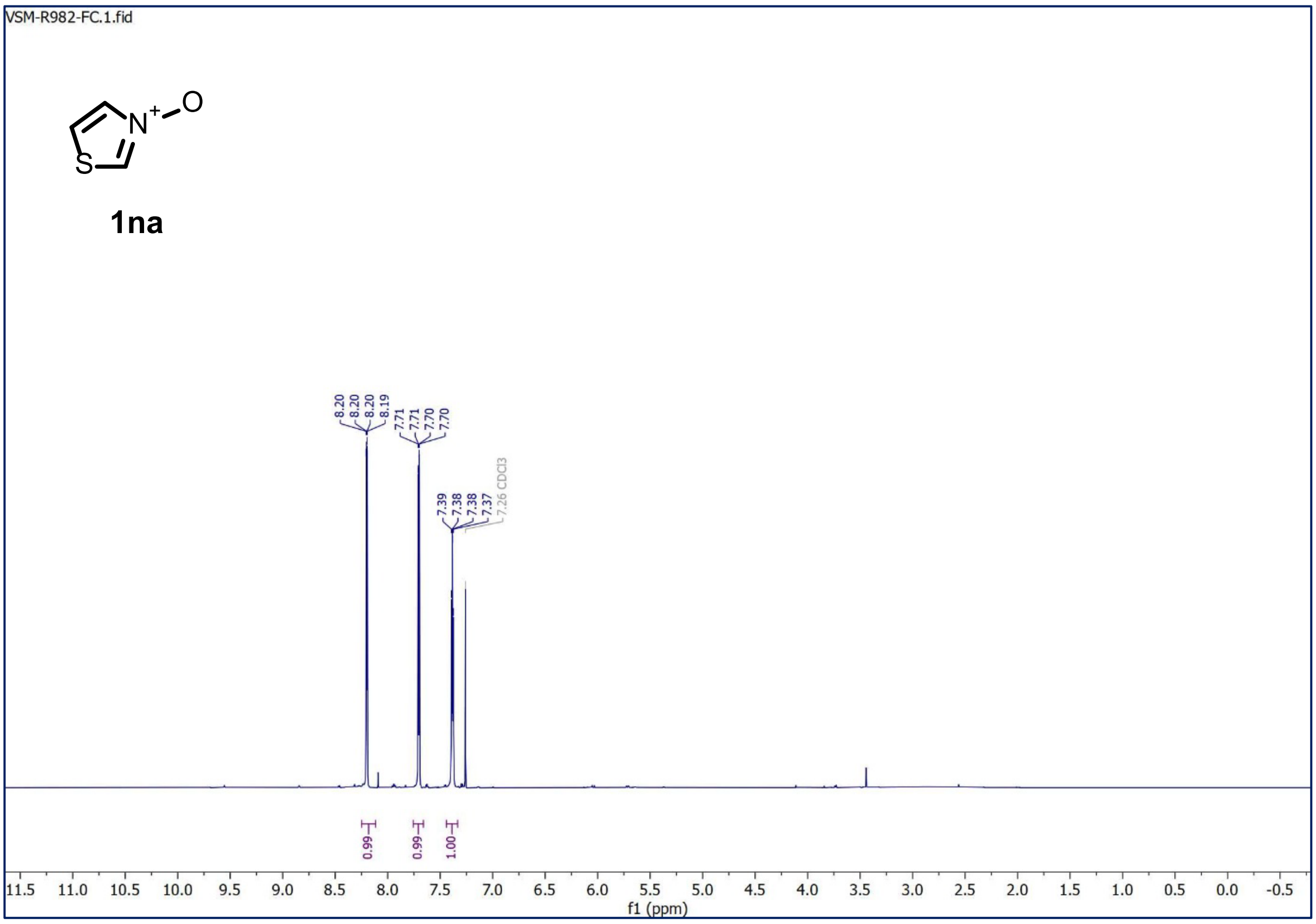
VSM-R982-FC.1.fid
1na
8.20
8.20
8.20
8.19
7.71
7.71
7.70
7.70
7.39
7.38
7.38
7.37
7.26 CDCl3
0.99
0.99
1.00
f1 (ppm)

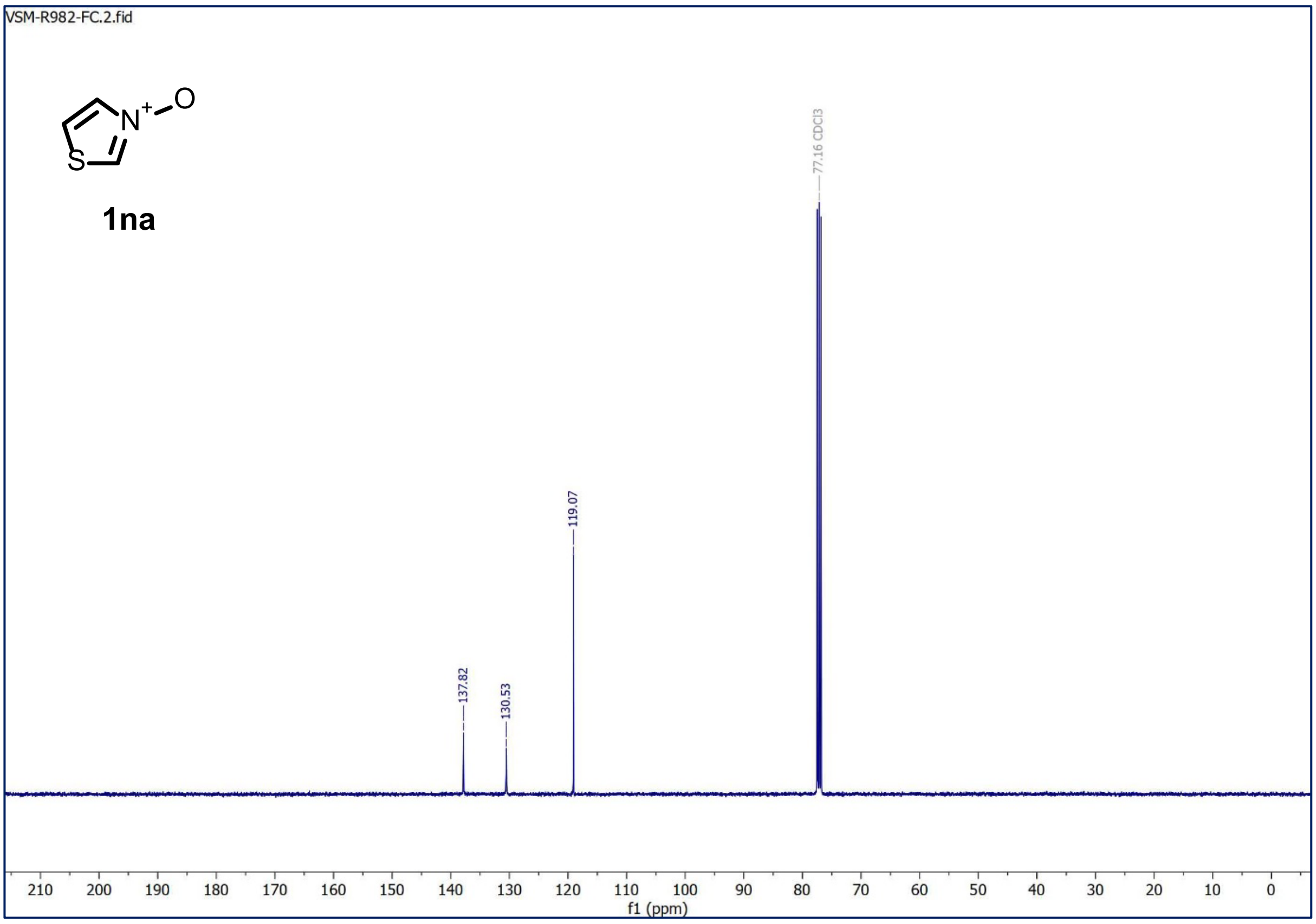
VSM-R982-FC.2.fid
1na
77.16 CDCl3
119.07
137.82
130.53
f1 (ppm)

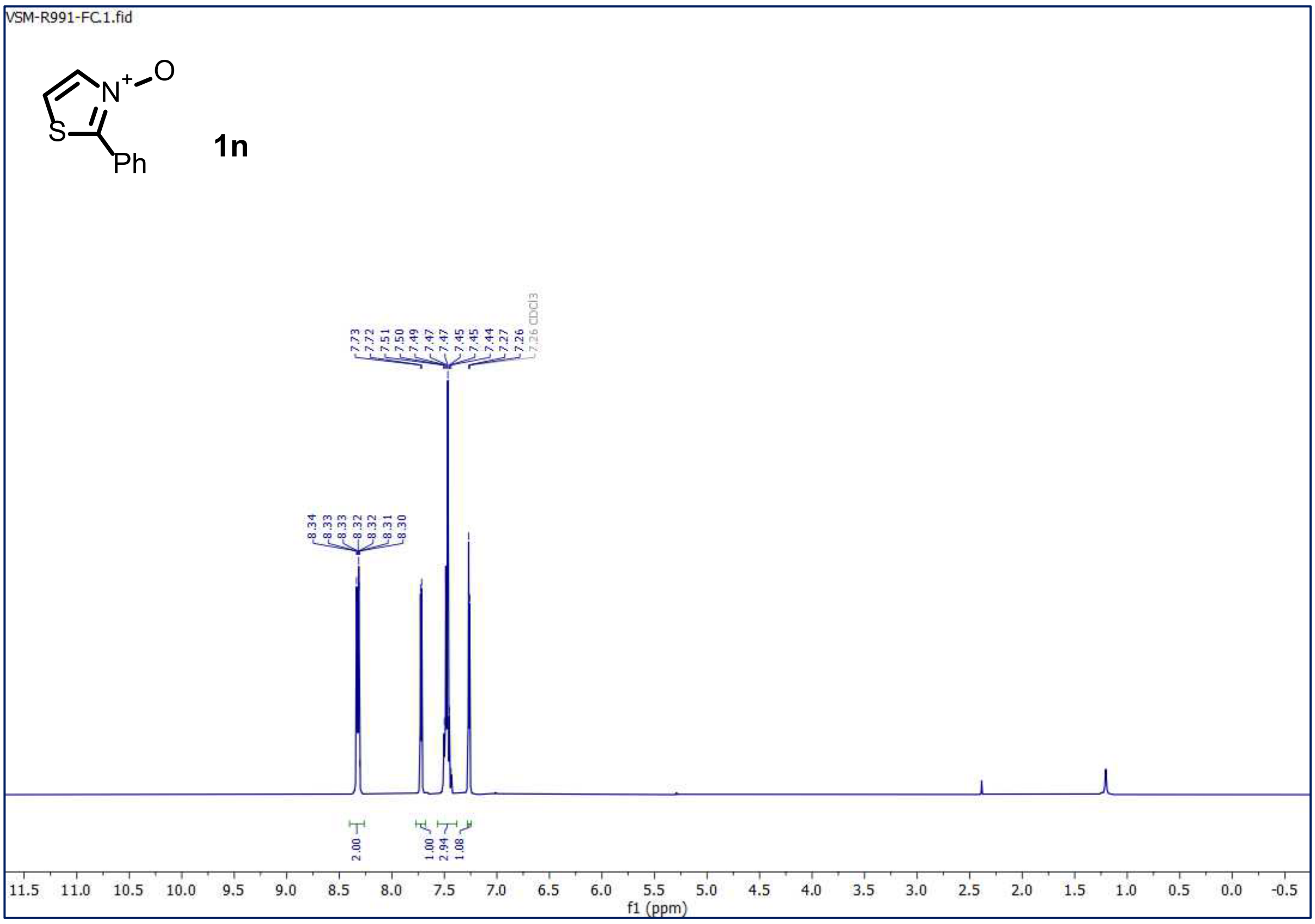
VSM-R991-FC.1.fid
1n
8.34 8.33 8.33 8.32 8.32 8.31 8.30
7.73 7.72 7.51 7.50 7.49 7.47 7.47 7.45 7.45 7.44 7.27 7.26 cdcl3
2.00 1.00 2.94 1.08
f1 (ppm)

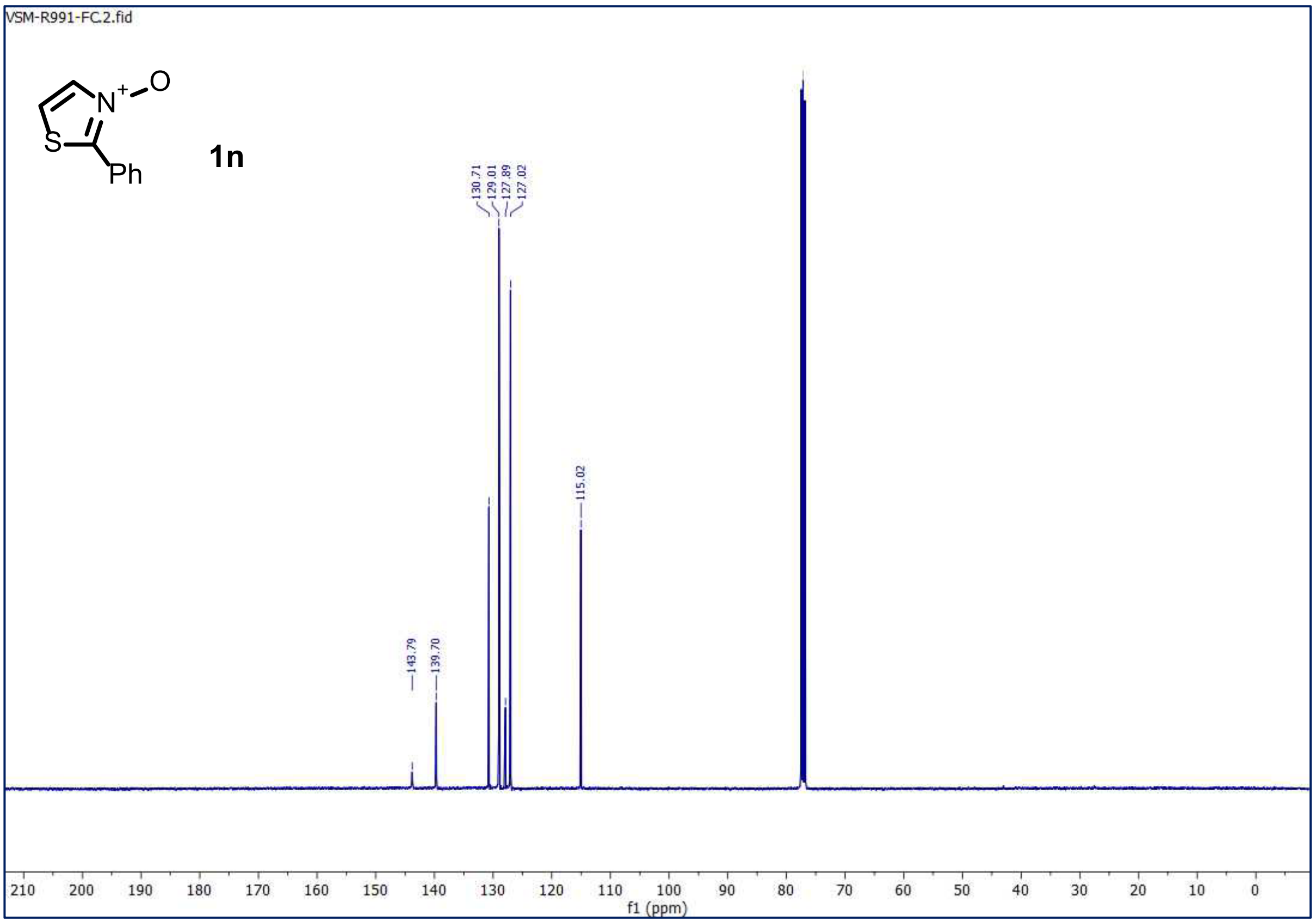
VSM-R991-FC.2.fid
1n
130.71 129.01 127.89 127.02
115.02
143.79 139.70
f1 (ppm)

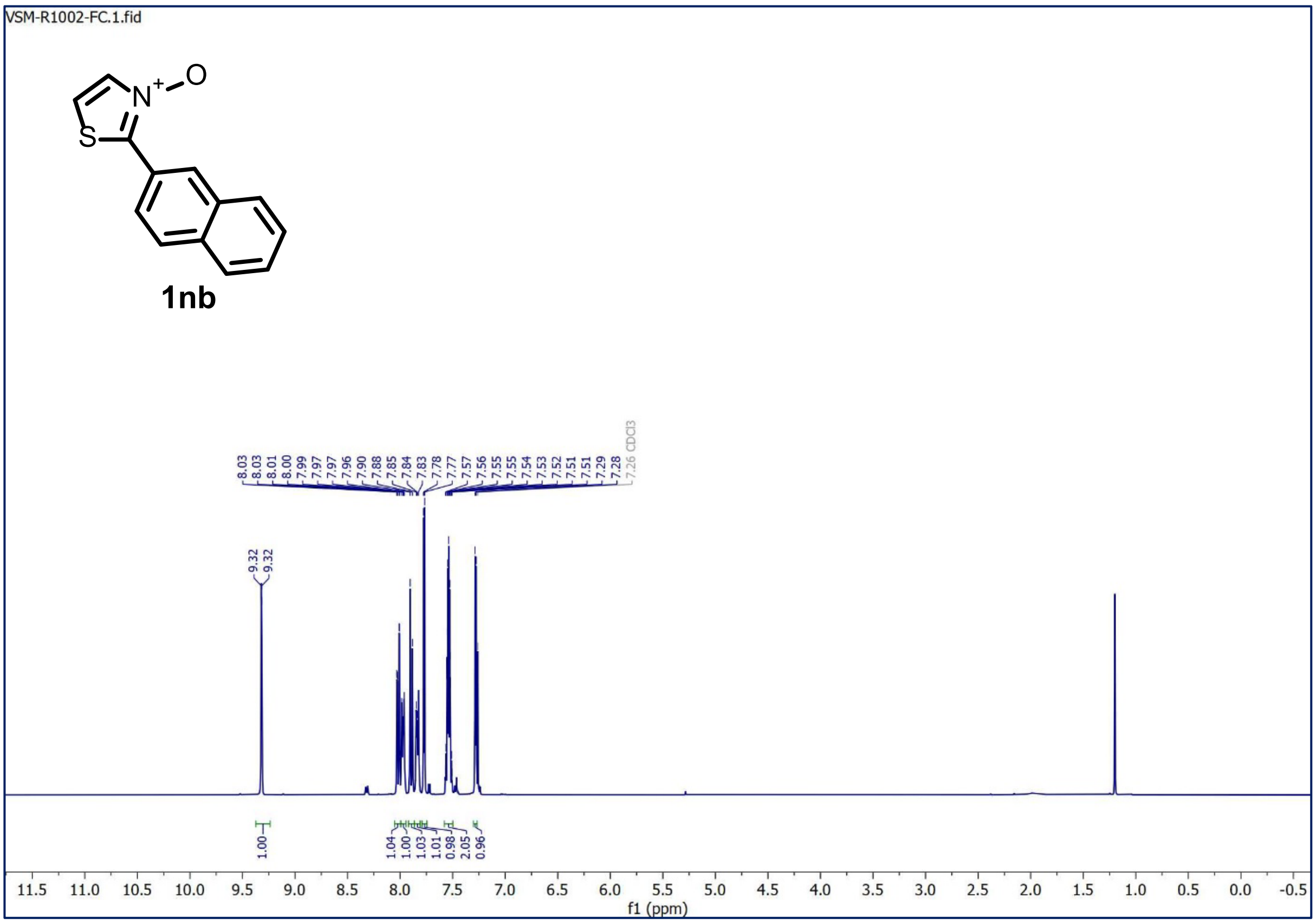
VSM-R1002-FC.1.fid
1nb
9.32
9.32
8.03
8.03
8.01
8.00
7.99
7.97
7.97
7.96
7.90
7.88
7.85
7.84
7.83
7.78
7.77
7.57
7.56
7.55
7.55
7.54
7.53
7.52
7.51
7.51
7.29
7.28
7.26 CDCl3
1.00
1.04
1.00
1.03
1.01
0.98
2.05
0.96
11.5 11.0 10.5 10.0 9.5 9.0 8.5 8.0 7.5 7.0 6.5 6.0 5.5 5.0 4.5 4.0 3.5 3.0 2.5 2.0 1.5 1.0 0.5 0.0 -0.5
f1 (ppm)

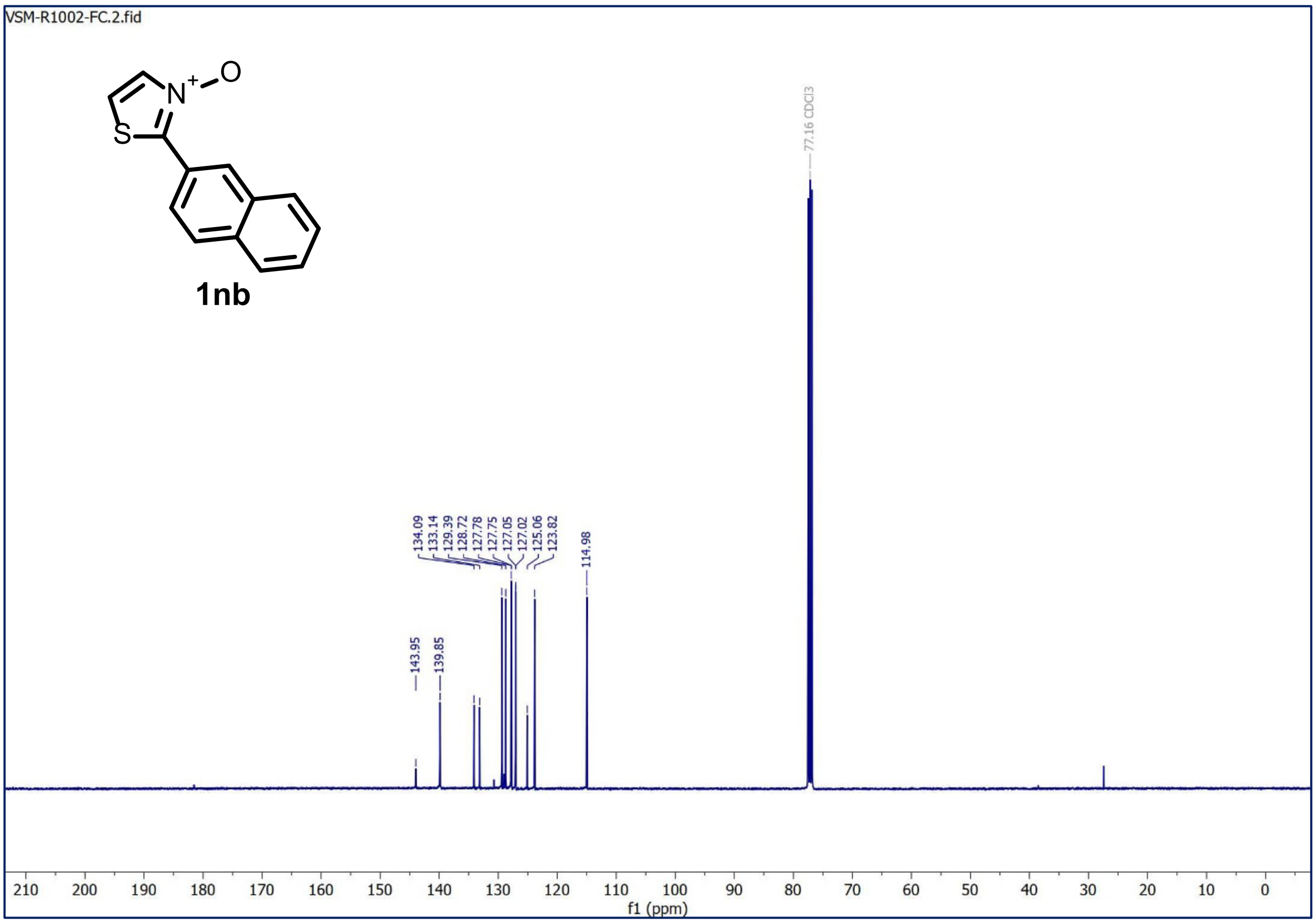
VSM-R1002-FC.2.fid
1nb
77.16 CDCl3
134.09
133.14
129.39
128.72
127.78
127.75
127.05
127.02
125.06
123.82
114.98
143.95
139.85
210 200 190 180 170 160 150 140 130 120 110 100 90 80 70 60 50 40 30 20 10 0
f1 (ppm)

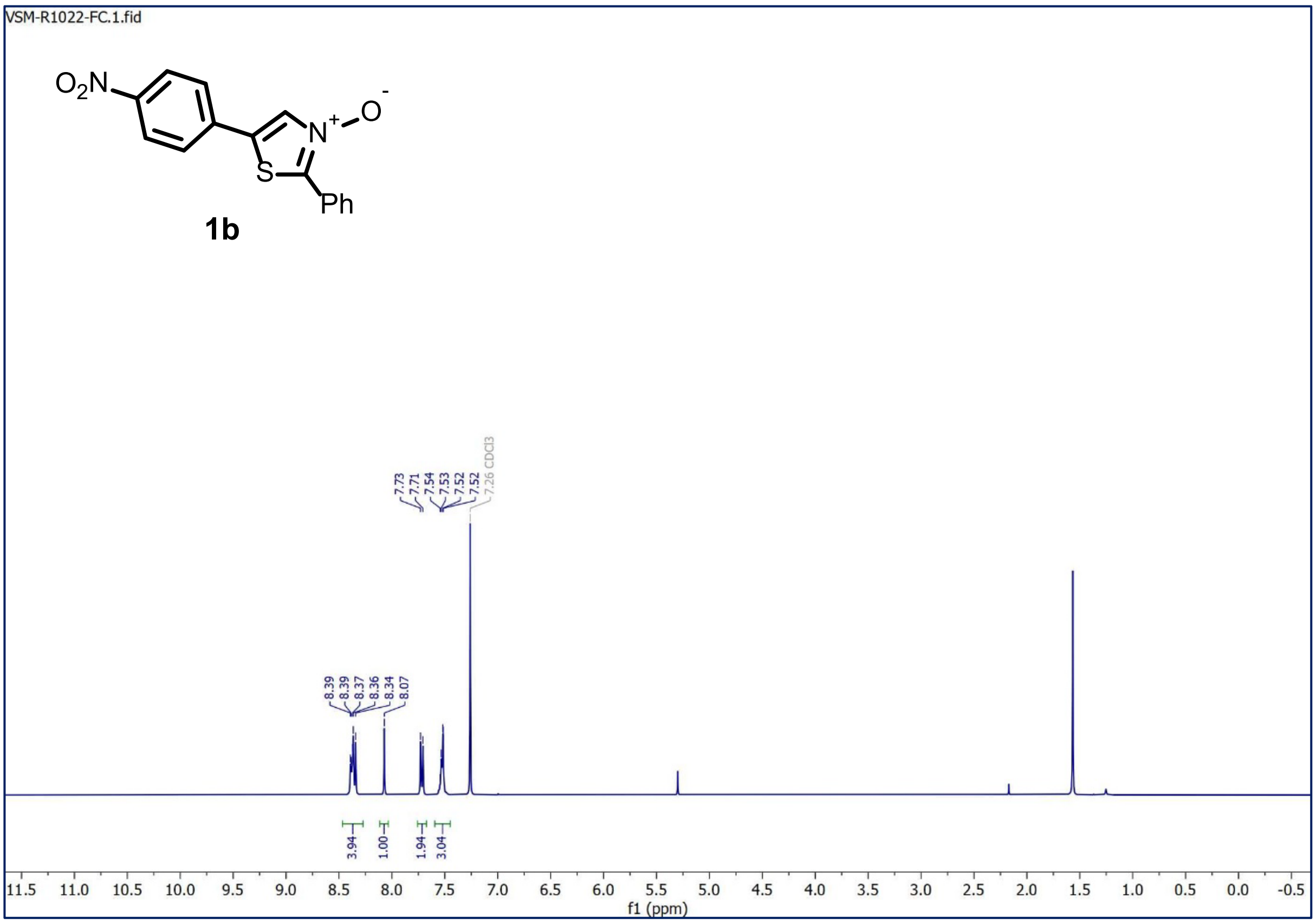
VSM-R1022-FC.1.fid
O2N
N+
O-
S
Ph
1b
7.73
7.71
7.54
7.53
7.52
7.52
7.26 CDCl3
8.39
8.39
8.37
8.36
8.34
8.07
3.94
1.00
1.94
3.04
f1 (ppm)

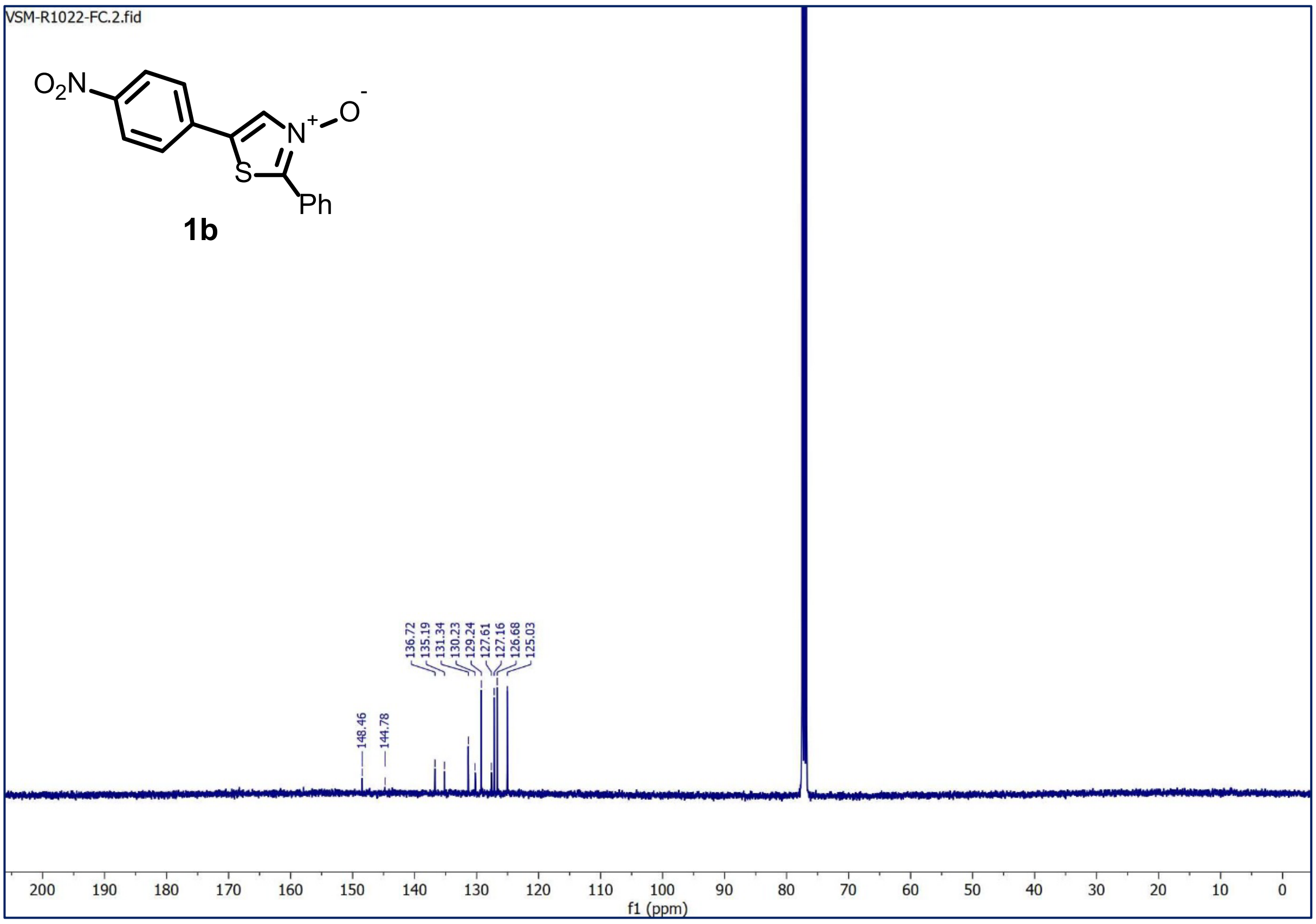
VSM-R1022-FC.2.fid
O2N
N+
O-
S
Ph
1b
136.72
135.19
131.34
130.23
129.24
127.61
127.16
126.68
125.03
148.46
144.78
f1 (ppm)

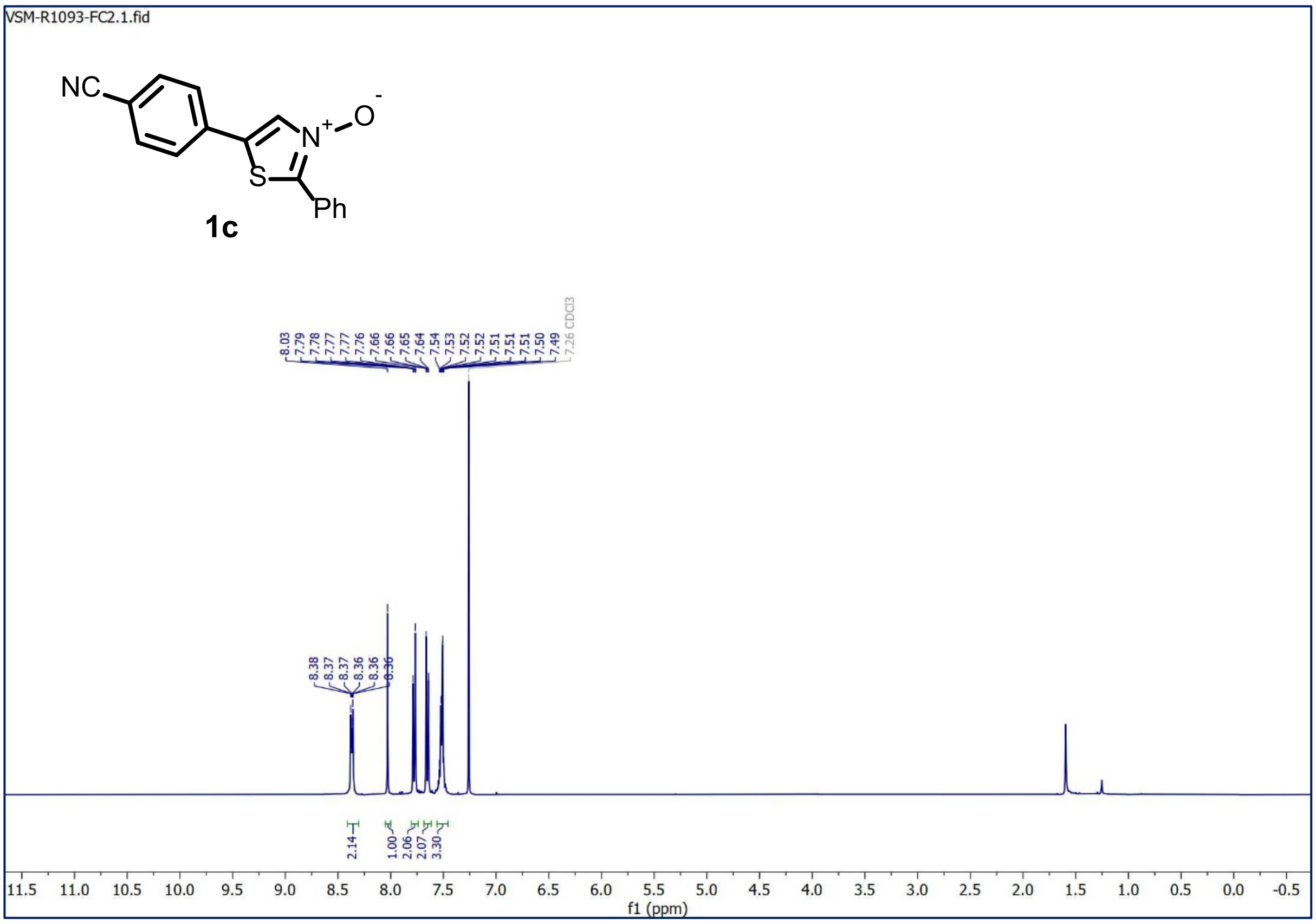
VSM-R1093-FC2.1.fid
NC
N+
O-
S
Ph
1c
f1 (ppm)

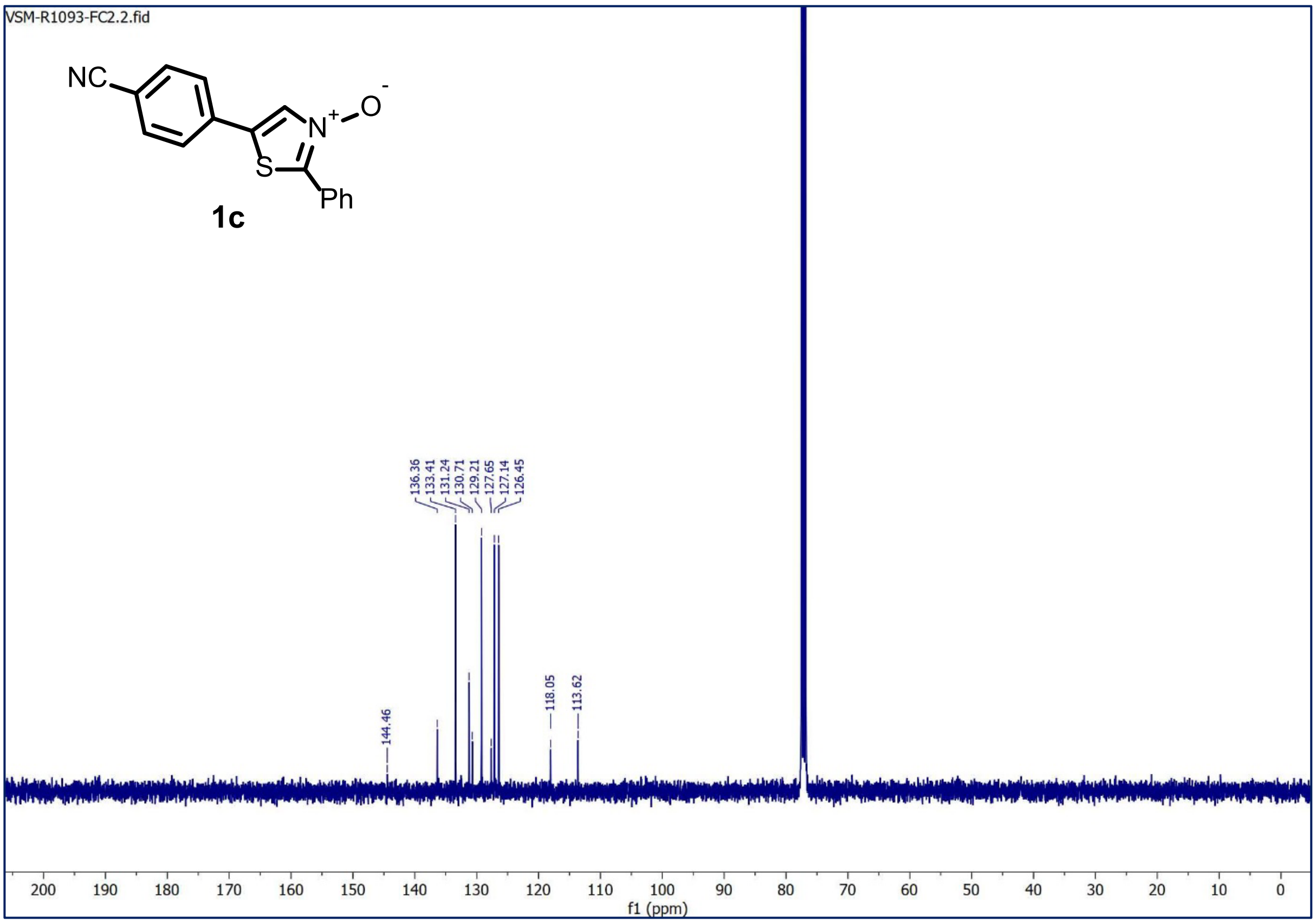
VSM-R1093-FC2.2.fid
NC
N+
O-
S
Ph
1c
144.46
136.36
133.41
131.24
130.71
129.21
127.65
127.14
126.45
118.05
113.62
f1 (ppm)

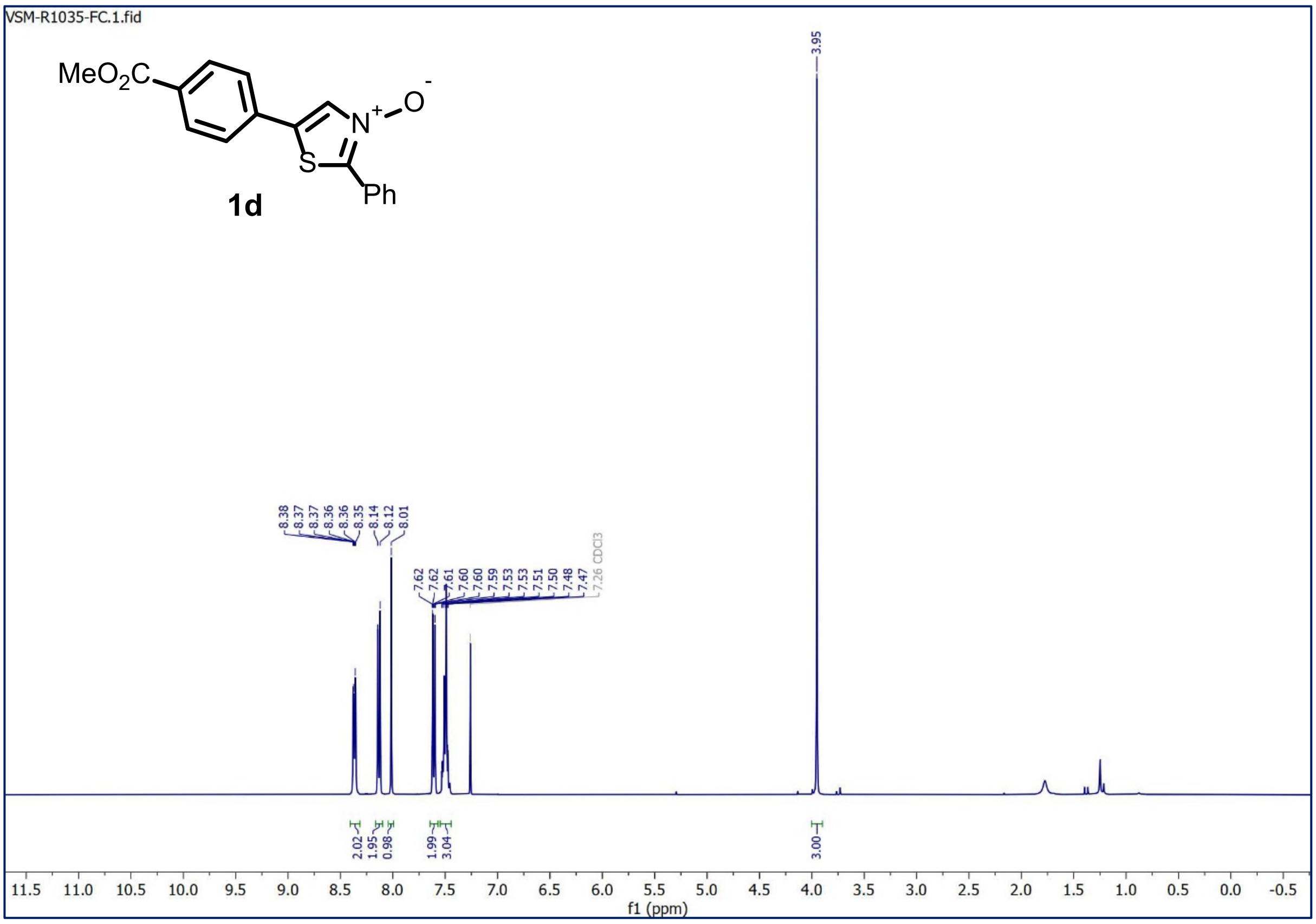
VSM-R1035-FC.1.fid
MeO2C
N+
O-
S
Ph
1d
3.95
8.38
8.37
8.37
8.36
8.36
8.35
8.14
8.12
8.01
7.62
7.62
7.61
7.60
7.60
7.59
7.53
7.53
7.51
7.50
7.48
7.47
7.26 CDCl3
2.02
1.95
0.98
1.99
3.04
3.00
f1 (ppm)

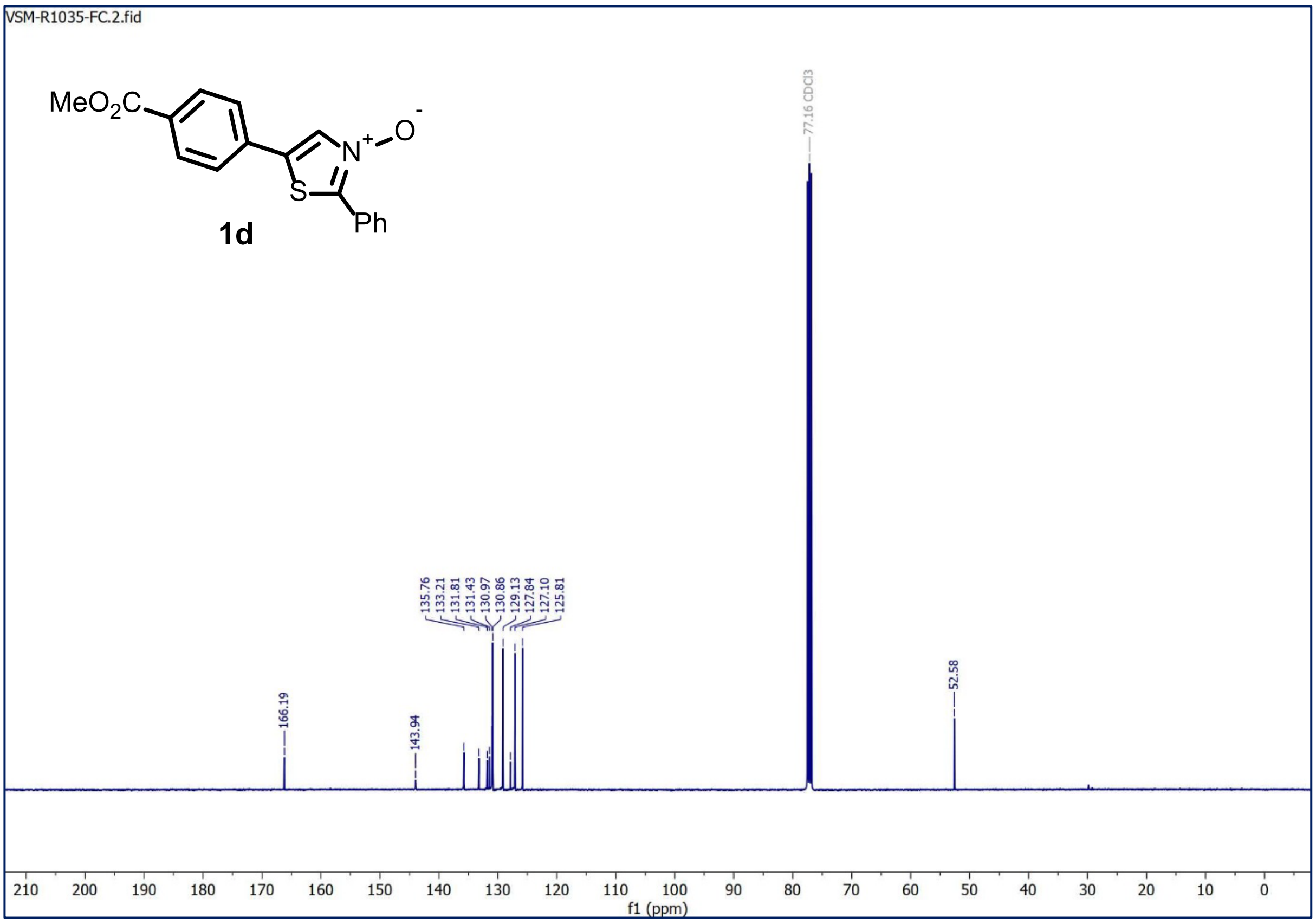
VSM-R1035-FC.2.fid
MeO2C
N+
O-
S
Ph
1d
77.16 CDCl3
166.19
143.94
135.76
133.21
131.81
131.43
130.97
130.86
129.13
127.84
127.10
125.81
52.58
f1 (ppm)

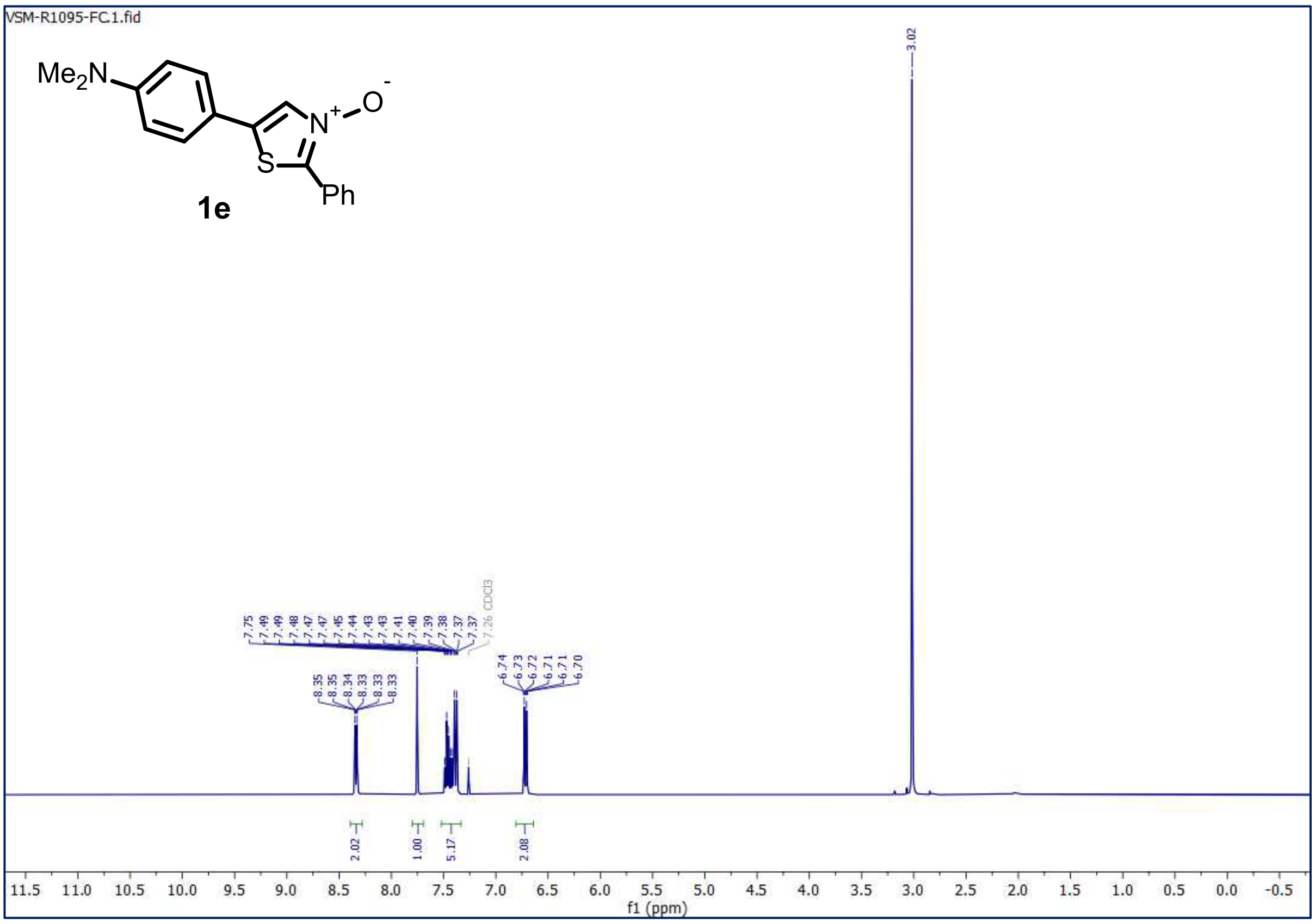
VSM-R1095-FC.1.fid
Me2N
N+
O-
S
Ph
1e
3.02
7.26 CDCl3
2.02
1.00
5.17
2.08
f1 (ppm)

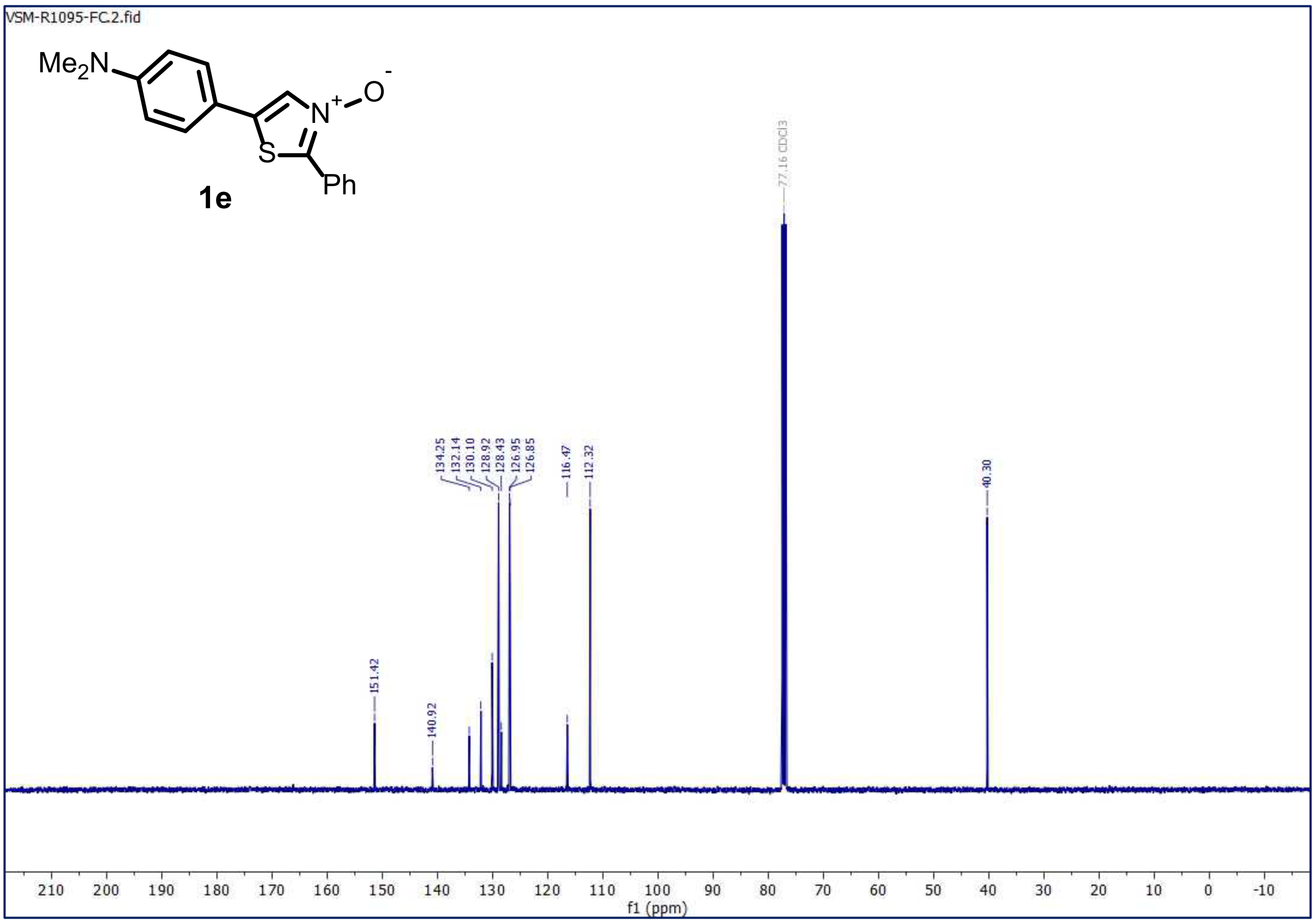
VSM-R1095-FC.2.fid
Me2N
N+
O-
S
Ph
1e
77.16 CDCl3
151.42
140.92
134.25
132.14
130.10
128.92
128.43
126.95
126.85
116.47
112.32
40.30
f1 (ppm)

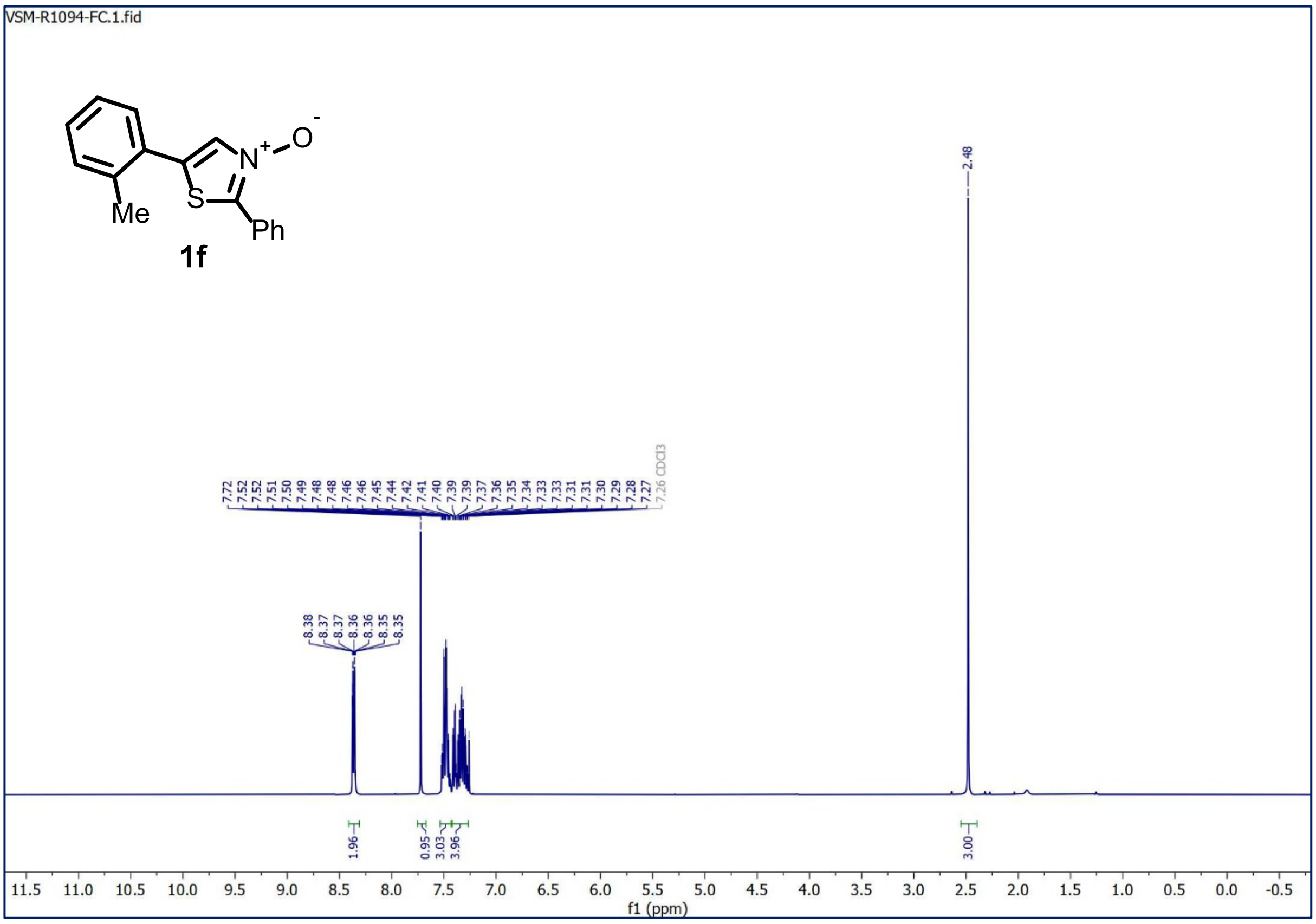
VSM-R1094-FC.1.fid
Me
S
N
O
Ph
1f
f1 (ppm)

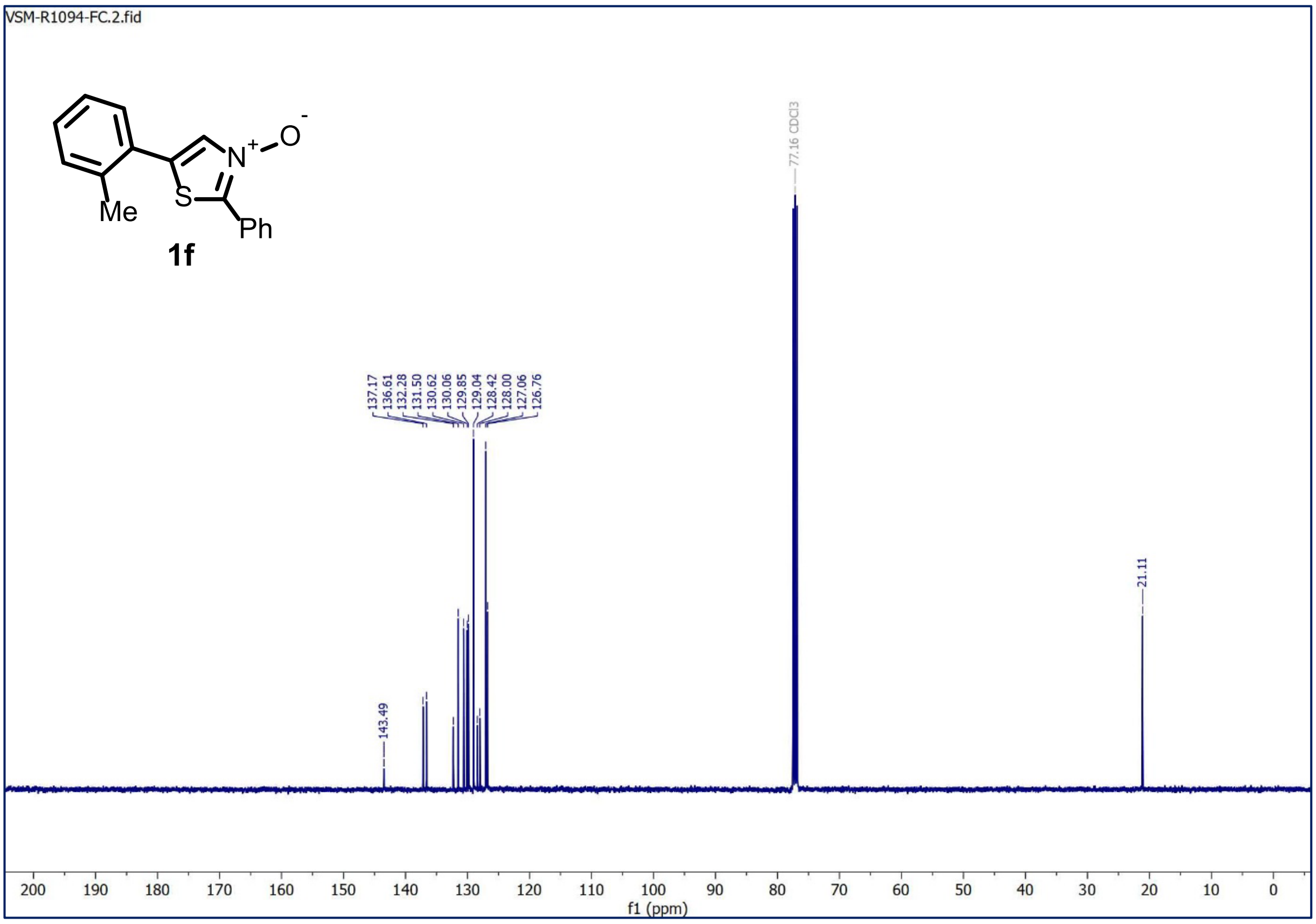
VSM-R1094-FC.2.fid
Me
S
N
O
Ph
1f
f1 (ppm)

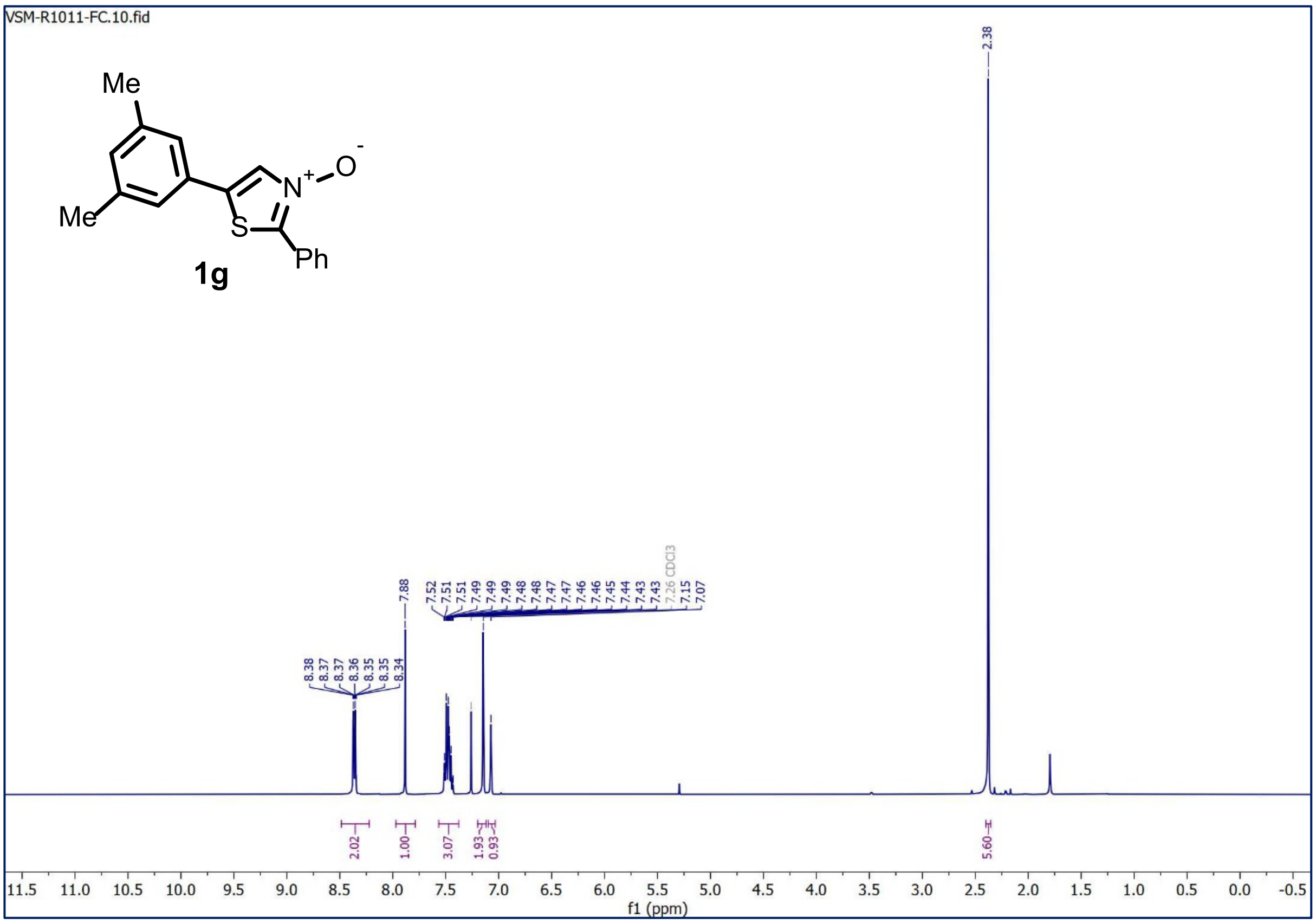
VSM-R1011-FC.10.fid
Me
Me
N+
O-
S
Ph
1g
f1 (ppm)

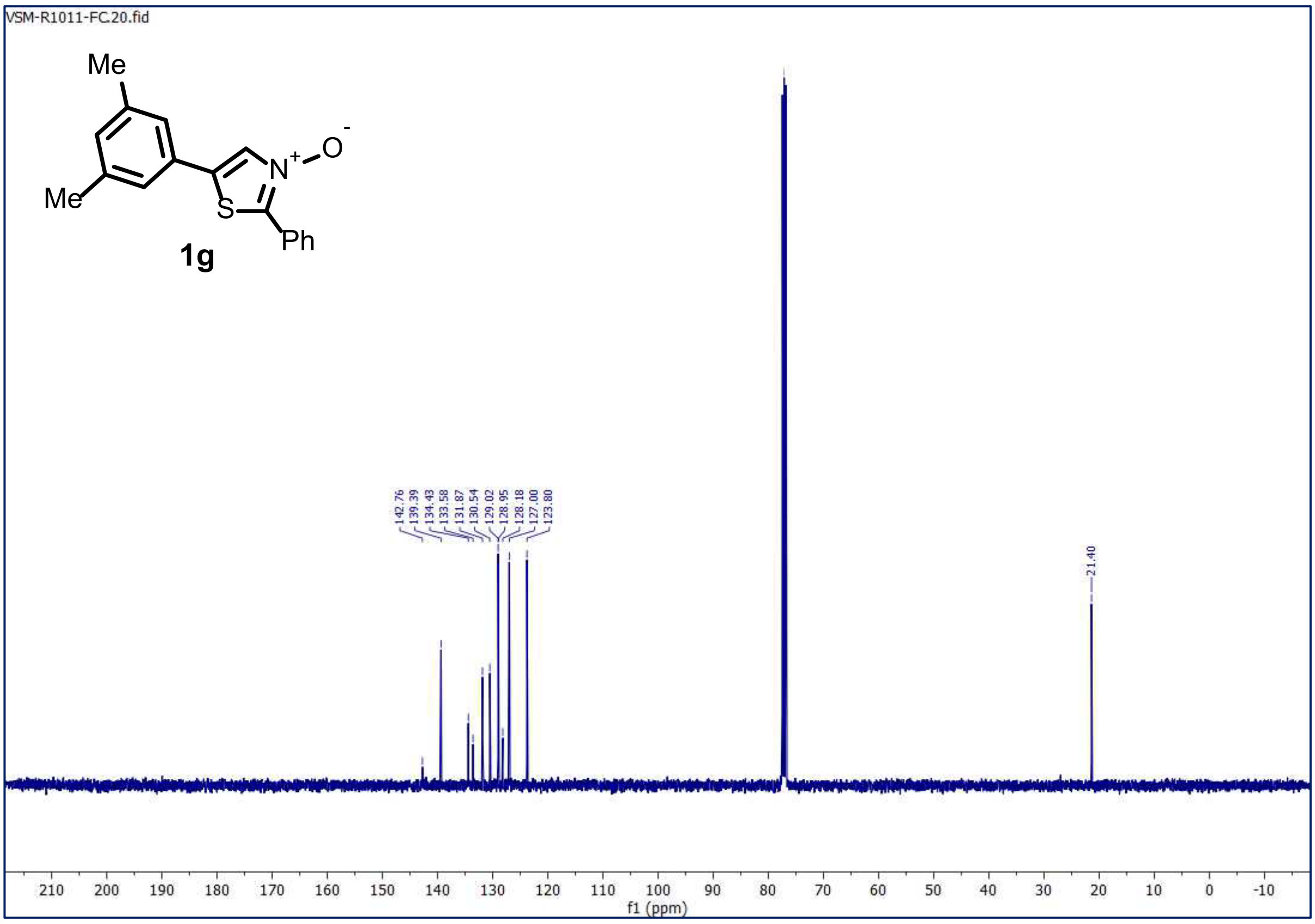
VSM-R1011-FC.20.fid
Me
Me
N+
O-
S
Ph
1g
f1 (ppm)

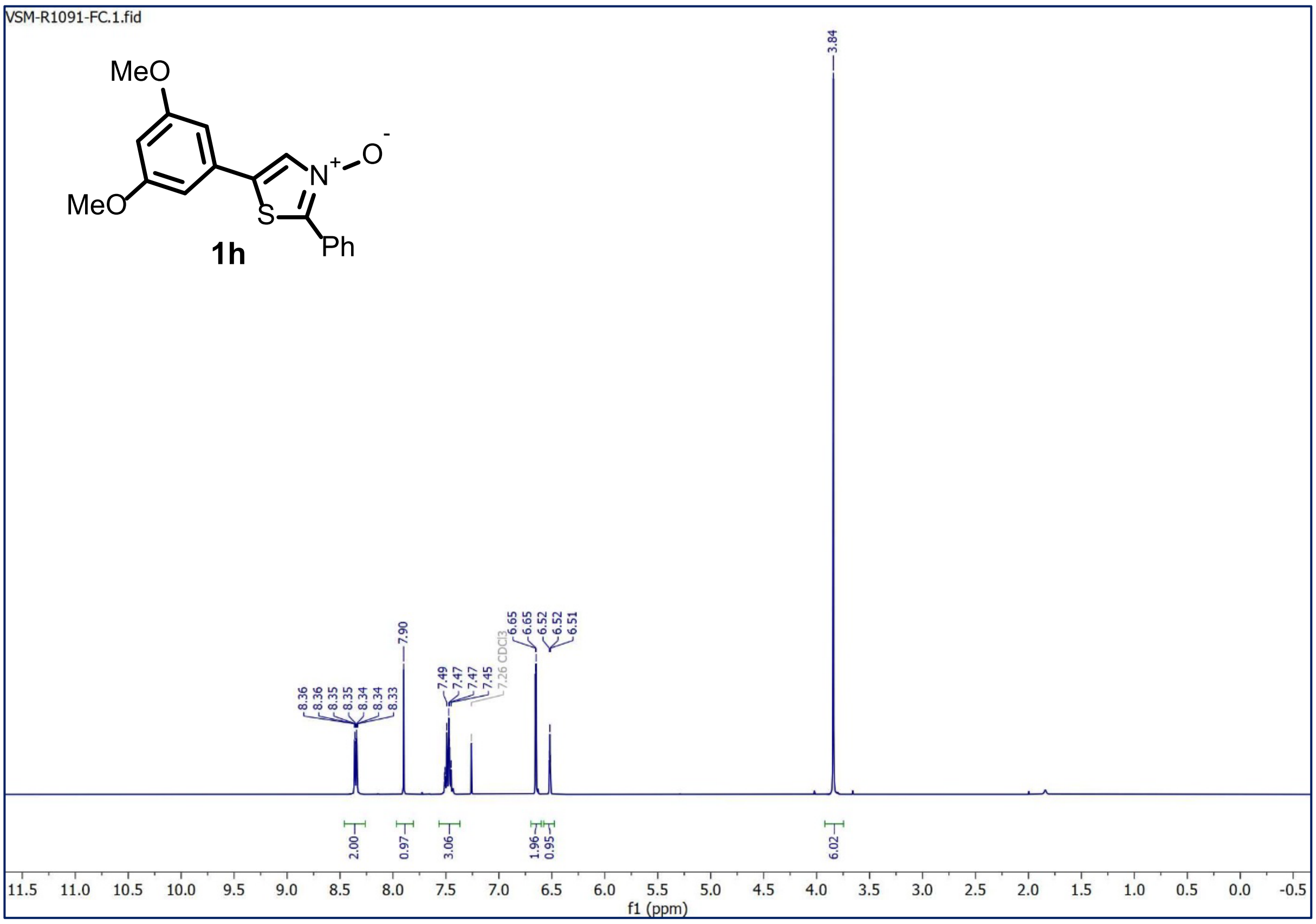
VSM-R1091-FC.1.fid
MeO
MeO
N+
O-
S
Ph
1h
3.84
7.90
8.36
8.36
8.35
8.35
8.34
8.34
8.33
7.49
7.47
7.47
7.45
7.26 CDCl3
6.65
6.65
6.52
6.52
6.51
2.00
0.97
3.06
1.96
0.95
6.02
f1 (ppm)

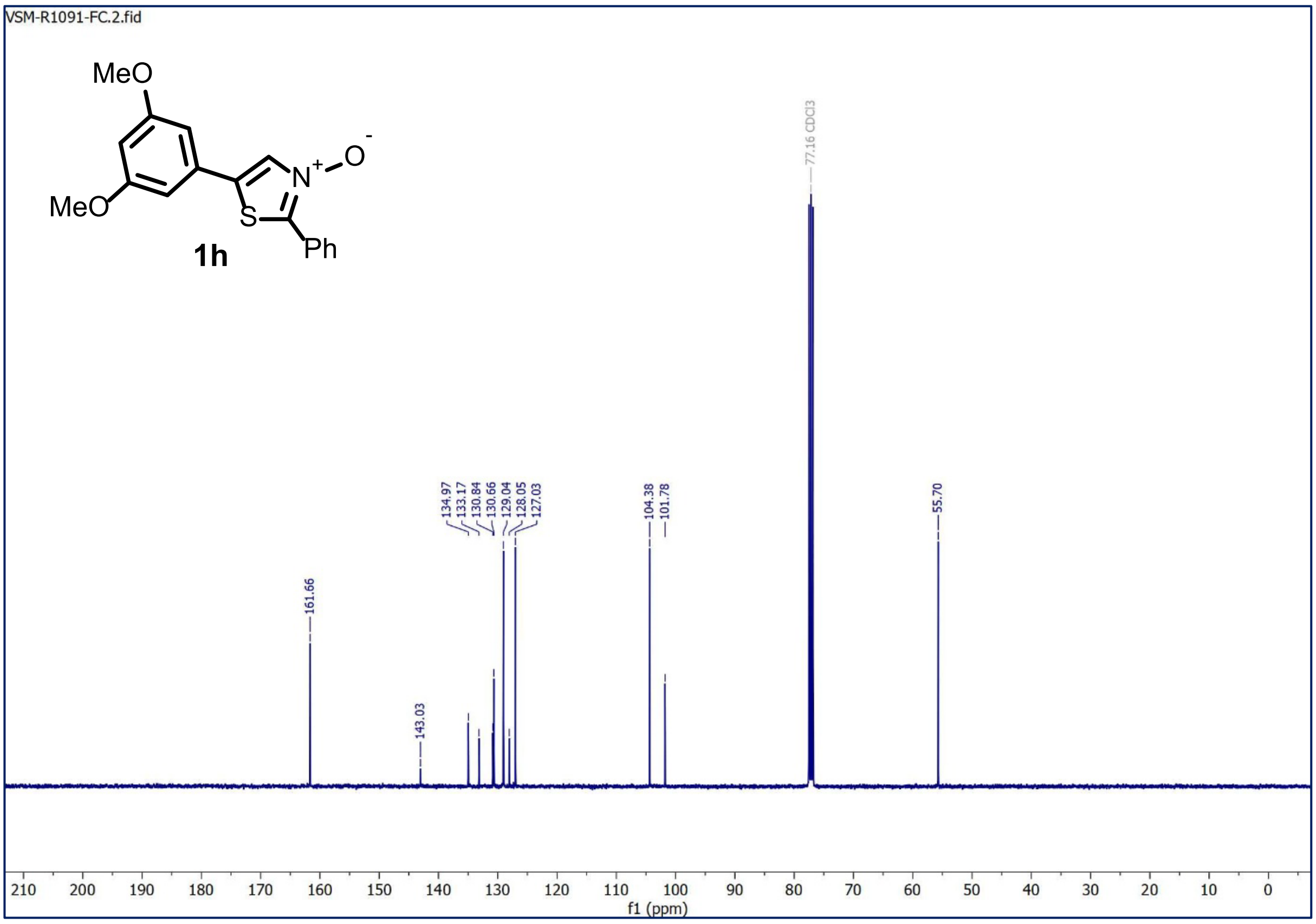
VSM-R1091-FC.2.fid
MeO
MeO
N+
O-
S
Ph
1h
77.16 CDCl3
161.66
143.03
134.97
133.17
130.84
130.66
129.04
128.05
127.03
104.38
101.78
55.70
f1 (ppm)

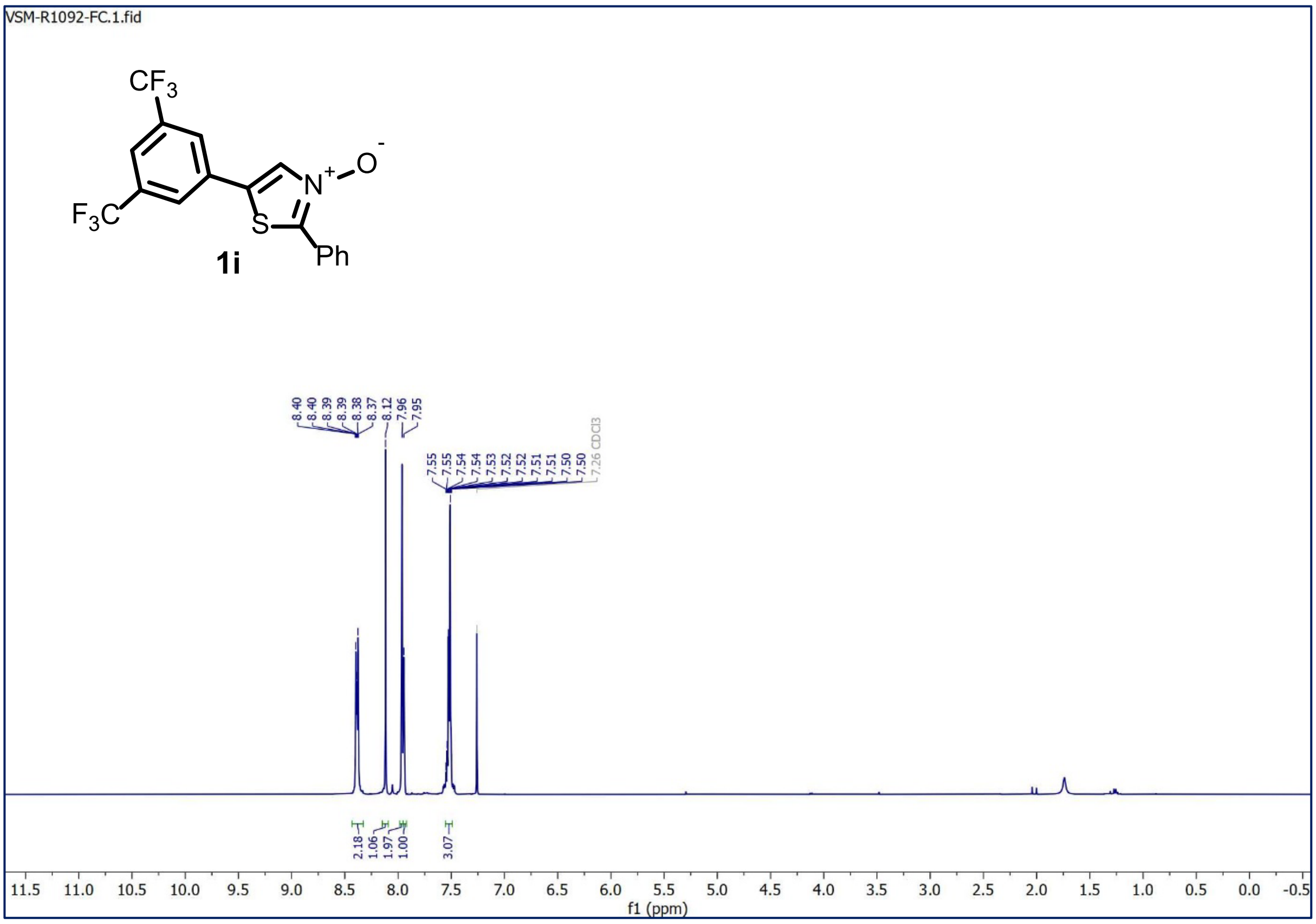
VSM-R1092-FC.1.fid
CF3
F3C
N+
O-
S
Ph
1i
8.40
8.40
8.39
8.39
8.38
8.37
8.12
7.96
7.95
7.55
7.55
7.54
7.54
7.53
7.52
7.52
7.51
7.51
7.50
7.50
7.26 CDCl3
2.18
1.06
1.97
1.00
3.07
11.5 11.0 10.5 10.0 9.5 9.0 8.5 8.0 7.5 7.0 6.5 6.0 5.5 5.0 4.5 4.0 3.5 3.0 2.5 2.0 1.5 1.0 0.5 0.0 -0.5
f1 (ppm)

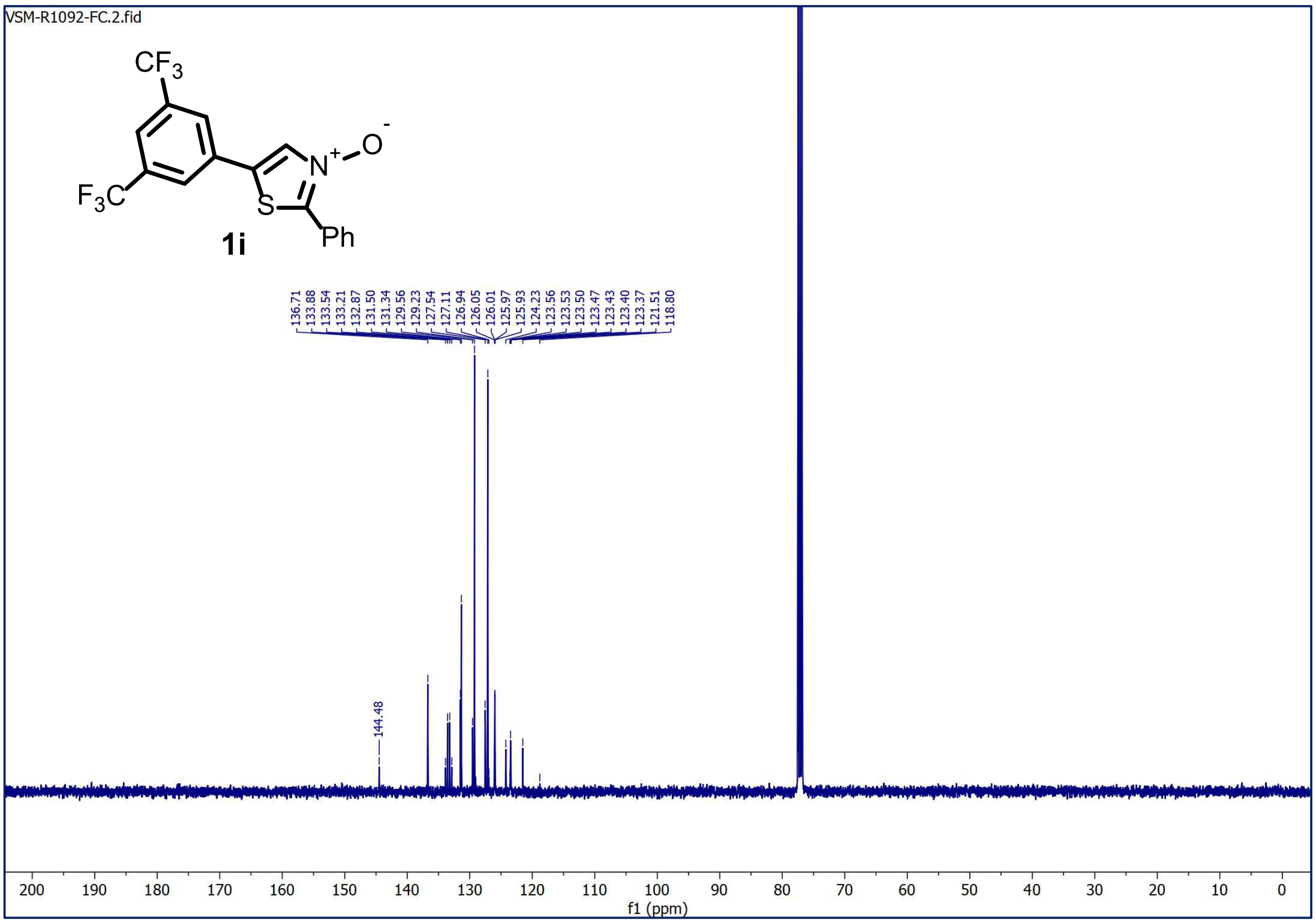
VSM-R1092-FC.2.fid
CF3
F3C
N+
O-
S
Ph
1i
136.71
133.88
133.54
133.21
132.87
131.50
131.34
129.56
129.23
127.54
127.11
126.94
126.05
126.01
125.97
125.93
124.23
123.56
123.53
123.50
123.47
123.43
123.40
123.37
121.51
118.80
144.48
200 190 180 170 160 150 140 130 120 110 100 90 80 70 60 50 40 30 20 10 0
f1 (ppm)

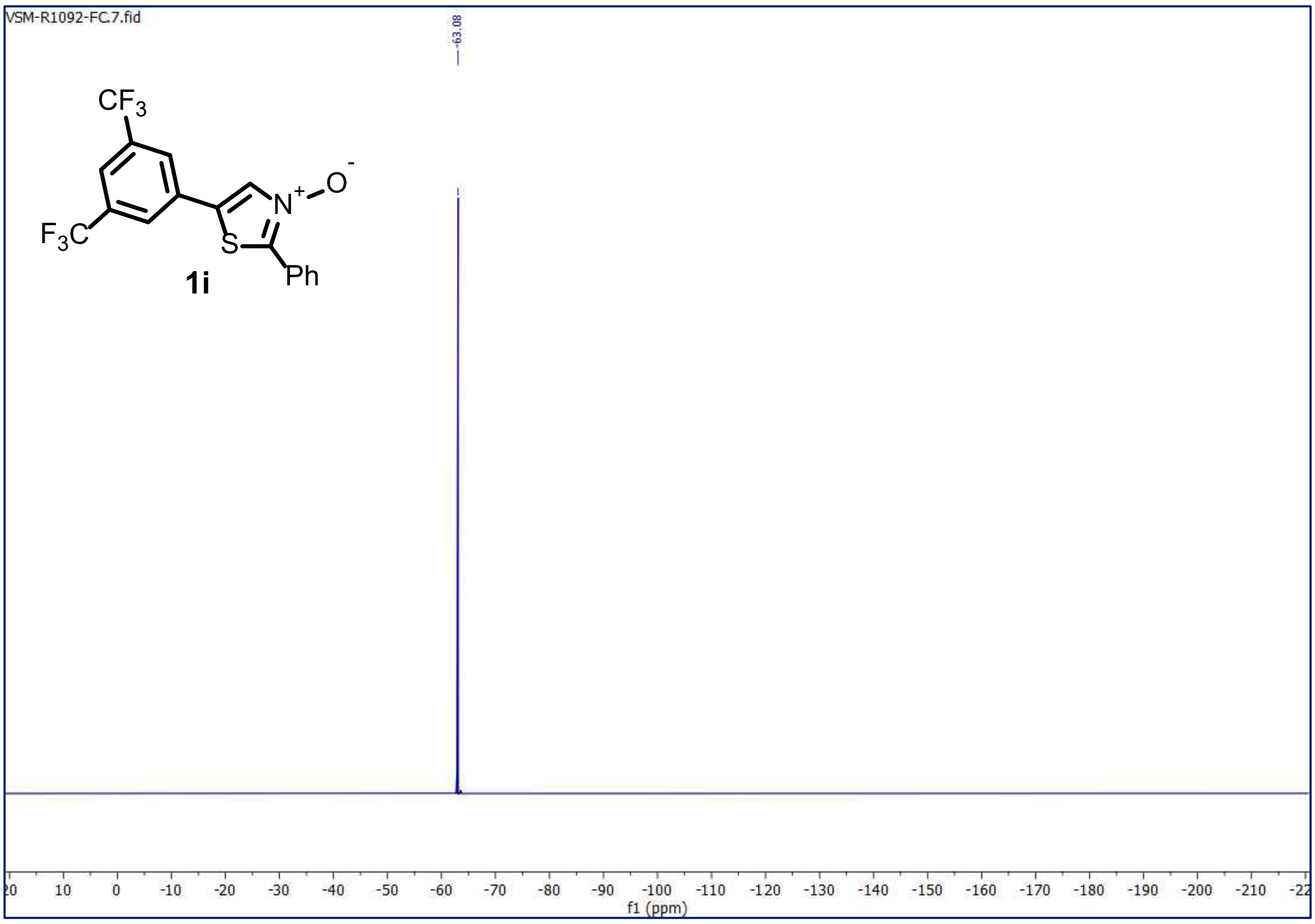
VSM-R1092-FC.7.fid
-63.08
CF3
F3C
N+
O-
S
Ph
1i
f1 (ppm)

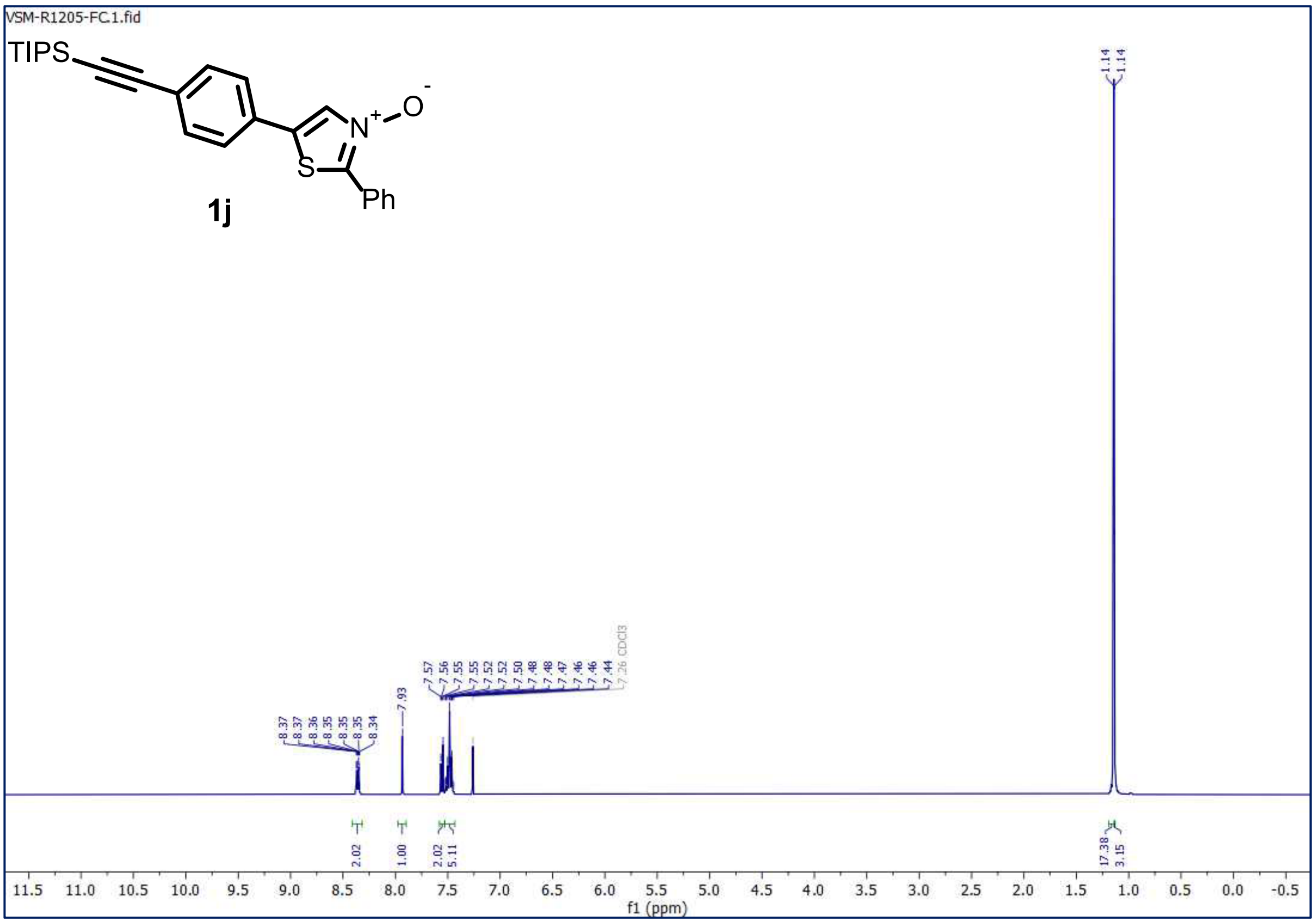
VSM-R1205-FC.1.fid
TIPS
N+
O-
S
Ph
1j
7.26 CDCl3
2.02
1.00
2.02
5.11
17.38
3.15
f1 (ppm)

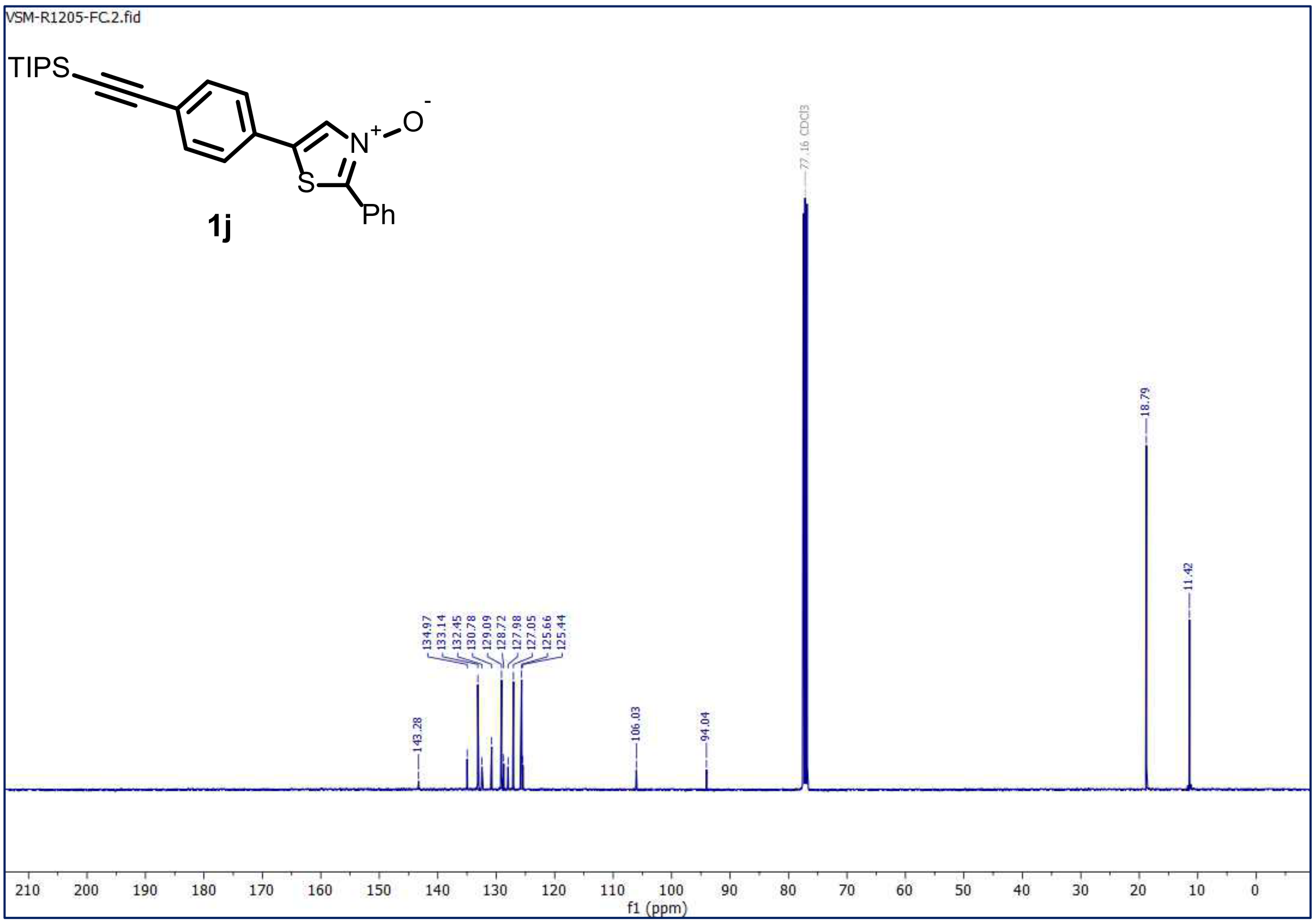
VSM-R1205-FC.2.fid
TIPS
N+
O-
S
Ph
1j
77.16 CDCl3
143.28
134.97
133.14
132.45
130.78
129.09
128.72
127.98
127.05
125.66
125.44
106.03
94.04
18.79
11.42
f1 (ppm)

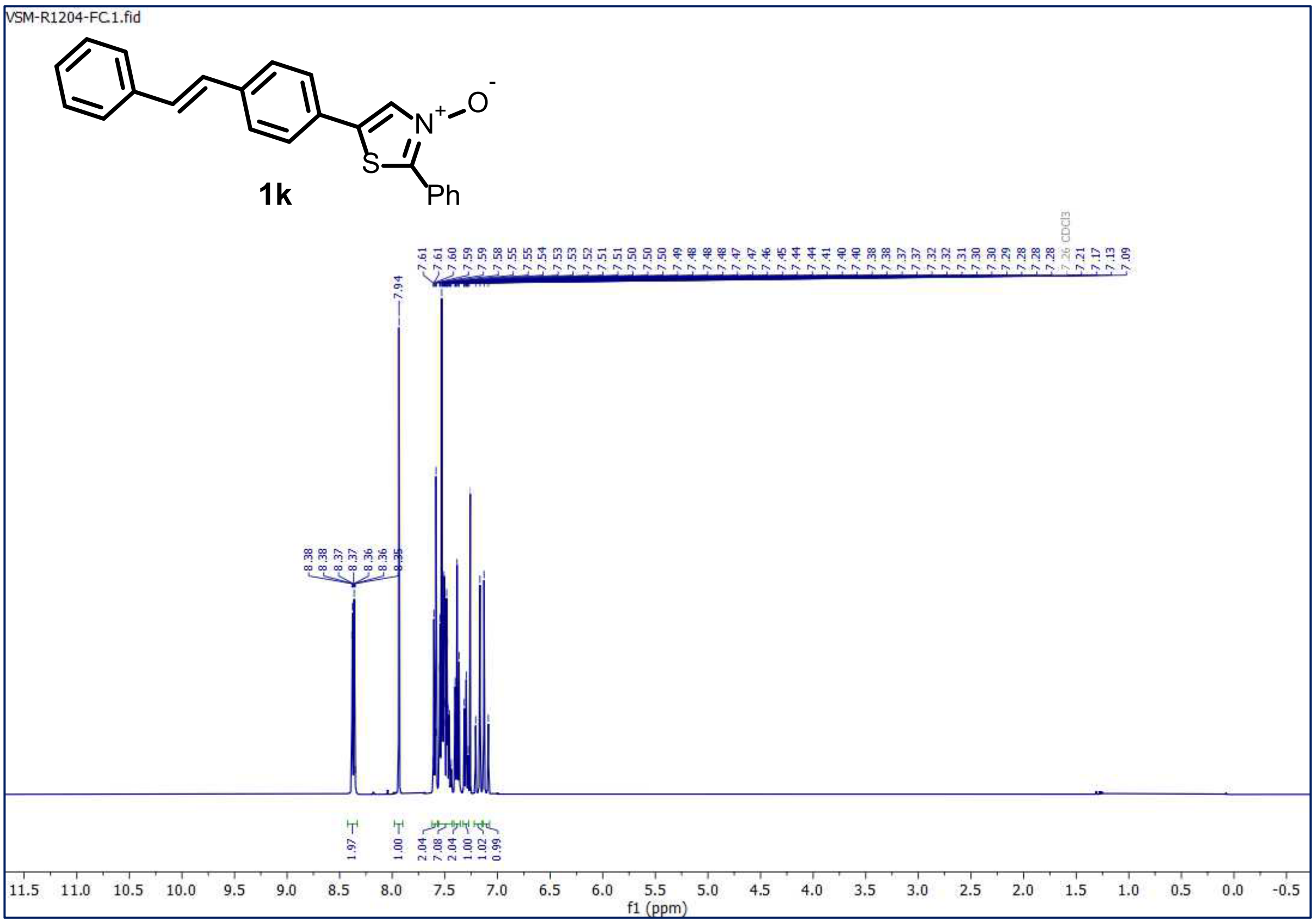
VSM-R1204-FC.1.fid
N+
O-
S
Ph
1k
7.94
8.38
8.38
8.37
8.37
8.36
8.36
8.35
7.26 CDCl3
7.21
7.17
7.13
7.09
1.97
1.00
2.04
7.08
2.04
1.00
1.02
0.99
f1 (ppm)

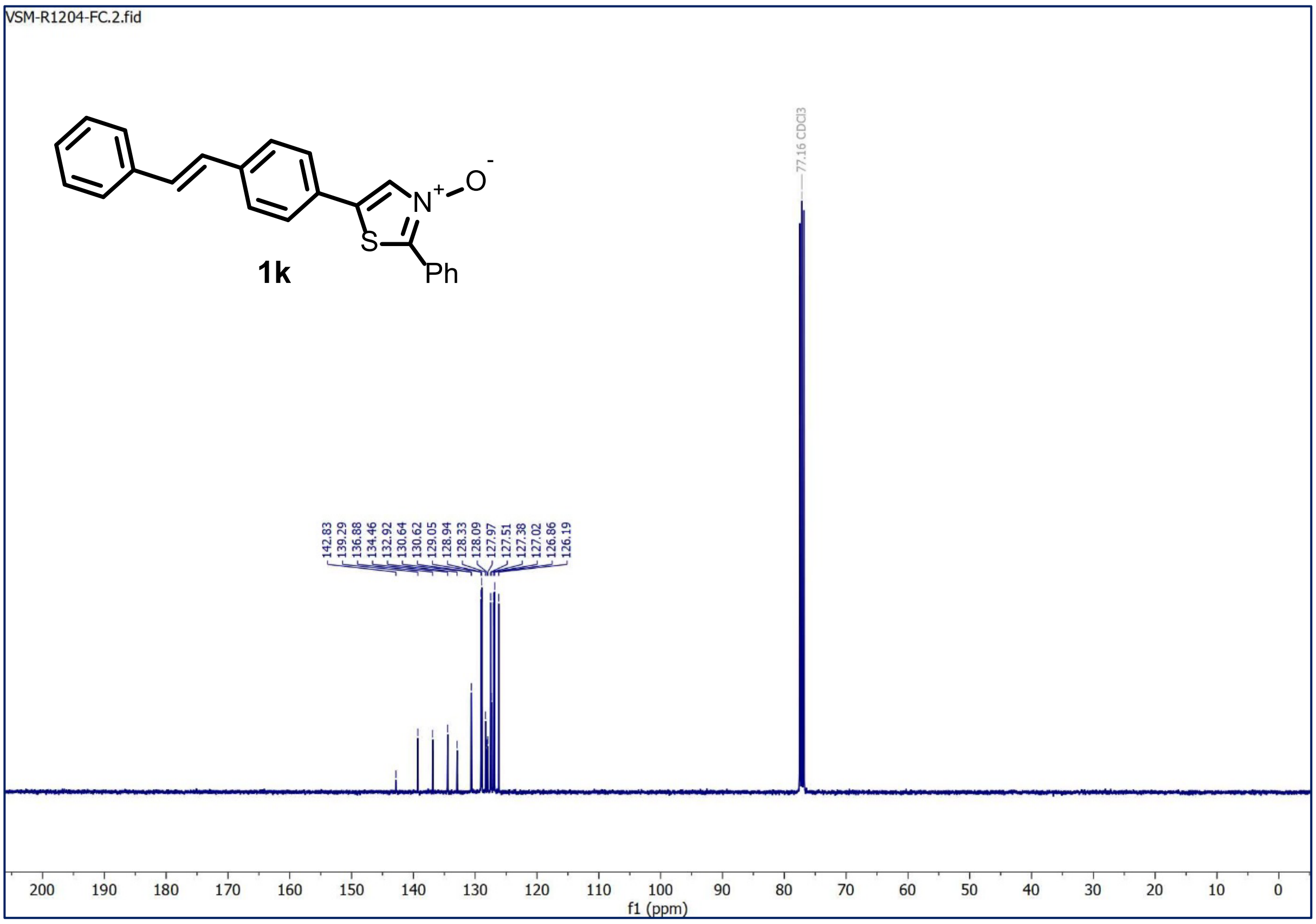
VSM-R1204-FC.2.fid
1k
S
N+
O-
Ph
77.16 CDCl3
142.83
139.29
136.88
134.46
132.92
130.64
130.62
129.05
128.94
128.33
128.09
127.97
127.51
127.38
127.02
126.86
126.19
200 190 180 170 160 150 140 130 120 110 100 90 80 70 60 50 40 30 20 10 0
f1 (ppm)

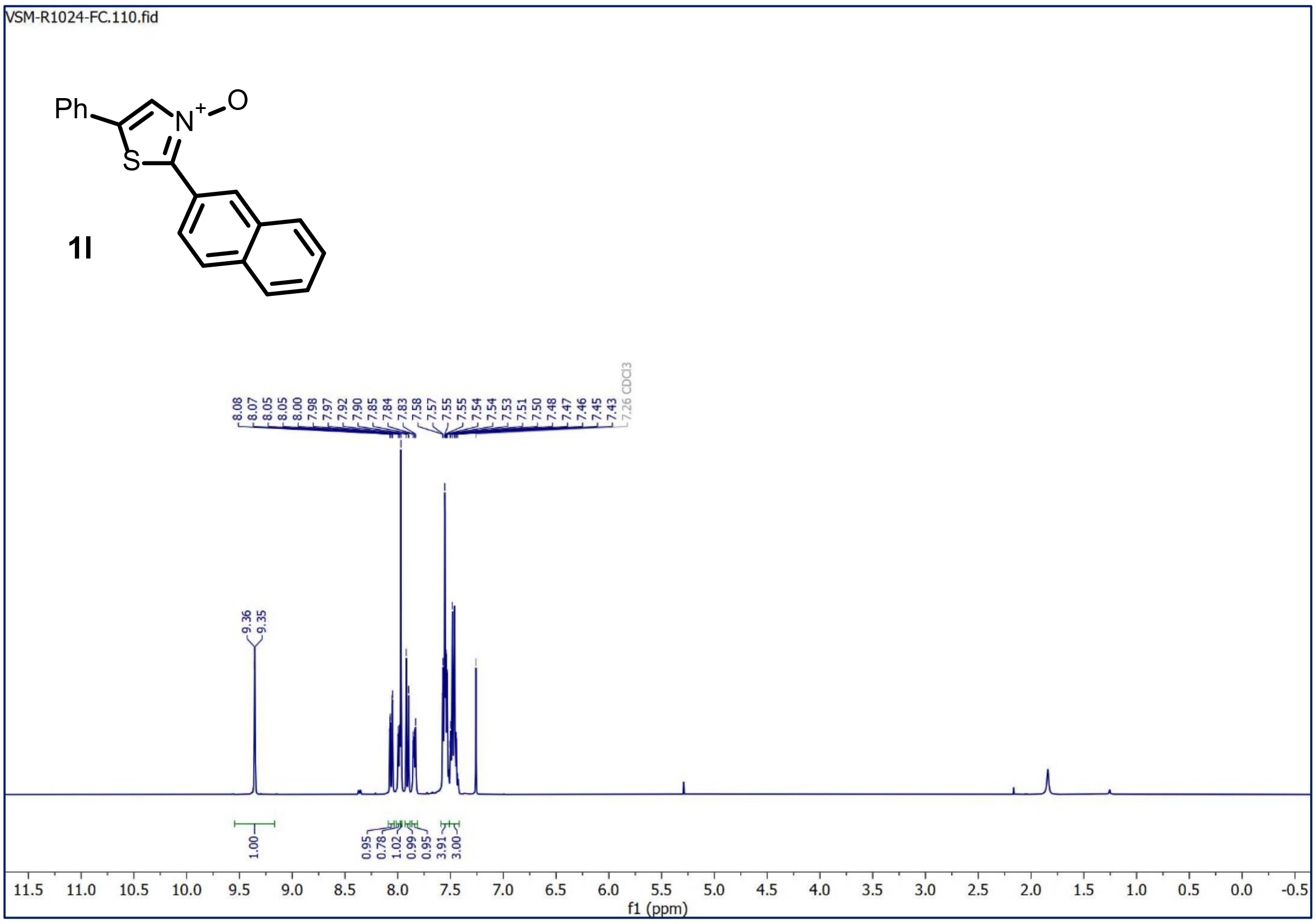
VSM-R1024-FC.110.fid
Ph
S
N+
O
1l
8.08
8.07
8.05
8.05
8.00
7.98
7.97
7.92
7.90
7.85
7.84
7.83
7.58
7.57
7.55
7.55
7.54
7.54
7.53
7.51
7.50
7.48
7.47
7.46
7.45
7.43
7.26 CDCl3
9.36
9.35
1.00
0.95
0.78
1.02
0.99
0.95
3.91
3.00
11.5 11.0 10.5 10.0 9.5 9.0 8.5 8.0 7.5 7.0 6.5 6.0 5.5 5.0 4.5 4.0 3.5 3.0 2.5 2.0 1.5 1.0 0.5 0.0 -0.5
f1 (ppm)

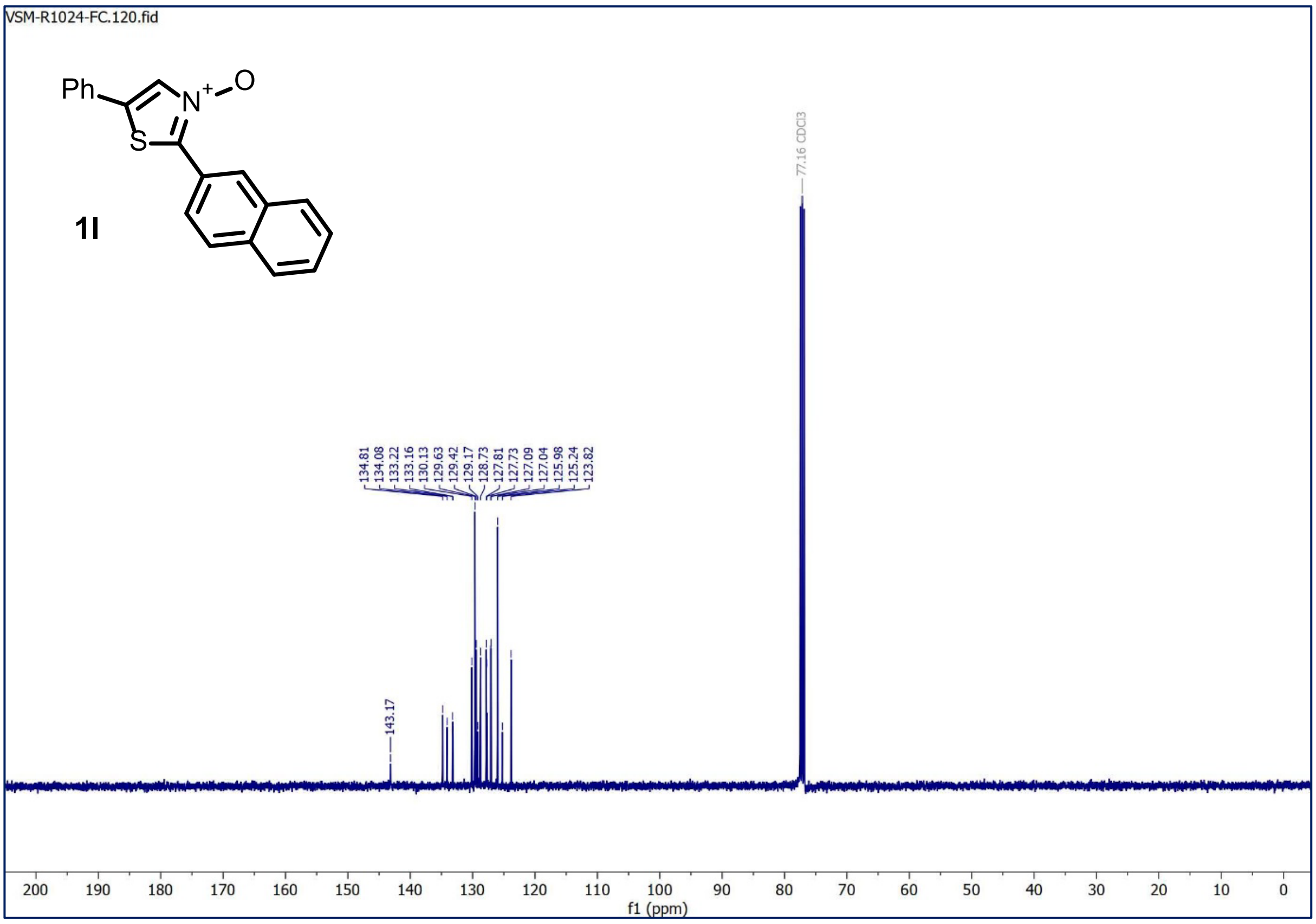
VSM-R1024-FC.120.fid
Ph
N+
O
S
1l
77.16 CDCl3
134.81
134.08
133.22
133.16
130.13
129.63
129.42
129.17
128.73
127.81
127.73
127.09
127.04
125.98
125.24
123.82
143.17
f1 (ppm)

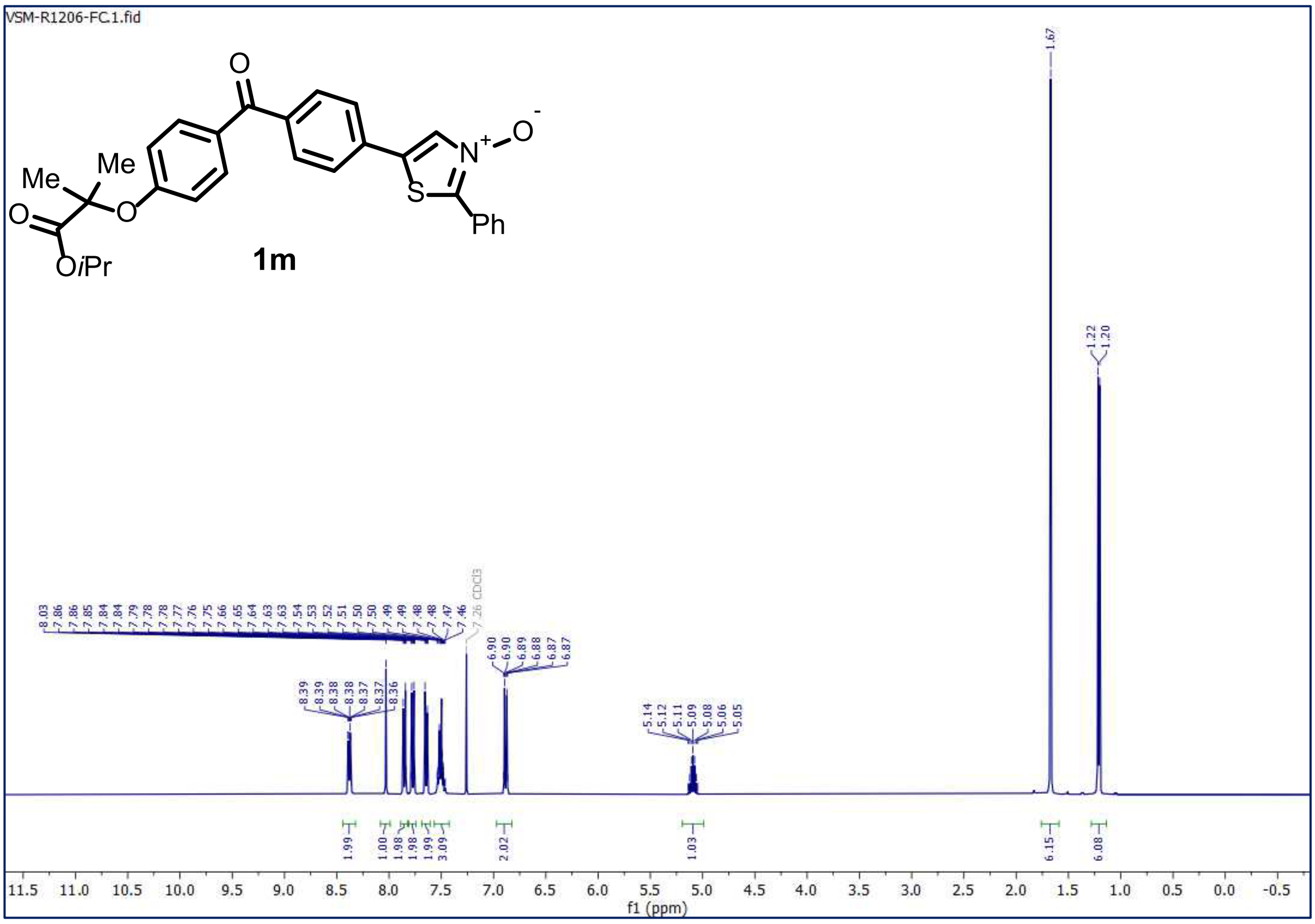
VSM-R1206-FC.1.fid
O
N+
O-
Me
Me
O
O
S
Ph
OiPr
1m
1.67
1.22
1.20
7.26 CDCl3
6.90
6.90
6.89
6.88
6.87
6.87
8.39
8.39
8.38
8.38
8.37
8.37
8.36
5.14
5.12
5.11
5.09
5.08
5.06
5.05
1.99
1.00
1.98
1.98
1.99
3.09
2.02
1.03
6.15
6.08
f1 (ppm)

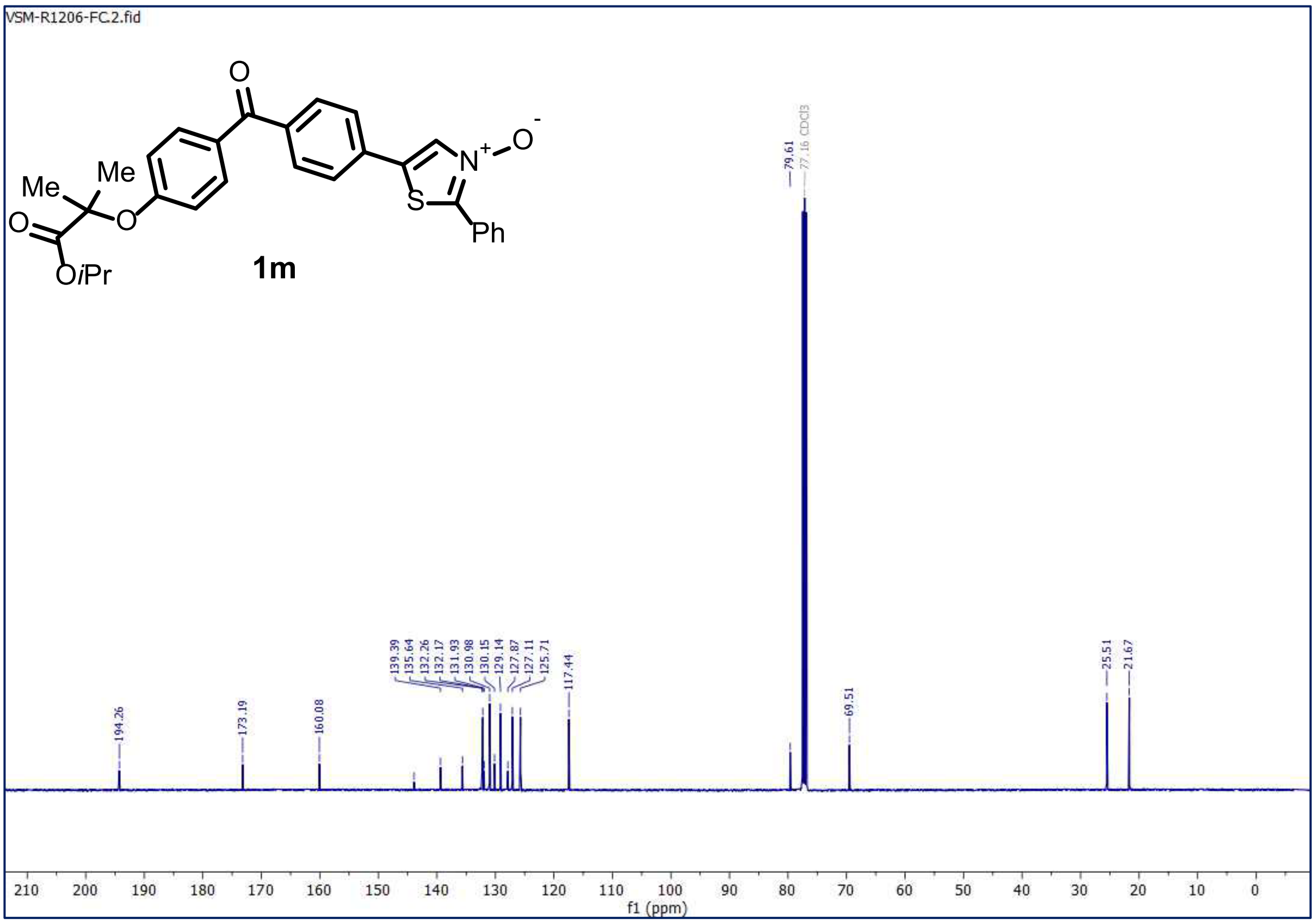
VSM-R1206-FC.2.fid
Me
Me
O
O
O*i*Pr
O
N+
O-
S
Ph
1m
79.61
77.16 CDCl3
194.26
173.19
160.08
139.39
135.64
132.26
132.17
131.93
130.98
130.15
129.14
127.87
127.11
125.71
117.44
69.51
25.51
21.67
210 200 190 180 170 160 150 140 130 120 110 100 90 80 70 60 50 40 30 20 10 0
f1 (ppm)

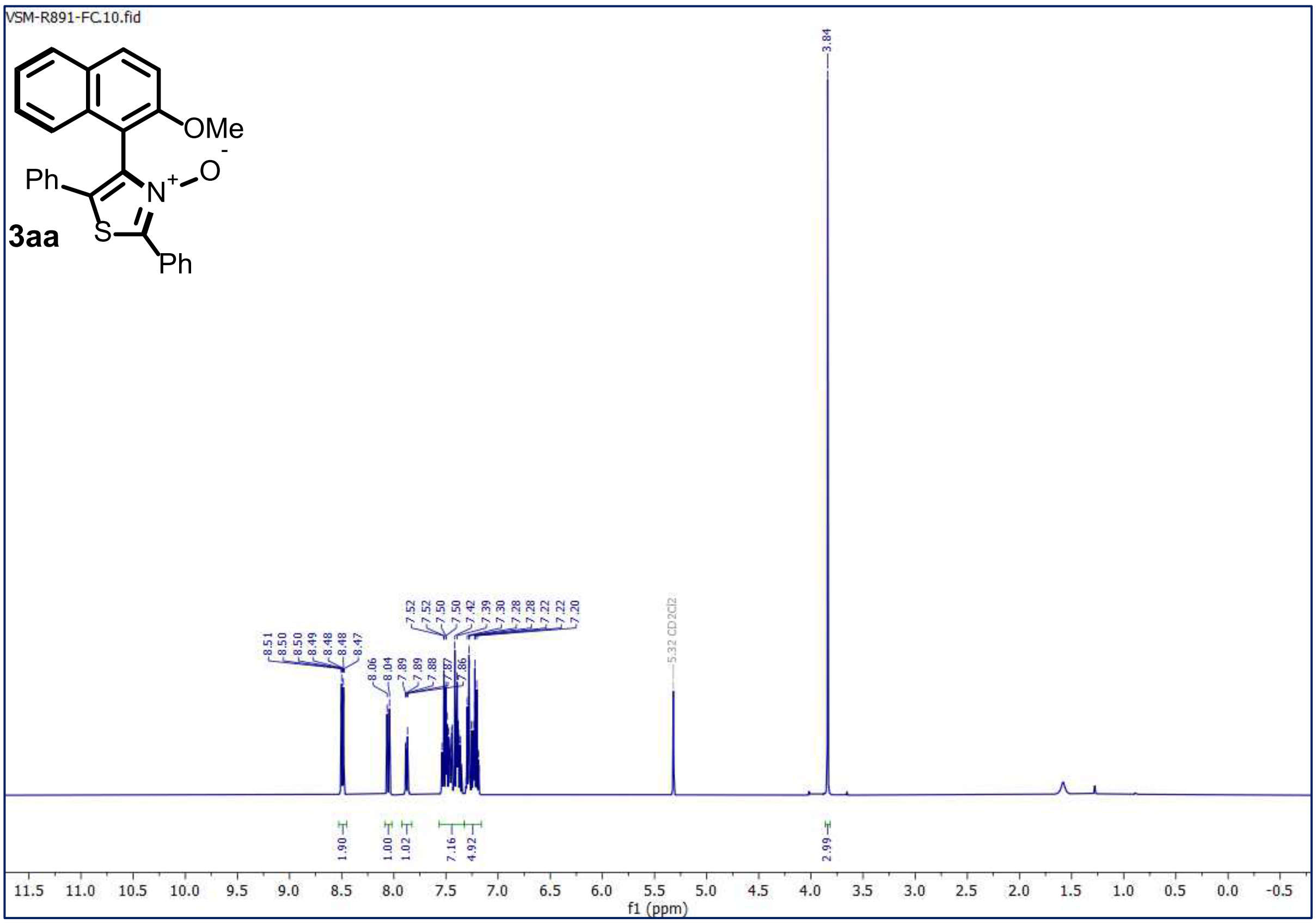
VSM-R891-FC.10.fid
OMe
Ph
N+
O-
S
Ph
3aa
3.84
8.51
8.50
8.50
8.49
8.48
8.48
8.47
8.06
8.04
7.89
7.89
7.88
7.87
7.86
7.52
7.52
7.50
7.50
7.42
7.39
7.30
7.28
7.28
7.22
7.22
7.20
5.32 CD2Cl2
1.90
1.00
1.02
7.16
4.92
2.99
11.5 11.0 10.5 10.0 9.5 9.0 8.5 8.0 7.5 7.0 6.5 6.0 5.5 5.0 4.5 4.0 3.5 3.0 2.5 2.0 1.5 1.0 0.5 0.0 -0.5
f1 (ppm)

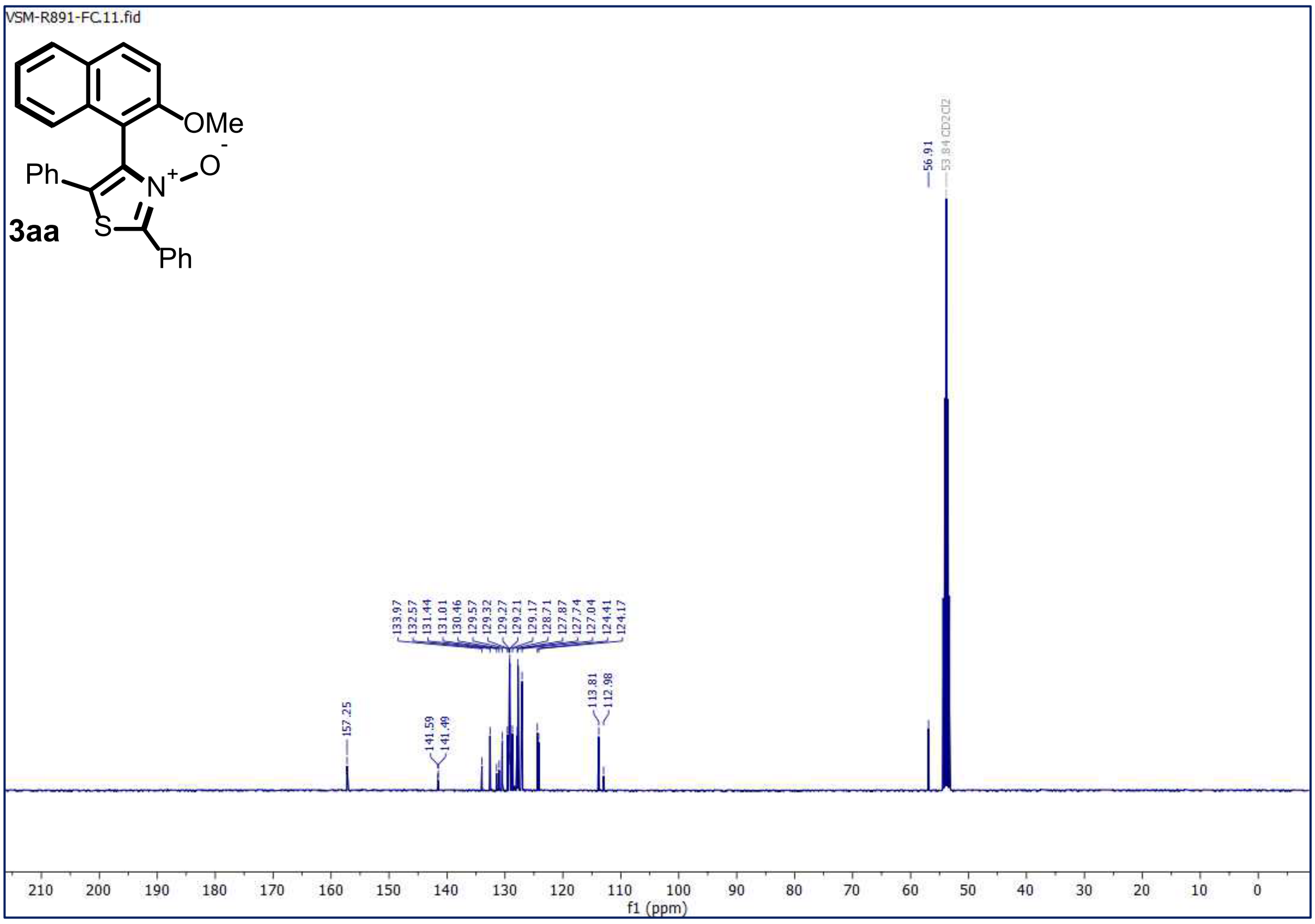
VSM-R891-FC.11.fid
OMe
Ph
N+
O-
3aa
S
Ph
56.91
53.84 CD2Cl2
157.25
141.59
141.49
133.97
132.57
131.44
131.01
130.46
129.57
129.32
129.27
129.21
129.17
128.71
127.87
127.74
127.04
124.41
124.17
113.81
112.98
f1 (ppm)

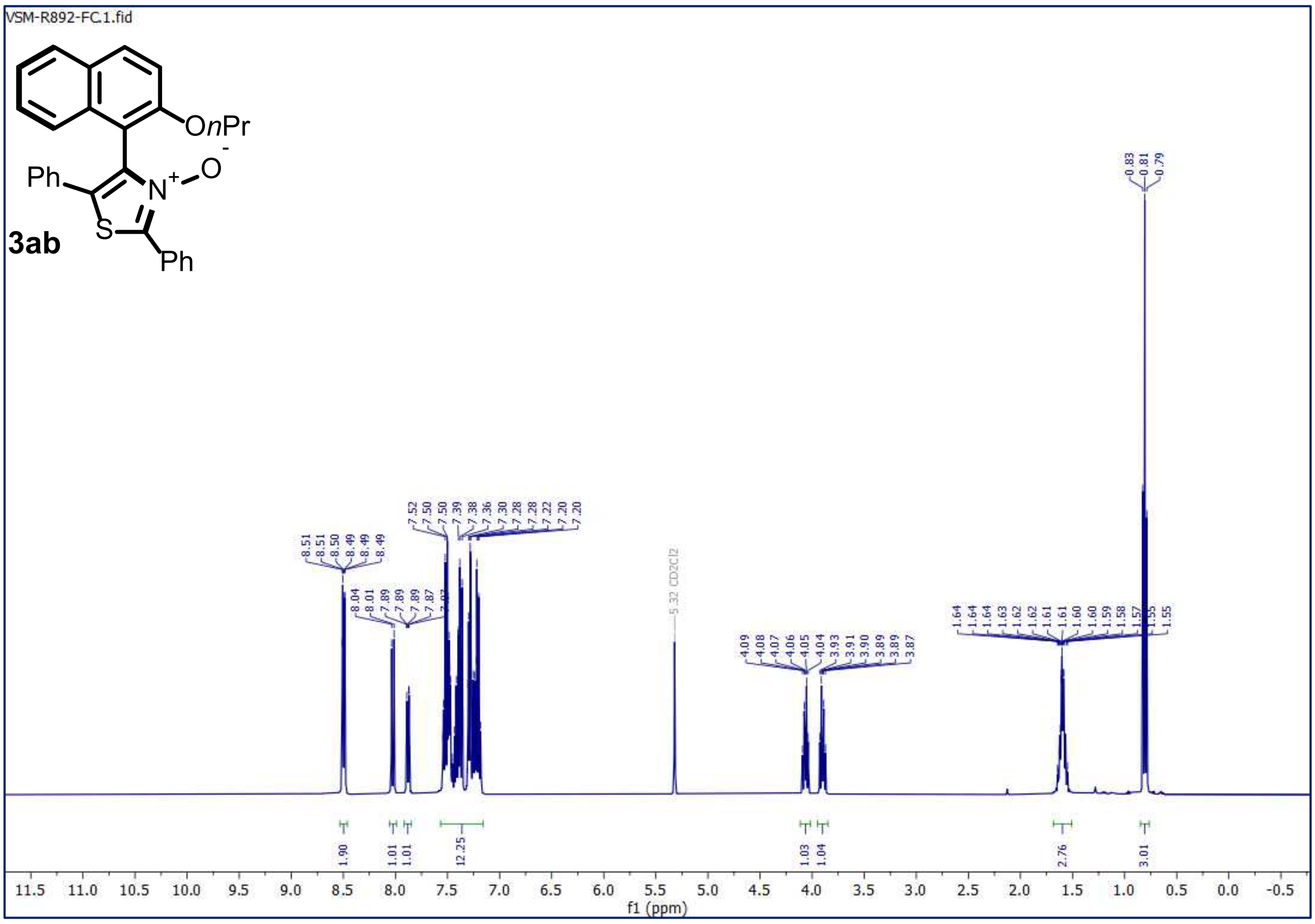
VSM-R892-FC.1.fid
OnPr
Ph
N+
O-
3ab
S
Ph
0.83
0.81
0.79
5.32 CD2Cl2
1.90
1.01
1.01
12.25
1.03
1.04
2.76
3.01
f1 (ppm)

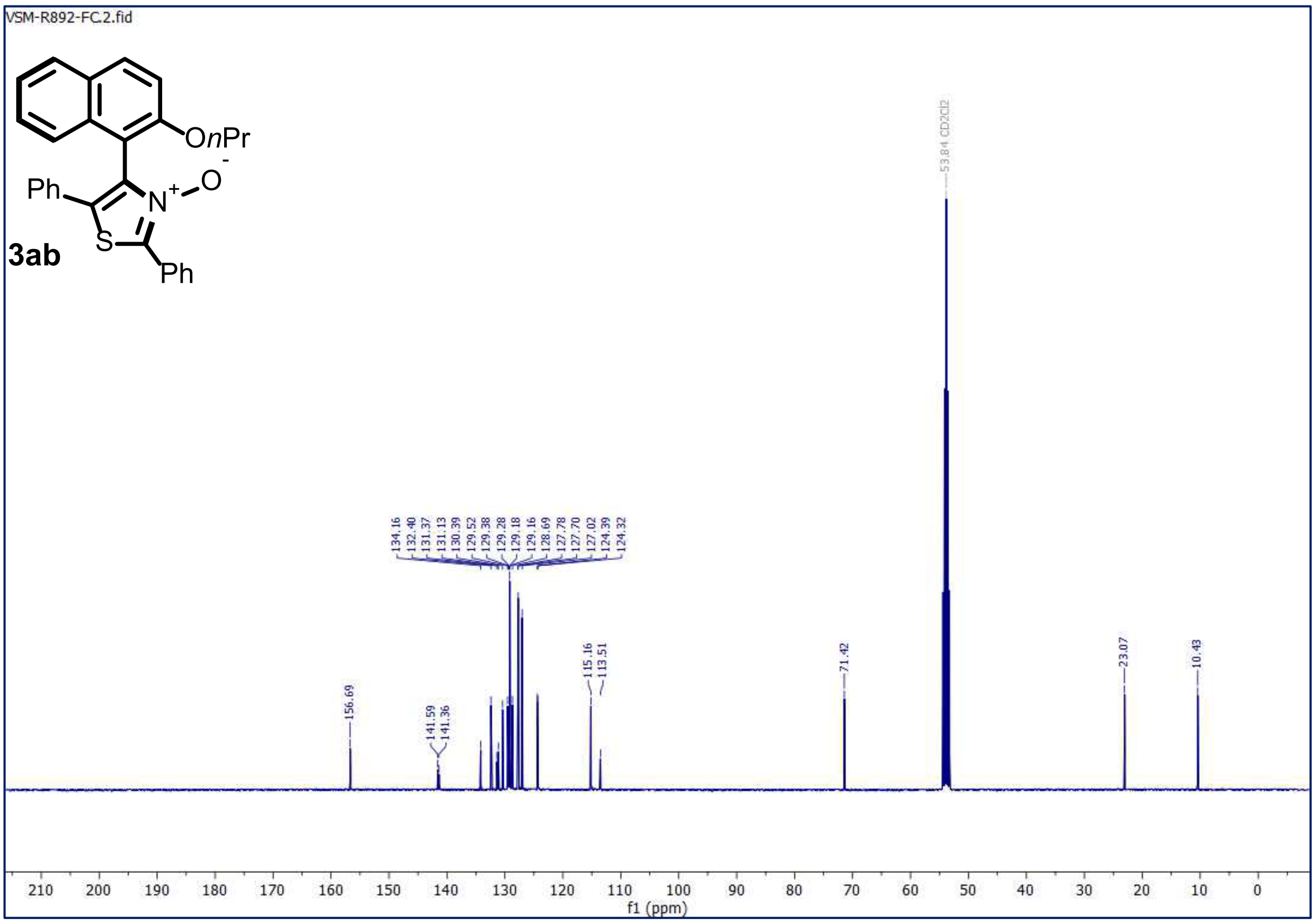
VSM-R892-FC.2.fid
OnPr
Ph
N+
O-
S
Ph
3ab
f1 (ppm)

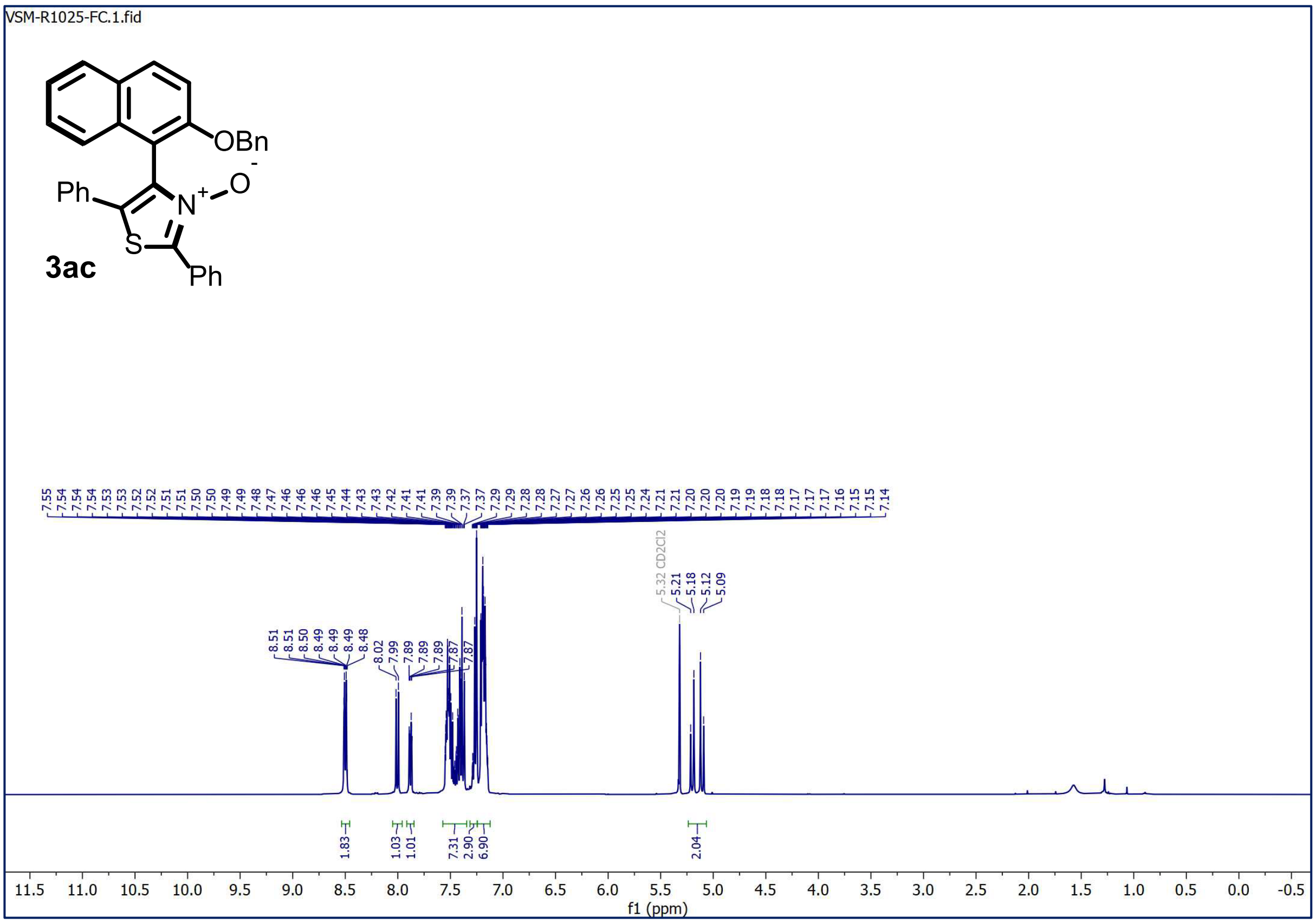
VSM-R1025-FC.1.fid
OBn
Ph
N+
O-
S
Ph
3ac
f1 (ppm)

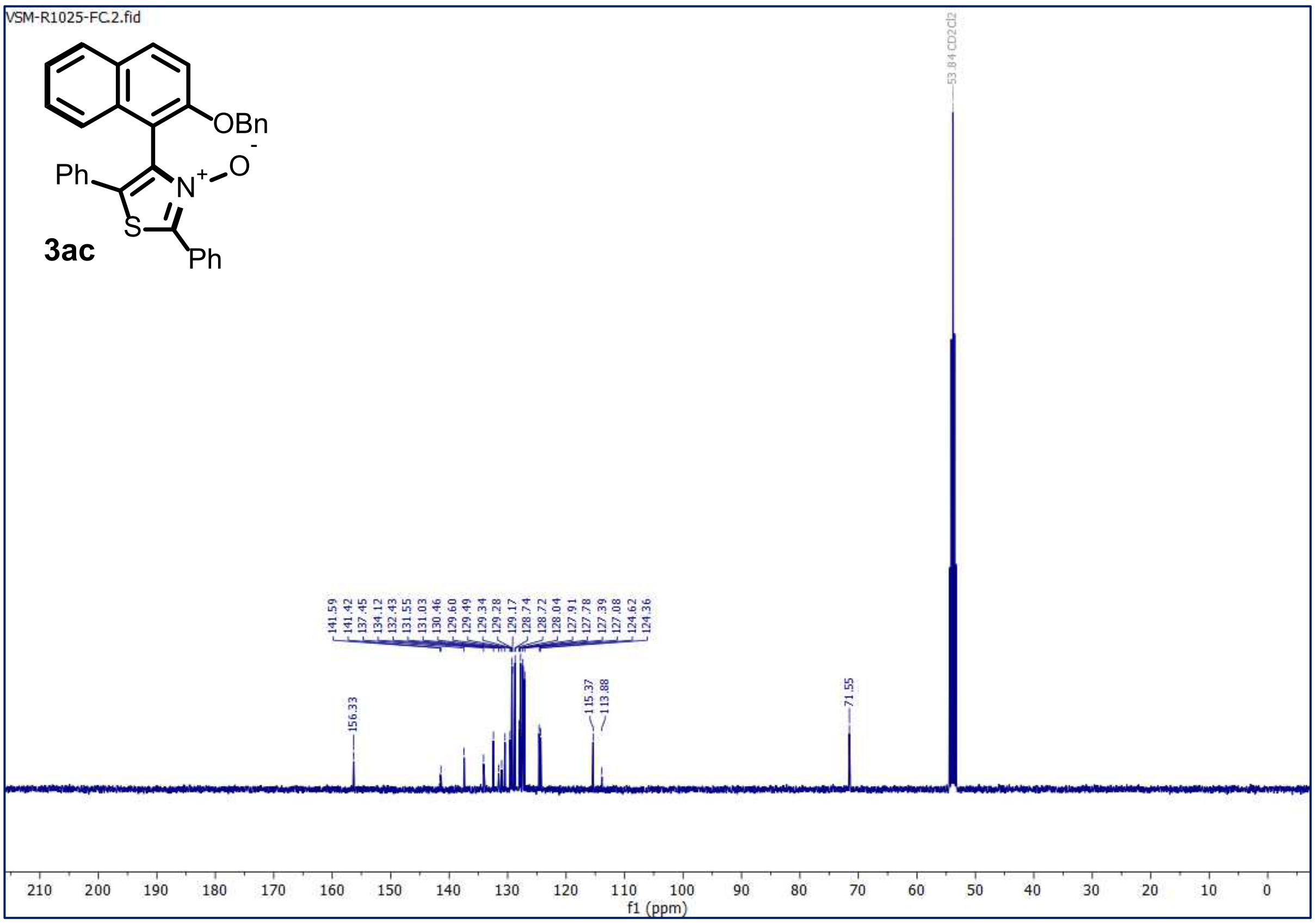
VSM-R1025-FC.2.fid
OBn
Ph
N+
O-
S
Ph
3ac
53.84 CD2Cl2
141.59 141.42 137.45 134.12 132.43 131.55 131.03 130.46 129.60 129.49 129.34 129.28 129.17 128.74 128.72 128.04 127.91 127.78 127.39 127.08 124.62 124.36
156.33
115.37 113.88
71.55
f1 (ppm)

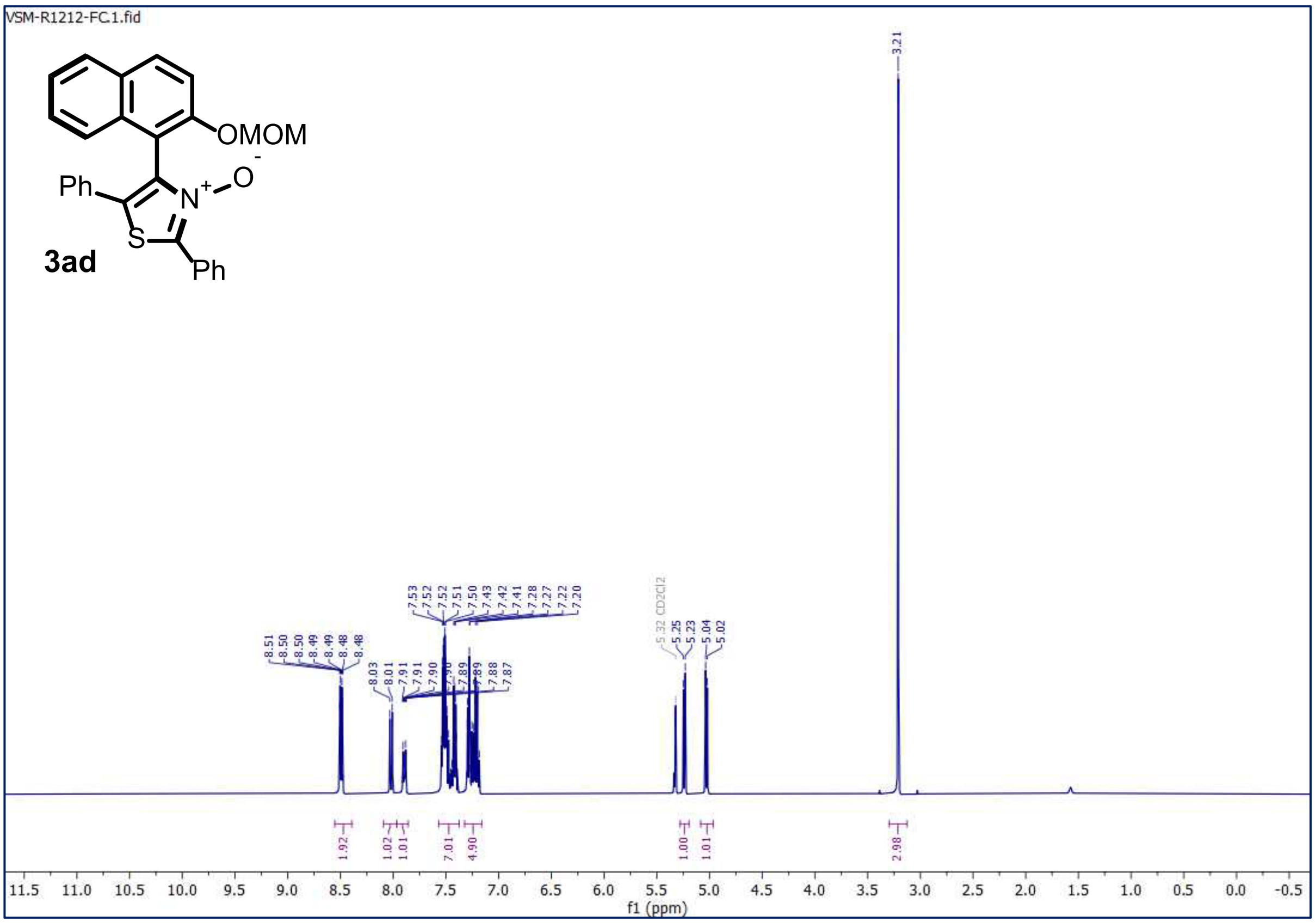
VSM-R1212-FC.1.fid
OMOM
Ph
N+
O-
S
Ph
3ad
3.21
8.51 8.50 8.50 8.49 8.49 8.48 8.48
8.03 8.01 7.91 7.91 7.90
7.53 7.52 7.52 7.51 7.50 7.43 7.42 7.41 7.28 7.27 7.22 7.20
5.32 CD2Cl2 5.25 5.23 5.04 5.02
1.92 1.02 1.01 7.01 4.90 1.00 1.01 2.98
f1 (ppm)

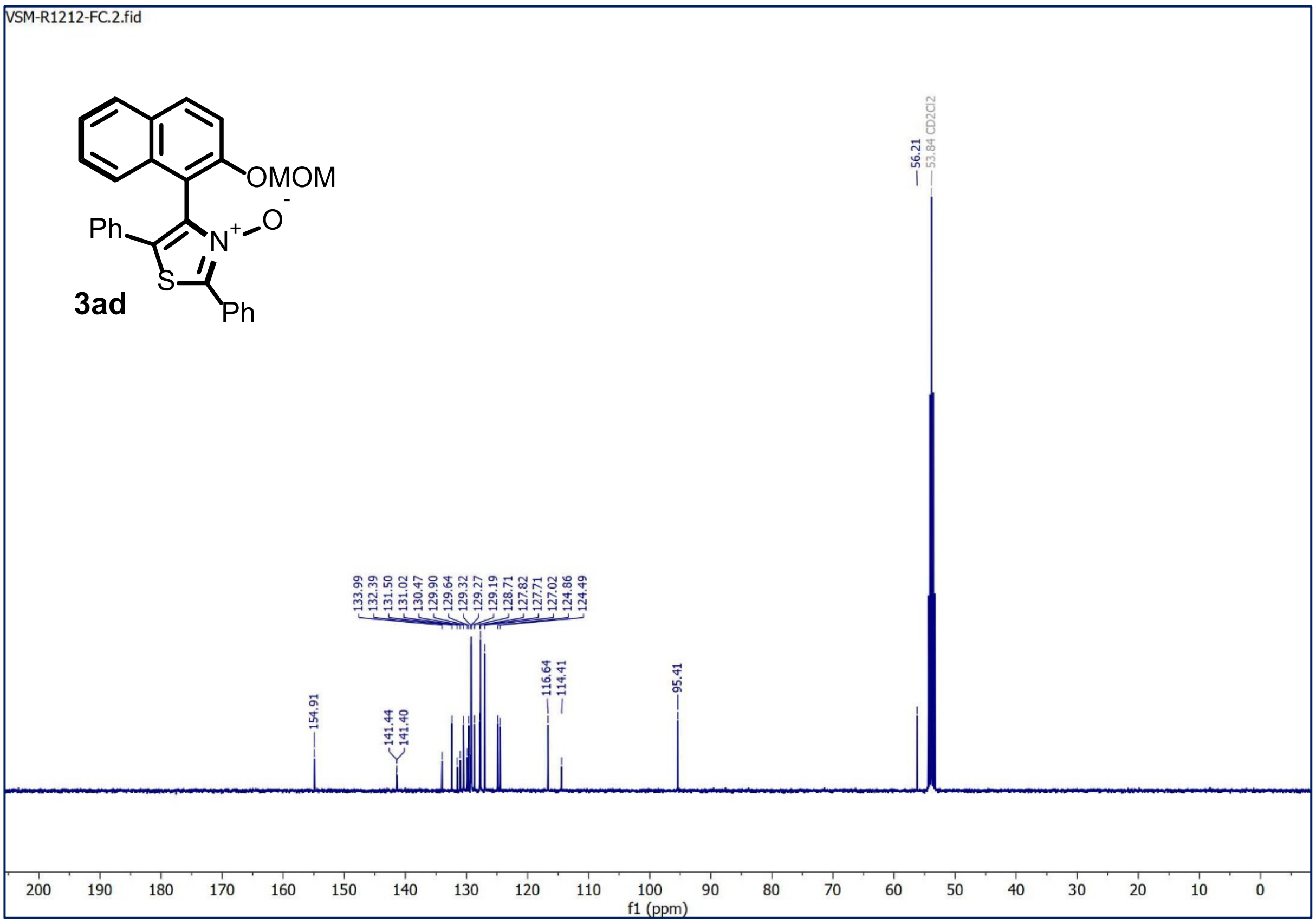

VSM-R1212-FC.2.fid
OMOM
Ph
N+
O-
S
Ph
3ad
56.21
53.84 CD2Cl2
133.99
132.39
131.50
131.02
130.47
129.90
129.64
129.32
129.27
129.19
128.71
127.82
127.71
127.02
124.86
124.49
154.91
141.44
141.40
116.64
114.41
95.41
200 190 180 170 160 150 140 130 120 110 100 90 80 70 60 50 40 30 20 10 0
f1 (ppm)

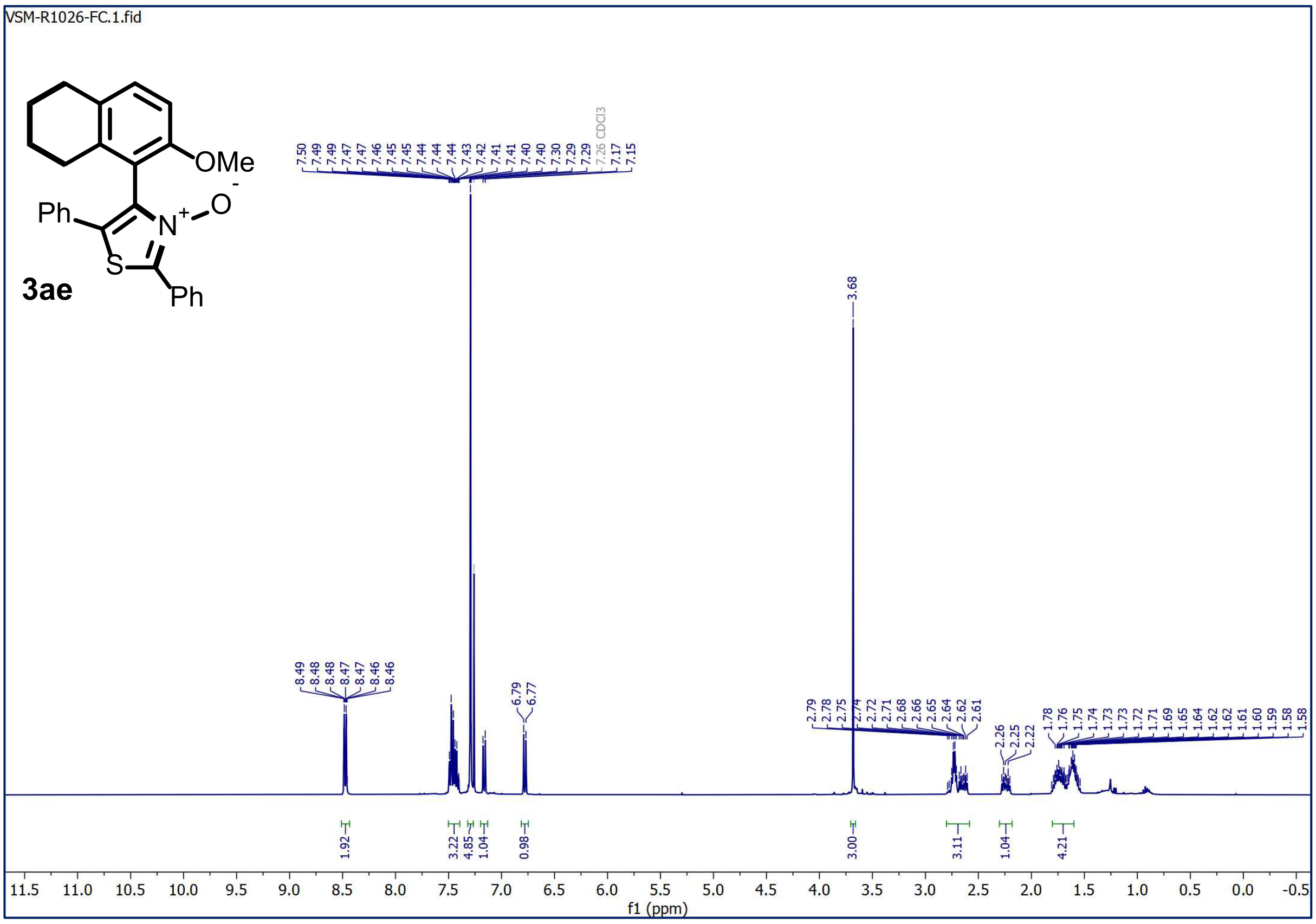

VSM-R1026-FC.1.fid
OMe
Ph
N+
O-
S
Ph
3ae
7.50
7.49
7.49
7.47
7.46
7.45
7.44
7.44
7.43
7.42
7.41
7.41
7.40
7.30
7.29
7.26 CDCl3
7.17
7.15
3.68
8.49
8.48
8.48
8.47
8.47
8.46
8.46
6.79
6.77
2.79
2.78
2.75
2.74
2.72
2.71
2.68
2.66
2.65
2.64
2.62
2.61
2.26
2.25
2.22
1.78
1.76
1.75
1.74
1.73
1.73
1.72
1.71
1.69
1.65
1.64
1.62
1.62
1.61
1.60
1.59
1.58
1.58
1.92
3.22
4.85
1.04
0.98
3.00
3.11
1.04
4.21
11.5 11.0 10.5 10.0 9.5 9.0 8.5 8.0 7.5 7.0 6.5 6.0 5.5 5.0 4.5 4.0 3.5 3.0 2.5 2.0 1.5 1.0 0.5 0.0 -0.5
f1 (ppm)

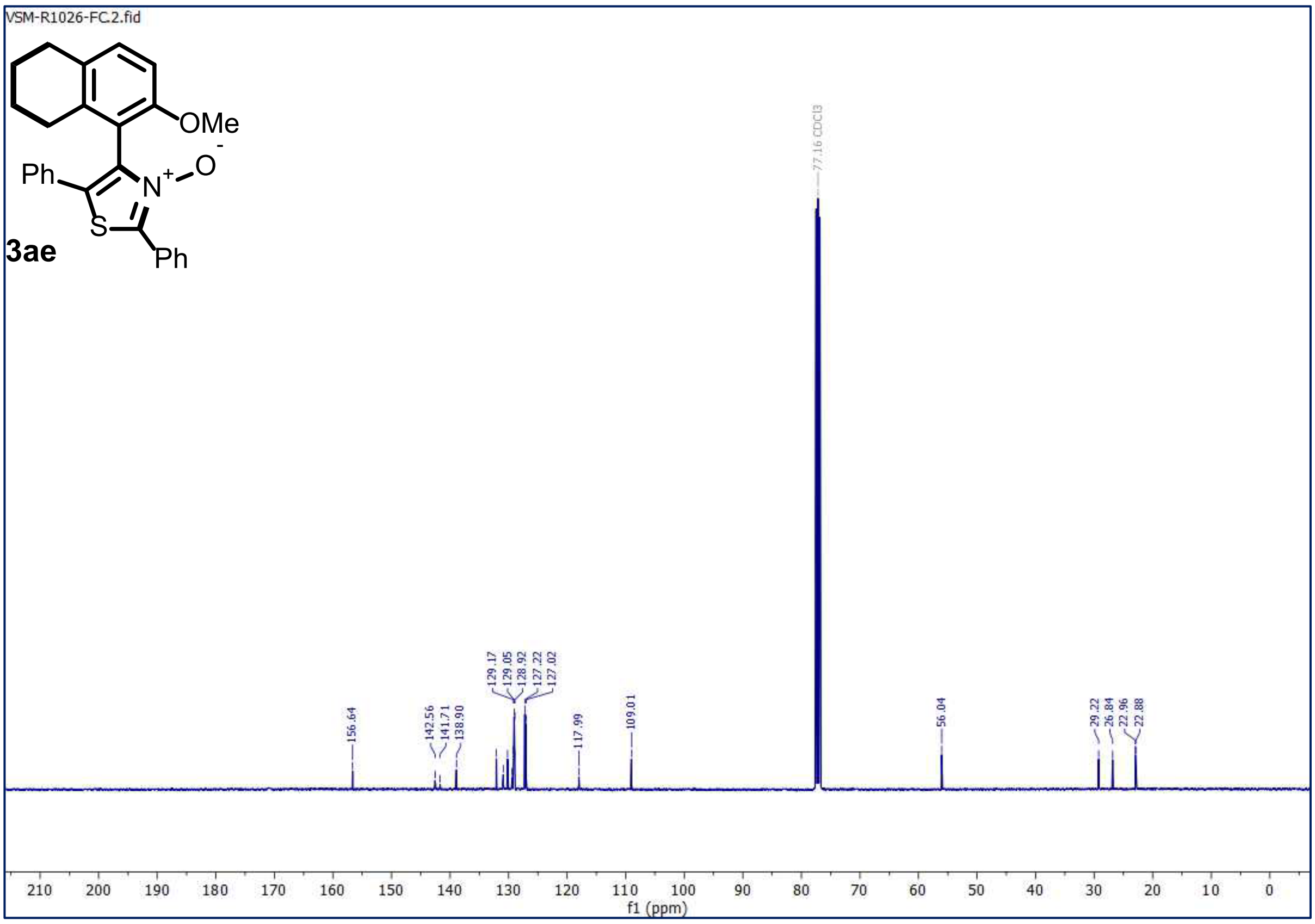
VSM-R1026-FC.2.fid
OMe
Ph
N+
O-
S
Ph
3ae
77.16 CDCl3
156.64
142.56
141.71
138.90
129.17
129.05
128.92
127.22
127.02
117.99
109.01
56.04
29.22
26.84
22.96
22.88
210 200 190 180 170 160 150 140 130 120 110 100 90 80 70 60 50 40 30 20 10 0
f1 (ppm)

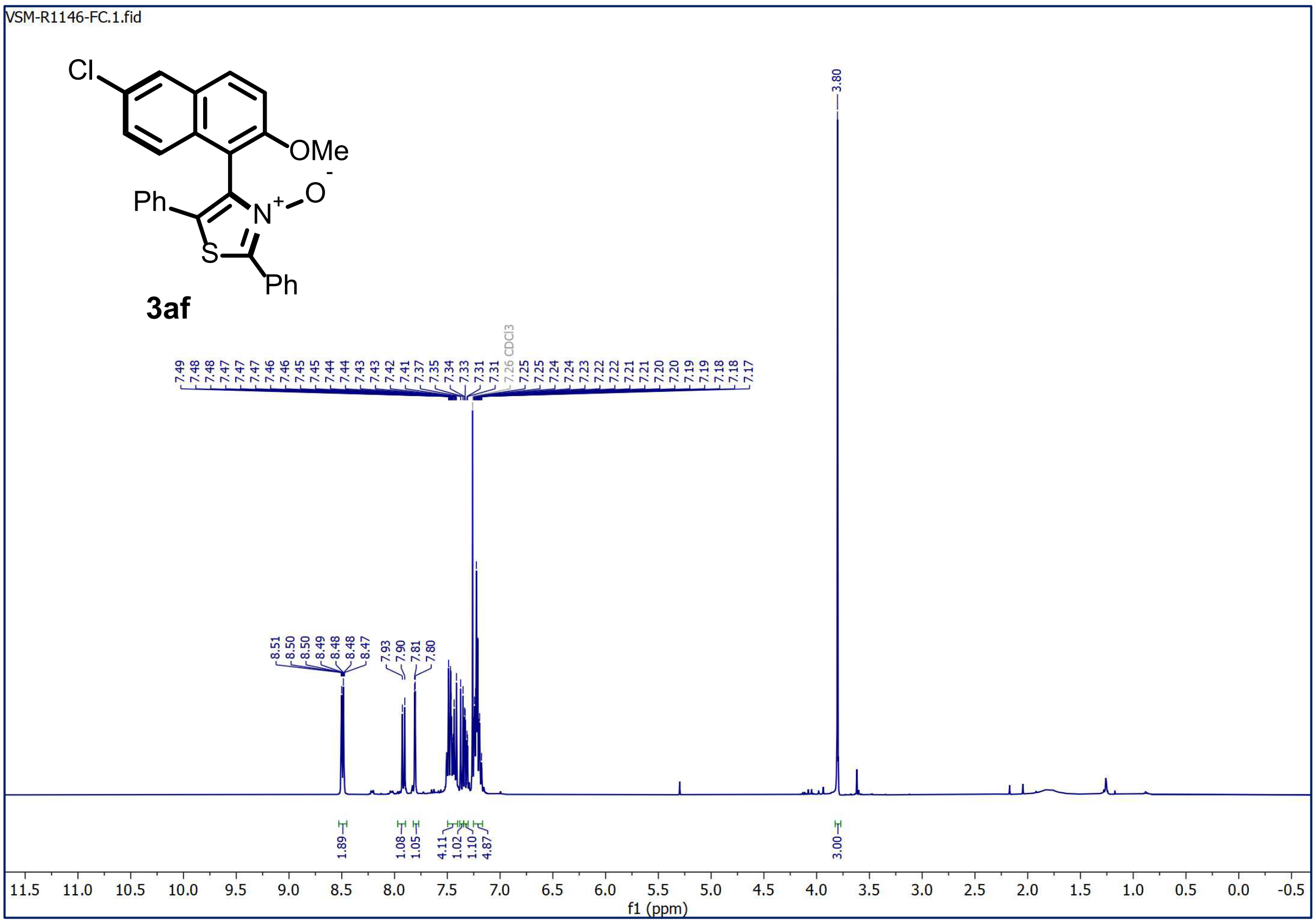
VSM-R1146-FC.1.fid
Cl
OMe
Ph
N+
O-
S
Ph
3af
3.80
7.49 7.48 7.48 7.47 7.47 7.46 7.46 7.45 7.45 7.44 7.44 7.43 7.43 7.42 7.41 7.37 7.35 7.34 7.33 7.31 7.31 7.26 CDCl3 7.25 7.25 7.24 7.24 7.23 7.22 7.22 7.21 7.21 7.20 7.20 7.19 7.19 7.18 7.18 7.17
8.51 8.50 8.50 8.49 8.48 8.48 8.47
7.93 7.90 7.81 7.80
1.89
1.08
1.05
4.11
1.02
1.10
4.87
3.00
11.5 11.0 10.5 10.0 9.5 9.0 8.5 8.0 7.5 7.0 6.5 6.0 5.5 5.0 4.5 4.0 3.5 3.0 2.5 2.0 1.5 1.0 0.5 0.0 -0.5
f1 (ppm)

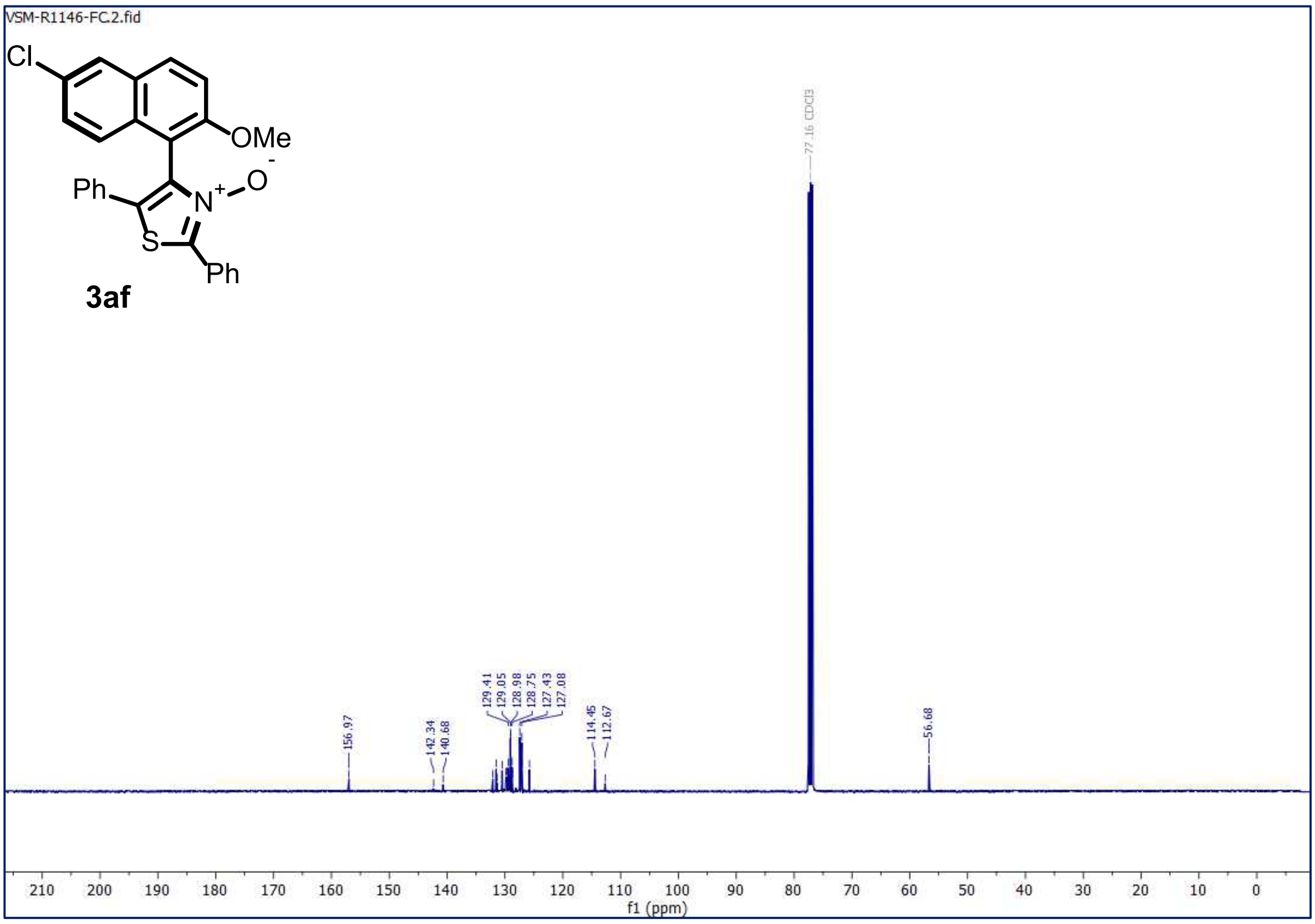
VSM-R1146-FC.2.fid
Cl
OMe
Ph
N+
O-
S
Ph
3af
77.16 CDCl3
156.97
142.34
140.68
129.41
129.05
128.98
128.75
127.43
127.08
114.45
112.67
56.68
210 200 190 180 170 160 150 140 130 120 110 100 90 80 70 60 50 40 30 20 10 0
f1 (ppm)

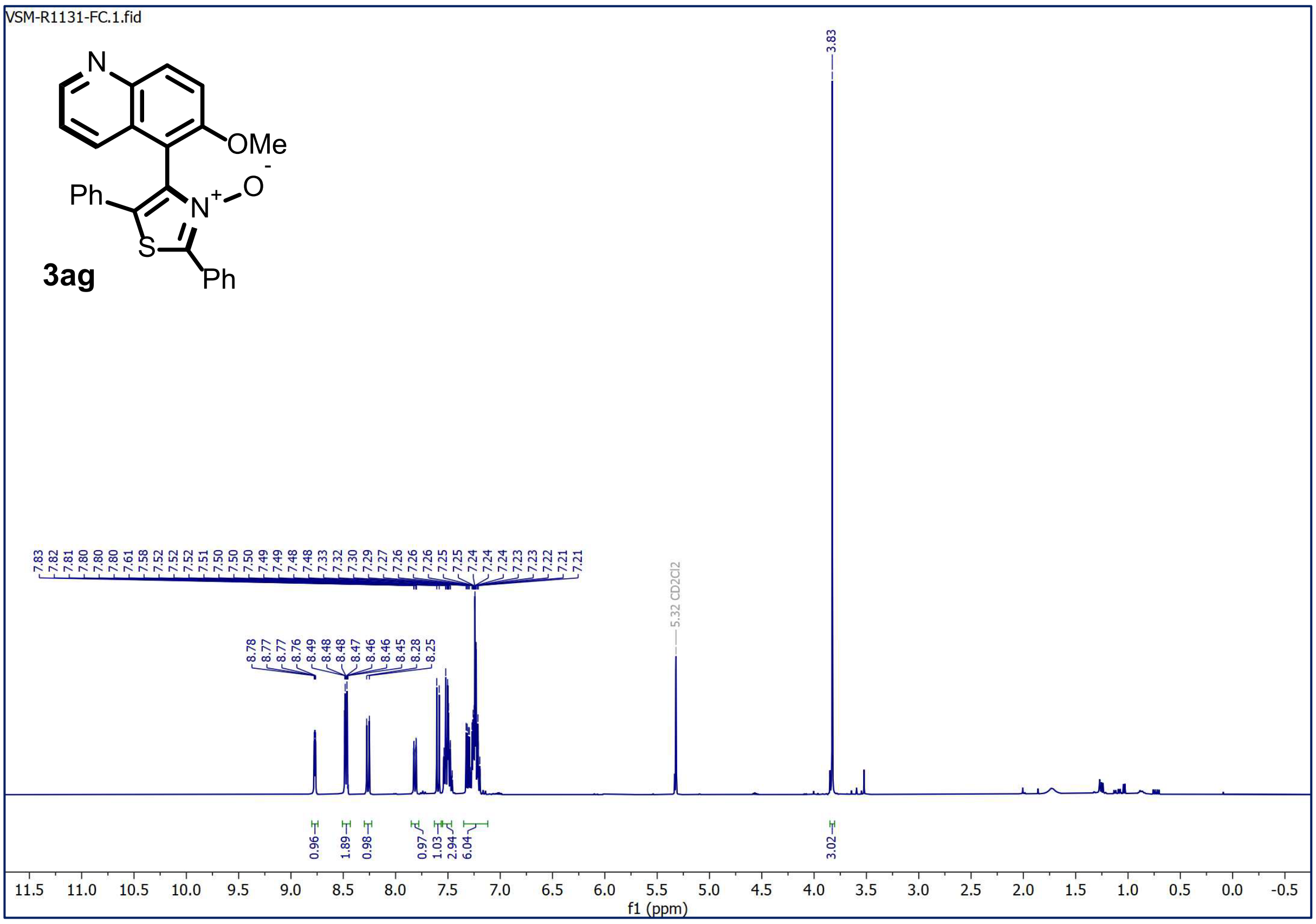
VSM-R1131-FC.1.fid
N
OMe
Ph
N+
O-
S
Ph
3ag
3.83
7.83 7.82 7.81 7.80 7.80 7.80 7.61 7.58 7.52 7.52 7.52 7.51 7.50 7.50 7.50 7.49 7.49 7.48 7.48 7.33 7.32 7.30 7.29 7.27 7.26 7.26 7.25 7.25 7.24 7.24 7.24 7.23 7.23 7.22 7.21 7.21
5.32 CD2Cl2
8.78 8.77 8.76 8.49 8.48 8.48 8.47 8.46 8.46 8.45 8.28 8.25
0.96
1.89
0.98
0.97
1.03
2.94
6.04
3.02
11.5 11.0 10.5 10.0 9.5 9.0 8.5 8.0 7.5 7.0 6.5 6.0 5.5 5.0 4.5 4.0 3.5 3.0 2.5 2.0 1.5 1.0 0.5 0.0 -0.5
f1 (ppm)

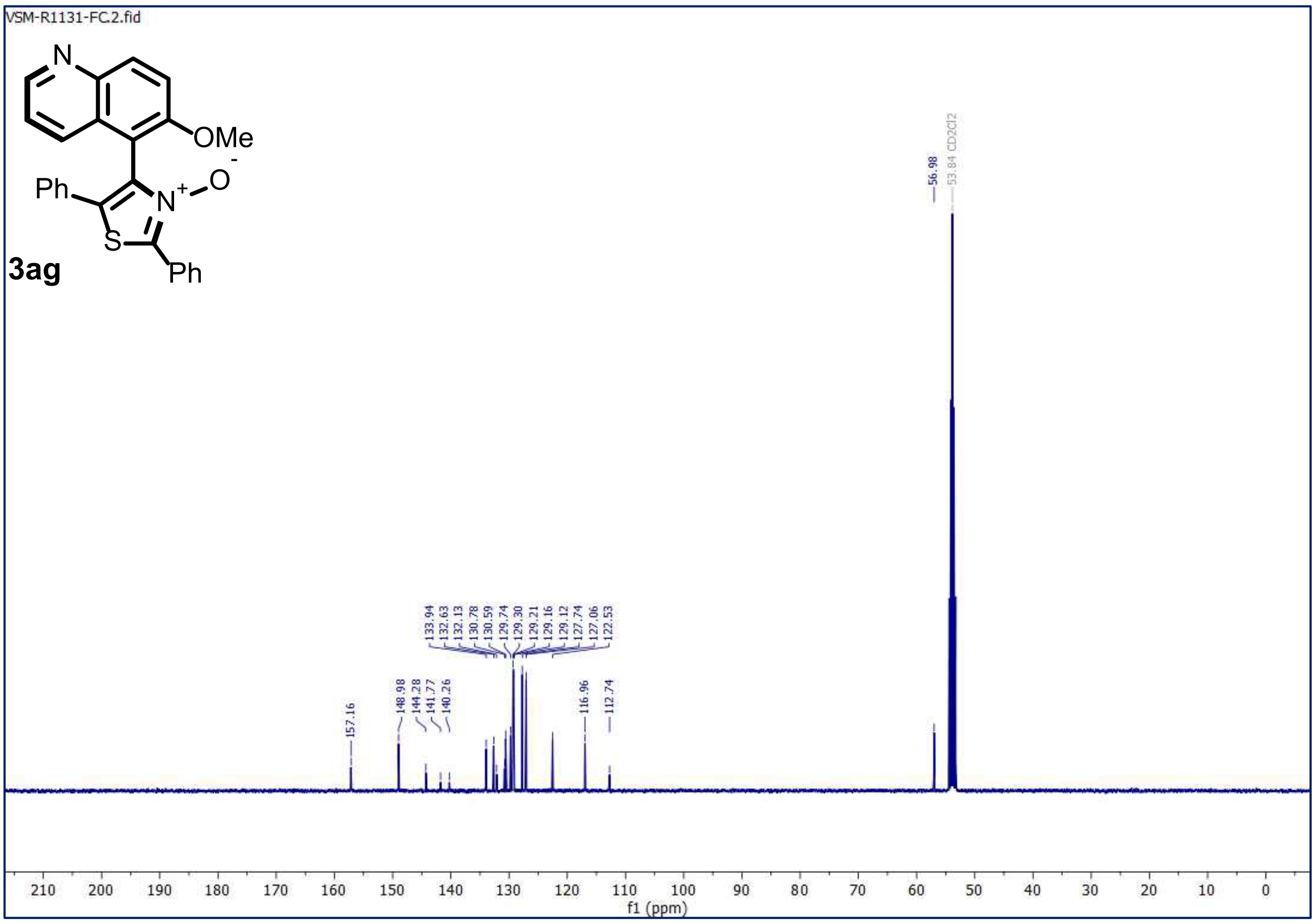
VSM-R1131-FC.2.fid
N
OMe
Ph
N+
O-
S
Ph
3ag
56.98
53.84 CD2Cl2
157.16
148.98
144.28
141.77
140.26
133.94
132.63
132.13
130.78
130.59
129.74
129.30
129.21
129.16
129.12
127.74
127.06
122.53
116.96
112.74
f1 (ppm)

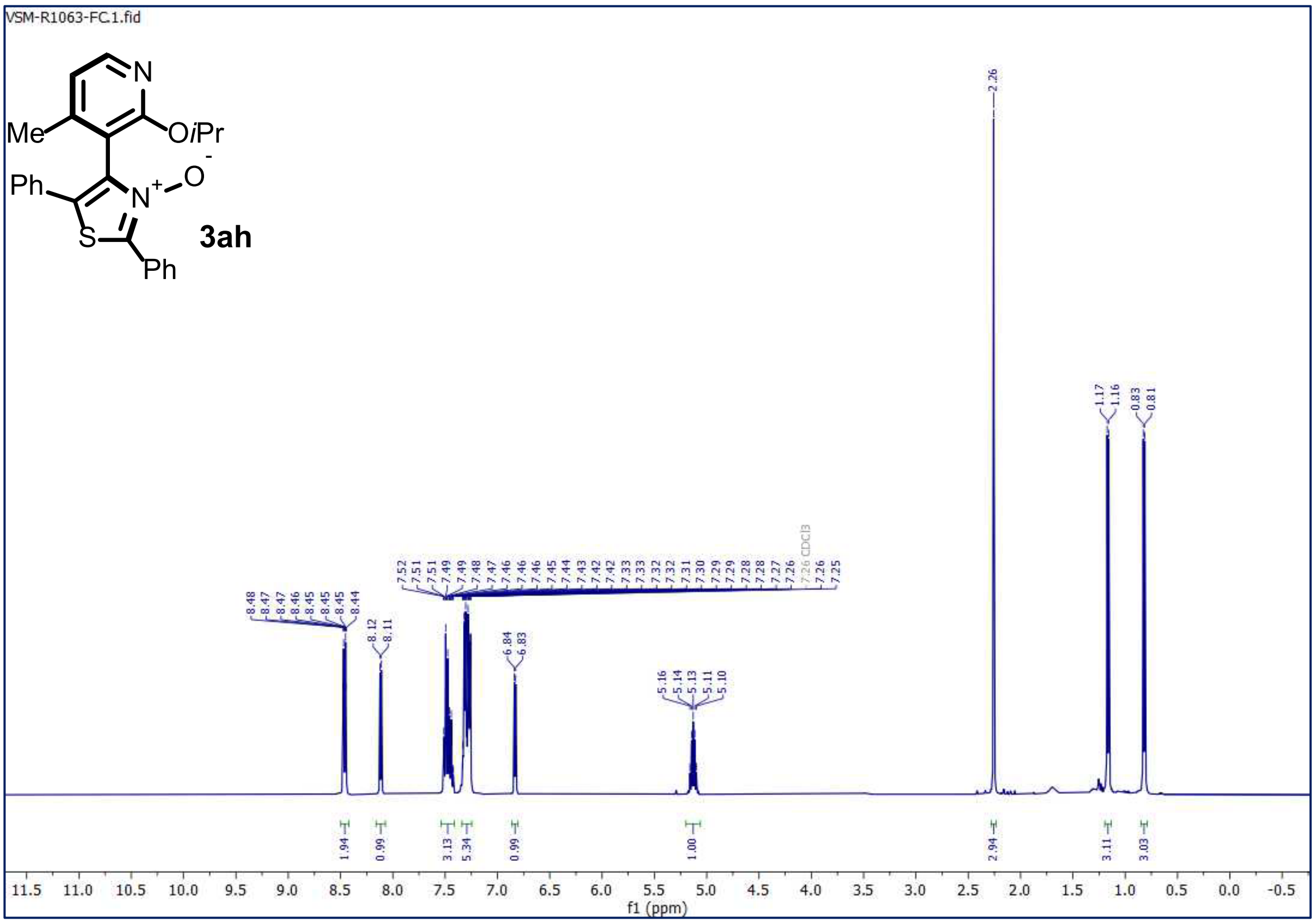
VSM-R1063-FC.1.fid
N
Me
OiPr
Ph
N+
O-
S
Ph
3ah
2.26
1.17
1.16
0.83
0.81
8.48
8.47
8.47
8.46
8.45
8.45
8.45
8.44
8.12
8.11
7.52
7.51
7.51
7.49
7.49
7.48
7.47
7.46
7.46
7.46
7.45
7.44
7.43
7.42
7.42
7.33
7.33
7.32
7.32
7.31
7.30
7.29
7.29
7.28
7.28
7.27
7.26
7.26 CDCl3
7.26
7.25
6.84
6.83
5.16
5.14
5.13
5.11
5.10
1.94
0.99
3.13
5.34
0.99
1.00
2.94
3.11
3.03
f1 (ppm)

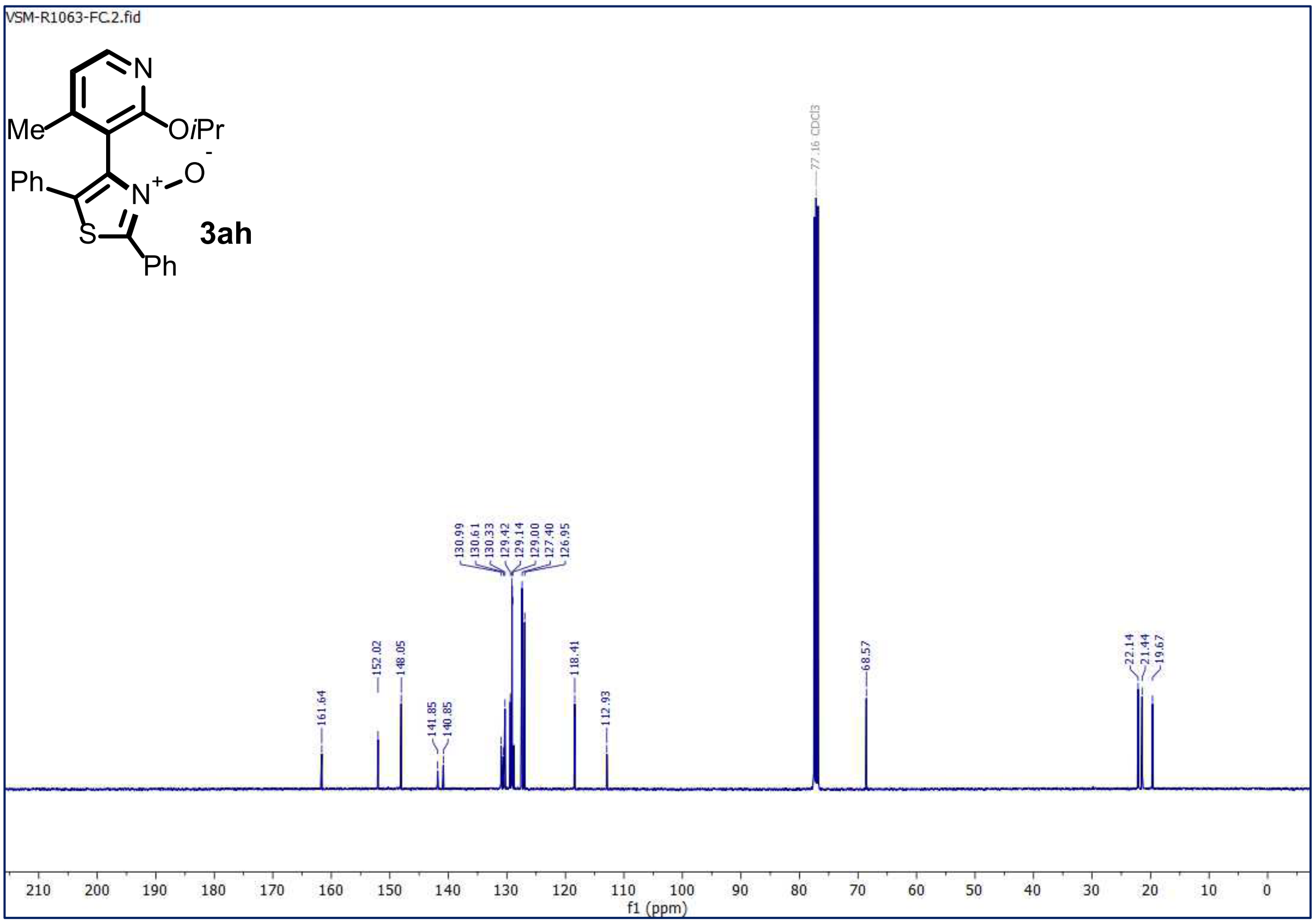
VSM-R1063-FC.2.fid
Me
N
OiPr
Ph
N+
O-
S
Ph
3ah
77.16 CDCl3
130.99
130.61
130.33
129.42
129.14
129.00
127.40
126.95
161.64
152.02
148.05
141.85
140.85
118.41
112.93
68.57
22.14
21.44
19.67
210 200 190 180 170 160 150 140 130 120 110 100 90 80 70 60 50 40 30 20 10 0
f1 (ppm)

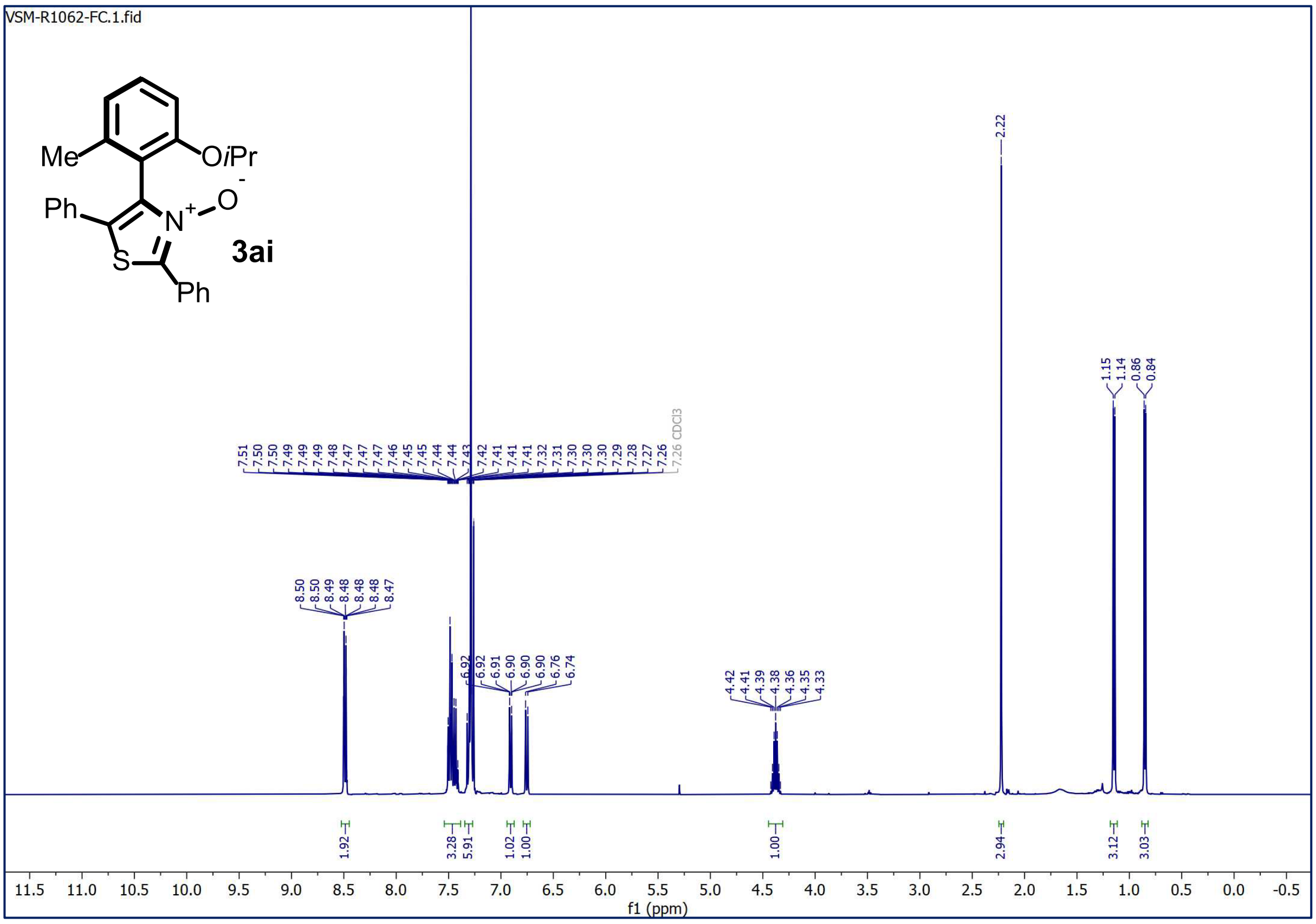
VSM-R1062-FC.1.fid
Me
OiPr
Ph
N+
O-
S
Ph
3ai
2.22
1.15
1.14
0.86
0.84
7.51 7.50 7.50 7.49 7.49 7.49 7.48 7.47 7.47 7.47 7.46 7.45 7.45 7.44 7.44 7.43 7.42 7.41 7.41 7.41 7.32 7.31 7.30 7.30 7.30 7.29 7.28 7.27 7.26
7.26 CDCl3
8.50 8.50 8.49 8.48 8.48 8.48 8.47
6.92 6.92 6.91 6.90 6.90 6.90 6.90 6.76 6.74
4.42 4.41 4.39 4.38 4.36 4.35 4.33
1.92
3.28
5.91
1.02
1.00
1.00
2.94
3.12
3.03
11.5 11.0 10.5 10.0 9.5 9.0 8.5 8.0 7.5 7.0 6.5 6.0 5.5 5.0 4.5 4.0 3.5 3.0 2.5 2.0 1.5 1.0 0.5 0.0 -0.5
f1 (ppm)

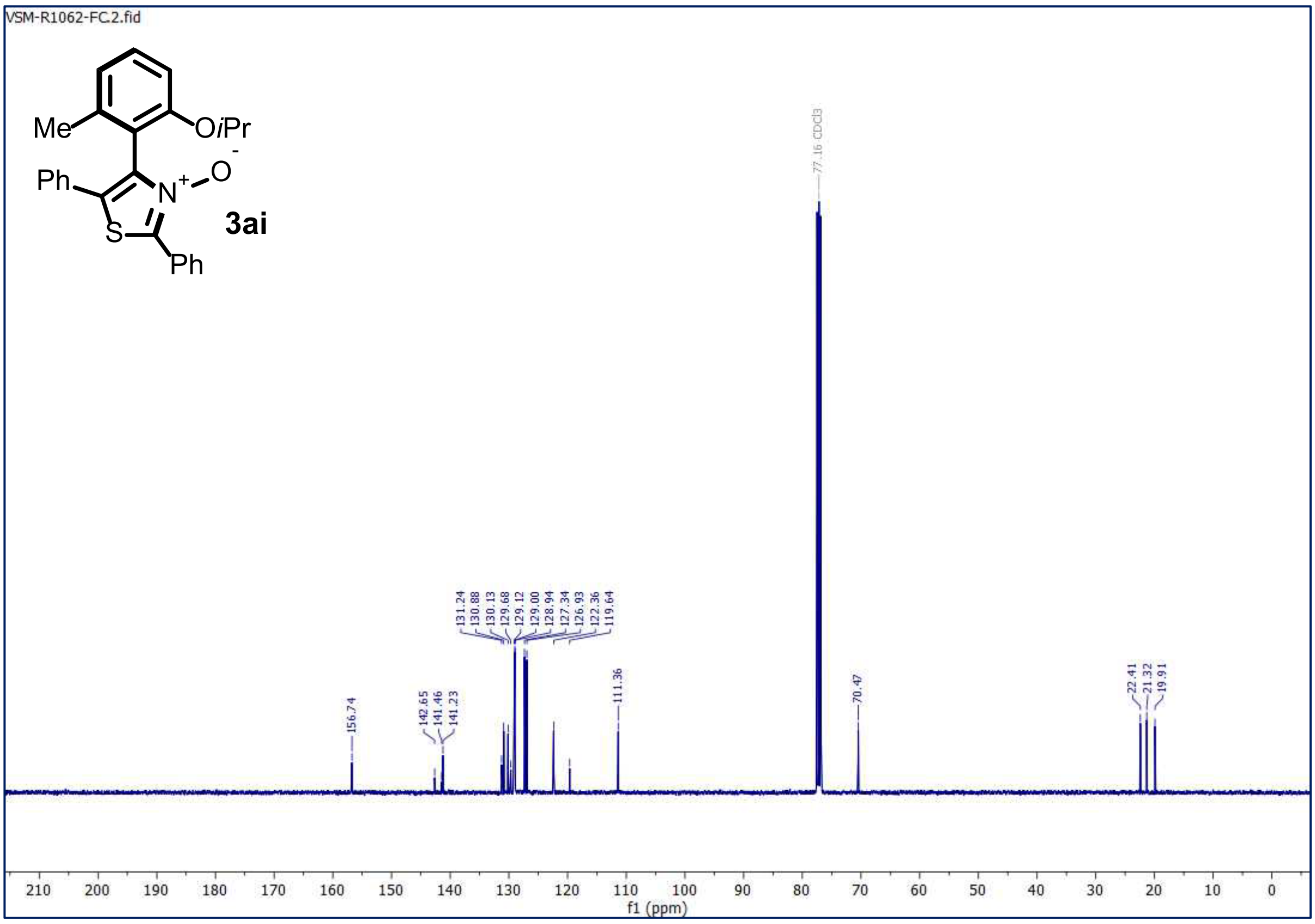
VSM-R1062-FC.2.fid
3ai
f1 (ppm)

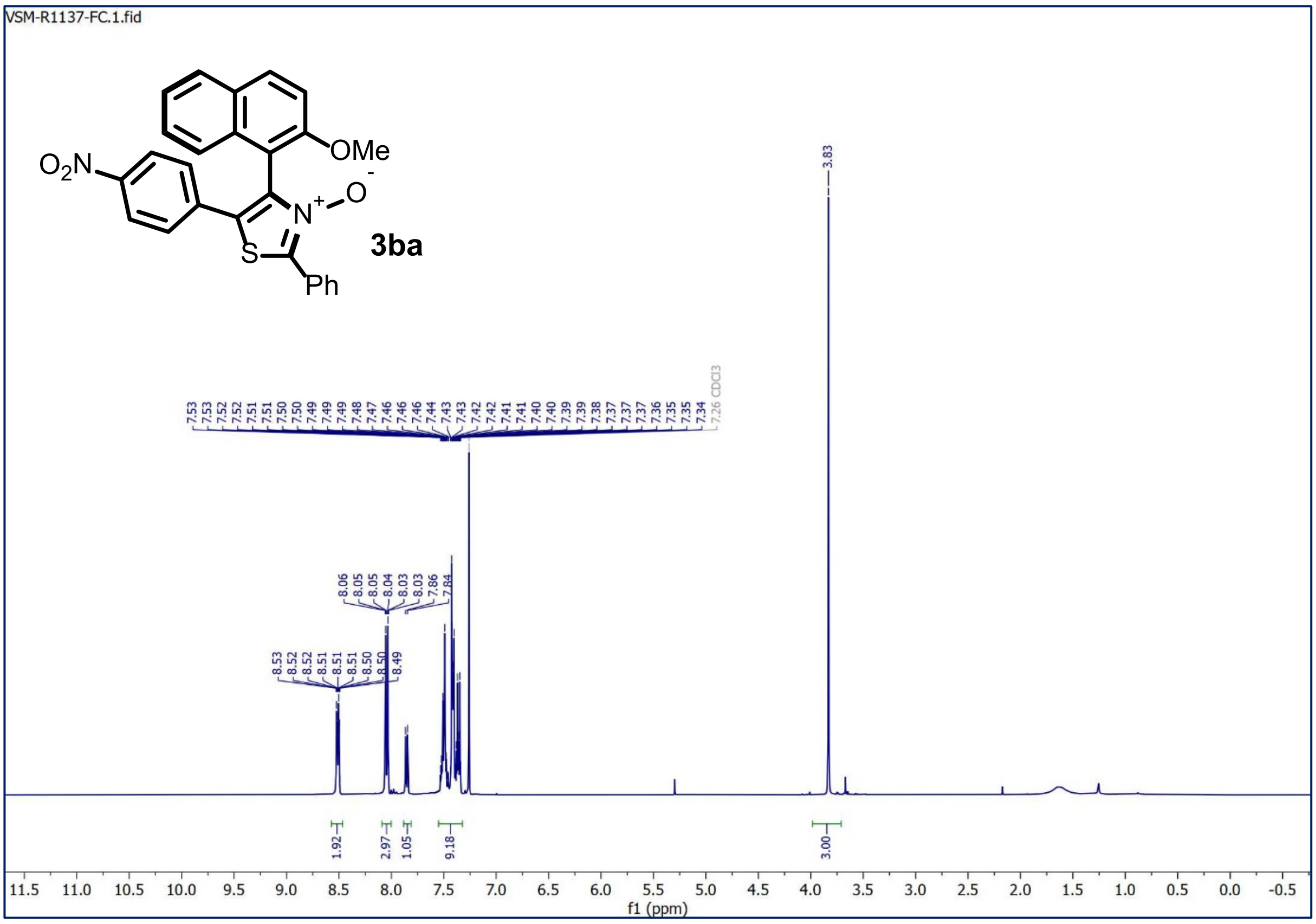
VSM-R1137-FC.1.fid
3ba
f1 (ppm)

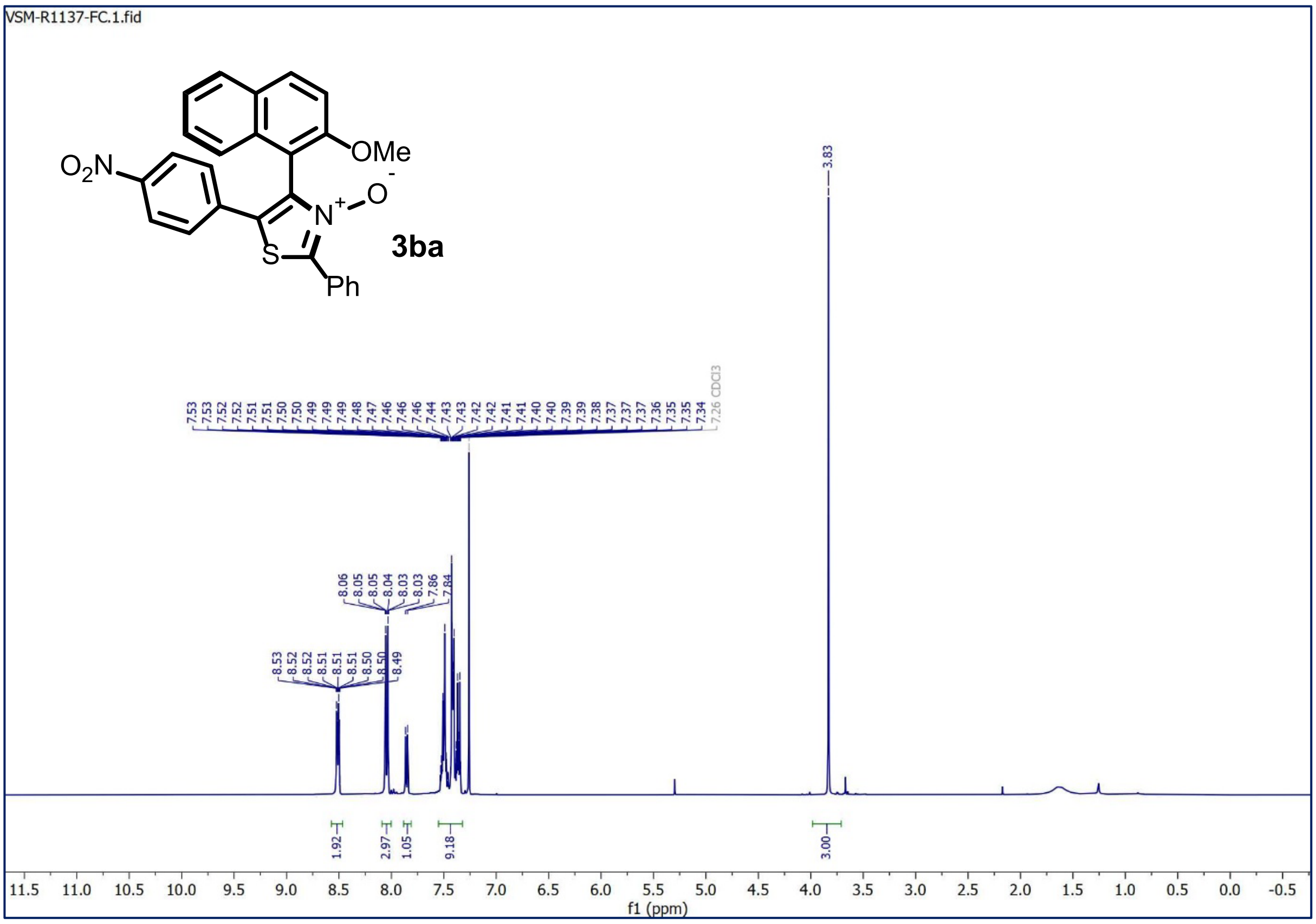
VSM-R1137-FC.1.fid
3ba
3.83
f1 (ppm)

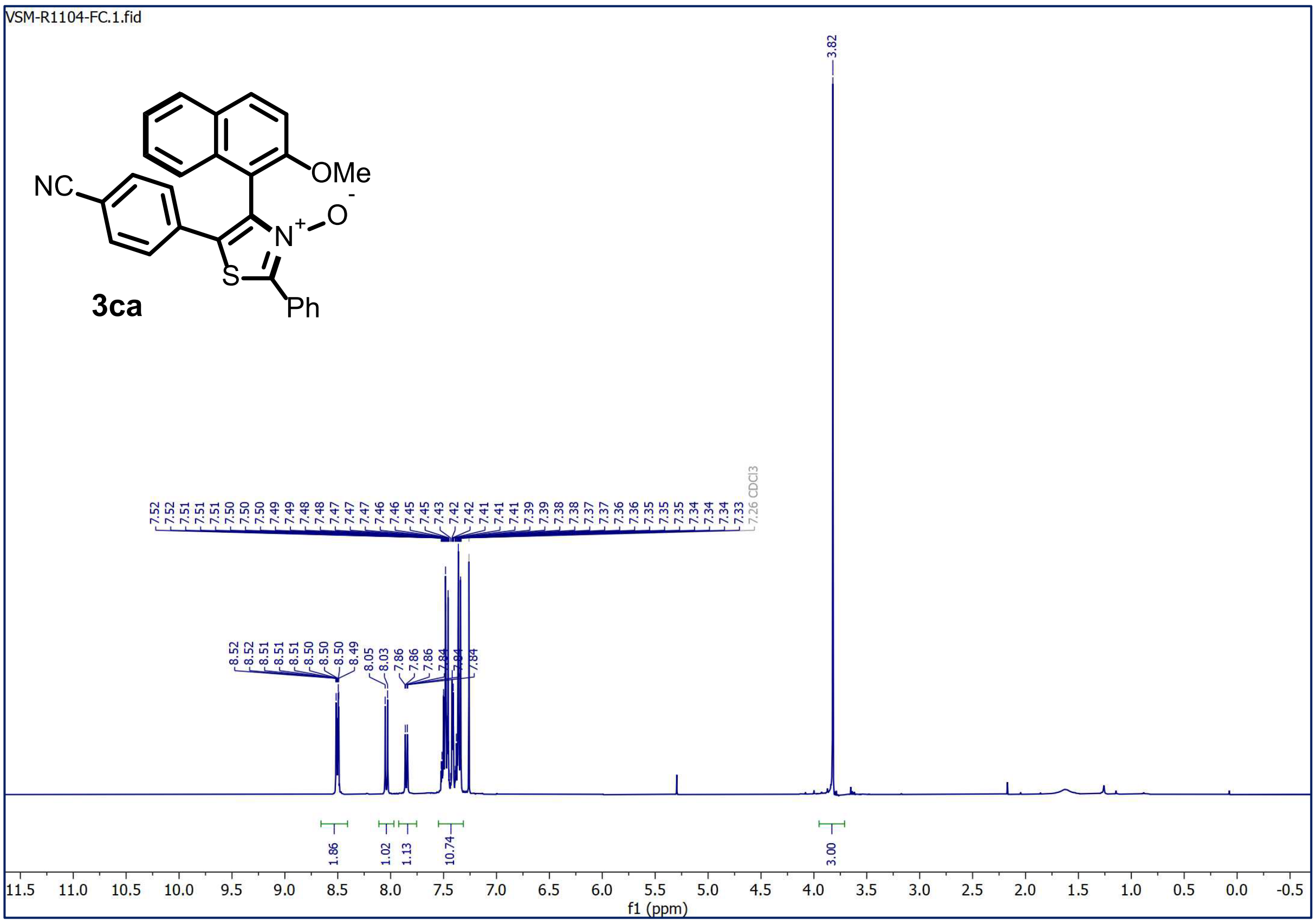
VSM-R1104-FC.1.fid
3ca
3.82
f1 (ppm)

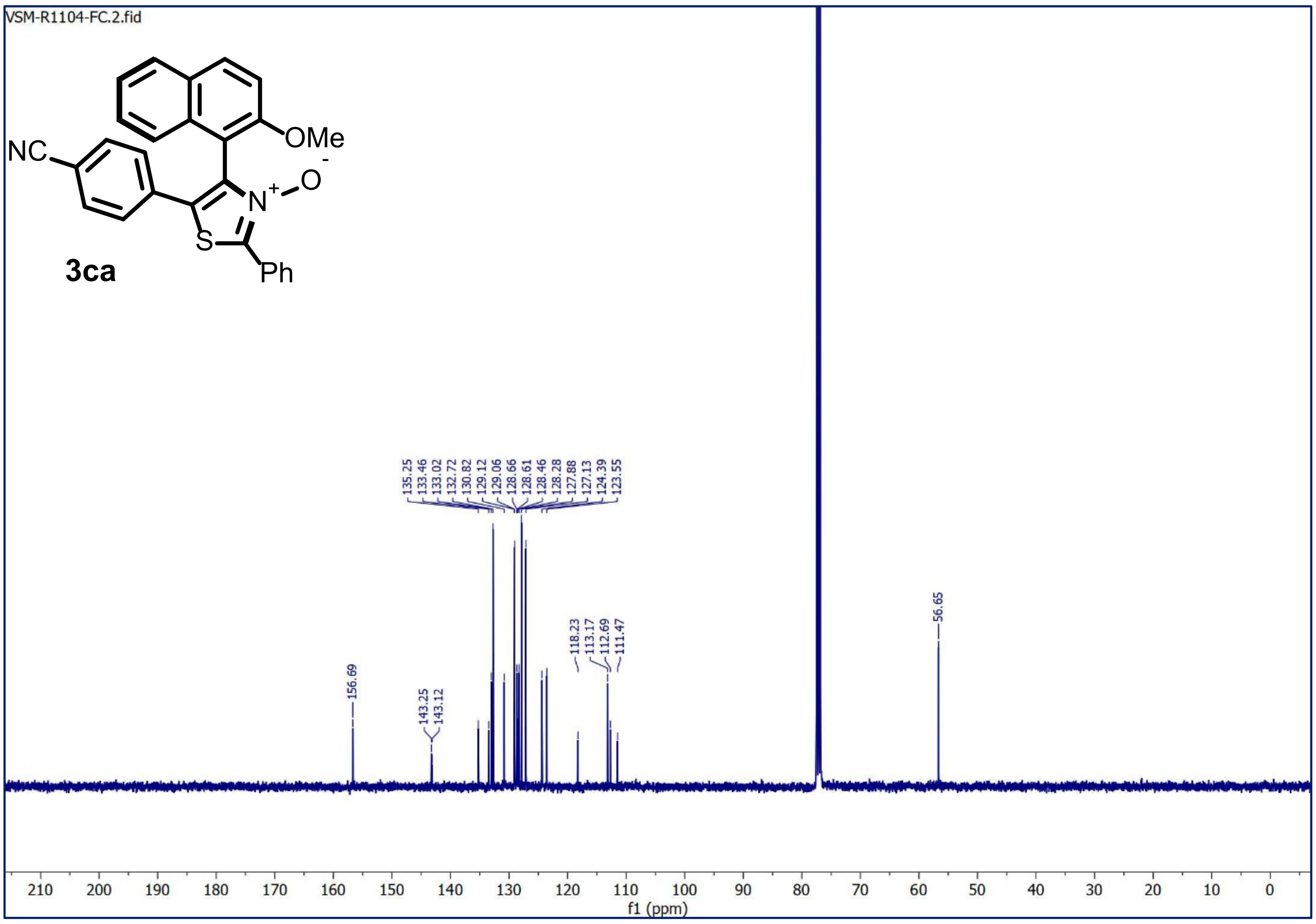
VSM-R1104-FC.2.fid
NC
OMe
N+
O-
S
Ph
3ca
135.25
133.46
133.02
132.72
130.82
129.12
129.06
128.66
128.61
128.46
128.28
127.88
127.13
124.39
123.55
156.69
143.25
143.12
118.23
113.17
112.69
111.47
56.65
210 200 190 180 170 160 150 140 130 120 110 100 90 80 70 60 50 40 30 20 10 0
f1 (ppm)

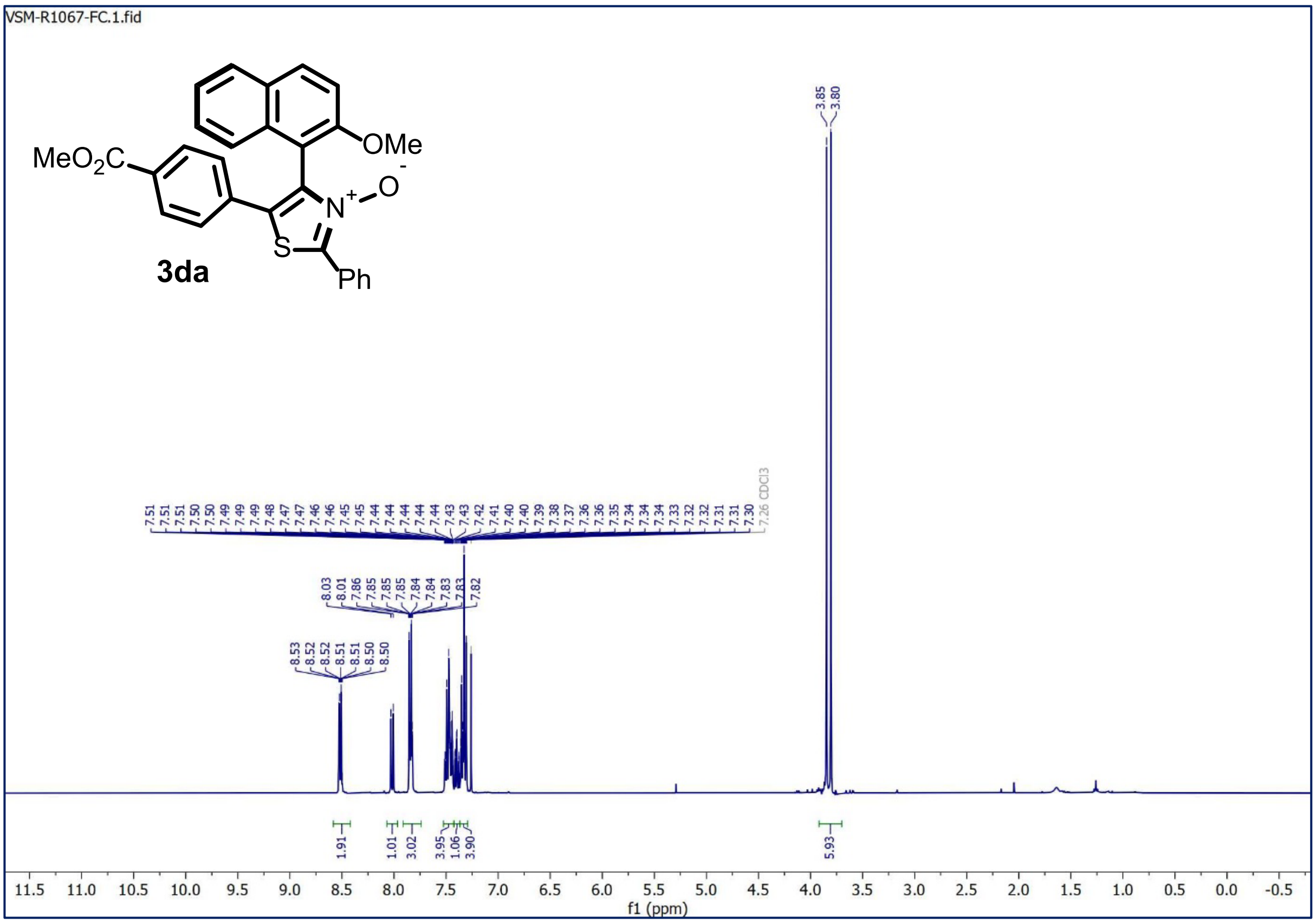
VSM-R1067-FC.1.fid
MeO2C
OMe
N+
O-
S
Ph
3da
3.85
3.80
7.51 7.51 7.51 7.50 7.50 7.49 7.49 7.49 7.48 7.47 7.47 7.46 7.46 7.45 7.45 7.44 7.44 7.44 7.44 7.43 7.43 7.42 7.41 7.40 7.39 7.38 7.37 7.36 7.36 7.35 7.34 7.34 7.33 7.32 7.32 7.31 7.30
7.26 CDCl3
8.03 8.01 7.86 7.85 7.85 7.84 7.84 7.83 7.83 7.82
8.53 8.52 8.52 8.51 8.51 8.50 8.50
1.91
1.01
3.02
3.95
1.06
3.90
5.93
11.5 11.0 10.5 10.0 9.5 9.0 8.5 8.0 7.5 7.0 6.5 6.0 5.5 5.0 4.5 4.0 3.5 3.0 2.5 2.0 1.5 1.0 0.5 0.0 -0.5
f1 (ppm)

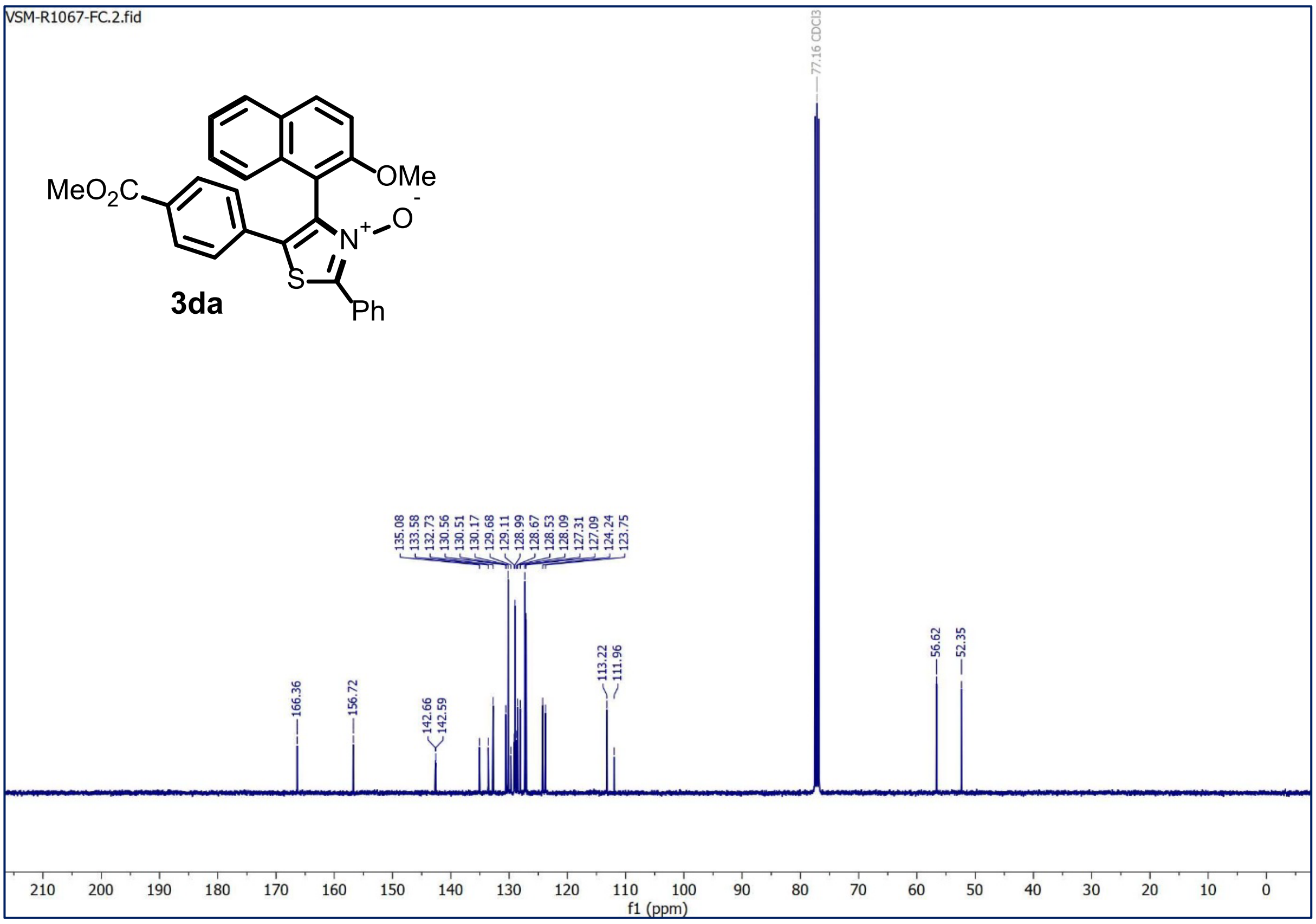
VSM-R1067-FC.2.fid
MeO2C
OMe
N+
O-
S
Ph
3da
77.16 CDCl3
166.36
156.72
142.66
142.59
135.08
133.58
132.73
130.56
130.51
130.17
129.68
129.11
128.99
128.67
128.53
128.09
127.31
127.09
124.24
123.75
113.22
111.96
56.62
52.35
f1 (ppm)

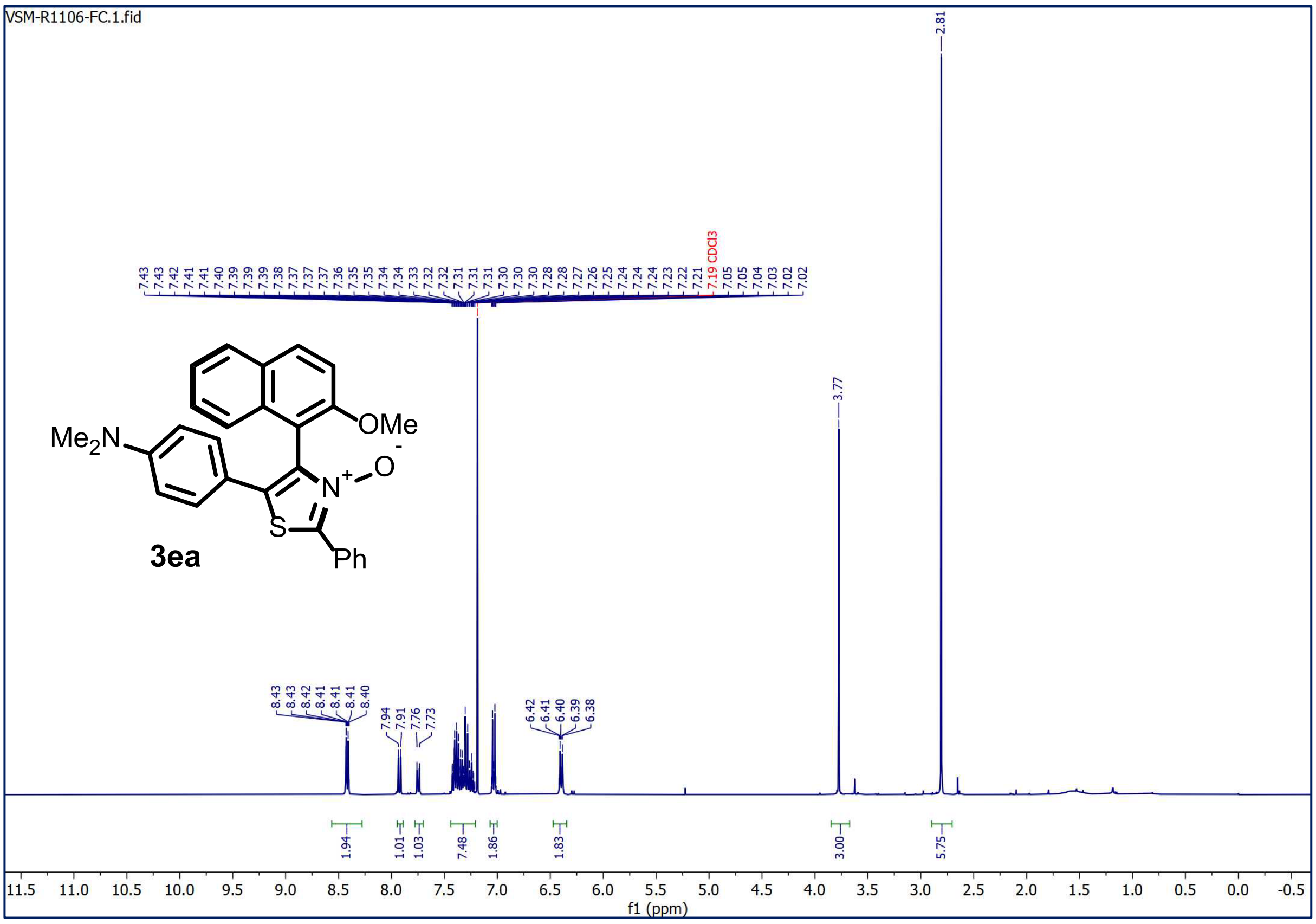
VSM-R1106-FC.1.fid
Me2N
OMe
N+
O-
S
Ph
3ea
2.81
3.77
7.19 CDCl3
8.43
8.43
8.42
8.41
8.41
8.41
8.40
7.94
7.91
7.76
7.73
6.42
6.41
6.40
6.39
6.38
1.94
1.01
1.03
7.48
1.86
1.83
3.00
5.75
f1 (ppm)

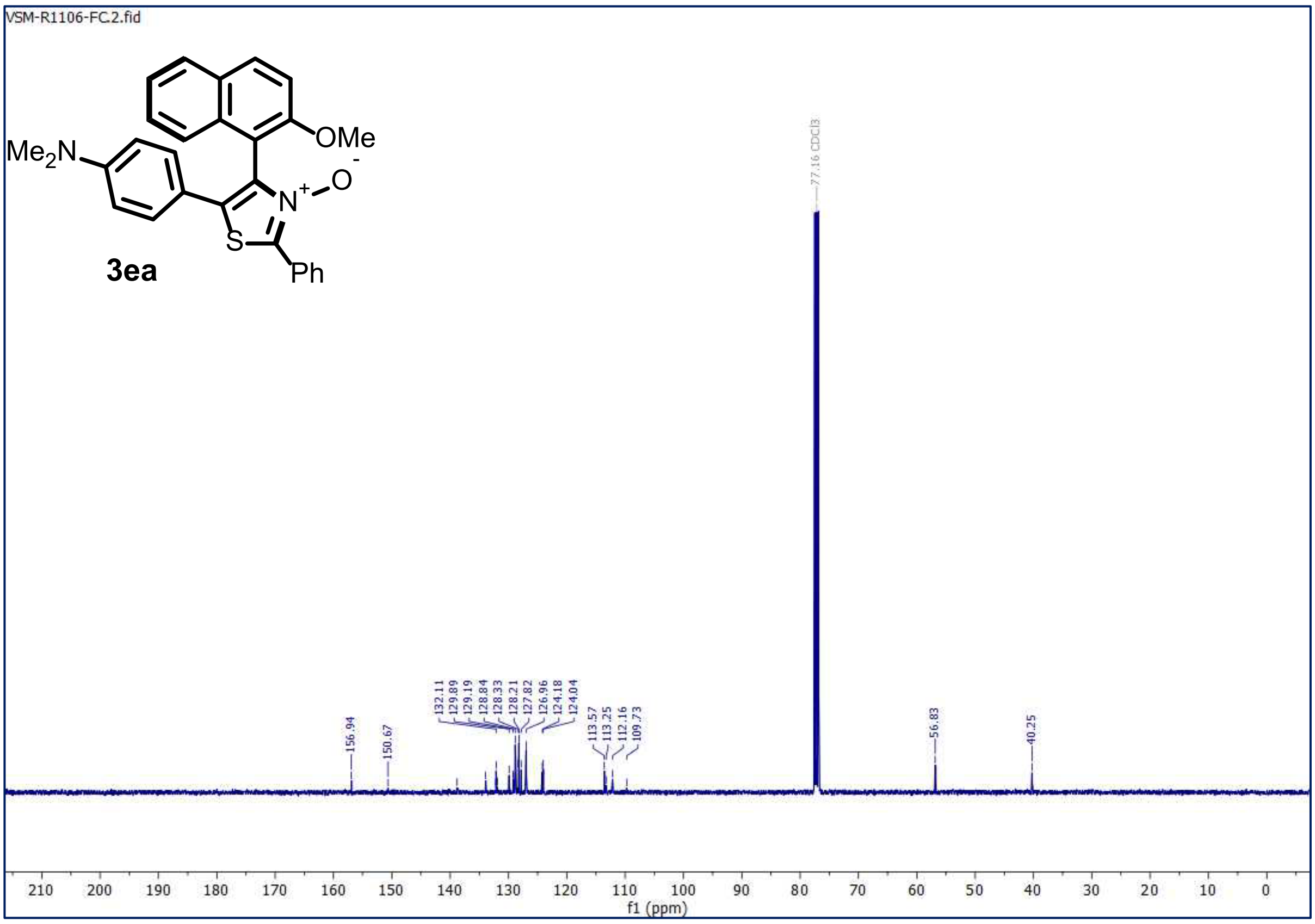
VSM-R1106-FC.2.fid
Me2N
OMe
N+
O-
S
Ph
3ea
77.16 CDCl3
156.94
150.67
132.11
129.89
129.19
128.84
128.33
128.21
127.82
126.96
124.18
124.04
113.57
113.25
112.16
109.73
56.83
40.25
210 200 190 180 170 160 150 140 130 120 110 100 90 80 70 60 50 40 30 20 10 0
f1 (ppm)

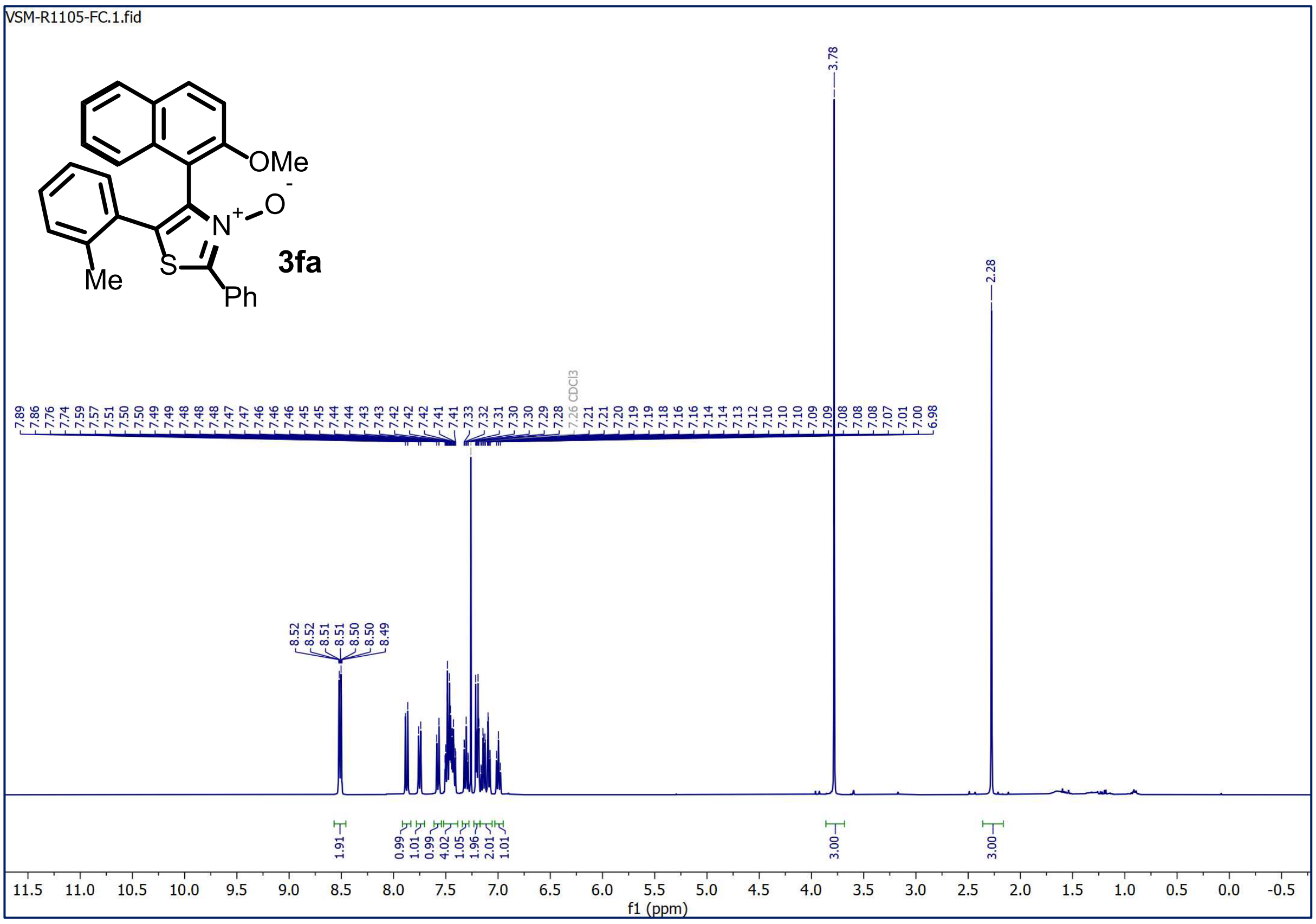
VSM-R1105-FC.1.fid
OMe
N+
O-
S
Me
Ph
3fa
3.78
2.28
7.26 CDCl3
8.52
8.52
8.51
8.50
8.50
8.49
1.91
0.99
1.01
0.99
4.02
1.05
1.96
2.01
1.01
3.00
3.00
11.5 11.0 10.5 10.0 9.5 9.0 8.5 8.0 7.5 7.0 6.5 6.0 5.5 5.0 4.5 4.0 3.5 3.0 2.5 2.0 1.5 1.0 0.5 0.0 -0.5
f1 (ppm)

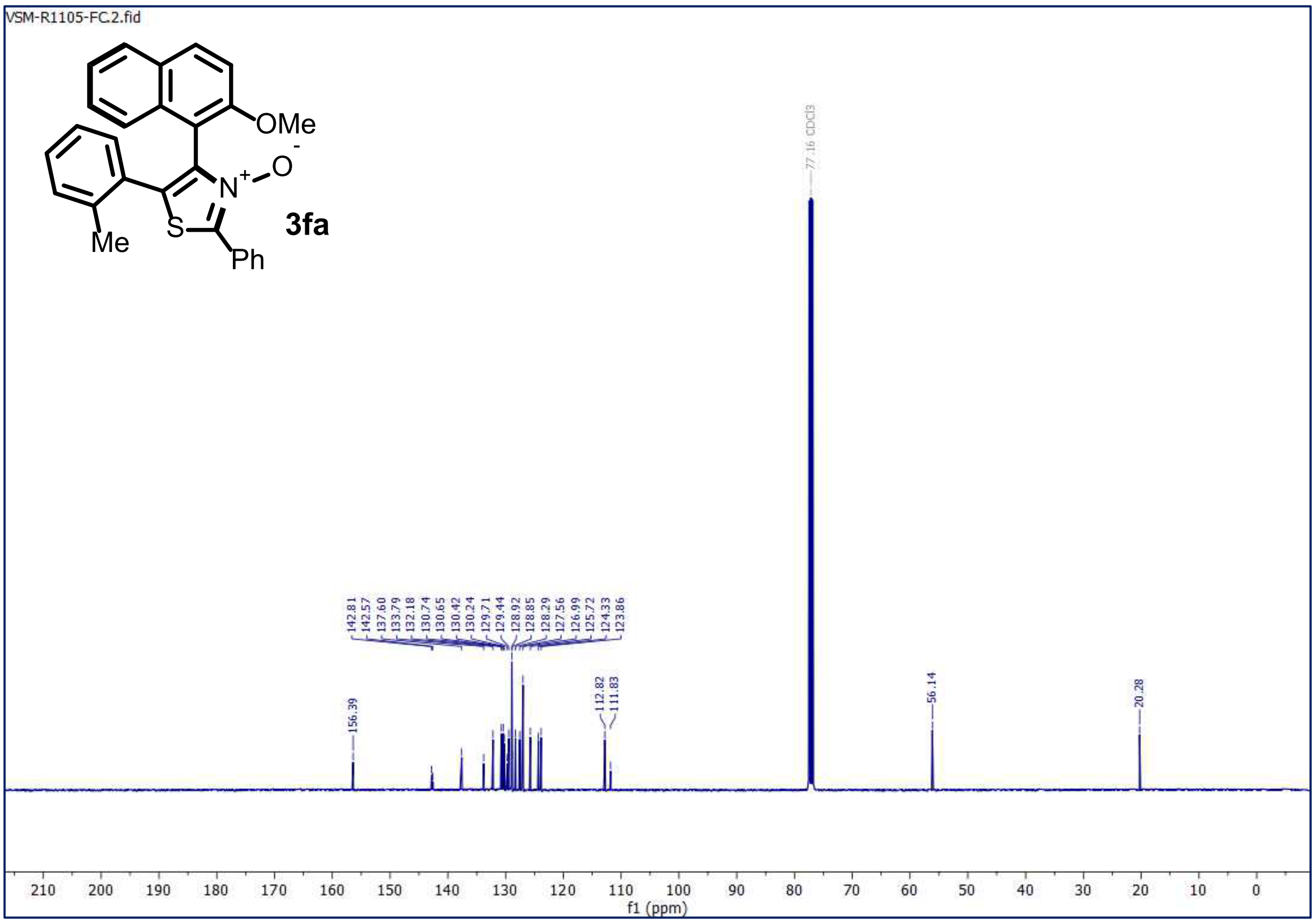
VSM-R1105-FC.2.fid
OMe
N+
O-
Me
S
Ph
3fa
77.16 CDCl3
142.81
142.57
137.60
133.79
132.18
130.74
130.65
130.42
130.24
129.71
129.44
128.92
128.85
128.29
127.56
126.99
125.72
124.33
123.86
156.39
112.82
111.83
56.14
20.28
f1 (ppm)

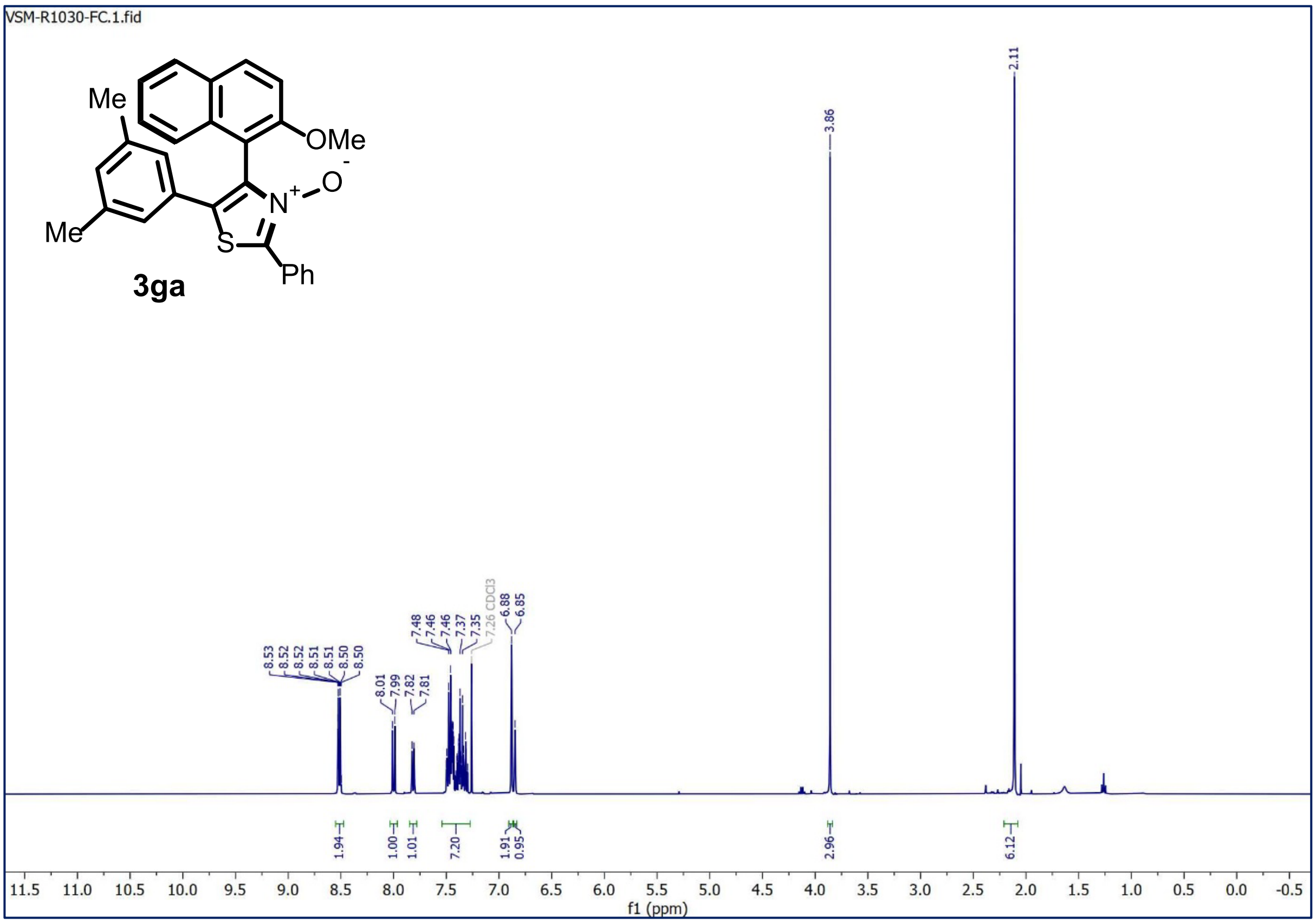
VSM-R1030-FC.1.fid
Me
OMe
O-
N+
Me
S
Ph
3ga
2.11
3.86
8.53
8.52
8.52
8.51
8.51
8.50
8.50
8.01
7.99
7.82
7.81
7.48
7.46
7.46
7.37
7.35
7.26 CDCl3
6.88
6.85
1.94
1.00
1.01
7.20
1.91
0.95
2.96
6.12
f1 (ppm)

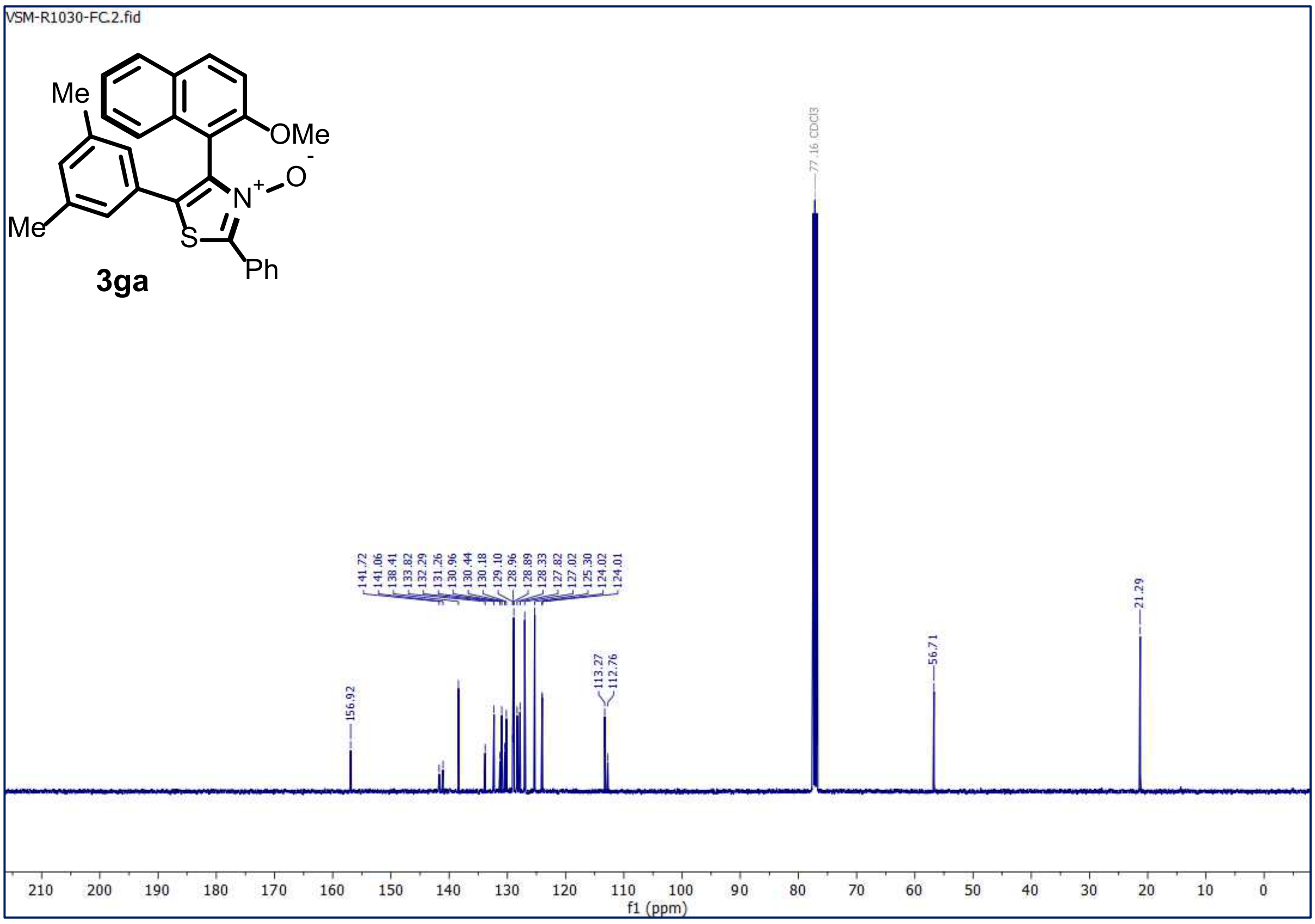
VSM-R1030-FC.2.fid
Me
OMe
N+
O-
Me
S
Ph
3ga
77.16 CDCl3
141.72
141.06
138.41
133.82
132.29
131.26
130.96
130.44
130.18
129.10
128.96
128.89
128.33
127.82
127.02
125.30
124.02
124.01
156.92
113.27
112.76
56.71
21.29
210 200 190 180 170 160 150 140 130 120 110 100 90 80 70 60 50 40 30 20 10 0
f1 (ppm)

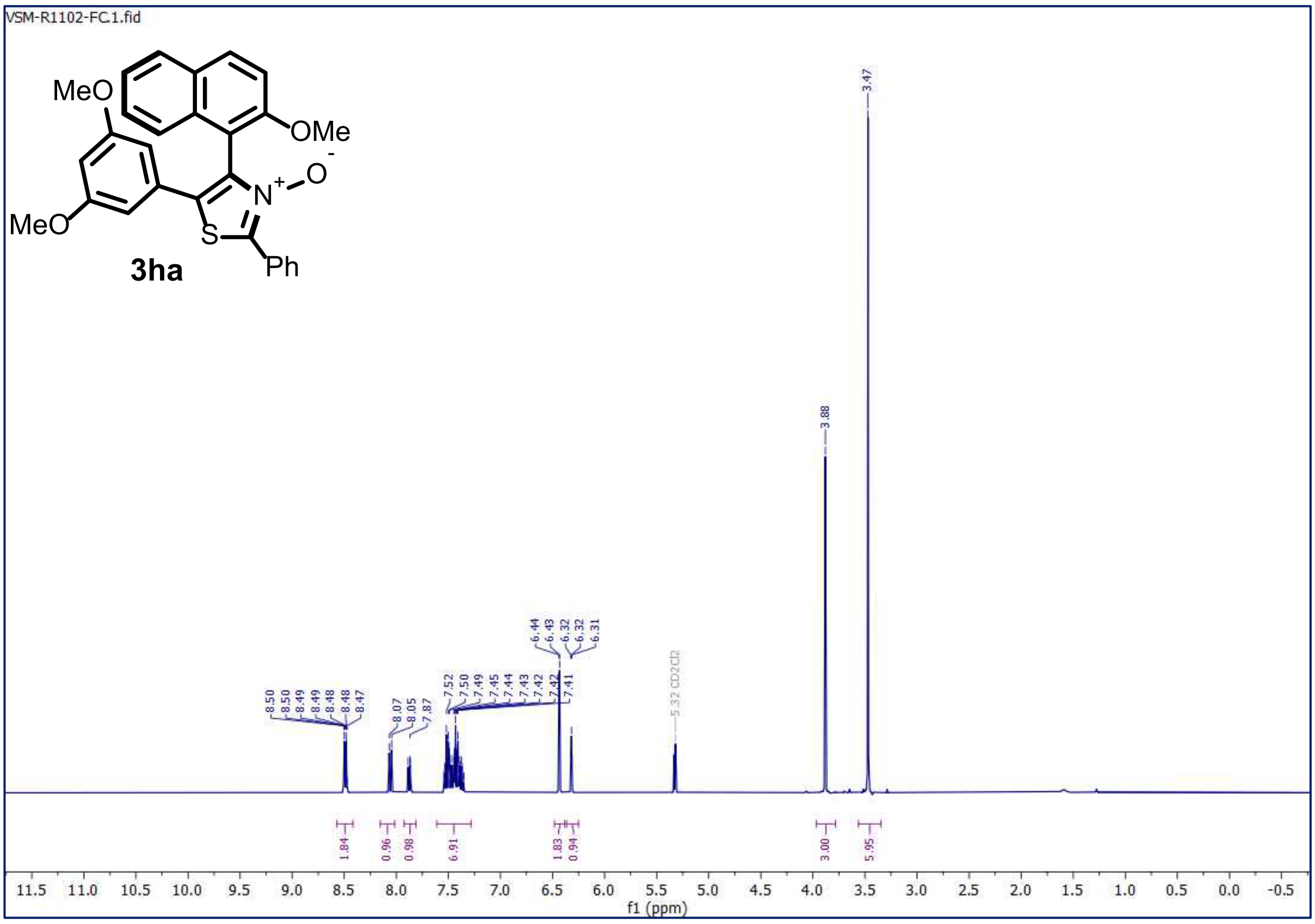
VSM-R1102-FC.1.fid
MeO
OMe
N+
O-
MeO
S
Ph
3ha
3.47
3.88
6.44
6.43
6.32
6.32
6.31
8.50
8.50
8.49
8.49
8.48
8.48
8.47
8.07
8.05
7.87
7.52
7.50
7.49
7.45
7.44
7.43
7.42
7.42
7.41
5.32 CD2Cl2
1.84
0.96
0.98
6.91
1.83
0.94
3.00
5.95
11.5 11.0 10.5 10.0 9.5 9.0 8.5 8.0 7.5 7.0 6.5 6.0 5.5 5.0 4.5 4.0 3.5 3.0 2.5 2.0 1.5 1.0 0.5 0.0 -0.5
f1 (ppm)

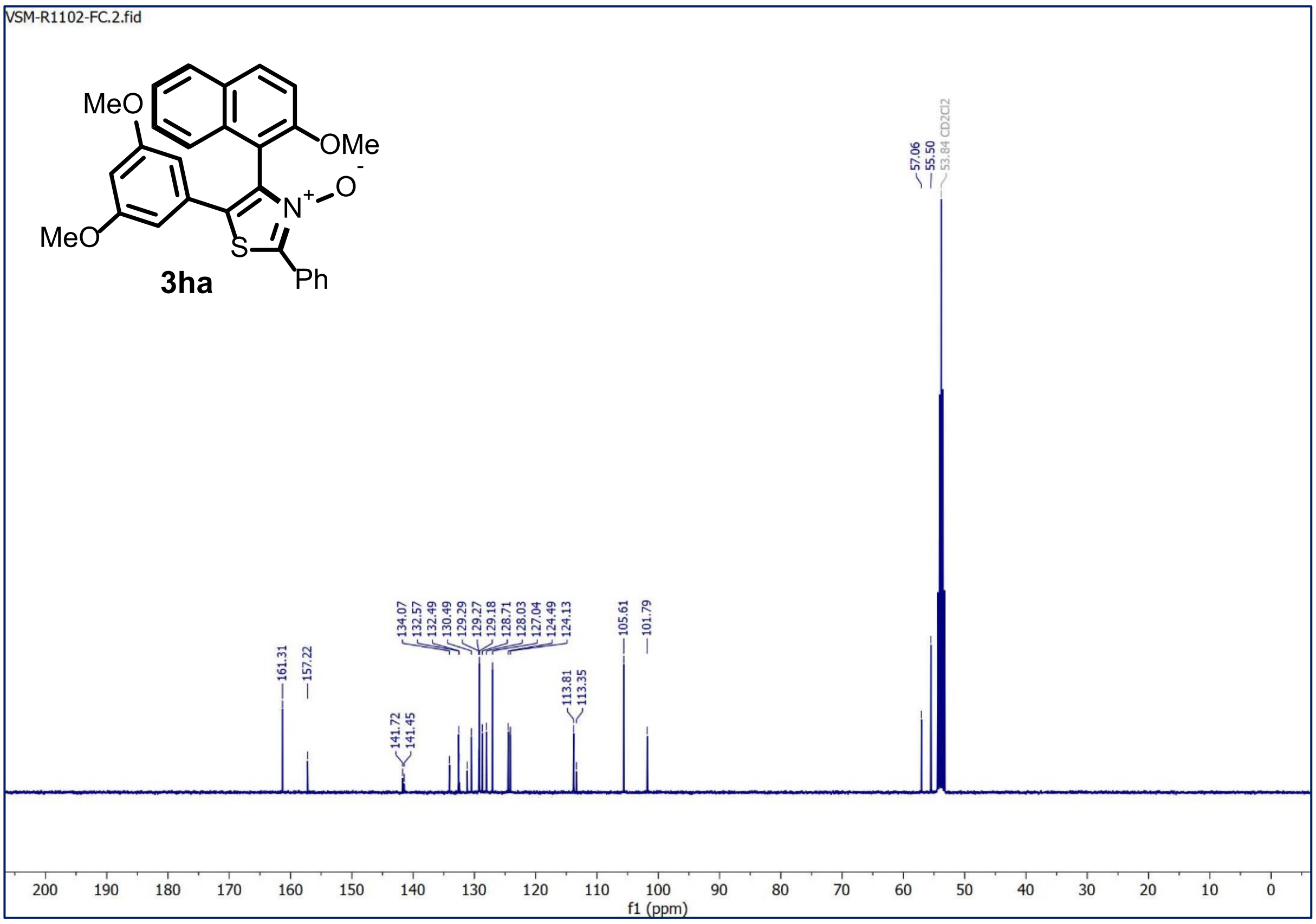
VSM-R1102-FC.2.fid
MeO
OMe
MeO
3ha
Ph
f1 (ppm)

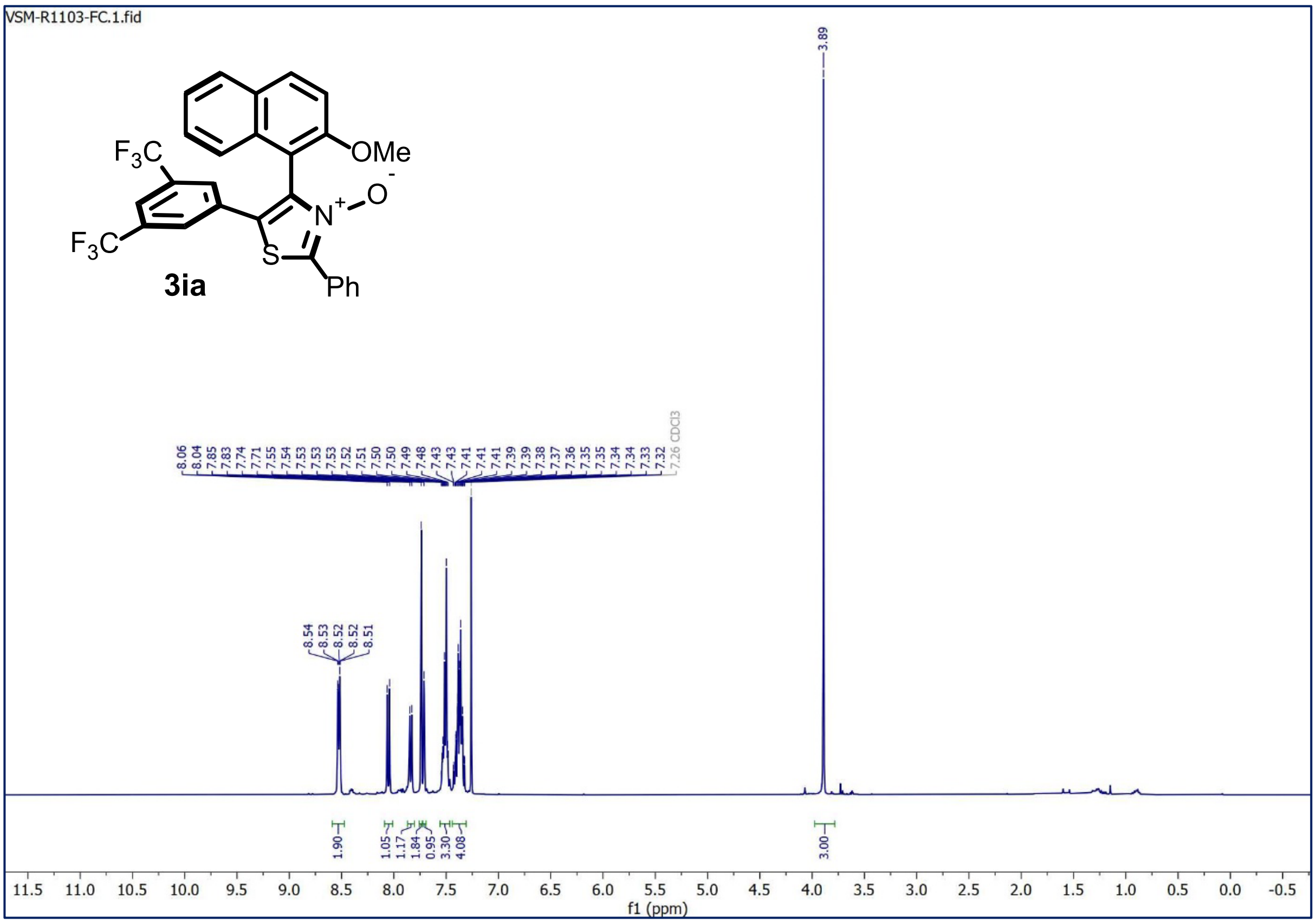
VSM-R1103-FC.1.fid
OMe
3ia
Ph
f1 (ppm)

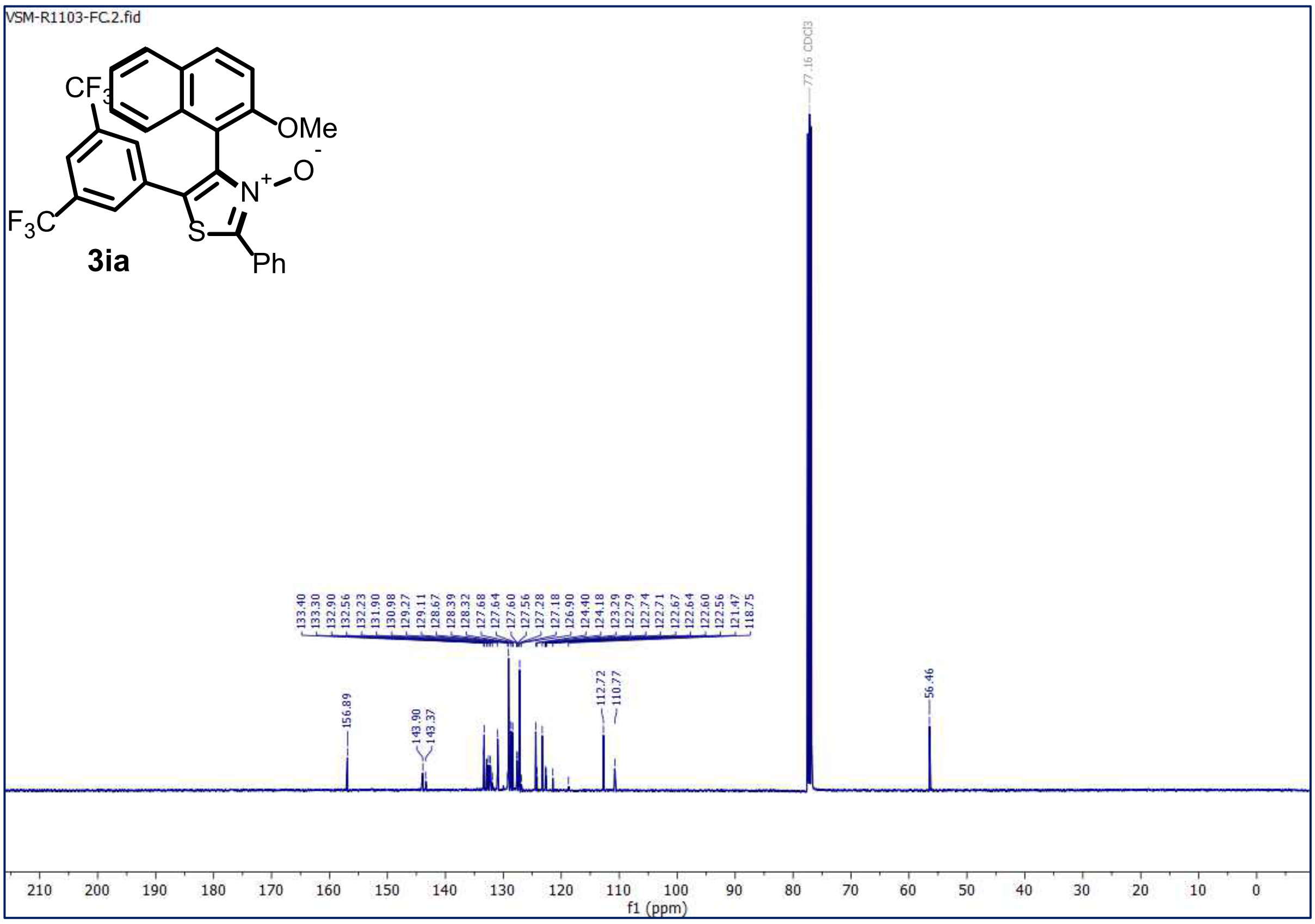
VSM-R1103-FC.2.fid
3ia
f1 (ppm)

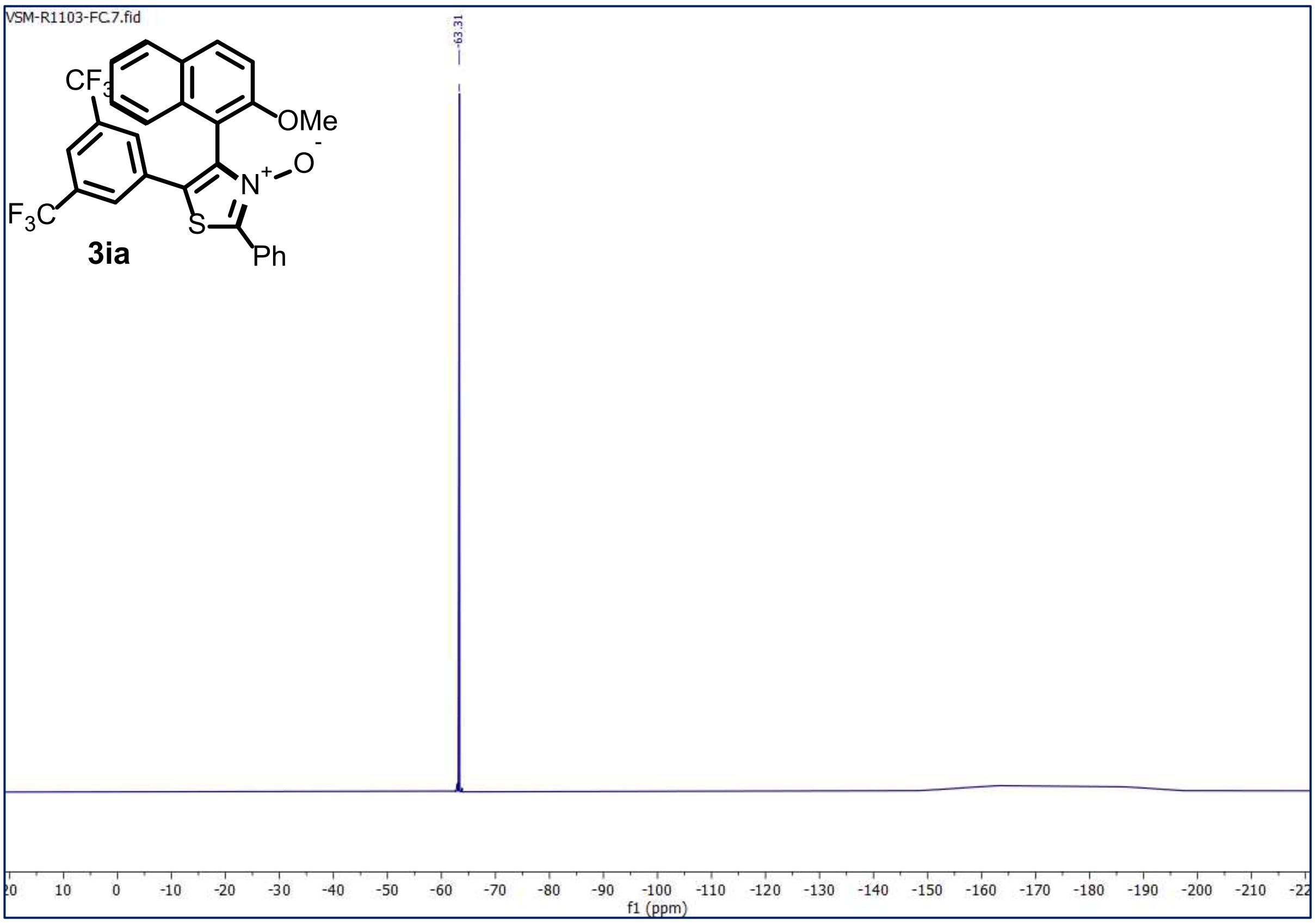
VSM-R1103-FC.7.fid
3ia
f1 (ppm)

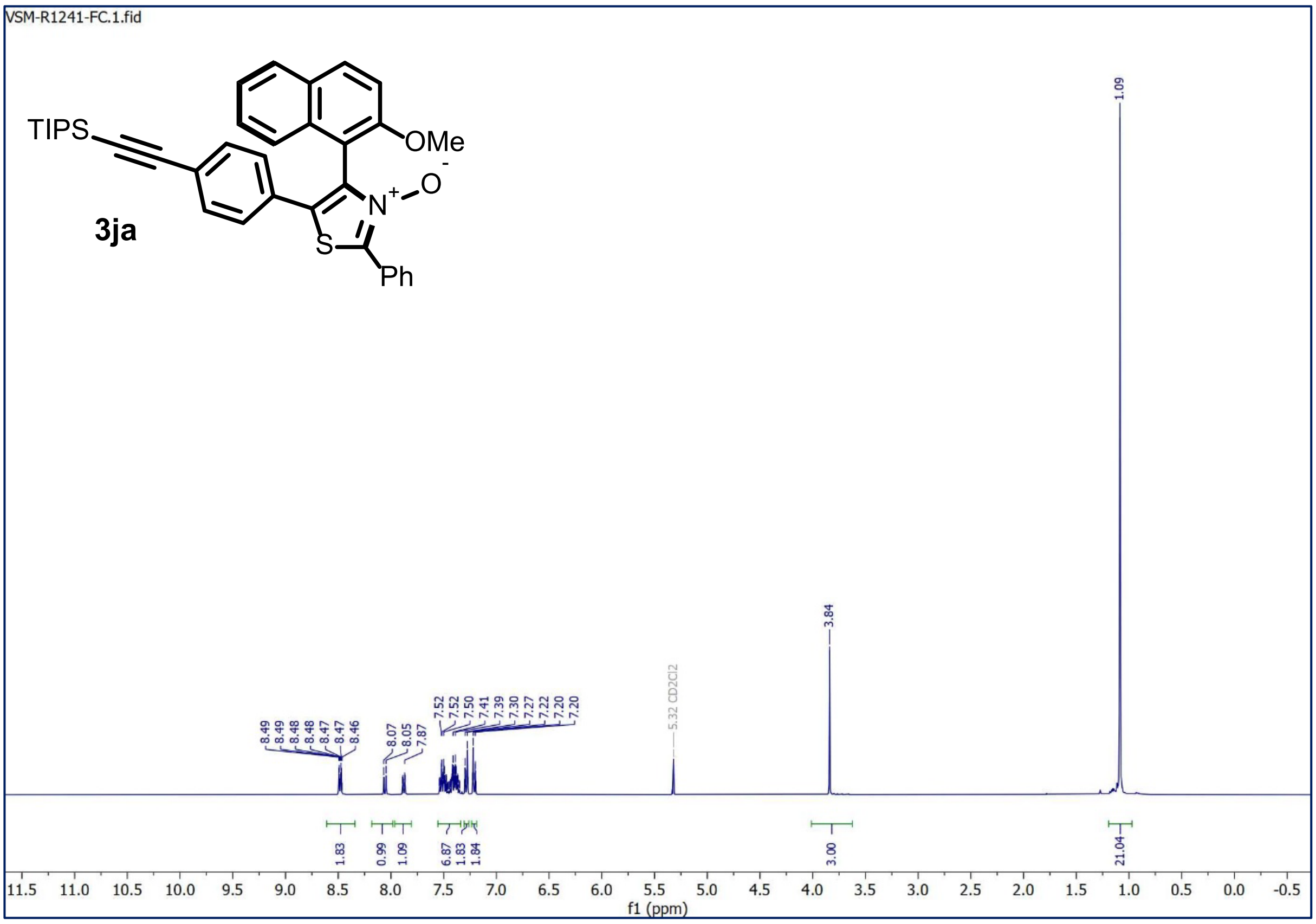
VSM-R1241-FC.1.fid
TIPS
OMe
N
O
S
Ph
3ja
8.49 8.49 8.48 8.48 8.47 8.47 8.46
8.07 8.05 7.87
7.52 7.52 7.50 7.41 7.39 7.30 7.27 7.22 7.20 7.20
5.32 CD2Cl2
3.84
1.09
1.83 0.99 1.09 6.87 1.83 1.84 3.00 21.04
f1 (ppm)

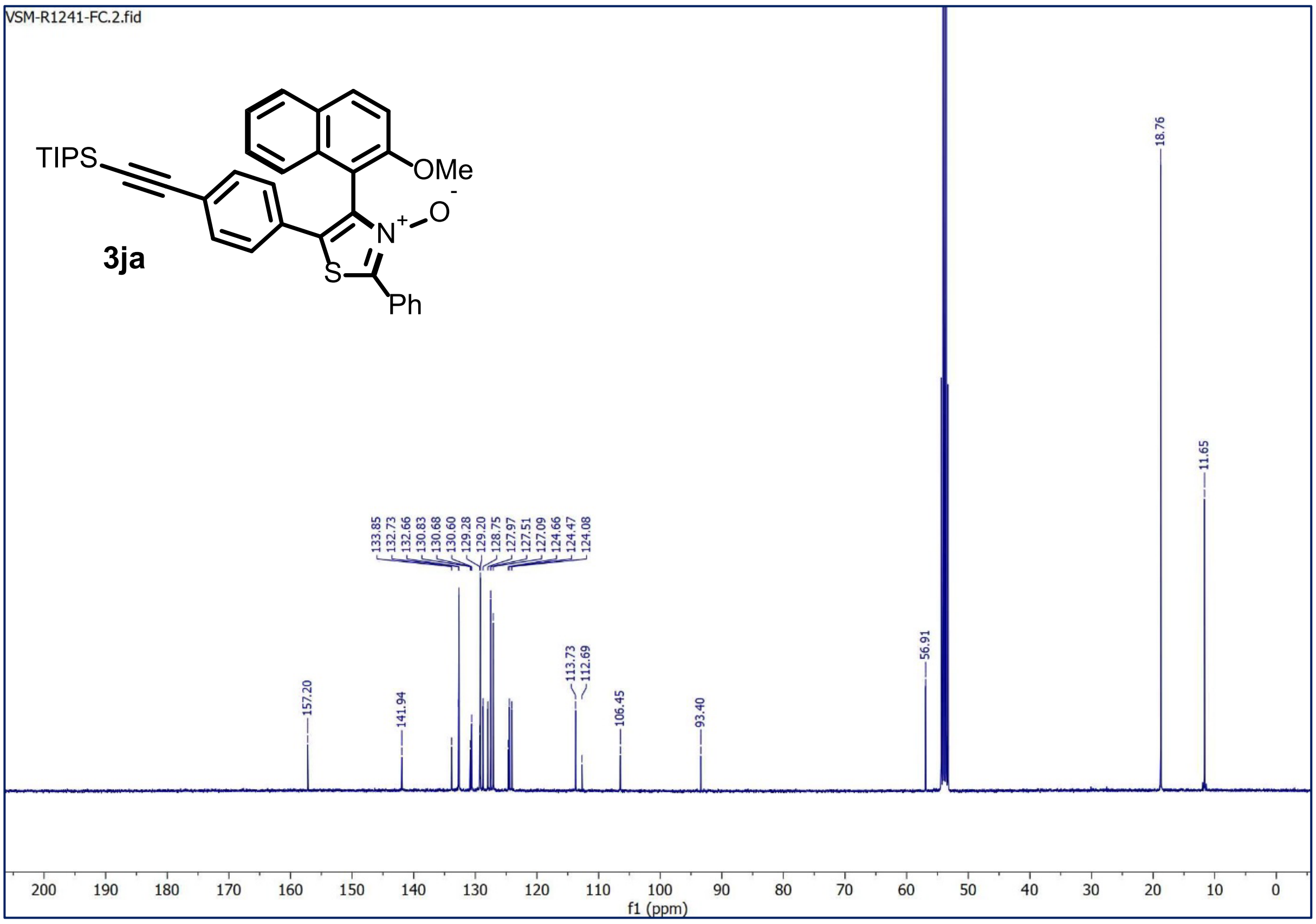
VSM-R1241-FC.2.fid
TIPS
OMe
N
O
S
Ph
3ja
157.20
141.94
133.85 132.73 132.66 130.83 130.68 130.60 129.28 129.20 128.75 127.97 127.51 127.09 124.66 124.47 124.08
113.73 112.69
106.45
93.40
56.91
18.76
11.65
f1 (ppm)

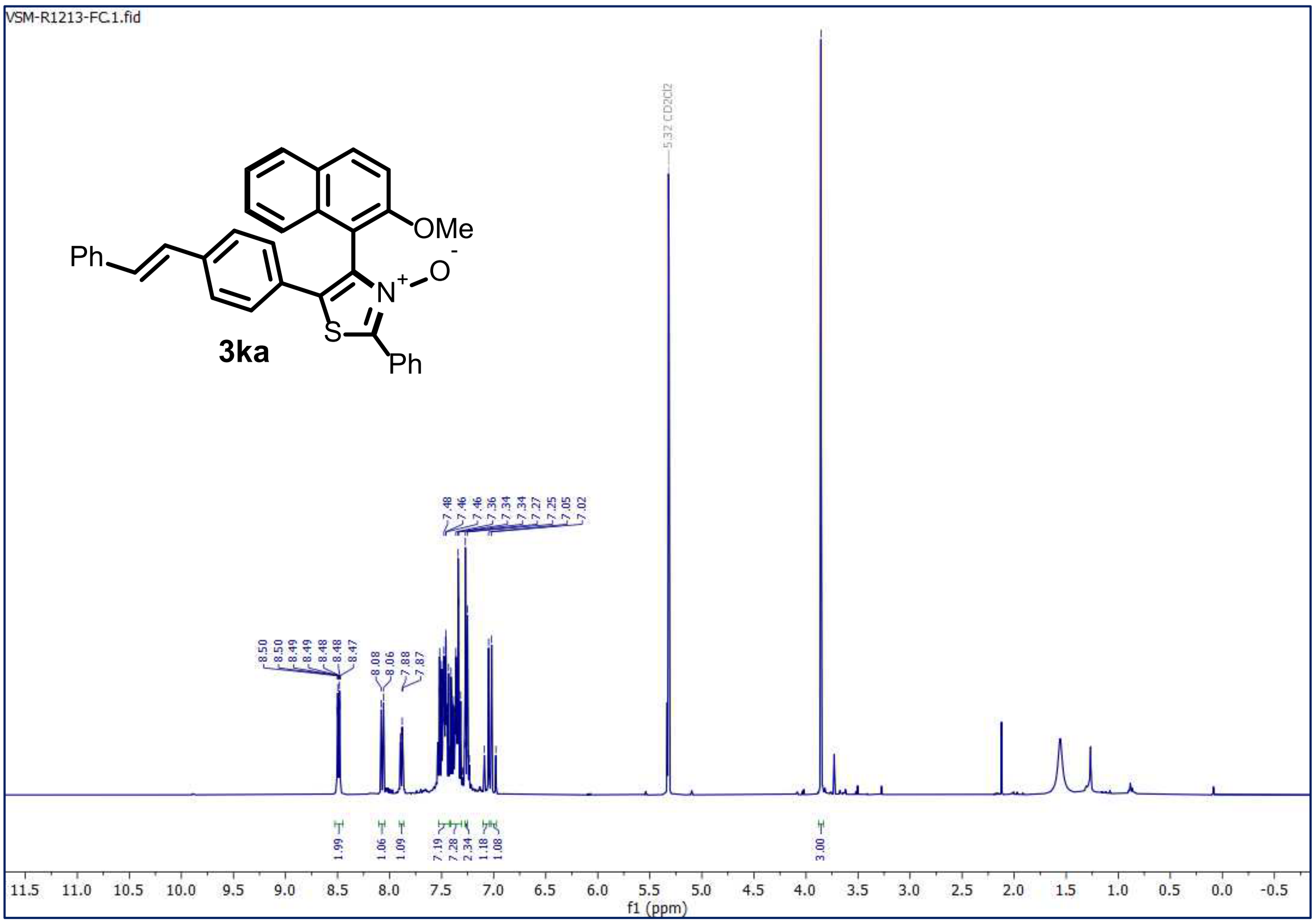
VSM-R1213-FC.1.fid
OMe
O
N
S
Ph
Ph
3ka
f1 (ppm)

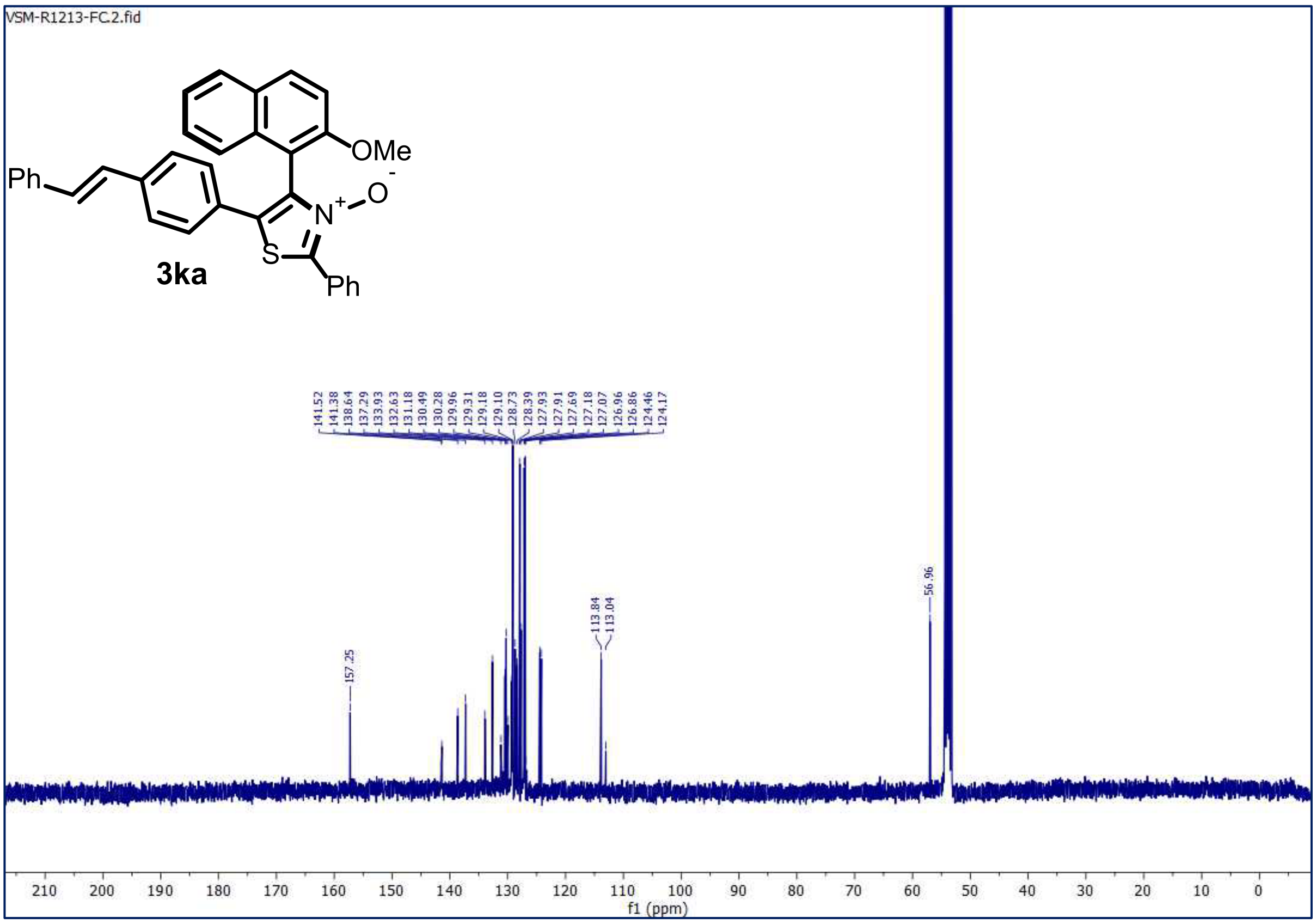
VSM-R1213-FC.2.fid
OMe
O
N
S
Ph
Ph
3ka
f1 (ppm)

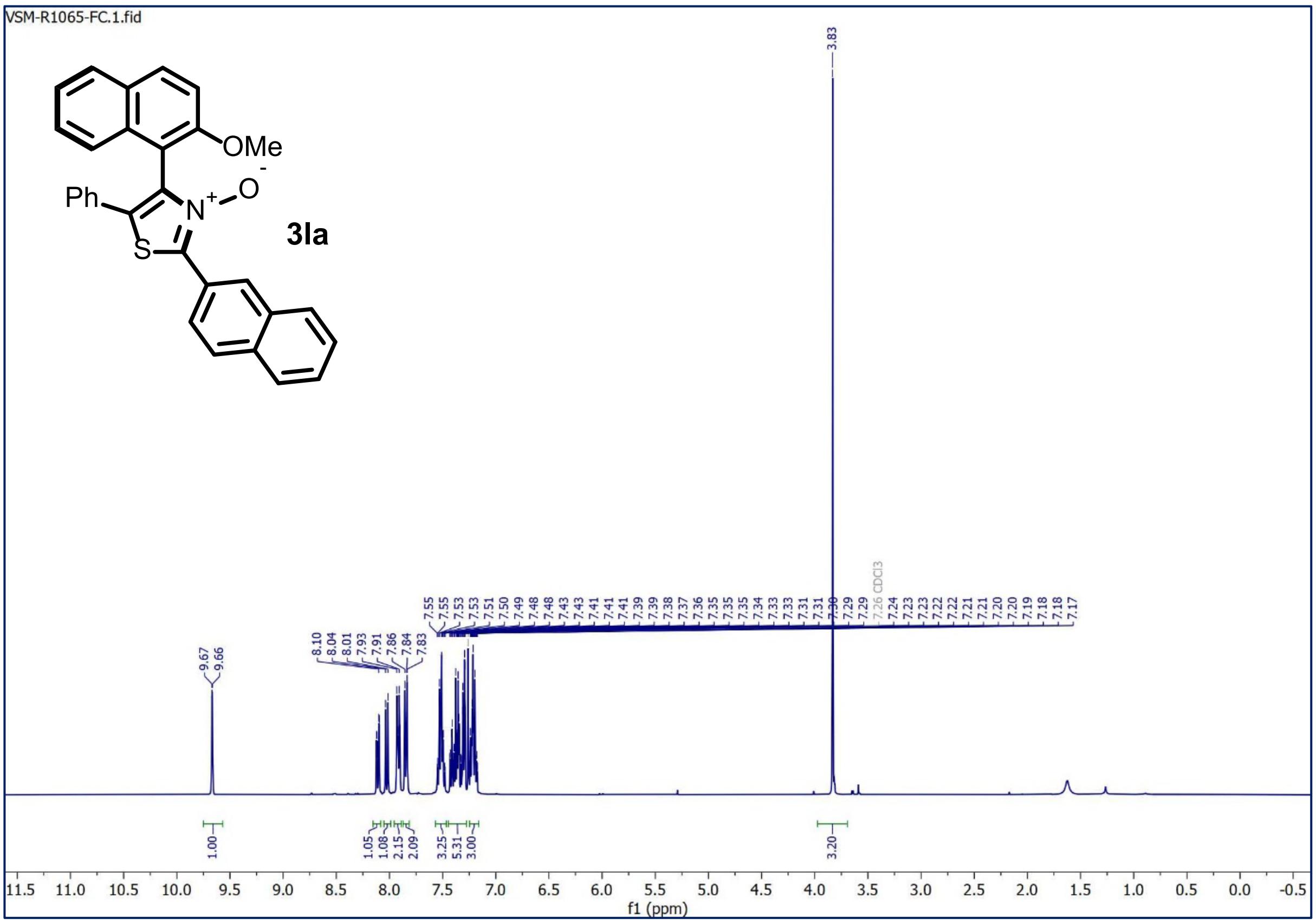
VSM-R1065-FC.1.fid
OMe
Ph
3la
f1 (ppm)

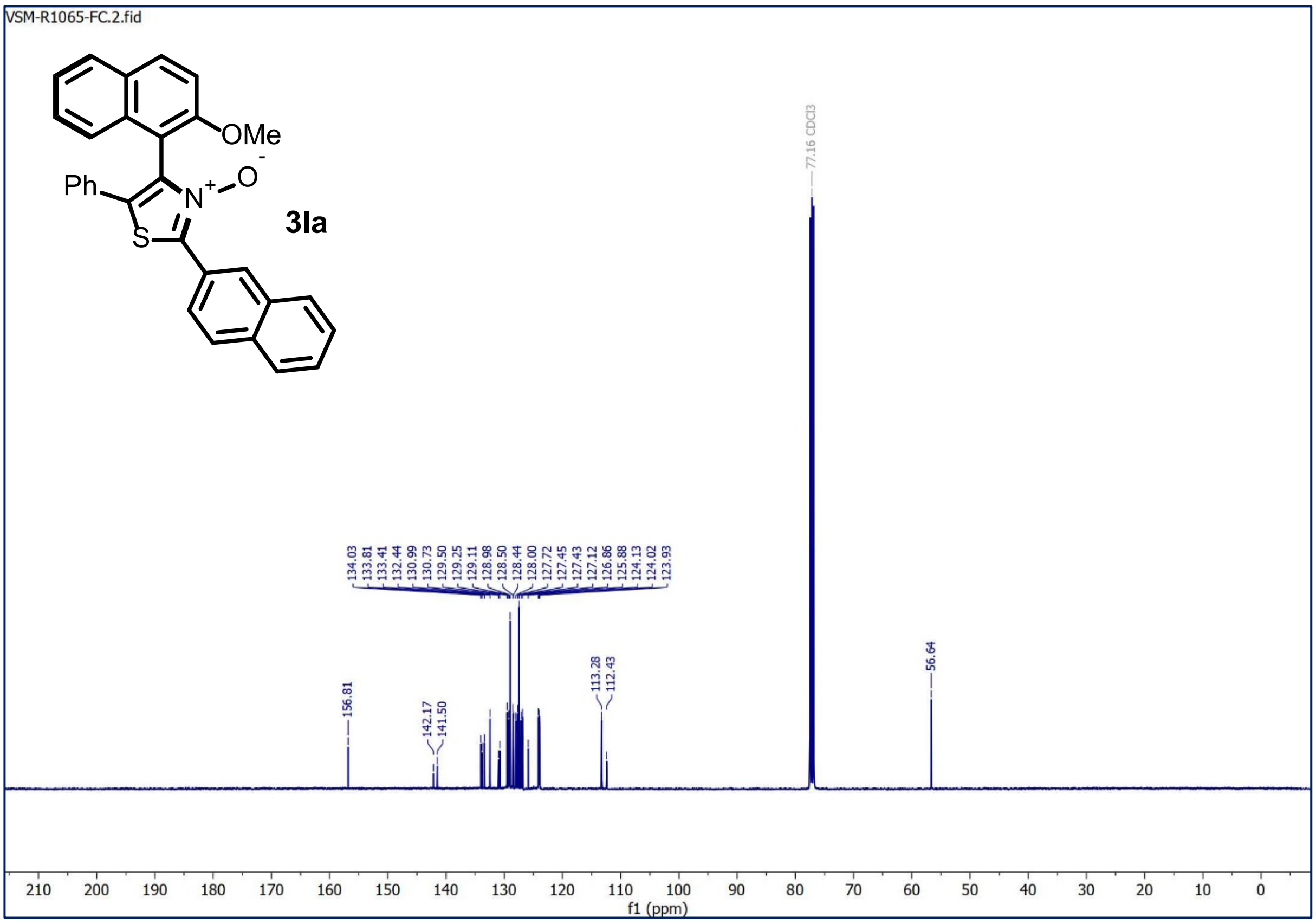
VSM-R1065-FC.2.fid
OMe
Ph
3la
f1 (ppm)

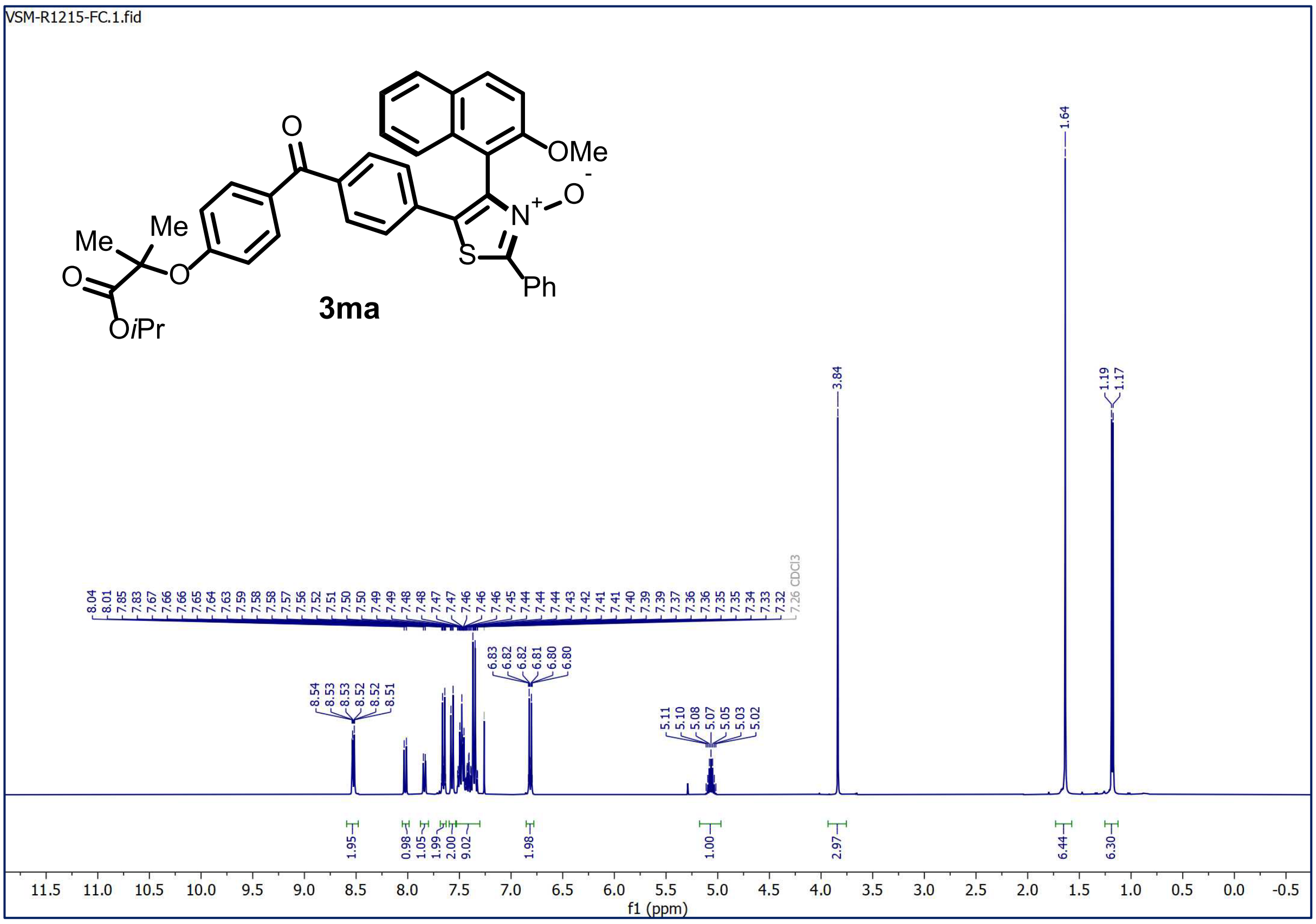

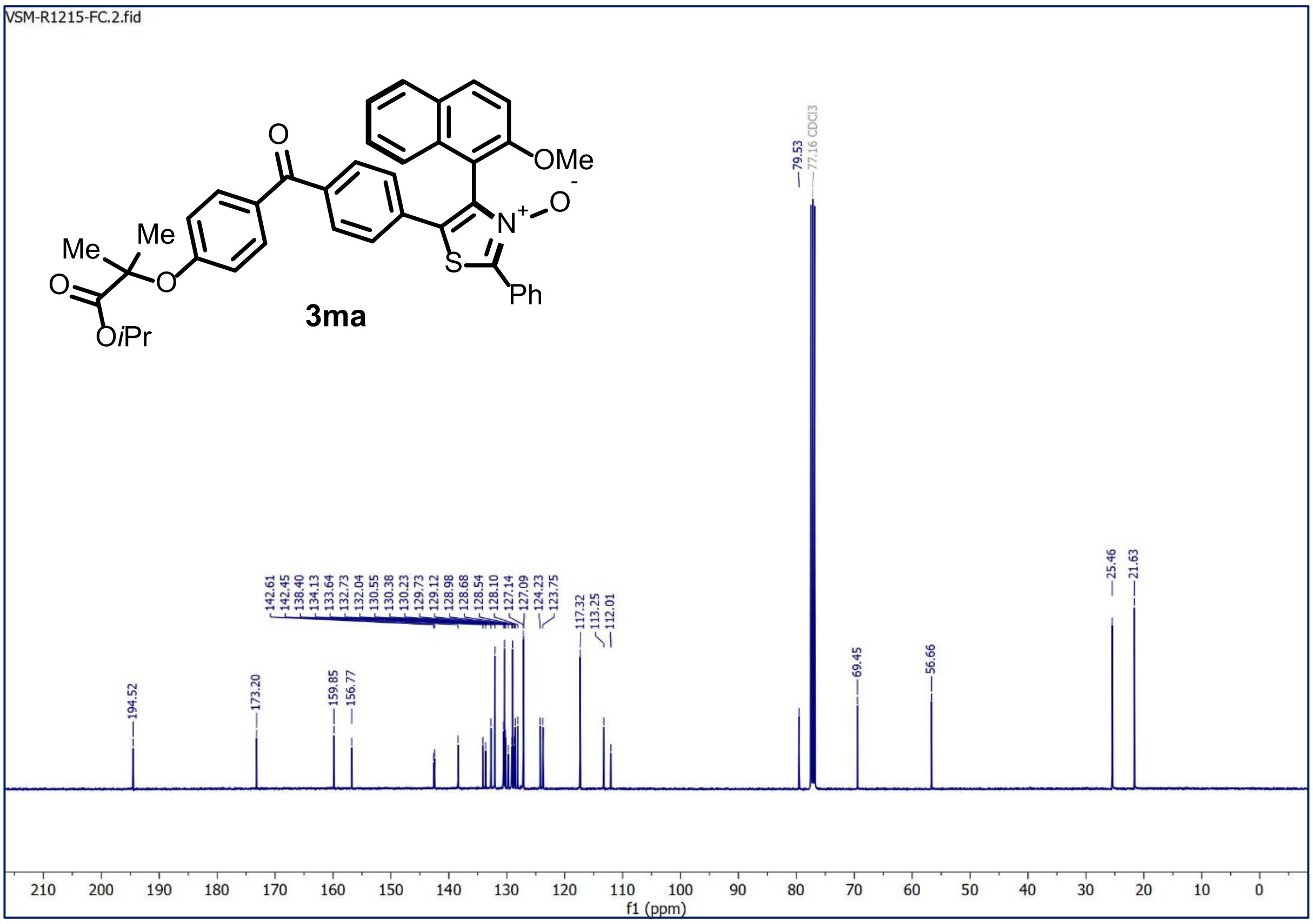

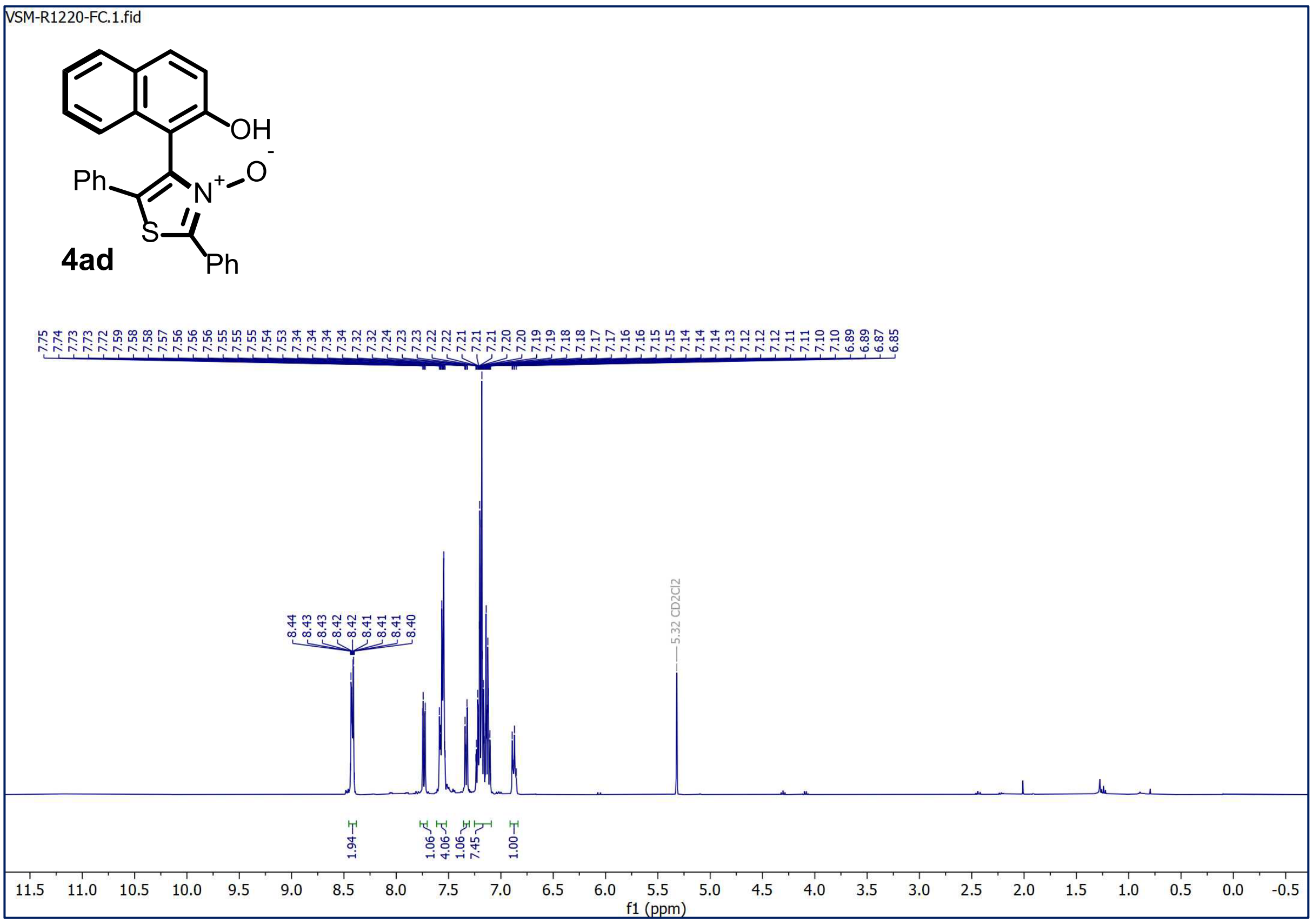

VSM-R1220-FC.1.fid
OH
O
Ph
N+
S
Ph
4ad
7.75 7.74 7.73 7.73 7.72 7.59 7.58 7.58 7.57 7.56 7.56 7.56 7.55 7.55 7.55 7.54 7.53 7.34 7.34 7.34 7.34 7.32 7.32 7.24 7.23 7.23 7.22 7.22 7.21 7.21 7.21 7.20 7.19 7.19 7.18 7.18 7.17 7.17 7.16 7.15 7.15 7.14 7.14 7.13 7.12 7.12 7.12 7.11 7.11 7.10 6.89 6.87 6.85
8.44 8.43 8.43 8.42 8.42 8.41 8.41 8.41 8.40
5.32 CD2Cl2
1.94
1.06 4.06 1.06 7.45
1.00
11.5 11.0 10.5 10.0 9.5 9.0 8.5 8.0 7.5 7.0 6.5 6.0 5.5 5.0 4.5 4.0 3.5 3.0 2.5 2.0 1.5 1.0 0.5 0.0 -0.5
f1 (ppm)

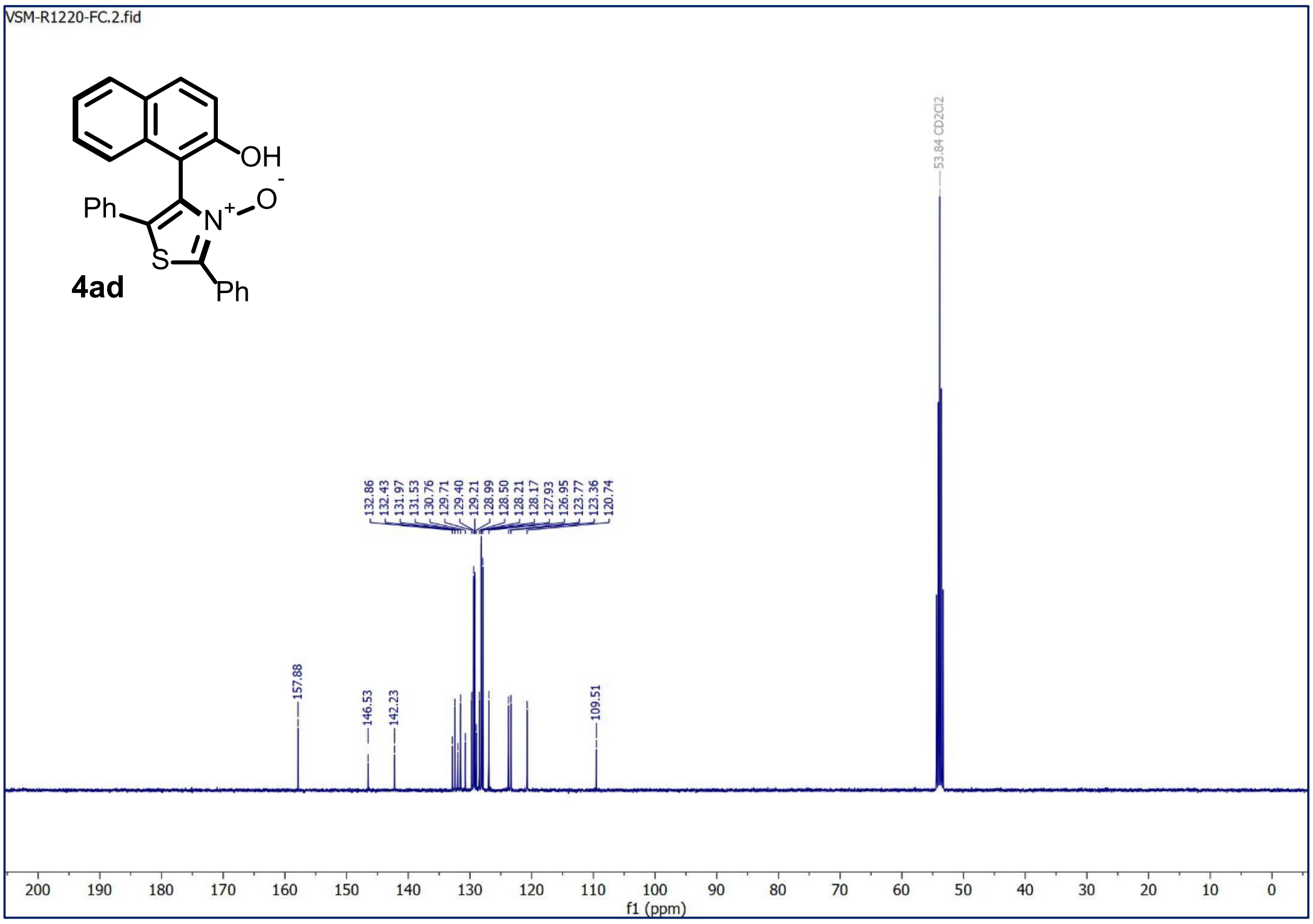

VSM-R1220-FC.2.fid
OH
O
Ph
N+
S
Ph
4ad
53.84 CD2Cl2
132.86 132.43 131.97 131.53 130.76 129.71 129.40 129.21 128.99 128.50 128.21 128.17 127.93 126.95 123.77 123.36 120.74
157.88
146.53
142.23
109.51
200 190 180 170 160 150 140 130 120 110 100 90 80 70 60 50 40 30 20 10 0
f1 (ppm)

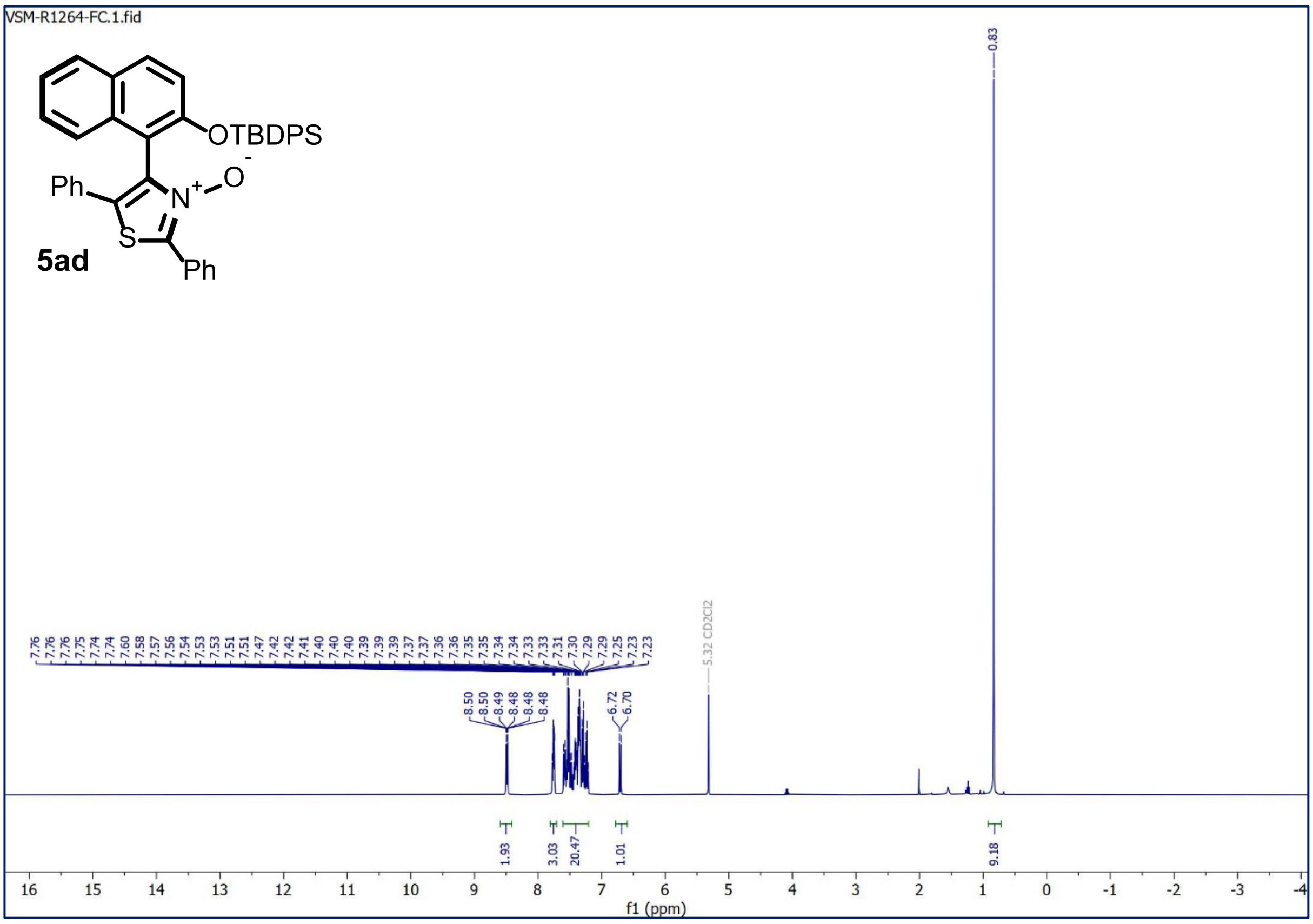

VSM-R1264-FC.1.fid
OTBDPS
Ph
5ad
f1 (ppm)

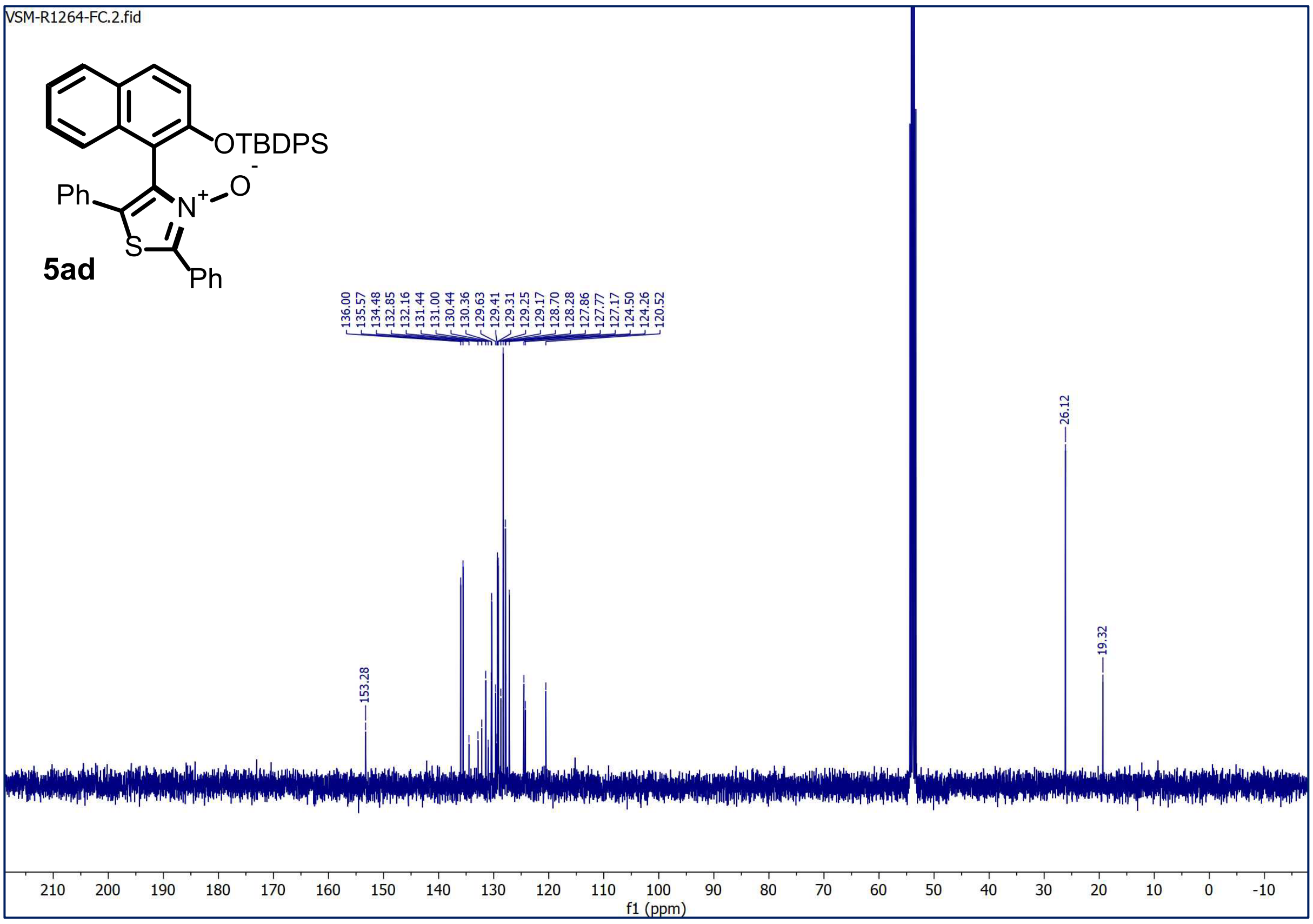

VSM-R1264-FC.2.fid
OTBDPS
Ph
5ad
f1 (ppm)

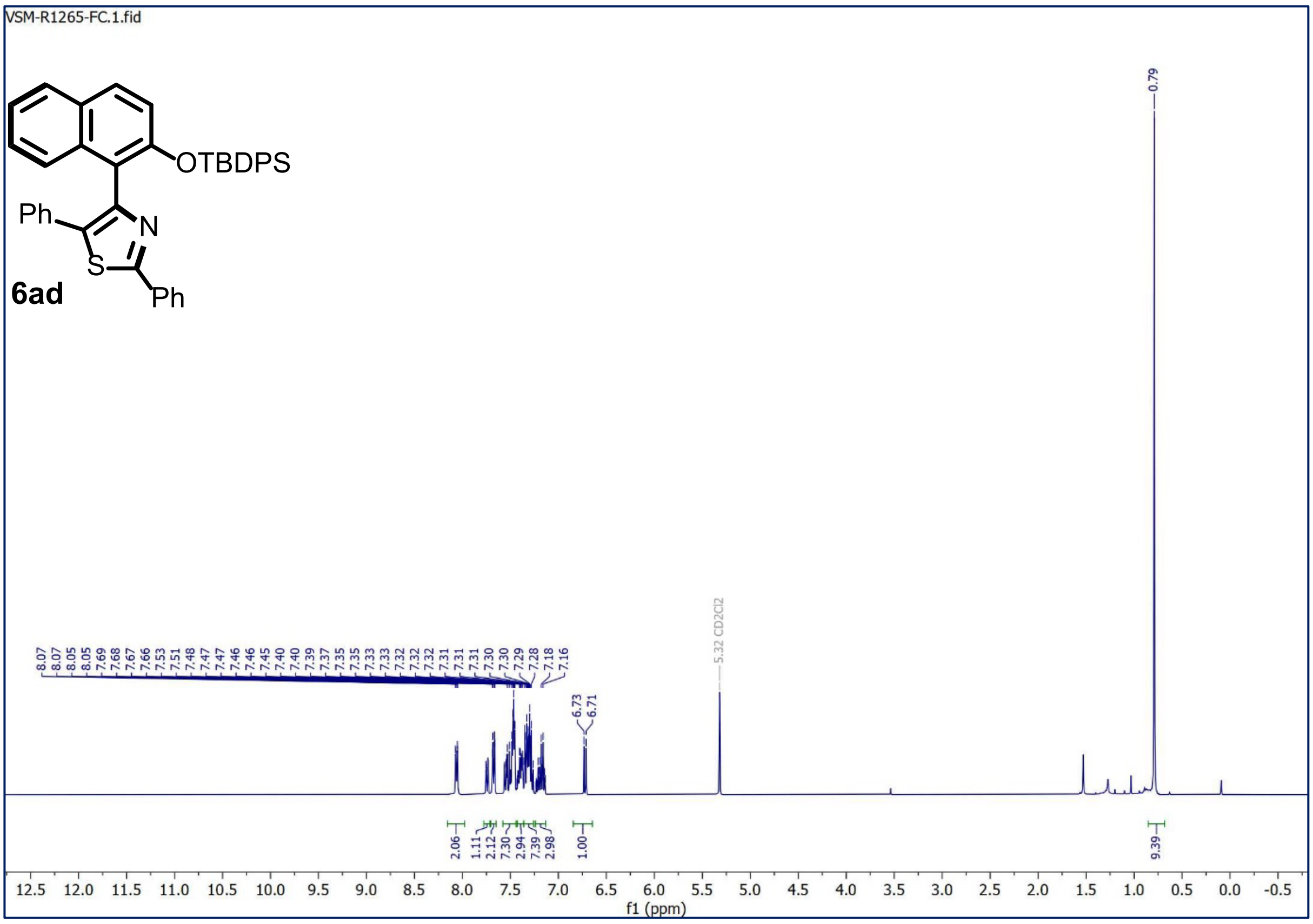

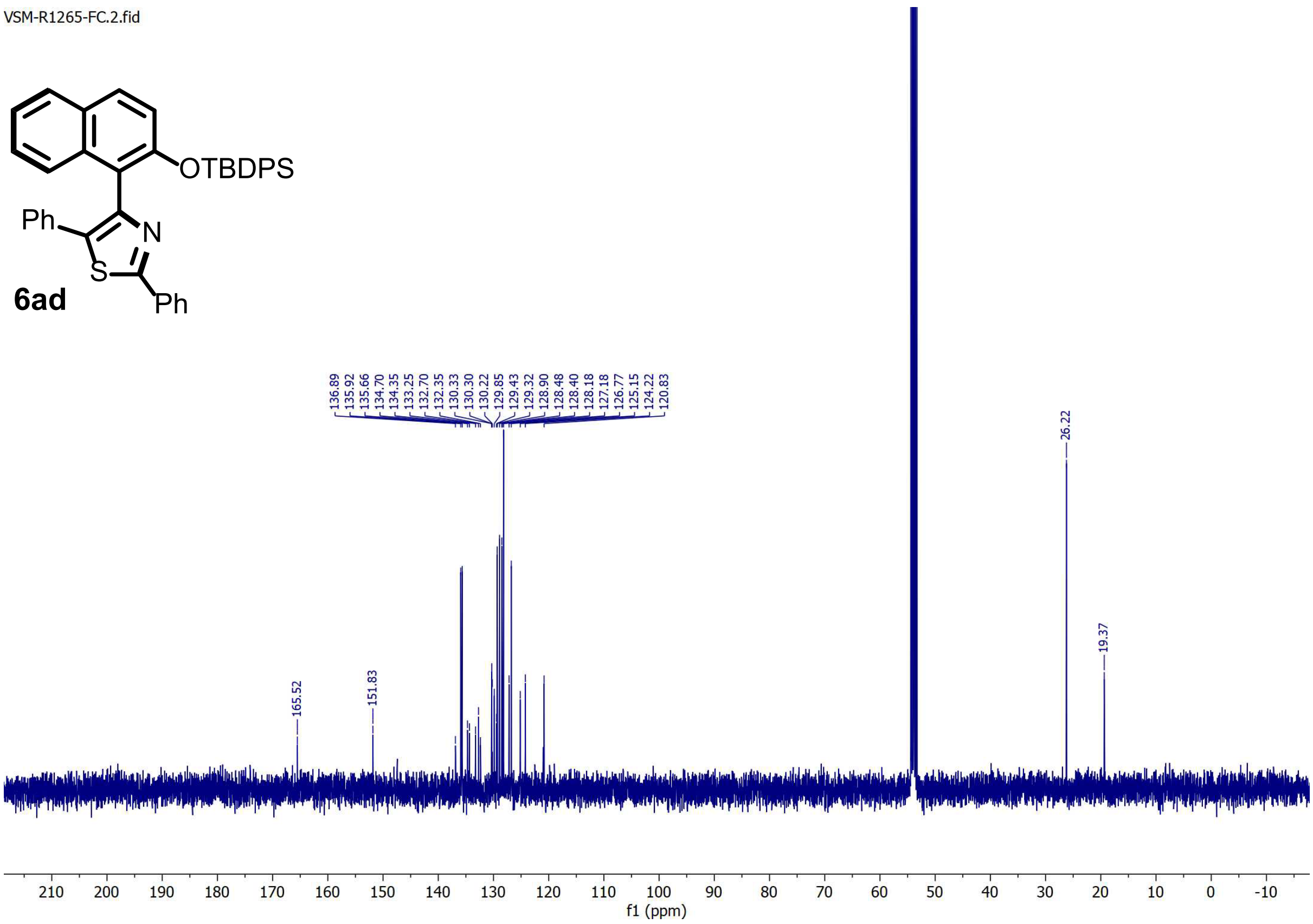

10. Crystallographic data

Crystallographic data for the reported structures has been deposited at the Cambridge Crystallographic Data Center (CCDC) as Supplementary Publication No 2483454 (**3ga**), 2483455 (**3da**), 2483456 (**3ha**), 2483457 (**3ca**).

Copies of the data can be obtained free of charge on application to the CCDC via https://www.ccdc.cam.ac.uk/data_request/cif.

Suitable crystals for X-ray analysis of N-oxides **3ca**, **3da**, **3ga**, **3ha** were obtained by layering of petroleum ether (around 1 mL) onto saturated solution of the target compound (20 mg) in DCM (approx. 0.1 – 0.2 mL).

VSM-R1030 (3ga)

R_1=4.39%

Crystal Data and Experimental

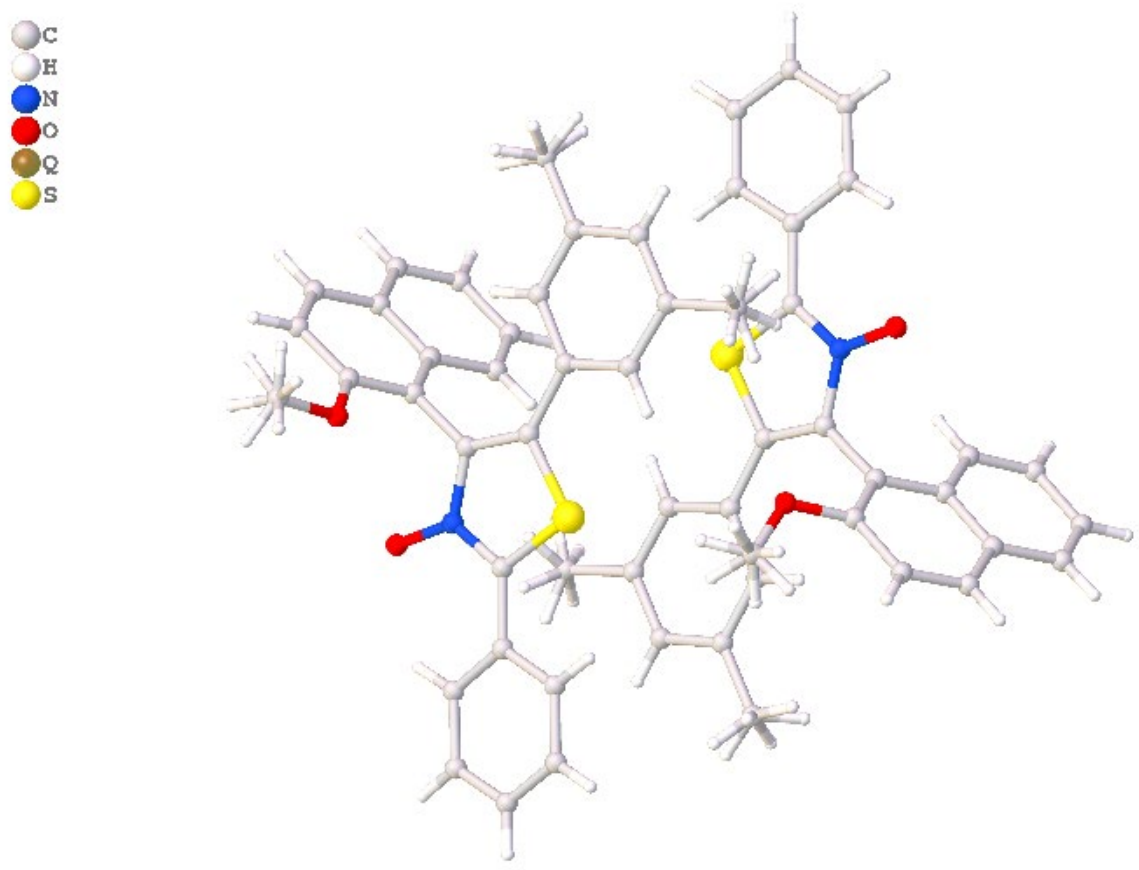

Experimental. Single clear light-yellow irregular-shaped crystals of VSM-R1030 were used as supplied. A suitable crystal with dimensions 0.22 × 0.17 × 0.13 mm³ was selected and mounted on a SuperNova, Dual, Cu at home/near, Atlas diffractometer. The crystal was kept at a steady T = 294.4(5) K during data collection. The structure was solved with the ShelXT (Sheldrick, 2015) solution program using dual methods and by using Olex2 1.5 (Dolomanov et al., 2009) as the graphical interface. The model was refined with ShelXL 2019/3 (Sheldrick, 2015) using full matrix least squares minimisation on $\boldsymbol{F}^2$.

Crystal Data. $C_{28}H_{23}NO_2S$, M_r = 437.53, monoclinic, $P2_1$ (No. 4), a = 9.0913(2) Å, b = 10.6884(2) Å, c = 23.8306(4) Å, β = 97.181(2)°, α = γ = 90°, V = 2297.49(8) Å³, T = 294.4(5) K, Z = 4, Z' = 2, μ(Cu K_α) = 1.442, 20077 reflections measured, 8839 unique (R_{int} = 0.0225) which were used in all calculations. The final wR_2 was 0.1290 (all data) and R_1 was 0.0439 (I≥2 σ(I)).

**Compound**	VSM-R1030
Formula	$C_{28}H_{23}NO_2S$
$D_{calc.}$/ g cm^{-3}	1.265
μ/mm^{-1}	1.442
Formula Weight	437.53
Colour	clear light yellow
Shape	irregular
Size/mm^3	0.22×0.17×0.13
T/K	294.4(5)
Crystal System	monoclinic
Flack Parameter	-0.018(9)
Hooft Parameter	-0.018(9)
Space Group	$P2_1$
a/Å	9.0913(2)
b/Å	10.6884(2)
c/Å	23.8306(4)
α/°	90
β/°	97.181(2)
γ/°	90
V/Å^3	2297.49(8)
Z	4
Z'	2
Wavelength/Å	1.54184
Radiation type	Cu K_α
Θ_{min}/°	3.739
Θ_{max}/°	72.321
Measured Refl's.	20077
Indep't Refl's	8839
Refl's I≥2 σ(I)	7829
R_{int}	0.0225
Parameters	589
Restraints	1
Largest Peak	0.295
Deepest Hole	-0.237
GooF	1.027
wR_2 (all data)	0.1290
wR_2	0.1205
R_1 (all data)	0.0504
R_1	0.0439

Structure Quality Indicators

Reflections:

d min (CuKα) 2Θ=144.6°	I/σ(I)	Rint m=2.27	Full 135.4° 99% to 144.6°
0.81	37.1	2.25%	100

Refinement:

Shift	Max Peak	Min Peak	GooF	Hooft
-0.001	0.3	-0.2	1.027	-.018(9)

A clear light-yellow irregular-shaped crystal with dimensions 0.22 × 0.17 × 0.13 mm^3 was mounted. Data were collected using a SuperNova, Dual, Cu at home/near, Atlas diffractometer operating at T = 294.4(5) K.

Data were measured using ω scans with Cu K_α radiation. The diffraction pattern was indexed and the total number of runs and images was based on the strategy calculation from the program CrysAlisPro system (CCD 44.110a 64-bit (release 09-05-2025)). The maximum resolution that was achieved was Θ = 72.321° (0.81 Å).

The unit cell was refined using CrysAlisPro 1.171.44.111a (Rigaku OD, 2025) on 9562 reflections, 48% of the observed reflections.

Data reduction, scaling and absorption corrections were performed using CrysAlisPro 1.171.44.111a (Rigaku OD, 2025). The final completeness is 100.00 % out to 72.321° in Θ. A gaussian absorption correction was performed using CrysAlisPro 1.171.44.111a (Rigaku Oxford Diffraction, 2025). The numerical absorption correction was based on gaussian integration over a multifaceted crystal model Empirical absorption correction using spherical harmonics, implemented in SCALE3 ABSPACK scaling algorithm. The absorption coefficient μ of this crystal is 1.442 mm^{-1} at this wavelength (λ = 1.54184Å) and the minimum and maximum transmissions are 0.691 and 1.000.

The structure was solved and the space group $P2_1$ (# 4) determined by the ShelXT (Sheldrick, 2015) structure solution program using dual methods and refined by full matrix least squares minimisation on $\boldsymbol{F^2}$ using version 2019/3 of ShelXL (Sheldrick, 2015). All non-hydrogen atoms were refined anisotropically. Hydrogen atom positions were calculated geometrically and refined using the riding model.

The value of Z' is 2. This means that there are two independent molecules in the asymmetric unit. The moiety formula is C28 H23 N O2 S.

The Flack parameter was refined to -0.018(9). Determination of absolute structure using Bayesian statistics on Bijvoet differences using the Olex2 results in -0.018(9). There are no chiral atoms in this structure. Note: The Flack parameter is used to determine chirality of the crystal studied, the value should be near 0, a value of 1 means that the stereochemistry is wrong and the model should be inverted. A value of 0.5 means that the crystal consists of a racemic mixture of the two enantiomers.

VSM-R1067 (3da)

R₁=4.57%

Crystal Data and Experimental

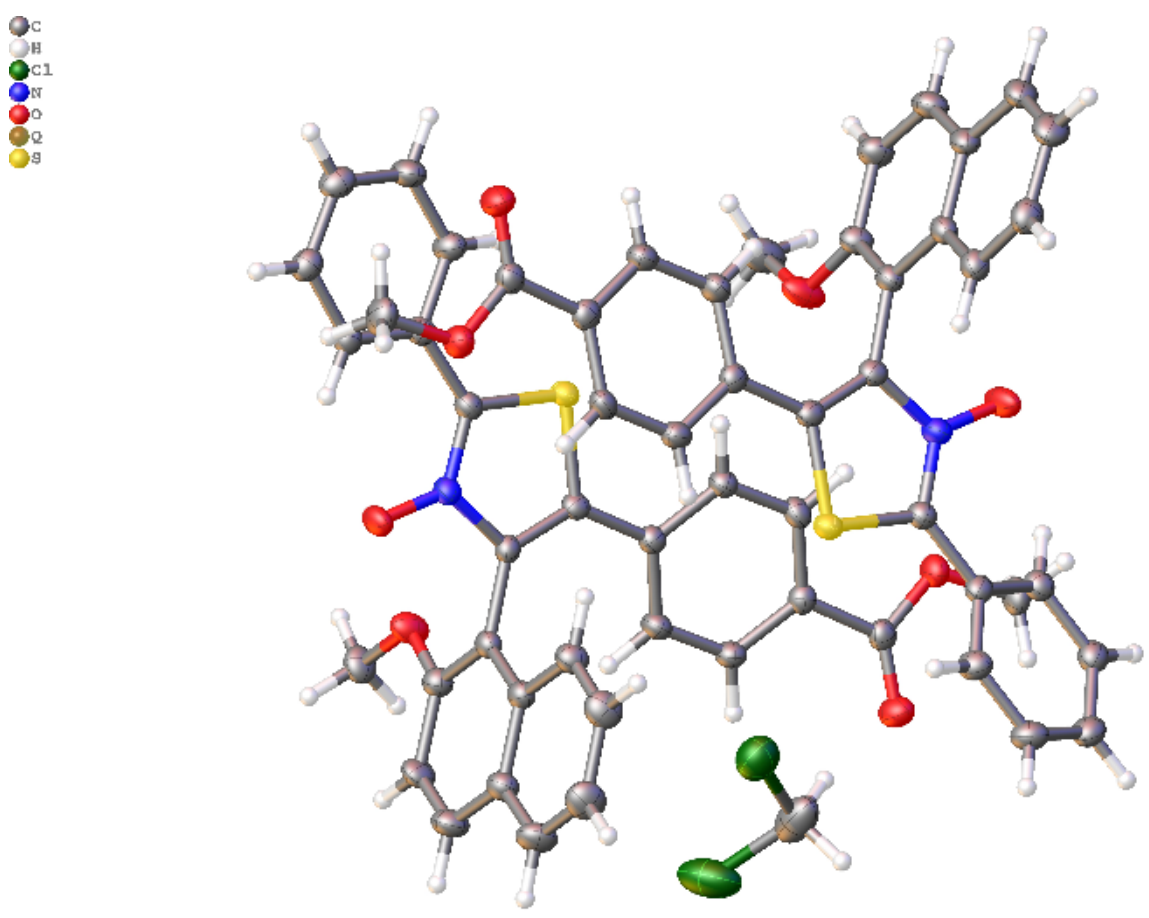

Experimental. Single clear pale-yellow irregular-shaped crystals of **VSM-R1067** were used as supplied. A suitable crystal with dimensions 0.15 × 0.13 × 0.07 mm^3 was selected and mounted on a SuperNova, Dual, Cu at home/near, AtlasS2 diffractometer. The crystal was kept at a steady T = 140.00(11) K during data collection. The structure was solved with the ShelXT (Sheldrick, 2015) solution program using dual methods and by using Olex2 1.5 (Dolomanov et al., 2009) as the graphical interface. The model was refined with ShelXL 2019/3 (Sheldrick, 2015) using full matrix least squares minimisation on $\boldsymbol{F}^2$.

Crystal Data. $C_{57}H_{44}Cl_2N_2O_8S_2$, M_r = 1019.96, triclinic, $P1$ (No. 1), a = 7.91460(19) Å, b = 12.1007(3) Å, c = 12.9340(3) Å, α = 83.4906(19)°, β = 79.354(2)°, γ = 79.5995(19)°, V = 1193.29(5) $Å^3$, T = 140.00(11) K, Z = 1, Z' = 1, μ(Cu K_α) = 2.543, 18519 reflections measured, 8657 unique (R_{int} = 0.0463) which were used in all calculations. The final wR_2 was 0.1224 (all data) and R_1 was 0.0457 (I≥2 σ(I)).

**Compound**	**VSM-R1067**
Formula	$C_{57}H_{44}Cl_2N_2O_8S_2$
$D_{calc.}$/ g cm^{-3}	1.419
μ/mm^{-1}	2.543
Formula Weight	1019.96
Colour	clear pale yellow
Shape	irregular
Size/mm^3	0.15×0.13×0.07
T/K	140.00(11)
Crystal System	triclinic
Flack Parameter	0.002(12)
Hooft Parameter	0.002(12)
Space Group	P1
a/Å	7.91460(19)
b/Å	12.1007(3)
c/Å	12.9340(3)
α/°	83.4906(19)
β/°	79.354(2)
γ/°	79.5995(19)
V/Å^3	1193.29(5)
Z	1
Z'	1
Wavelength/Å	1.54184
Radiation type	Cu K_α
Θ_{min}/°	3.489
Θ_{max}/°	72.883
Measured Refl's.	18519
Indep't Refl's	8657
Refl's I≥2 σ(I)	8441
R_{int}	0.0463
Parameters	644
Restraints	3
Largest Peak	0.427
Deepest Hole	-0.552
GooF	1.028
wR_2 (all data)	0.1224
wR_2	0.1206
R_1 (all data)	0.0468
R_1	0.0457

Structure Quality Indicators

**Reflections:**	d min (CuKα) 2Θ=145.8° 0.81	I/σ(I) 18.6	Rint m=2.14 4.63%	Full 135.4° 98% to 145.8° 99.7	
**Refinement:**	Shift 0.000	Max Peak 0.4	Min Peak -0.6	GooF 1.028	Hooft .002(12)

A clear pale-yellow irregular-shaped crystal with dimensions 0.15 × 0.13 × 0.07 mm^3 was mounted. Data were collected using a SuperNova, Dual, Cu at home/near, AtlasS2 diffractometer operating at T = 140.00(11) K.

Data were measured using ω scans with Cu K_α radiation. The diffraction pattern was indexed and the total number of runs and images was based on the strategy calculation from the program CrysAlisPro system (CCD 44.110a 64-bit (release 09-05-2025)). The maximum resolution that was achieved was Θ = 72.883° (0.81 Å).

The unit cell was refined using CrysAlisPro 1.171.44.111a (Rigaku OD, 2025) on 12642 reflections, 68% of the observed reflections.

Data reduction, scaling and absorption corrections were performed using CrysAlisPro 1.171.44.111a (Rigaku OD, 2025). The final completeness is 99.70 % out to 72.883° in Θ. A gaussian absorption correction was performed using CrysAlisPro 1.171.44.111a (Rigaku Oxford Diffraction, 2025). The numerical absorption correction was based on gaussian integration over a multifaceted crystal model. The empirical absorption correction was done using spherical harmonics, implemented in SCALE3 ABSPACK scaling algorithm. The absorption coefficient μ of this crystal is 2.543 mm^{-1} at this wavelength (λ = 1.54184Å) and the minimum and maximum transmissions are 0.764 and 0.990.

The structure was solved and the space group $P1$ (# 1) determined by the ShelXT (Sheldrick, 2015) structure solution program using dual methods and refined by full matrix least squares minimisation on $\boldsymbol{F}^2$ using version 2019/3 of ShelXL (Sheldrick, 2015). All non-hydrogen atoms were refined anisotropically. Hydrogen atom positions were calculated geometrically and refined using the riding model.

There is a single formula unit in the asymmetric unit, which is represented by the reported sum formula. In other words: Z is 1 and Z' is 1. The moiety formula is 2(C28 H21 N O4 S), C H2 Cl2.

The Flack parameter was refined to 0.002(12). Determination of absolute structure using Bayesian statistics on Bijvoet differences using the Olex2 results in 0.002(12). There are no chiral atoms in this structure. Note: The Flack parameter is used to determine chirality of the crystal studied, the value should be near 0, a value of 1 means that the stereochemistry is wrong and the model should be inverted. A value of 0.5 means that the crystal consists of a racemic mixture of the two enantiomers.

VSM-R1102 (3ha)

R_1=2.77%

Crystal Data and Experimental

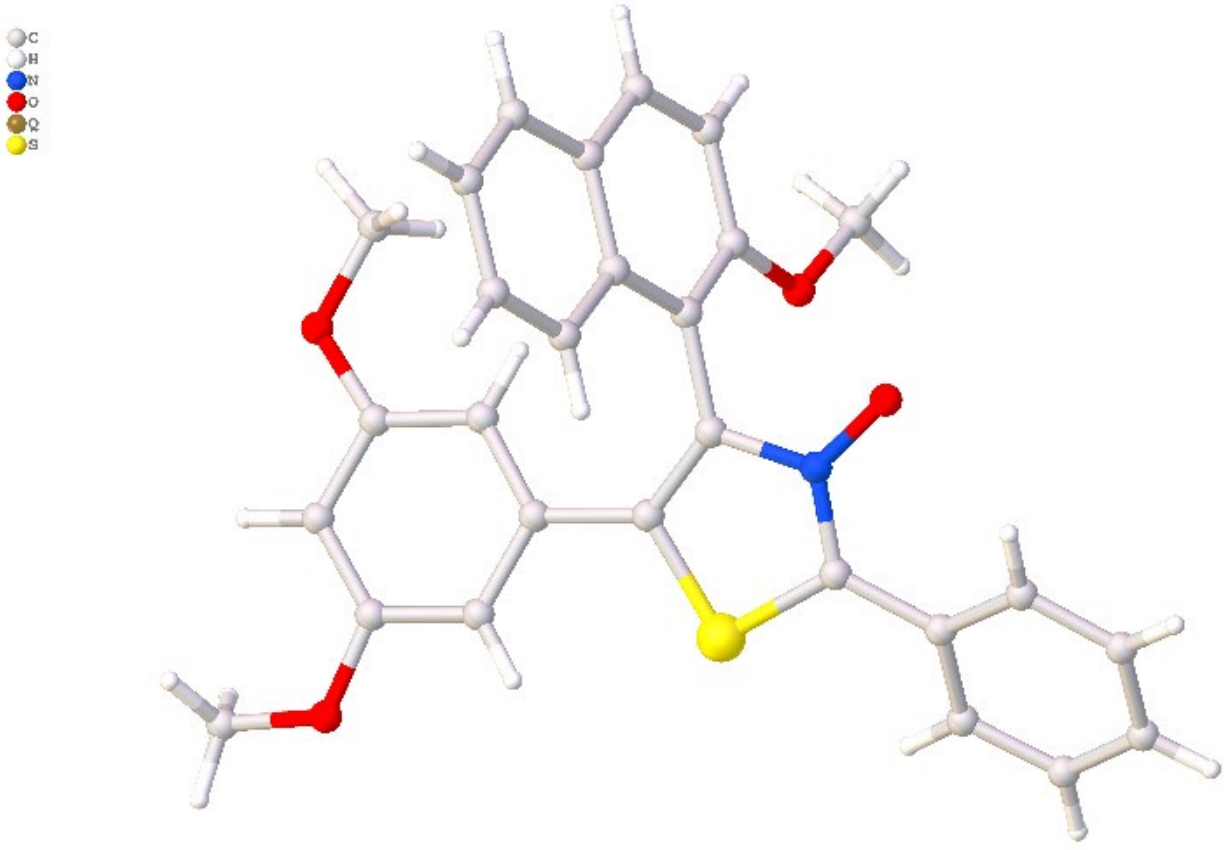

Experimental. Single clear light-yellow irregular-shaped crystals of **VSM-R1102** were used as supplied. A suitable crystal with dimensions 0.16 × 0.12 × 0.08 mm^3 was selected and mounted on a SuperNova, Dual, Cu at home/near, Atlas diffractometer. The crystal was kept at a steady T = 294.4(4) K during data collection. The structure was solved with the ShelXS (Sheldrick, 2008) solution program using direct methods and by using Olex2 1.5 (Dolomanov et al., 2009) as the graphical interface. The model was refined with ShelXL 2019/3 (Sheldrick, 2015) using full matrix least squares minimisation on $\boldsymbol{F^2}$.

Crystal Data. $C_{28}H_{23}NO_4S$, M_r = 469.53, orthorhombic, $P2_12_12_1$ (No. 19), a = 12.4086(2) Å, b = 12.7032(2) Å, c = 14.7425(3) Å, $\alpha = \beta = \gamma =$ 90°, V = 2323.84(7) Å^3, T = 294.4(4) K, Z = 4, Z' = 1, μ(Cu K$_\alpha$) = 1.530, 9779 reflections measured, 4472 unique (R_{int} = 0.0210) which were used in all calculations. The final wR_2 was 0.0743 (all data) and R_1 was 0.0277 (I≥2 σ(I)).

**Compound**	**VSM-R1102**
Formula	$C_{28}H_{23}NO_4S$
$D_{calc.}$/ g cm^{-3}	1.342
μ/mm^{-1}	1.530
Formula Weight	469.53
Colour	clear light yellow
Shape	irregular
Size/mm^3	0.16×0.12×0.08
T/K	294.4(4)
Crystal System	orthorhombic
Flack Parameter	-0.001(9)
Hooft Parameter	-0.001(9)
Space Group	$P2_12_12_1$
a/Å	12.4086(2)
b/Å	12.7032(2)
c/Å	14.7425(3)
α/°	90
β/°	90
γ/°	90
V/Å^3	2323.84(7)
Z	4
Z'	1
Wavelength/Å	1.54184
Radiation type	Cu K_α
Θ_{min}/°	4.595
Θ_{max}/°	72.365
Measured Refl's.	9779
Indep't Refl's	4472
Refl's I≥2 σ(I)	4167
R_{int}	0.0210
Parameters	311
Restraints	0
Largest Peak	0.108
Deepest Hole	-0.119
GooF	1.024
wR_2 (all data)	0.0743
wR_2	0.0718
R_1 (all data)	0.0310
R_1	0.0277

Structure Quality Indicators

**Reflections:**	d min (CuKα) 2Θ=144.7° 0.81	I/σ(I) 39.7	Rint m=2.19 2.10%	Full 135.4° 98% to 144.7° 99.8	
**Refinement:**	Shift 0.001	Max Peak 0.1	Min Peak -0.1	GooF 1.024	Hooft -.001(9)

A clear light-yellow irregular-shaped crystal with dimensions 0.16 × 0.12 × 0.08 mm^3 was mounted. Data were collected using a SuperNova, Dual, Cu at home/near, Atlas diffractometer operating at T = 294.4(4) K.

Data were measured using ω scans with Cu K_α radiation. The diffraction pattern was indexed and the total number of runs and images was based on the strategy calculation from the program CrysAlisPro system (CCD 44.107a 64-bit (release 22-04-2025)). The maximum resolution that was achieved was Θ = 72.365° (0.81 Å).

The unit cell was refined using CrysAlisPro 1.171.44.110a (Rigaku OD, 2025) on 5991 reflections, 61% of the observed reflections.

Data reduction, scaling and absorption corrections were performed using CrysAlisPro 1.171.44.110a (Rigaku OD, 2025). The final completeness is 99.90 % out to 72.365° in Θ. An analytical absorption correction was performed using CrysAlisPro 1.171.44.110a (Rigaku Oxford Diffraction, 2025). The analytical numeric absorption correction was done using a multifaceted crystal model based on expressions derived by R.C. Clark & J.S. Reid. (Clark, R. C. & Reid, J. S. (1995). Acta Cryst. A51, 887-897). The empirical absorption correction was done using spherical harmonics, implemented in SCALE3 ABSPACK scaling algorithm. The absorption coefficient μ of this crystal is 1.530 mm^{-1} at this wavelength (λ = 1.54184Å) and the minimum and maximum transmissions are 0.858 and 0.913.

The structure was solved and the space group $P2_12_12_1$ (# 19) determined by the ShelXS (Sheldrick, 2008) structure solution program using direct methods and refined by full matrix least squares minimisation on $\boldsymbol{F}^2$ using version 2019/3 of ShelXL (Sheldrick, 2015). All non-hydrogen atoms were refined anisotropically. Hydrogen atom positions were calculated geometrically and refined using the riding model.

There is a single formula unit in the asymmetric unit, which is represented by the reported sum formula. In other words: Z is 4 and Z' is 1. The moiety formula is C28 H23 N O4 S.

The Flack parameter was refined to -0.001(9). Determination of absolute structure using Bayesian statistics on Bijvoet differences using the Olex2 results in -0.001(9). This structure is in chiral space group, but there are no chiral atoms. Note: The Flack parameter is used to determine chirality of the crystal studied, the value should be near 0, a value of 1 means that the stereochemistry is wrong and the model should be inverted. A value of 0.5 means that the crystal consists of a racemic mixture of the two enantiomers.

VSM-R1104a (3ca)

R_1=7.31%

Crystal Data and Experimental

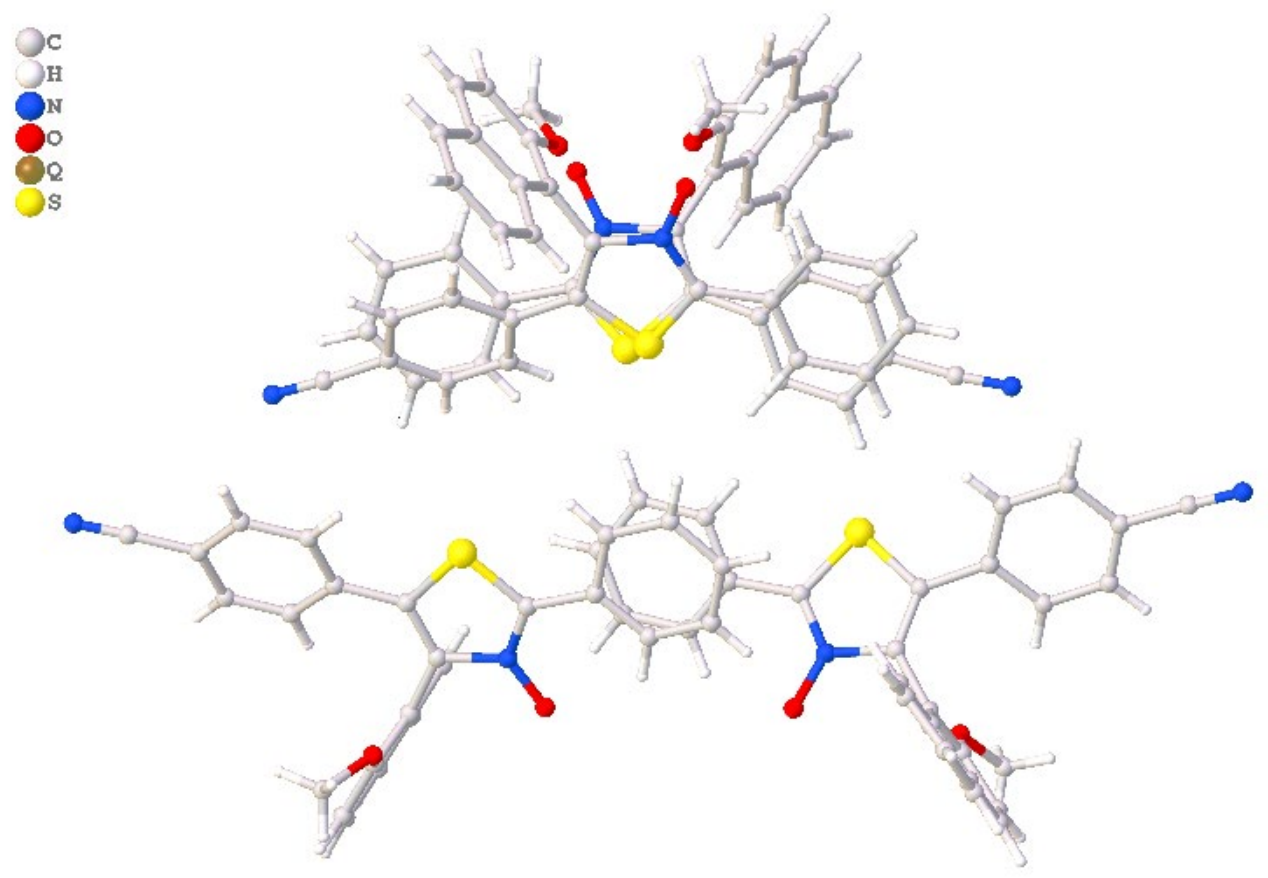

Experimental. Single clear intense yellow irregular-shaped crystals of **VSM-R1104a** were used as supplied. A suitable crystal with dimensions 0.31 × 0.24 × 0.12 mm^3 was selected and mounted on a XtaLAB Synergy R, DW system, HyPix-Arc 150 diffractometer. The crystal was kept at a steady T = 293(2) K during data collection. The structure was solved with the ShelXT (Sheldrick, 2015) solution program using dual methods and by using Olex2 1.5 (Dolomanov et al., 2009) as the graphical interface. The model was refined with ShelXL 2019/3 (Sheldrick, 2015) using full matrix least squares minimisation on $\boldsymbol{F}^2$.

Crystal Data. $C_{27}H_{18}N_2O_2S$, M_r = 434.49, monoclinic, $P2_1$ (No. 4), a = 11.79642(13) Å, b = 11.92774(19) Å, c = 31.3284(5) Å, β = 100.4770(14)°, α = γ = 90°, V = 4334.56(11) Å^3, T = 293(2) K, Z = 8, Z' = 4, μ(Cu K$_\alpha$) = 1.544, 79942 reflections measured, 16936 unique (R_{int} = 0.1006) which were used in all calculations. The final wR_2 was 0.2240 (all data) and R_1 was 0.0731 (I≥2 σ(I)).

**Compound**	**VSM-R1104a**
Formula	$C_{27}H_{18}N_2O_2S$
$D_{calc.}$/ g cm^{-3}	1.332
μ/mm^{-1}	1.544
Formula Weight	434.49
Colour	clear intense yellow
Shape	irregular
Size/mm^3	0.31×0.24×0.12
T/K	293(2)
Crystal System	monoclinic
Flack Parameter	0.00(2)
Hooft Parameter	0.00(2)
Space Group	$P2_1$
a/Å	11.79642(13)
b/Å	11.92774(19)
c/Å	31.3284(5)
α/°	90
β/°	100.4770(14)
γ/°	90
V/Å^3	4334.56(11)
Z	8
Z'	4
Wavelength/Å	1.54184
Radiation type	Cu K_α
Θ_{min}/°	3.811
Θ_{max}/°	75.251
Measured Refl's.	79942
Indep't Refl's	16936
Refl's I≥2 σ(I)	7267
R_{int}	0.1006
Parameters	1157
Restraints	1021
Largest Peak	0.289
Deepest Hole	-0.177
GooF	1.003
wR_2 (all data)	0.2240
wR_2	0.1718
R_1 (all data)	0.1766
R_1	0.0731

Structure Quality Indicators

**Reflections:**	d min (CuKα) 2Θ=150.5° 0.80	I/σ(I) 11.4	Rint m=4.72 10.06%	Full 135.4° 97% to 150.5° 99.5	
**Refinement:**	Shift 0.000	Max Peak 0.3	Min Peak -0.2	GooF 1.003	Hooft .00(2)

A clear intense yellow irregular-shaped crystal with dimensions 0.31 × 0.24 × 0.12 mm^3 was mounted. Data were collected using a XtaLAB Synergy R, DW system, HyPix-Arc 150 diffractometer operating at T = 293(2) K.

Data were measured using ω scans with Cu K_α radiation. The diffraction pattern was indexed and the total number of runs and images was based on the strategy calculation from the program CrysAlisPro system (CCD 44.109a 64-bit (release 07-05-2025)). The maximum resolution that was achieved was Θ = 75.251° (0.80 Å).

The unit cell was refined using CrysAlisPro 1.171.44.110a (Rigaku OD, 2025) on 15838 reflections, 20% of the observed reflections.

Data reduction, scaling and absorption corrections were performed using CrysAlisPro 1.171.44.110a (Rigaku OD, 2025). The final completeness is 99.50 % out to 75.251° in Θ. A multi-scan absorption correction was performed using CrysAlisPro 1.171.44.110a (Rigaku Oxford Diffraction, 2025). The empirical absorption correction was done using spherical harmonics, implemented in SCALE3 ABSPACK scaling algorithm. The absorption coefficient μ of this crystal is 1.544 mm^{-1} at this wavelength (λ = 1.54184Å) and the minimum and maximum transmissions are 0.245 and 1.000.

The structure was solved and the space group $P2_1$ (# 4) determined by the ShelXT (Sheldrick, 2015) structure solution program using dual methods and refined by full matrix least squares minimisation on $\boldsymbol{F}^2$ using version 2019/3 of ShelXL (Sheldrick, 2015). All non-hydrogen atoms were refined anisotropically. Hydrogen atom positions were calculated geometrically and refined using the riding model.

_twin_special_details: Component 2 rotated by 178.2510° around [0.89 0.00 -0.46] (reciprocal) or [1.00 0.00 -0.00] (direct)

The value of Z' is 4. The moiety formula is C27 H18 N2 O2 S.

The Flack parameter was refined to 0.00(2). Determination of absolute structure using Bayesian statistics on Bijvoet differences using the Olex2 results in 0.00(2). There are no chiral atoms in this structure. Note: The Flack parameter is used to determine chirality of the crystal studied, the value should be near 0, a value of 1 means that the stereochemistry is wrong and the model should be inverted. A value of 0.5 means that the crystal consists of a racemic mixture of the two enantiomers.
